# Ammonia Inhibition of Anaerobic Volatile Fatty Acid Degrading Microbial Communities

**DOI:** 10.3389/fmicb.2018.02921

**Published:** 2018-11-30

**Authors:** Fabian Bonk, Denny Popp, Sören Weinrich, Heike Sträuber, Sabine Kleinsteuber, Hauke Harms, Florian Centler

**Affiliations:** ^1^Department of Environmental Microbiology, UFZ–Helmholtz Center for Environmental Research, Leipzig, Germany; ^2^Biochemical Conversion Department, DBFZ-Deutsches Biomasseforschungszentrum gGmbH, Leipzig, Germany

**Keywords:** anaerobic digestion, biogas, methanogens, syntrophic propionate oxidation, 16S rRNA amplicon sequencing, T-RFLP, ADM1, chemostat

## Abstract

Ammonia inhibition is an important reason for reactor failures and economic losses in anaerobic digestion. Its impact on acetic acid degradation is well-studied, while its effect on propionic and butyric acid degradation has received little attention and is consequently not considered in the Anaerobic Digestion Model No. 1 (ADM1). To compare ammonia inhibition of the degradation of these three volatile fatty acids (VFAs), we fed a mixture of them as sole carbon source to three continuous stirred tank reactors (CSTRs) and increased ammonium bicarbonate concentrations in the influent from 52 to 277 mM. The use of this synthetic substrate allowed for the determination of degradation efficiencies for the individual acids. While butyric acid degradation was hardly affected by the increase of ammonia concentration, propionic acid degradation turned out to be even more inhibited than acetic acid degradation with degradation efficiencies dropping to 31 and 65% for propionic and acetic acid, respectively. The inhibited reactors acclimatized and approximated pre-disturbance degradation efficiencies toward the end of the experiment, which was accompanied by strong microbial community shifts, as observed by amplicon sequencing of 16S rRNA genes and terminal restriction fragment length polymorphism (T-RFLP) of *mcrA* genes. The acetoclastic methanogen *Methanosaeta* was completely replaced by *Methanosarcina*. The propionic acid degrading genus *Syntrophobacter* was replaced by yet unknown propionic acid degraders. The butyric acid degrading genus *Syntrophomonas* and hydrogenotrophic *Methanomicrobiaceae* were hardly affected. We hypothesized that the ammonia sensitivity of the initially dominating taxa *Methanosaeta* and *Syntrophobacter* led to a stronger inhibition of the acetic and propionic acid degradation compared to butyric acid degradation and hydrogenotrophic methanogenesis, which were facilitated by the ammonia tolerant taxa *Syntrophomonas* and *Methanomicrobiaceae*. We implemented this hypothesis into a multi-taxa extension of ADM1, which was able to simulate the dynamics of both microbial community composition and VFA concentration in the experiment. It is thus plausible that the effect of ammonia on VFA degradation strongly depends on the ammonia sensitivity of the dominating taxa, for syntrophic propionate degraders as much as for acetoclastic methanogens.

## Introduction

Biogas production is an important renewable energy source and organic waste treatment technology (Plugge, 2017). For nitrogen-rich organic waste, the accumulation of ammonia can become a major problem. Ammonia inhibition has been held responsible for heavy economic losses and even reactor failures (Rajagopal et al., 2013). Suggested solutions to ammonia inhibition are based on the direct removal of ammonia from the reactor, the prevention of high ammonia concentrations by dilution or co-digestion with nitrogen-poor substrates (e.g., maize silage), or by adaptation of the microbial community (Krakat et al., 2017). Bioaugmentation (Fotidis et al., 2014) or support media (Poirier et al., 2017) have been suggested to speed up this adaptation.

Several, partly contradicting theories have been presented on ammonia inhibition in anaerobic digestion. A major controversy is whether acetoclastic or hydrogenotrophic methanogens are more strongly inhibited, with experimental evidence for both cases (Krakat et al., 2017). Furthermore, the shift toward more syntrophic acetate oxidation (SAO) instead of acetoclastic methanogenesis at elevated ammonia concentrations has received much attention (Schnürer and Nordberg, 2008; Werner et al., 2014; Luo et al., 2016). Commonly, free ammonia is thought responsible for ammonia inhibition because it can diffuse into the cells (Rajagopal et al., 2013), but also the ammonia ion is thought to cause inhibition (Astals et al., 2018). The concentration of free ammonia depends on total ammonia nitrogen (TAN) concentration, pH, and temperature. Several underlying and partly connected mechanisms of free ammonia inhibition after diffusion into a cell have been put forward and summarized by Krakat et al. (2017): proton imbalance, change of the intracellular pH, increase in maintenance energy requirement, and inhibition of specific enzymatic reactions. Interestingly, these mechanisms have been solely discussed as explanations for ammonia inhibition in the context of methanogenesis. However, they might also apply to other functional groups, for example proton-reducing bacteria degrading propionic or butyric acid.

Propionic acid and in fewer cases and less in strength also butyric acid accumulations have been observed repeatedly in the context of ammonia inhibition (Li et al., 2017b; Yirong et al., 2017; Peng et al., 2018; Yang et al., 2018). Nevertheless, the inhibition of syntrophic propionic and butyric acid oxidizing bacteria is often neglected in mechanistic descriptions of ammonia inhibition. For example, in the Anaerobic Digestion Model No. 1 (ADM1), only the inhibition of acetoclastic methanogens is included (Batstone et al., 2002). In a more recent adaptation of ADM1, ammonia inhibition was implemented for syntrophic acetic acid oxidizing bacteria (Wett et al., 2014) but still not for syntrophic propionic and butyric acid oxidizers. Quantitative descriptions of the inhibition of volatile fatty acid (VFA) degradation are difficult in complex systems because these acids are simultaneously produced and consumed. While there are several studies on ammonia inhibition using acetic acid as sole carbon source (Steinhaus et al., 2007; Hao et al., 2015; Westerholm et al., 2017), there is only one study on the impact of ammonia inhibition on an open, methanogenic culture fermenting propionic acid as sole carbon source (Li et al., 2017a). In their study, methanogenesis from propionic acid was strongly inhibited, but it could not be concluded if propionic acid oxidizing bacteria are directly inhibited by ammonia or indirectly inhibited by accumulation of hydrogen via an inhibition of the hydrogenotrophic methanogens as suggested earlier (Wiegant and Zeeman, 1986). There are no studies on ammonia inhibition using butyric acid as sole carbon source.

The goal of our study was to compare ammonia inhibition of acetic acid degradation with that of propionic and butyric acid degradation. Therefore, we used a synthetic mixture of acetic, propionic, and butyric acid as substrate amended with micronutrients in three continuous stirred tank reactors (CSTRs), and followed VFA degradation efficiencies and microbial community dynamics over time. Deliberate ammonia inhibition was induced by increasing ammonium bicarbonate concentration in the substrate in two reactors while the third reactor remained unchanged as a control. Furthermore, we added HCl to one of the reactors to reduce the pH and thus the share of free ammonia, which has been shown previously to successfully alleviate ammonia inhibition (Strik et al., 2006). Microbial community compositions were analyzed over the course of the experiment using 16S rRNA gene amplicon sequencing for bacteria and terminal restriction fragment length polymorphism (T-RFLP) profiling of *mcrA* genes for methanogenic archaea. At the end of the experiment, the functional resilience of the microbial communities was studied by deliberate disturbances in the form of pulse feedings. Finally, ADM1 was amended by second populations for acetic and propionic acid degradation, respectively, to model the effect of microbial community changes on the VFA concentration.

## Materials and methods

### Laboratory-scale CSTR experiments

**Figure 1:**
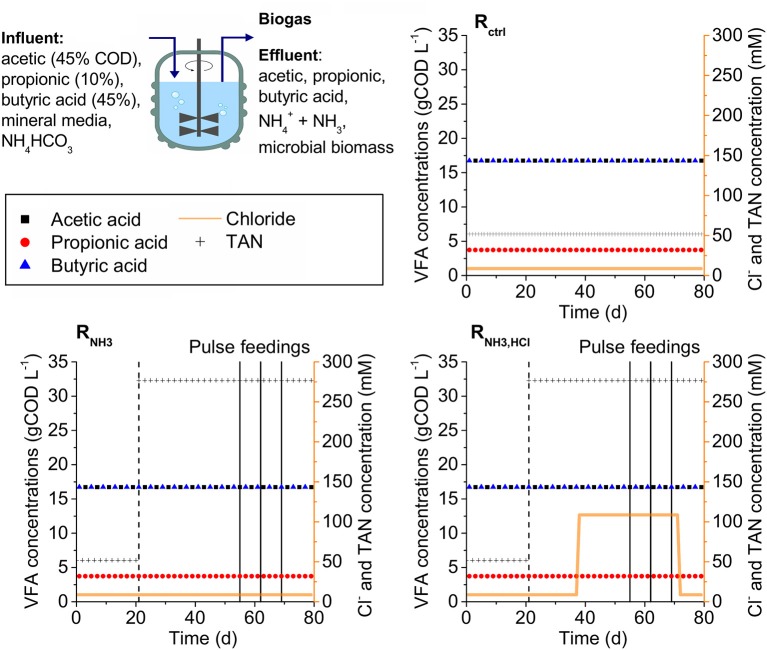
Experimental set-up. Three CSTRs (R_ctrl_, R_NH3_, R_NH3, HCl_) were fed with a synthetic medium containing VFAs, mineral medium and varying concentrations of total ammonia nitrogen (TAN) and HCl. Dashed vertical lines indicate the start of the increase in ammonia concentration in the influent of R_NH3_ and R_NH3, HCl_. Solid vertical lines indicate deliberate disturbances of R_NH3_ and R_NH3, HCl_.

Three CSTRs (R_ctrl_, R_NH3_, R_NH3, HCl_) with working volumes of 6 L were operated in parallel at 37°C at a hydraulic retention time (HRT) of 5.5 d. All CSTRs were fed continuously with a synthetic, liquid substrate containing a mixture of VFAs as the only carbon source (45% acetic, 10% propionic, and 45% butyric acid based on chemical oxygen demand, COD) with a total concentration of 37.2 gCOD L^−1^ in a mineral medium containing all necessary trace elements, macronutrients, and vitamins (see Supplementary Material A, Table S1). The CSTRs were inoculated from a lab-scale digester operated at a HRT of 5.5 d with the same synthetic substrate. The ammonia concentration in the substrate was 52 mM at the start for all CSTRs, and was increased to 277 mM for R_NH3_ and R_NH3, HCl_ on day 21 until the end of the experiment (see Figure 1). For R_NH3, HCl_, 100 mM HCl were added starting on day 38. On day 55, 62, and 69, R_NH3_ and R_NH3, HCl_ were fed not continuously but with the volume of one daily feeding at once as deliberate disturbances. On the day after each disturbance, continuous feeding was resumed as usual. On day 73, HCl addition to the medium of R_NH3HCl_ was ceased until the end of the experiment on day 79. Biogas composition (CH_4_, CO_2_, O_2_, H_2_, and H_2_S content), biogas production rate, pH, VFA concentrations, total solids content (TS), and volatile solids content (VS) were determined as described previously (Mulat et al., 2016). The TS and VS content measurements were modified in a way that a reactor sample was not added directly to a crucible, but only the pellet after centrifuging 100 mL reactor content (10,000 × g, 10 min, 10°C) to increase the amount of biomass per analysis and washing the pellet with phosphate buffered saline (PBS) solution (140 mM NaCl, 10 mM Na_2_HPO_4_ × 2 H_2_O, 2.7 mM KCl, 1.8 mM KH_2_PO_4_, pH 7.4) to reduce the potential error of VFAs and ammonia in the reactor sample.

The degradation efficiencies *E*_*i*_ (t) for each individual VFA *i* were calculated for each sampling date based on mass balances over 1 day considering the VFA input via the influent over the last day *S*_*i*_,_*in*_*/HRT*·Δ*t*, the VFA output via the effluent over the last day Δ*S*_*i, out*_ (t), the amount of VFAs in the reactor at the sampling date minus the amount at the previous day Δ*S*_*i*_(t) and, for acetic acid degradation only, the production of acetic acid from propionic and butyric acid oxidation over the last day Δ*S*_*i, p*_ (t). VFA mass degraded during 1 day is then given by *S*_*i,in*_/*HRT*.Δ*t* + Δ*S*_*i,p*_(*t*) − Δ*S*_*i,out*_(*t*) − Δ*S*_*i*_(*t*). Based on a constant reaction volume, the masses of each component can be calculated based on the respective concentrations in the influent and reactor digestate so that degradation efficiency can be expressed as:

(1a) $$E_{i}\left(t\right)=\frac{\frac{S_{i,in}}{HRT}\cdot \Delta t-\Delta S_{i,out}(t)+\Delta S_{i,p}(t)-\Delta S_{i}(t)}{\frac{S_{i,in}}{HRT}\cdot \Delta t+\Delta S_{i,p}(t)}$$

(1b) $$\Delta S_{i,out}(t)=\frac{S_{i}(t)+S_{i}(t-\Delta t)}{2\cdot HRT}\cdot \Delta t$$

(1c) $$\Delta S_{i}(t)=S_{i}(t)-S_{i}(t-\Delta t)$$

(1d) $$\begin{array}{l} \Delta S_{ac,p}(t)=\frac{S_{pro,in}}{HRT}\cdot \Delta t\cdot E_{pro}(t)\cdot f_{ac,pro}\\ \;+\frac{S_{bu,in}}{HRT}\cdot \Delta t\cdot E_{bu}(t)\cdot f_{ac,bu} \end{array}$$

(1e) $$\Delta S_{pro,p}(t)=\Delta S_{bu,p}(t)=0$$

with *f*_*ac,pro*_ and *f*_*ac,bu*_ being the stoichiometric conversion factors of propionate oxidation (1 mol acetate per mol propionate) and of butyrate oxidation (2 mol acetate per mol of butyrate), respectively. Between sampling dates, VFA concentrations were linearly interpolated. *E* values of 1 correspond to a complete degradation in steady state. *E* values can reach values higher than 1 when accumulated VFAs are degraded (Δ*S*_*i*_(t) < 0).

### Microbial community analyses

#### DNA and RNA extraction

Reactor samples were taken using 5 mL pipetting tips that were cut at the top to prevent the exclusion of aggregates of microorganisms and thereby a biased community analysis. Samples were centrifuged at −7°C, 15,000 × g for 2 min and the supernatant was discarded. The low centrifugation temperature and short duration did not lead to sample freezing and were chosen in order to prevent RNA degradation. Pellets were stored at −80°C prior to RNA extraction and −20°C prior to DNA extraction. NucleoSpin® Soil Kit (MACHEREY-NAGEL GmbH & Co. KG, Germany, buffer SL2, no enhancer) was used to extract DNA from the pellets. The quality and quantity of purified DNA were determined by agarose gel electrophoresis and by NanoDrop ND 1000 spectral photometer (Thermo Fisher Scientific, USA). DNA was stored at −20°C. The ZR Soil/Fecal RNA MicroPrepTM Kit (Zymo Research, USA) was used for RNA extraction. DNA was removed using the DNA-free™ DNA Removal Kit (InvitrogenTM, Thermo Fisher Scientific, USA). The quality and quantity of extracted RNA were determined by Qubit® Fluorometer 3.0 (Life technologies, USA, Oregon, Eugene) with the high sensitivity Qubit® RNA HS Assay Kit. RNA was converted to cDNA using the RevertAid H Minus First Strand cDNA Synthesis Kit (Thermo Scientific™, Thermo Fischer Scientific, USA). The resulting cDNA was stored at −20°C. The concentration of cDNA was quantified with a NanoDrop® ND-1000 spectral photometer (Thermo Fisher Scientific, USA).

#### Composition of the methanogenic community

T-RFLP was used to analyze the methanogenic community composition based on *mcrA* gene amplicons as described previously (Sträuber et al., 2016). The database of Bühligen et al. (2016) was used for the taxonomic assignment. PCR primers mlas (GGT GGT GTM GGD TTC ACM CAR TA) and mcrA-rev (CGT TCA TBG CGT AGT TVG GRT AGT) and PCR program were applied as described previously (Steinberg and Regan, 2008). *Bst*NI (New England Biolabs) was used as restriction enzyme.

#### Composition of the bacterial community

Amplicon sequencing of 16S rRNA genes was used to analyze the bacterial community compositions. PCR amplification using the primers 341f (CCT ACG GGN GGC WGC AG) and 785r (GAC TAC HVG GGT ATC TAA KCC) (Klindworth et al., 2013) and amplicon sequencing with the MiSeq platform (V3, 2 × 300 bp, Illumina) were performed by LGC Genomics GmbH (Berlin, Germany). De-multiplexing and removal of barcodes (allowing 1 mismatch), adapter and primer sequences (allowing 3 mismatches) were performed by LGC Genomics. Forward and reverse reads were merged using the BBMerge 34.48 software (http://bbmap.sourceforge.net/). Merged reads were processed using the QIIME 1.9.1 Virtual Box release (Caporaso et al., 2010). Quality filtering was applied removing low quality reads (quality threshold lower than 20) and allowing no ambiguous base calls. Chimeric sequences were removed and operational taxonomic unit (OTU) clustering was performed using the usearch tool (Edgar, 2010). Taxonomic assignment was performed using the latest MiDAS taxonomy 2.1 (McIlroy et al., 2015) and the RDP classifier 2.2 with a confidence threshold of 0.8 (Wang et al., 2007). The OTU tables were rarefied to 33,013 sequences per sample. The rarefied OTU tables were filtered for bacterial OTUs because the applied primers only partially amplify archaeal 16S rRNA genes and the resulting methanogenic community composition is therefore potentially biased (Klindworth et al., 2013). Raw de-multiplexed sequence data was deposited at EMBL European Nucleotide Archive (ENA) under accession number PRJEB27940 (http://www.ebi.ac.uk/ena/data/view/ PRJEB27940). Relative abundances of 16S rRNA genes were converted to relative genome abundances using the average 16S rRNA gene copy number per genome taken from the rrnDB database (version 5.2).

### ADM1 model extension

We modified the original ADM1 model structure (Batstone et al., 2002) in various aspects. Mass balances were closed by amending the ordinary differential equations for inorganic carbon and inorganic nitrogen. Biogas production rate was calculated considering the overpressure in the headspace. If not indicated otherwise, parameter values from the benchmark model by Rosen and Jeppsson (2006) were used.

The acetoclastic methanogens (X_ac_) were replaced by two competing populations: *Methanosarcina* (X_ac,1_, see Equation 2a) and *Methanosaeta* (X_ac,2_, see Equation 2b). Accordingly, the differential equation for acetic acid was adapted to Equation 2c. The original ADM1 inhibition functions (Batstone et al., 2002) were used with different parameter values for X_ac,1_ and X_ac,2_ concerning the ammonia inhibition function (Equation 2d, Table 1).

(2a) $$\begin{array}{l} \frac{dX_{ac,1}}{dt}=\frac{1}{HRT}\cdot \left(X_{in,ac,1}-X_{ac,1}\right)\\ \;+\left(Y_{ac,1}\cdot k_{m,ac,1}\cdot \frac{S_{ac}}{S_{ac}+K_{S,ac,1}}\cdot I_{ac,1}-k_{dec}\right)\cdot X_{ac,1} \end{array}$$

(2b) $$\begin{array}{l} \frac{dX_{ac,2}}{dt}=\frac{1}{HRT}\cdot \left(X_{in,ac,2}-X_{ac,2}\right)\\ \;+\left(Y_{ac,2}\cdot k_{m,ac,2}\cdot \frac{S_{ac}}{S_{ac}+K_{S,ac,2}}\cdot I_{ac,2}-k_{dec}\right)\cdot X_{ac,2} \end{array}$$

(2c) $$\begin{array}{l} \frac{dS_{ac}}{dt}=\frac{1}{HRT}\cdot (S_{ac,in}\\ \;-S_{ac})-k_{m,ac,1}\cdot \frac{S_{ac}}{S_{ac}+K_{S,ac,1}}\cdot I_{ac,1}\cdot X_{ac,1}\\ \;-k_{m,ac,2}\cdot \frac{S_{ac}}{S_{ac}+K_{S,ac,2}}\cdot I_{ac,2}\cdot X_{ac,2}+\sum_{j=5}^{11}v_{7,j}\cdot \rho_{j}, \end{array}$$

(2d) $$I_{nh3,\;i}=\frac{1}{1+\frac{S_{nh3}}{K_{I,nh3,i}}}$$

with *Y* being the biomass yield, *k*_*m*_ the maximum substrate uptake rate, *K*_*S*_ the half saturation constant, *k*_*dec*_ the decay rate, *I* the inhibition functions, *S* the substrate concentration, *HRT* the hydraulic retention time, *ac* the index for acetic acid, *ac1* the index for *Methanosarcina, ac2* the index for *Methanosaeta*, $\overset{11}{\underset{j=5}{\sum }}\upsilon_{7,j}\cdot \rho_{j}$ the acetic acid producing reactions (see Peterson Matrix, Supplementary Material B), *I*_*nh*3,*i*_ the inhibition ammonia inhibition function, *S*_*nh*3_ the ammonia concentration and *K*_*I,nh*3,*i*_ the empirical ammonia inhibition parameter with different values for *Methanosarcina* (*i* = *ac1*) and *Methanosaeta* (*i* = *ac2*).

**Table 1:** Model parameter values for acetic and propionic acid degrading populations.

**Microbial biomass population**	**Yield Y (gCOD_**X**_ gCOD${}_{S}^{-1}$)**	**Maximum substrate uptake rate k_**m**_ (gCOD_**S**_ gCOD${}_{X}^{-1}$ d^**−1**^)**	**Half saturation constant K_**S**_ (gCOD_**S**_ L^**−1**^)**	**Ammonia inhibition K_**I, nh3**_ (M)**	**Reference**
X_ac,1_	0.05	8	**0.34**	**0.3387**	This study
X_ac,2_	0.05	8	0.15	**0.0052**	This study
X_ac_	0.05	8	0.15	0.0018^1^	Rosen and Jeppsson, 2006
X_pro,1_	0.04	13	**0.34**	**0.4887**	This study
X_pro,2_	0.04	13	0.10	**0.0036**	This study
X_pro_	0.04	13	0.10	N/A	Rosen and Jeppsson, 2006

1 *K_I, nh3, ac_ was manually adjusted to 0.008 for simulating the original ADM1 structure (Figure 6A), because the value of 0.0018 leads to process failure*.

*N/A, Not applicable*.

*Changes to the default values by Rosen and Jeppsson (2006) are highlighted in bold letters*.

The propionic acid degraders (X_pro_) were replaced by two competing populations: an unknown taxon (X_pro,1_) and *Syntrophobacter* (X_pro,2_) following the same approach as described above for the acetoclastic methanogens (Supplementary Material A, Section Implementation of two competing propionic acid oxidizers in ADM1). Ammonia inhibition was introduced for both populations following the same inhibition equation that is used for acetic acid degraders in the original ADM1 structure with different parameter values for X_pro,1_ and X_pro,2_ (see Table 1). A detailed description of the extended model structure is included in the Supplementary Material (Peterson Matrix in Supplementary Material B). All differential and algebraic equations were implemented and solved in the software environment Matlab R2014b (Mathworks).

Cation and anion concentrations in the influent were calculated based on the composition of our synthetic media to 78.3 and 19.8 mM, respectively. Inorganic carbon concentrations were equal to inorganic nitrogen concentrations in the influent with 52 mM before and 277 mM after day 21 based on the ammonium bicarbonate concentration in the synthetic media. Initial concentrations for all state variables (except X_ac,1_ and X_pro,1_) were approximated based on simulation results for steady state concentrations for the inorganic nitrogen concentration of 52 mM in the influent (ADM1 Simulation in Supplementary Material B).

Only parameter values for acetic acid and propionic acid degraders were changed compared to the benchmark model parameter values by Rosen and Jeppsson (2006). The initial concentrations and half-saturation constants of the ammonia tolerant populations (X_ac1_(*t* = 0), X_pro1_(*t* = 0), K_S,ac1_, K_S,pro1_) and ammonia inhibition constants of all acetic acid and propionic acid degraders (K_I,nh3,ac1_, K_I,nh3,ac2_, K_I,nh3,pro1_, K_I,nh3,pro2_) were estimated using the solver fmincon (algorithm: interior point) in Matlab R2014b (Mathworks) minimizing the sum of squared error between experimental and simulation results, first for propionic acid concentration and second for acetic acid concentration.

## Results

### CSTR performance

**Figure 2:**
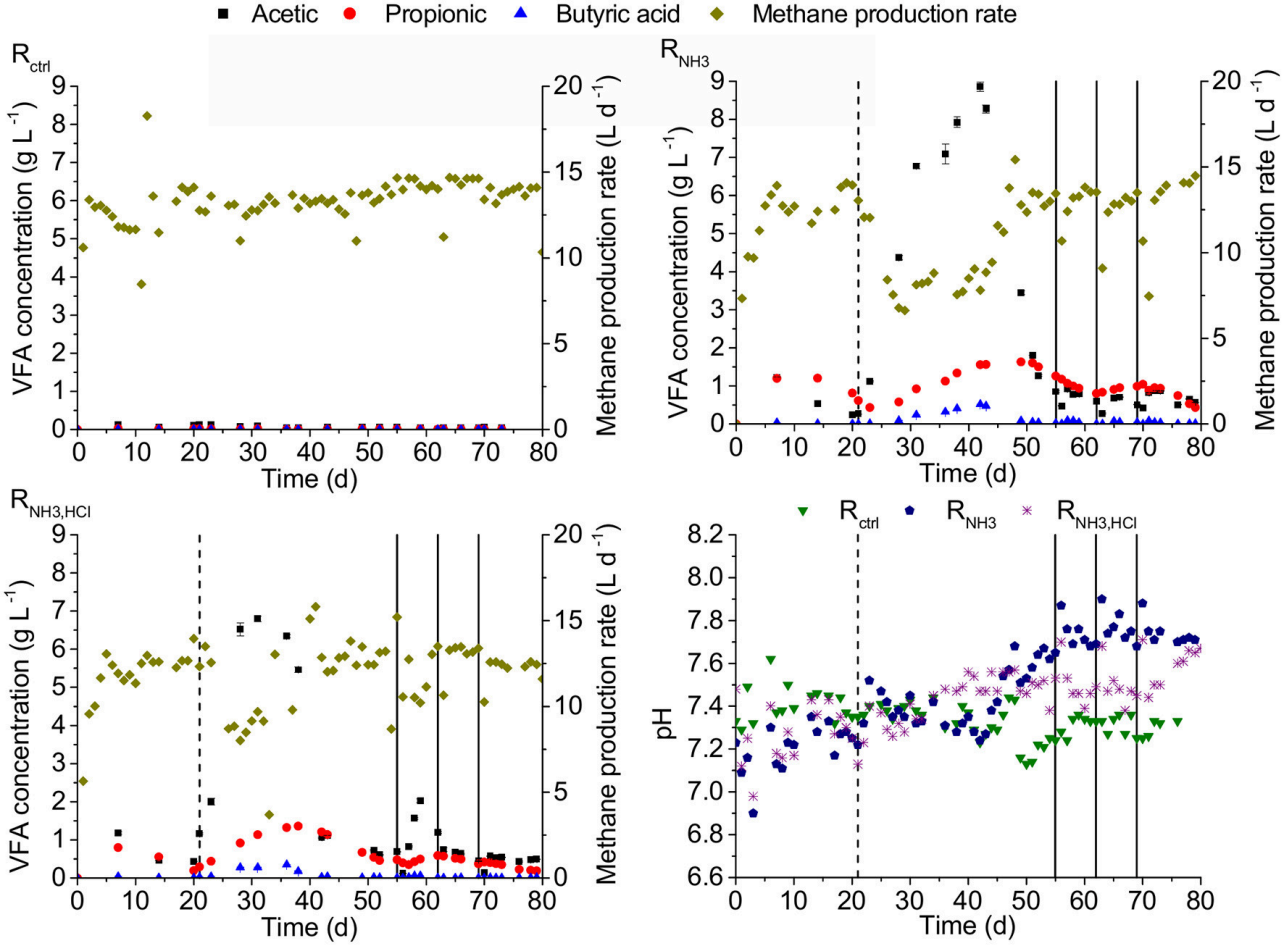
VFA concentration, methane production rate and pH for the three CSTRs R_ctrl_, R_NH3_, and R_NH3, HCl_. Dashed vertical lines indicate the start of the increase in ammonia concentration for R_NH3_ and R_NH3, HCl_. Solid vertical lines indicate deliberate disturbances of R_NH3_ and R_NH3, HCl_. Error bars represent one standard error of the mean.

On day 21, after 3 HRTs under low ammonia concentrations of 52 mM, all three CSTRs (see Figure 2) had total VFA concentrations of maximal 1.5 g L^−1^ and pH values of maximal 7.35. Increasing the ammonia concentration in the influent to 277 mM in R_NH3_ and R_NH3, HCl_ starting on day 21 was followed by an accumulation of VFAs and decline in methane production rates. The strongest accumulation was observed for acetic acid with up to 8.86 g L^−1^ (R_NH3_ on day 42). Propionic acid accumulated up to 1.63 g L^−1^ (R_NH3_ on day 49) and butyric acid showed the smallest accumulation with up to 0.52 g L^−1^ (R_NH3_ on day 42). On day 55, acetic, propionic, and butyric acid concentrations had declined to 0.85, 1.26, and 0.02 g L^−1^ for R_NH3_ as well as 0.69, 0.48, and 0.01 g L^−1^ for R_NH3, HCl_, respectively. This decline was accompanied by increases in pH values to 7.7 and 7.53 for R_NH3_ and R_NH3, HCl_, respectively. Starting on day 73, after HCl was omitted from the medium of R_NH3, HCl_, the pH value in R_NH3, HCl_ rose to 7.7 toward the end of the experiment, equal to R_NH3_, while total VFA concentrations remained at about 1 gCOD L^−1^. Hydrogen partial pressures in the biogas were hardly affected by the increasing ammonia concentration and remained below 17 ppm in all CSTRs between day 21 and 55.

On days 55, 62, and 69, R_NH3_ and R_NH3, HCl_ were given the amount of one daily feeding within 20 min instead of continuous feeding. Both reactors recovered within 3 days to pre-disturbance acetic and butyric acid degradation efficiencies except for R_NH3, HCl_ on day 55 when an acetic acid accumulation was observed followed by a recovery over about one HRT. In both reactors, propionic acid accumulation was observed after a disturbance followed by a recovery over about two HRTs.

**Figure 3:**
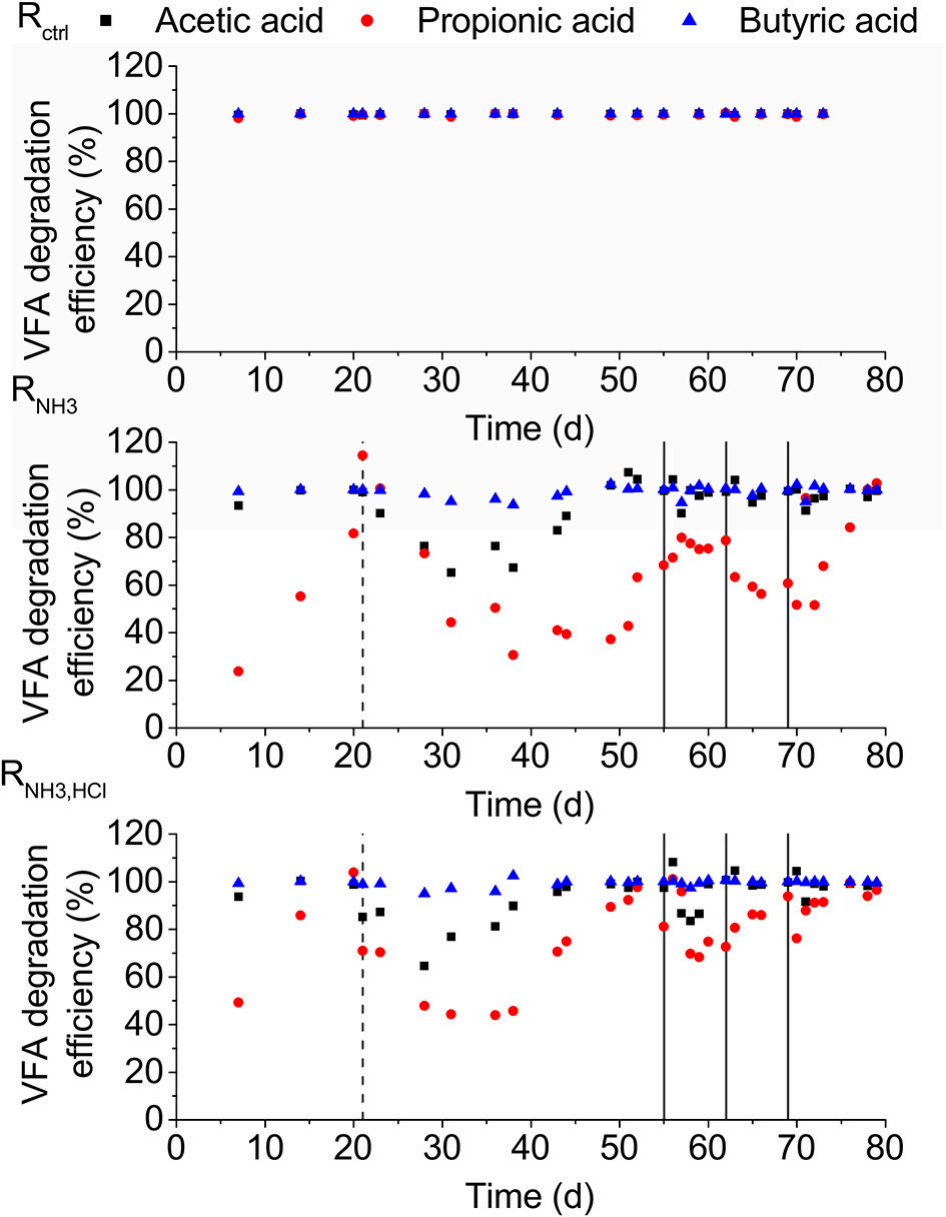
VFA degradation efficiencies for the three reactors. Dashed lines mark the beginning of the ammonia inhibition and solid lines the deliberate disturbances.

During the start of the experiment, the degradation efficiencies of acetic and propionic acid in R_NH3_ and R_NH3, HCl_ showed declines but recovered again until day 21. On day 21, the degradation efficiencies of all VFAs were higher than 86% in all reactors (Figure 3). After the increase in ammonia concentration on day 21, the lowest degradation efficiencies were 31% for propionic and 65% for acetic acid. Butyric acid degradation was almost not affected showing efficiencies of higher than 94% throughout the experiment.

After increasing the ammonia concentration, the formation of aggregates was observed, which reached a size of up to one mm in diameter toward the end of the experiment (see Supplementary Material A, Figure S1).

### Methanogenic community dynamics

**Figure 4:**
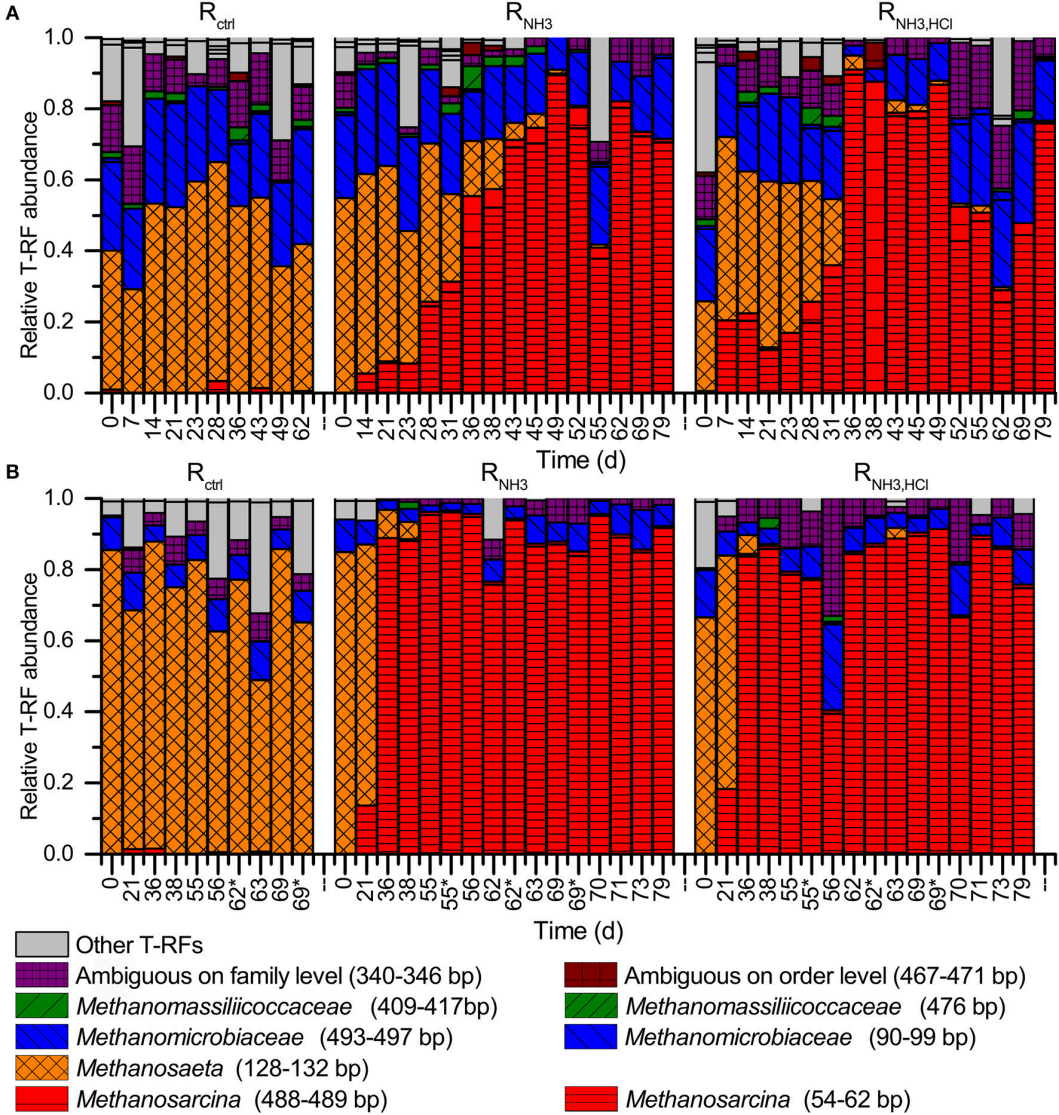
Methanogenic community analyses by T-RFLP profiling using *Bst*NI as restriction enzyme **(A)** on DNA basis and **(B)** on mRNA (cDNA) basis. Note the different time scales in **(A)** and **(B)**. Days labeled with an asterisk indicate samples taken 5 h after the deliberate disturbances. T-RFs with a length of 340–346 bp can be *Methanocorpusculaceae, Methanomicrobiaceae, Methanoregulaceae*, and/or *Methanospirillacea*. T-RFs with a length of 467–471 bp can be *Methanobacteriales, Methanococcales*, and/or *Methanomassiliicoccales*.

On day 21, all CSTRs showed a similar methanogenic community composition on DNA level, which was dominated by the acetoclastic *Methanosaeta* (47–56%) and the hydrogenotrophic *Methanomicrobiaceae* (25–30%). *Methanosarcina*, which can perform both acetoclastic and hydrogenotrophic methanogenesis, reached a relative abundance of up to 13% (Figure 4).

After increasing the ammonia concentration in the influent to 277 mM in R_NH3_ and R_NH3, HCl_ starting on day 21, a strong increase in the relative abundance of *Methanosarcina* with a simultaneous decrease of *Methanosaeta* was detected for R_NH3_ and R_NH3, HCl_, while *Methanosaeta* continued to dominate over *Methanosarcina* in R_ctrl_ (Figure 4A). *Methanomicrobiaceae* remained in all reactors throughout the experiments and reached a relative abundance of about 20% in R_NH3_ and R_NH3, HCl_ at the end of the experiment.

On cDNA level, the shift from *Methanosaeta* to *Methanosarcina* as the dominant methanogen was even more pronounced (Figure 4B). For R_NH3_, *Methanosarcina* reached a relative abundance of 90% already on day 36 on cDNA level compared to day 49 on DNA level. Relative T-RF abundances were in general higher on cDNA level compared to DNA level for *Methanosarcina* in R_NH3_ and R_NH3, HCl_ and for *Methanosaeta* in R_ctrl_. No major impacts of the deliberate disturbances on the relative T-RF abundances of *mcrA* transcripts were observed.

### Bacterial community composition

On day 21, 96 to 110 OTUs were detected in the three CSTRs. All CSTRs were dominated by the OTUs *Syntrophomonas* (8–27% of bacterial reads), *Syntrophobacter* (13–16%), *Thermovirga* (15–22%), and Blvii28 wastewater-sludge group (6–38%).

**Figure 5:**
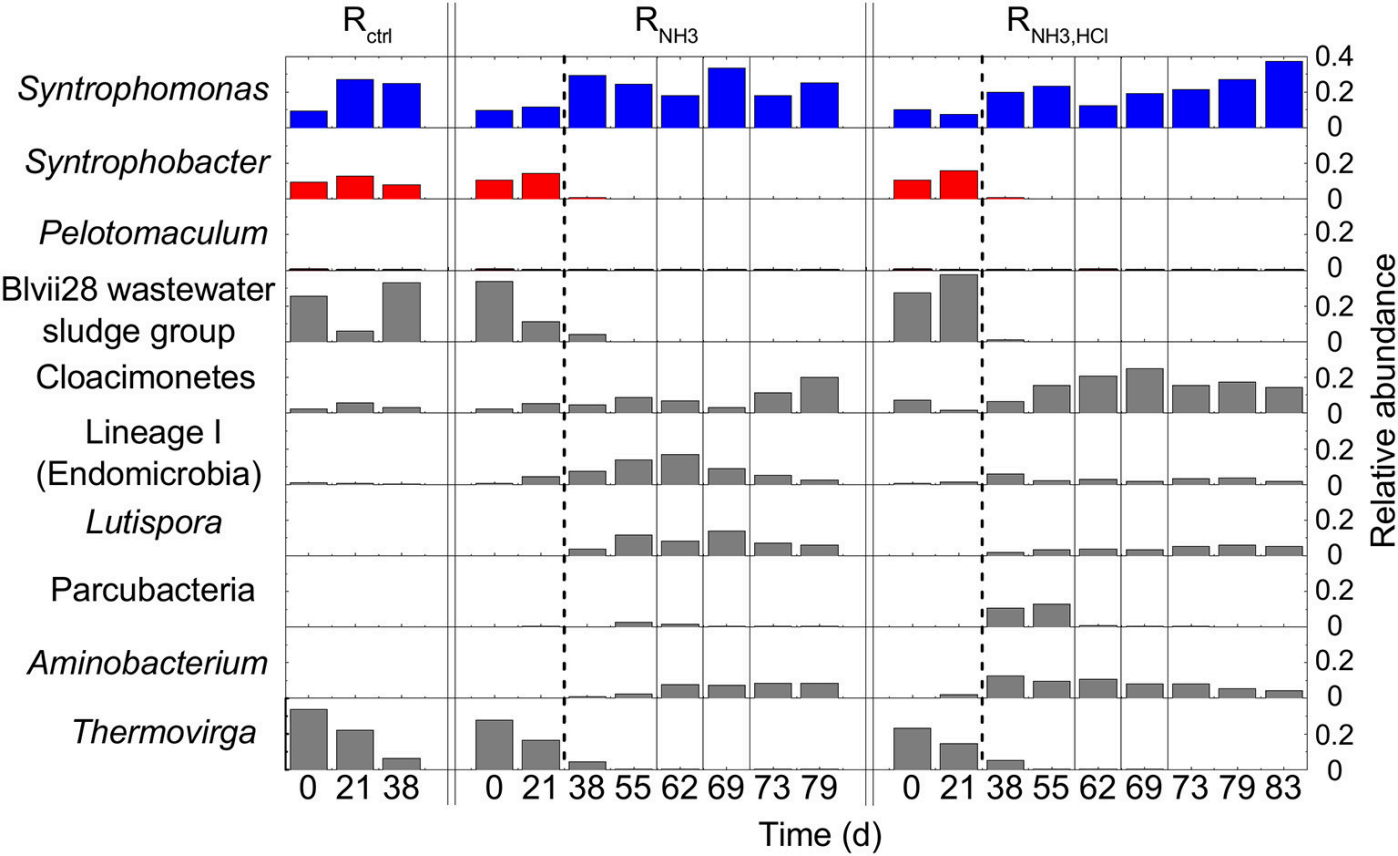
Bacterial community composition based on 16S rRNA gene amplicon sequencing. Dashed lines mark the beginning of the ammonia inhibition and single solid lines the deliberate disturbances. Only *Syntrophomonas, Syntrophobacter, Pelotomaculum*, and the seven most abundant OTUs (>12% relative abundance to total bacteria) are shown. Known butyric acid oxidizers are shown in blue, known propionic acid oxidizers in red, and other OTUs in gray.

After the increase in ammonia concentration, the community composition of R_NH3_ and R_NH3, HCl_ showed strong changes. *Syntrophobacter, Thermovirga* and Blvii28 wastewater-sludge group decreased in relative abundance to < 0.1% of all bacterial reads in both R_NH3_ and R_NH3, HCl_ toward the end of the experiment (Figure 5). *Aminobacterium* and *Lutispora* increased in both reactors from below 1.9% to 4.4–12.6% and from below 0.1% to 1.8–13.9% of all bacterial reads, respectively. The OTU Lineage 1 (Endomicrobia) strongly increased in relative abundance shortly after the ammonia inhibition in R_NH3_ and decreased again toward the end of the experiment. A similar behavior was observed for Parcubacteria in R_NH3, HCl_. *Syntrophomonas* remained one of the most abundant bacterial genera throughout the experiment in all CSTRs.

Our primer pair also led to the amplification of 16S rRNA genes from methanogens despite the lower coverage of archaea compared to bacteria (Klindworth et al., 2013). The ratio of bacterial to total reads varied only slightly throughout the experiment. On day 21, bacterial reads made up 18% and 16% of total reads for R_NH3_ and R_NH3, HCl_, respectively, compared to 13 and 17%, respectively, on day 79.

### ADM1 simulation

**Figure 6:**
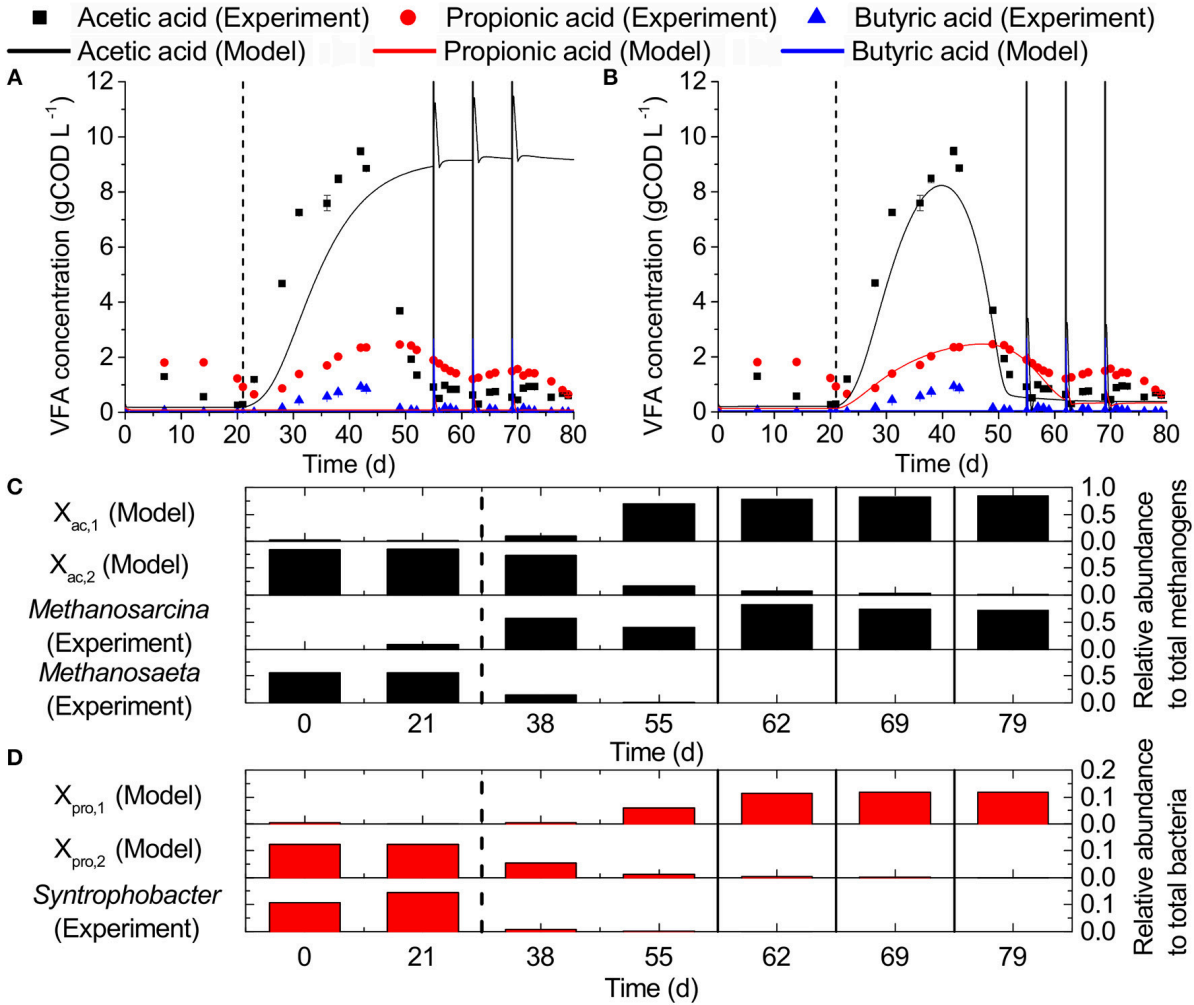
Experimental observations vs. ADM1 simulations: VFA concentrations for **(A)** the original ADM1 structure and **(B)** our model including second competing populations for acetic and propionic acid degradation, respectively, as well as ammonia inhibition of propionic acid degradation. **(C,D)** Relative abundances of acetic acid degraders and propionic acid degraders, respectively. Dashed vertical lines mark the beginning of the ammonia inhibition and solid vertical lines the deliberate disturbances.

Simulations using the default ADM1 implementation of ammonia inhibition did not resemble our observed VFA concentrations (Figure 6A, *r*${}_{\text{acetic}}^{2}$_acid_ = 0.05, *r*${}_{\text{propionic}}^{2}$_acid_ = 0.07). Only acetic acid accumulation was predicted, but not the acclimatization toward the end of the experiment. After implementing our model modifications, i.e., second populations for acetic and propionic acid degradation, respectively, the simulations represented the observed VFA concentrations better (Figure 6B, *r*${}_{\text{acetic}}^{2}$_acid_ = 0.98, *r*${}_{\text{propionic}}^{2}$_acid_ = 0.56). After the increase of ammonia concentration in the influent on day 21, the ammonia sensitive populations in the simulations (X_ac2_, X_pro2_) became strongly inhibited and were washed out, while accumulations of 8.2 and 2.5 gCOD L^−1^ occurred for acetic and propionic acid, respectively. At the same time, ammonia tolerant populations (X_ac1_, X_pro1_) increased in relative abundance (Figures 6C,D). On day 55, X_ac1_ reached a relative abundance to total methanogens of 0.72 and acetic acid accumulation was reduced to 0.5 gCOD L^−1^. On day 62, X_pro1_ reached a relative abundance to total bacteria of 0.12 and propionic acid accumulation was reduced to 0.4 gCOD L^−1^.

## Discussion

Apart from a lower pH, the addition of HCl to the influent of R_NH3, HCl_ starting on day 36 led only to minor differences in VFA concentrations, acclimatization time and microbial community composition compared to R_NH3_. Apparently, at the time of HCl addition, both reactors were already successfully acclimatizing to ammonia inhibition. Furthermore, both reactors were overall little affected by the disturbances. Therefore, both reactors are discussed in the following with the focus on their common response to ammonia inhibition. The VFA accumulations during the start-up of R_NH3_ and R_NH3, HCl_ seemed to have no decisive effects on the overall experiments since both reactors recovered before the ammonia inhibition was induced.

### Strong ammonia inhibition of both acetic and propionic acid degradation

The inhibition of VFAs other than acetic acid are neglected in ADM1 (Batstone et al., 2002). However, looking at VFA degradation efficiencies (Figure 3), it became clear that propionic acid degradation was even more inhibited than acetic acid degradation. We also observed a stronger or similarly strong inhibition of propionic acid degradation compared to acetic acid degradation in several additional experiments we conducted (see Supplementary Material A). This suggests that ammonia inhibition of propionic acid degradation should receive more attention and be included in anaerobic digestion models.

Calculating VFA degradation efficiencies is only possible in synthetic systems like ours and not for more complex substrates such as manure, because the amounts of individual VFAs produced in acidogenesis from complex substrates are hard to quantify and thus, the VFA degradation efficiencies cannot be calculated. Therefore, the impact of ammonia inhibition on propionic acid degradation might have appeared weaker than it actually was in many studies because lower propionic acid concentrations than acetic acid concentrations were reached.

**Figure 7:**
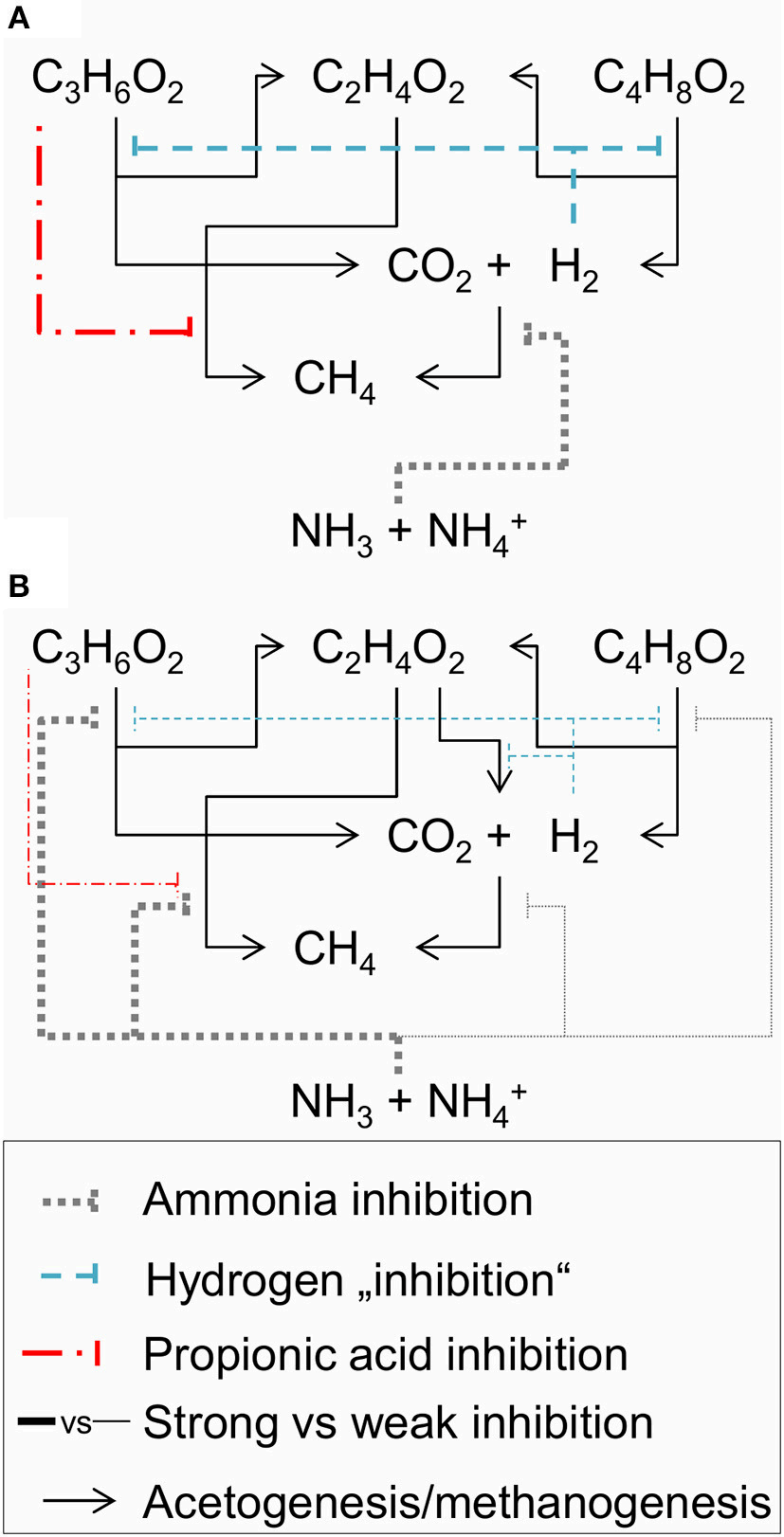
Inhibition scheme of acetogenesis and methanogenesis following **(A)** Wiegant and Zeeman (1986) and **(B)** our working hypothesis. Hydrogen “inhibition” means that increased hydrogen partial pressures increase the Gibbs free energy change of catabolism of propionic and butyric acid oxidation. C_2_H_4_O_2_, C_3_H_6_O_2_, C_4_H_8_O_2_, CO_2_, H_2_, CH_4_, NH_3_, and NH${}_{4}^{+}$ are the molecular formulas of acetic, propionic, and butyric acid, as well as carbon dioxide, hydrogen, methane, free ammonia and ammonium cation, respectively.

Concerning the mechanism of ammonia inhibition of propionic acid degradation, an indirect inhibition mechanism was suggested by Wiegant and Zeeman (1986) who argued that a strong ammonia inhibition of hydrogenotrophic methanogenesis can lead to accumulation of hydrogen, which inhibits propionic acid degradation by increasing the Gibbs energy change of catabolism to near or above zero (Figure 7A). However, this indirect route seemed not to play a major role in our experiment since we did not observe any hydrogen accumulation. Furthermore, butyric acid degradation was almost not inhibited in our experiment, which would have been the case if hydrogen accumulated. Therefore, we assumed direct ammonia inhibition of propionic acid degradation as the working hypothesis for our study. Based on our observations, acetic and propionic acid degradation were strongly inhibited in our experiment while butyric acid degradation and hydrogen conversion were hardly affected (Figure 7B).

### Relationship of VFA degradation rates and microbial community composition

After the increase in ammonia concentration started, it took about 5 HRTs until VFA concentrations reached values similar to the start of the experiment in R_NH3_ and R_NH3, HCl_. These increases and decreases in VFA concentrations were accompanied by strong shifts in the microbial community.

The propionic acid oxidizing *Syntrophobacter* was washed out of the reactors after ammonia inhibition started. Also in several other experiments we conducted at elevated ammonia concentrations, *Syntrophobacter* was washed out (see Supplementary Materials A,C). The absence of *Syntrophobacter* at high ammonia concentrations was also observed in a mesophilic reactor treating household waste at TAN concentrations of 386–414 mM (Westerholm et al., 2015), supporting our observation that *Syntrophobacter* is an ammonia sensitive taxon. The only other known syntrophic propionic acid degrading genus in our experiments was *Pelotomaculum*, which was not competitive with a relative abundance of about 0.1% independent of the ammonia concentration.

Since propionic acid oxidation continued after these two taxa reached negligible relative abundances, one or several yet unknown ammonia tolerant propionic acid oxidizing taxa must be among the detected bacteria. The OTUs that increased in relative abundance after the ammonia inhibition such as *Aminobacterium* and *Lutispora* are possible candidates; however, they could grow on butyric acid or decaying biomass as well. Other studies support the ammonia tolerance of these two taxa. *Lutispora* was found to correlate with the recovery of biogas production in an ammonia inhibited biogas reactor digesting wastewater treatment plant sludge (Chen et al., 2018). *Aminobacterium* increased in abundance after an increase in ammonia concentration in an anaerobic digester treating chicken manure and feathers with wood shavings (Belostotskiy et al., 2015).

The butyric acid degrading *Syntrophomonas* tolerated the increase in ammonia concentration in our experiment. A tolerance to TAN of up to 6.5 g L^−1^ was observed earlier (Lee et al., 2018). Poirier et al. (2017) even observed relative abundances to total bacteria of 22% for ammonia concentrations as high as 25 g L^−1^, supporting our observation that *Syntrophomonas* is ammonia tolerant.

The bacterial taxa *Thermovirga* and Blvii28 wastewater sludge group became abundant with up to 30% relative abundance to total bacteria each during the first 21 days. It is unlikely that they were mainly propionic acid degraders in our reactors because of their high abundance compared to the low share of propionic acid in the feed and it is unlikely that they were mainly acetic acid degraders because at the low free ammonia concentrations during the first 21 days in all reactors (<0.028 g L^−1^), acetoclastic methanogenesis commonly dominates over SAO (Luo et al., 2016). Therefore, they were most likely syntrophic butyric acid degraders. Since both taxa were washed out after the increase in ammonia concentration in our experiment as well as in an experiment by Lee et al. (2018), they likely are ammonia sensitive.

Concerning acetoclastic methanogens, *Methanosaeta* dominated all CSTRs at the start of the experiments when ammonia and acetic acid concentrations were low. Its advantage over *Methanosarcina* at low acetic acid concentrations can be explained by its higher substrate affinity and minimum concentration threshold for acetic acid (Jetten et al., 1992). After the increase in ammonia concentration, *Methanosaeta* was washed out during the ammonia inhibition and replaced by *Methanosarcina*. The sensitivity of *Methanosaeta* to ammonia has been shown in a pure culture study (Steinhaus et al., 2007). *Methanosarcina* is known to be more tolerant against ammonia inhibition than *Methanosaeta* (De Vrieze et al., 2012). This tolerance has been connected with their ability to form aggregates (De Vrieze et al., 2012). Aggregates with diameters up to 1 mm were also observed in our experiment. After *Methanosarcina* completely dominated the acetoclastic methanogens, acetic acid degradation efficiencies recovered up to almost 100% toward the end of the experiment, showing that the applied ammonia concentrations were not inhibitory for *Methanosarcina*.

The hydrogenotrophic methanogens of the family *Methanomicrobiaceae* were also tolerant to the increase in ammonia concentration and remained abundant until the end of the experiment. We observed this tolerance in several other experiments on ammonia inhibition that we conducted (see Supplementary Material A). *Methanomicrobiaceae* have been found previously to be tolerant to TAN concentrations of about 3 g L^−1^ in pure culture (Schnürer et al., 1999; Nettmann et al., 2010; Wang et al., 2015) and mixed culture studies (Angenent et al., 2002; Westerholm et al., 2011, 2012).

Theoretically, it is possible that all acetic acid was converted via SAO and hydrogenotrophic methanogenesis after day 21. In that case, *Methanosarcina* would be hydrogenotrophic instead of acetoclastic as assumed above. However, this appears to be unlikely. First, a switch to SAO should have led to an increase in relative abundance of bacteria compared to methanogens that we did not observe. Second, genera known for SAO were observed only in negligible abundances in our reactors. Five species capable of SAO have been cultured belonging to the genera *Pseudothermotoga, Thermacetogenium, Clostridium, Syntrophaceticus*, and *Tepidanaerobacter* (Müller et al., 2016). From them, only *Clostridium* and *Syntrophaceticus* were detected with maximum relative abundances of 0.2% to total bacteria. Third, anaerobic digesters dominated by SAO have been observed to be dominated by *Methanoculleus* or *Methanobrevibacter* instead of *Methanosarcina* (Luo et al., 2016). Finding acetoclastic *Methanosarcina* dominant in ammonia inhibited biogas plants is not unusual. Luo et al. (2016) observed the dominance of acetoclastic methanogenesis in industrial biogas plants with up to 0.46 g L^−1^ of free ammonia which is almost double the maximum free ammonia concentration of 0.25 g L^−1^ that we observed. Schnürer and Nordberg (2008) observed a dominance of acetoclastic methanogenesis over SAO at free ammonia nitrogen concentrations of up to about 0.3 g L^−1^ in lab-scale digesters. Therefore, we assumed in our ADM1 simulations that *Methanosarcina* replaces *Methanosaeta* as an acetoclastic methanogen. Still, it cannot be excluded that *Methanosarcina* additionally took up hydrogen and that some SAO occurred in our reactors.

### Implementation of taxon-specific ammonia inhibition in ADM1

The relationship between microbial community dynamics and ammonia as well as VFA concentrations could be successfully reproduced with our extended ADM1 model. However, major changes to the original ADM1 structure were necessary. First of all, inhibition of propionic acid degradation was not implemented in the original ADM1 structure. However, we could show in our experiment that propionic acid degradation was even stronger inhibited than acetic acid degradation, and therefore, the addition of an ammonia inhibition term for propionic acid degradation was necessary. Secondly, simulations based on the original ADM1 structure did not (and in principle cannot) lead to a process recovery after the increase in VFA concentrations following ammonia inhibition because only one taxon of each functional group is part of the original structure. For example, only one taxon is capable of acetoclastic methanogenesis. As a consequence, our simulations using the original ADM1 structure could only lead to no VFA accumulation, to a constant elevated VFA concentration, or process breakdown. However, our experimental data clearly showed an increase in VFA concentration due to ammonia inhibition, followed by a decrease in VFA concentration toward the end of the experiment after the microbial community adapted to the new conditions. Therefore, the addition of at least a second taxon of acetic and propionic acid degraders was necessary in ADM1 to reflect the reactor performance dynamics as a consequence of the microbial community dynamics.

When fitting the simulations to the experimental results, we only changed the ammonia inhibition constants of X_ac,1_, X_ac,2_, X_pro,1_, and X_pro2_, and the initial concentration and half saturation constants of X_ac,1_ and X_pro,1_ compared to the benchmark model parameter values by Rosen and Jeppsson (2006). The fitting resulted in higher substrate affinities (lower half saturation constants) for the ammonia sensitive populations (X_ac,2_ and X_pro,2_) compared to the ammonia tolerant populations (X_ac,1_ and X_pro,1_). This reflects the higher substrate affinities previously observed for *Methanosaeta* compared to *Methanosarcina* (Jetten et al., 1992; Straub et al., 2006). However, the advantage of *Syntrophobacter* over its competitors is not known. Other advantages than substrate affinity are possible, such as higher maximum substrate uptake rates, higher specific growth yields or a combination thereof. A high sensitivity for the parameters K_I,nh3,ac,2_, K_S,ac,1_, K_I,nh3,pro,2_, K_S,ac,2_ and the initial concentrations of X_ac,1_ and X_pro,1_ was found (see Sensitivity Analysis in Supplementary Material A). By contrast, increasing the values of the ammonia inhibition constants of the ammonia resistant taxa (K_I,nh3,ac,1_ and K_I,nh3,pro,1_), i.e., decreasing the ammonia sensitivity of these populations, did not change the simulation results. This indicates that higher ammonia concentrations are necessary to unambiguously determine these parameter values (see Supplementary Material A, Figure S6). Hydrogenotrophic methanogenesis and butyric acid oxidation were not changed in ADM1 because their performance was hardly impacted by the increase in ammonia concentration in our experiment.

While our model helps to illustrate the relationship between microbial community dynamics and VFA accumulation, extending it for the use as predictive model remains a challenge, in particular regarding parameter identification. In communities with competing taxa, the quantification of taxon-specific substrate uptake rates is a major obstacle. Fitting these rates based on gross consumption likely leads to non-unique solutions and a loss of generality and predictive power. A predictive model would require the physiological characterization of all relevant taxa in pure cultures or defined co-cultures on defined media. Isolating prokaryotes was not always successful in the past (Pelletier et al., 2008). However, the advent of metaomics techniques gives hope that suitable cultivation conditions can be inferred more easily in the future (Overmann et al., 2017). Success in cultivation would be rewarded with high benefits: A predictive multi-taxa ADM1 model could be an essential resource in gaining deterministic control over the microbial community composition, for example by changing process parameters and/or bioaugmentation, in order to increase the productivity of biogas plants suffering from ammonia and other inhibitions.

In conclusion, ammonia inhibition is a major challenge for the biogas process, resulting in economic losses and even process failures. Both our experimental and simulation results showed the importance of ammonia-sensitive taxa, such as *Methanosaeta* and *Syntrophobacter*, and ammonia-tolerant taxa, such as *Methanomicrobiaceae* and *Syntrophomonas*, for understanding the reactor performance as a result of microbial community dynamics of anaerobic digesters impacted by high ammonia concentrations.

## Data availability statement

The datasets generated for this study can be found in the Supplementary Material B and the EMBL European Nucleotide Archive (ENA) under accession number PRJEB27940 (http://www.ebi.ac.uk/ena/data/view/ PRJEB27940).

## Author contributions

FB, HS, DP, SK, and FC designed the experiments. FB performed the experiments and simulations and analyzed the reactor and T-RFLP data. SW and FB developed the ADM1 model extension. DP performed the 16S rRNA gene sequencing data analysis. HH contributed to the discussion of the results. All authors contributed to the preparation of the manuscript. All authors read and approved the manuscript.

### Conflict of interest statement

The authors declare that the research was conducted in the absence of any commercial or financial relationships that could be construed as a potential conflict of interest.
