# Supplementary material for: Ammonia Inhibition of Anaerobic Volatile Fatty Acid Degrading Microbial Communities
Source: Front Microbiol. 2018 Nov 30;9:2921. doi: 10.3389/fmicb.2018.02921 (PMC6284035; doi:10.3389/fmicb.2018.02921)
Supplement: Supplementary file 1 [file Data_Sheet_1.zip › Supplementary Materials C.html]

Javascript must be enabled to view this page.

magnitude

Exp1\_Rctrl\_t=0
Exp1\_RNH3\_t=0
Exp1\_RNH3,HCl\_t=0
Exp1\_Rctrl\_t=21
Exp1\_RNH3\_t=21
Exp1\_RNH3,HCl\_t=21
Exp1\_Rctrl\_t=38
Exp1\_RNH3\_t=38
Exp1\_RNH3,HCl\_t=38
Exp1\_RNH3\_t=55
Exp1\_RNH3,HCl\_t=55
Exp1\_RNH3\_t=62
Exp1\_RNH3,HCl\_t=62
Exp1\_RNH3\_t=69
Exp1\_RNH3,HCl\_t=69
Exp1\_RNH3\_t=73
Exp1\_RNH3,HCl\_t=73
Exp1\_RNH3\_t=79
Exp1\_RNH3,HCl\_t=79
Exp1\_RNH3,HCl\_t=83
Exp2\_t=0
Exp2\_t=46
Exp2\_t=103
Exp3\_Rconti\_t=0
Exp3\_Rconti\_t=58
Exp3\_Rconti\_t=100
Exp3\_Rconti\_t=108
Exp3\_Rconti\_t=154
Exp3\_Rdisco\_t=0
Exp3\_Rdisco\_t=58
Exp3\_Rdisco\_t=108
Exp4\_t=46

 1
 1
 1
 1
 1
 1
 1
 1
 1
 1
 1
 1
 1
 1
 1
 .999999999999999
 1
 1
 1
 1
 1
 1
 1
 1
 1
 1
 1
 1
 1
 1
 1
 1

 1
 1
 1
 1
 1
 1
 1
 1
 1
 1
 1
 1
 1
 1
 1
 .999999999999999
 1
 1
 1
 1
 1
 1
 1
 1
 1
 1
 1
 1
 1
 1
 1
 1

 2.93786720541504E-02
 1.99144354193786E-02
 2.85071151883208E-02
 4.21953967427297E-02
 3.43909718609451E-02
 1.07023962986331E-02
 2.77859370197915E-02
 3.98464969039385E-03
 7.88630567597099E-04
 2.79950956696965E-03
 2.23348057419267E-03
 5.09976083212148E-03
 4.03746853842582E-03
 2.56008486165011E-03
 2.80861351416377E-03
 5.44988126748536E-03
 3.60334137594483E-03
 1.22409400569599E-02
 2.9056690452128E-03
 2.8014250229784E-03

 2.93786720541504E-02
 1.99144354193786E-02
 2.85071151883208E-02
 4.21953967427297E-02
 3.43909718609451E-02
 1.07023962986331E-02
 2.77859370197915E-02
 3.98464969039385E-03
 7.88630567597099E-04
 2.79950956696965E-03
 2.23348057419267E-03
 5.09976083212148E-03
 4.03746853842582E-03
 2.56008486165011E-03
 2.80861351416377E-03
 5.44988126748536E-03
 3.60334137594483E-03
 1.22409400569599E-02
 2.9056690452128E-03
 2.8014250229784E-03

 2.93786720541504E-02
 1.99144354193786E-02
 2.85071151883208E-02
 4.21953967427297E-02
 3.43909718609451E-02
 1.07023962986331E-02
 2.77859370197915E-02
 3.98464969039385E-03
 7.88630567597099E-04
 2.79950956696965E-03
 2.23348057419267E-03
 5.09976083212148E-03
 4.03746853842582E-03
 2.56008486165011E-03
 2.80861351416377E-03
 5.44988126748536E-03
 3.60334137594483E-03
 1.22409400569599E-02
 2.9056690452128E-03
 2.8014250229784E-03

 2.93786720541504E-02
 1.99144354193786E-02
 2.85071151883208E-02
 4.21953967427297E-02
 3.43909718609451E-02
 1.07023962986331E-02
 2.77859370197915E-02
 3.98464969039385E-03
 7.88630567597099E-04
 2.79950956696965E-03
 2.23348057419267E-03
 5.09976083212148E-03
 4.03746853842582E-03
 2.56008486165011E-03
 2.80861351416377E-03
 5.44988126748536E-03
 3.60334137594483E-03
 1.22409400569599E-02
 2.9056690452128E-03
 2.8014250229784E-03

 2.93786720541504E-02
 1.99144354193786E-02
 2.85071151883208E-02
 4.21953967427297E-02
 3.43909718609451E-02
 1.07023962986331E-02
 2.77859370197915E-02
 3.98464969039385E-03
 7.88630567597099E-04
 2.79950956696965E-03
 2.23348057419267E-03
 5.09976083212148E-03
 4.03746853842582E-03
 2.56008486165011E-03
 2.80861351416377E-03
 5.44988126748536E-03
 3.60334137594483E-03
 1.22409400569599E-02
 2.9056690452128E-03
 2.8014250229784E-03

 1.10946706544333E-03
 1.37746458107555E-03
 9.86159373433409E-04
 9.64201415703173E-04
 1.0111682914853E-03
 2.80542457354095E-04
 3.95042178913327E-04
 1.5523087162837E-03
 1.98288196209473E-03
 1.67461572278914E-03
 4.97034029295453E-04
 8.8601905366455E-04
 4.36409275189581E-04
 0
 2.25008241759464E-04
 1.05844801475797E-04
 1.46379539297974E-03
 0
 5.97765584722946E-04
 2.72039206776937E-04
 1.53870774093463E-02
 4.90605044997832E-04
 6.48610414849813E-03
 2.56112966395596E-03
 2.85133505815079E-04
 .117409749439199
 2.51551163118659E-02
 1.05869641403837E-02
 1.10141581065115E-03
 2.18957212408759E-03
 .386066119585654
 .160567947209802

 3.43736988972567E-04
 6.10269118197227E-04
 5.0878369154013E-04
 5.63558254711792E-04
 6.59345227360261E-04
 2.80542457354095E-04
 2.50323756935026E-04
 1.00148949437638E-03
 1.84032182102892E-03
 1.23602589063008E-03
 4.97034029295453E-04
 8.8601905366455E-04
 3.42281784462416E-04
 0
 2.25008241759464E-04
 1.05844801475797E-04
 1.07889301872999E-03
 0
 5.97765584722946E-04
 2.72039206776937E-04
 1.04572370743216E-02
 4.90605044997832E-04
 6.48610414849813E-03
 2.56112966395596E-03
 0
 .113407406491267
 1.66575485570593E-02
 1.05869641403837E-02
 1.10141581065115E-03
 2.18957212408759E-03
 .385691159895357
 .159549001680761

 7.52560271715735E-05
 6.10269118197227E-04
 1.44659297543338E-04
 4.55276319308538E-04
 3.65530456233259E-04
 2.80542457354095E-04
 0
 1.00148949437638E-03
 1.55520153889761E-03
 7.9743605847102E-04
 4.97034029295453E-04
 3.47710965827425E-04
 3.42281784462416E-04
 0
 2.25008241759464E-04
 1.05844801475797E-04
 4.37389061646721E-04
 0
 3.44864760416924E-04
 2.72039206776937E-04
 1.04572370743216E-02
 4.90605044997832E-04
 5.46077911634594E-03
 0
 0
 .110594328638649
 1.55564092162354E-02
 1.05869641403837E-02
 6.35432198452905E-04
 2.23425726947446E-04
 .382884974660326
 .158029188413162

 7.52560271715735E-05
 6.10269118197227E-04
 1.44659297543338E-04
 4.55276319308538E-04
 3.65530456233259E-04
 2.80542457354095E-04
 0
 1.00148949437638E-03
 1.55520153889761E-03
 7.9743605847102E-04
 4.97034029295453E-04
 3.47710965827425E-04
 3.42281784462416E-04
 0
 2.25008241759464E-04
 1.05844801475797E-04
 4.37389061646721E-04
 0
 3.44864760416924E-04
 2.72039206776937E-04
 1.04572370743216E-02
 4.90605044997832E-04
 5.46077911634594E-03
 0
 0
 .109442057164662
 1.54501231905339E-02
 1.05869641403837E-02
 6.35432198452905E-04
 2.23425726947446E-04
 .382884974660326
 .158029188413162

 7.52560271715735E-05
 6.10269118197227E-04
 1.44659297543338E-04
 2.73165791585183E-04
 6.09217427057107E-05
 1.40271228677048E-04
 0
 2.50372373594233E-04
 1.03680102593103E-03
 7.9743605847102E-04
 3.97627223436363E-04
 2.31807310551489E-04
 6.84563568924833E-05
 0
 1.50005494506227E-04
 0
 4.37389061646721E-04
 0
 2.29909840277823E-04
 1.81359471184525E-04
 0
 0
 0
 0
 0
 9.57498312897472E-05
 1.88416136470064E-04
 0
 0
 0
 5.11308668587037E-04
 6.48419882116847E-04

 0
 0
 0
 0
 0
 0
 0
 0
 0
 0
 0
 0
 0
 0
 0
 0
 0
 0
 0
 0
 1.04572370743216E-02

 0
 0
 0
 0
 0
 0
 0
 0
 0
 0
 0
 0
 0
 0
 0
 0
 0
 0
 0
 0
 0
 4.90605044997832E-04

 0
 0
 0
 0
 0
 0
 0
 0
 0
 0
 0
 0
 0
 0
 0
 0
 0
 0
 0
 0
 0
 0
 5.46077911634594E-03

 0
 0
 0
 1.82110527723355E-04
 0
 0
 0
 0
 0
 0
 0
 0
 0
 0
 0
 0
 0
 0
 0
 0
 0
 0
 0
 0

 0
 0
 0
 0
 3.04608713527548E-04
 0
 0
 0
 0
 0
 0
 0
 0
 0
 0
 0
 0
 0
 0
 0
 0
 0
 0
 0
 0

 0
 0
 0
 0
 0
 1.40271228677048E-04
 0
 0
 0
 0
 0
 0
 0
 0
 0
 0
 0
 0
 0
 0
 0
 0
 0
 0
 0
 .109346307333372

 0
 0
 0
 0
 0
 0
 0
 0
 0
 0
 0
 0
 0
 0
 0
 0
 0
 0
 0
 0
 0
 0
 0
 0
 0
 0
 1.52617070540638E-02

 0
 0
 0
 0
 0
 0
 0
 7.51117120782149E-04
 0
 0
 0
 0
 0
 0
 0
 0
 0
 0
 0
 0
 0
 0
 0
 0
 0
 0
 0
 1.05869641403837E-02

 0
 0
 0
 0
 0
 0
 0
 0
 5.18400512966583E-04
 0
 0
 0
 0
 0
 0
 0
 0
 0
 0
 0
 0
 0
 0
 0
 0
 0
 0
 0
 6.35432198452905E-04

 0
 0
 0
 0
 0
 0
 0
 0
 0
 0
 0
 0
 0
 0
 0
 0
 0
 0
 0
 0
 0
 0
 0
 0
 0
 0
 0
 0
 0
 2.23425726947446E-04

 0
 0
 0
 0
 0
 0
 0
 0
 0
 0
 9.94068058590906E-05
 0
 0
 0
 0
 0
 0
 0
 0
 0
 0
 0
 0
 0
 0
 0
 0
 0
 0
 0
 .382373665991739

 0
 0
 0
 0
 0
 0
 0
 0
 0
 0
 0
 1.15903655275936E-04
 0
 0
 0
 0
 0
 0
 0
 0
 0
 0
 0
 0
 0
 0
 0
 0
 0
 0
 0
 .157380768531045

 0
 0
 0
 0
 0
 0
 0
 0
 0
 0
 0
 0
 2.73825427569933E-04

 0
 0
 0
 0
 0
 0
 0
 0
 0
 0
 0
 0
 0
 0

 0
 0
 0
 0
 0
 0
 0
 0
 0
 0
 0
 0
 0
 0
 7.50027472532371E-05

 0
 0
 0
 0
 0
 0
 0
 0
 0
 0
 0
 0
 0
 0
 0
 1.05844801475797E-04

 0
 0
 0
 0
 0
 0
 0
 0
 0
 0
 0
 0
 0
 0
 0
 0
 0

 0
 0
 0
 0
 0
 0
 0
 0
 0
 0
 0
 0
 0
 0
 0
 0
 0
 0

 0
 0
 0
 0
 0
 0
 0
 0
 0
 0
 0
 0
 0
 0
 0
 0
 0
 0
 1.14954920139101E-04

 0
 0
 0
 0
 0
 0
 0
 0
 0
 0
 0
 0
 0
 0
 0
 0
 0
 0
 0
 9.06797355924121E-05

 0
 0
 0
 0
 0
 0
 0
 0
 0
 0
 0
 0
 0
 0
 0
 0
 0
 0
 0
 0
 0
 0
 0
 0
 0
 1.15227147398715E-03
 1.06286025701555E-04
 0
 0
 0
 0
 0

 0
 0
 0
 0
 0
 0
 0
 0
 0
 0
 0
 0
 0
 0
 0
 0
 0
 0
 0
 0
 0
 0
 0
 0
 0
 9.36220572614559E-04
 0
 0
 0
 0
 0
 0

 0
 0
 0
 0
 0
 0
 0
 0
 0
 0
 0
 0
 0
 0
 0
 0
 0
 0
 0
 0
 0
 0
 0
 0
 0
 2.16050901372591E-04
 1.06286025701555E-04
 0
 0
 0
 0
 0

 0
 0
 0
 0
 0
 0
 0
 0
 0
 4.38589832159061E-04
 0
 2.54988041607059E-04
 0
 0
 0
 0
 0
 0
 2.52900824306023E-04
 0
 0
 0
 1.02532503215219E-03
 0
 0
 2.3847127792981E-04
 2.07257750118031E-04
 0
 0
 1.96614639714014E-03
 3.28974067902225E-04
 2.03789105808962E-04

 0
 0
 0
 0
 0
 0
 0
 0
 0
 4.38589832159061E-04
 0
 2.54988041607059E-04
 0
 0
 0
 0
 0
 0
 2.52900824306023E-04
 0
 0
 0
 0
 0
 0
 0
 0
 0
 0
 0
 0
 0

 0
 0
 0
 0
 0
 0
 0
 0
 0
 4.38589832159061E-04
 0
 2.54988041607059E-04
 0
 0
 0
 0
 0
 0
 2.52900824306023E-04
 0
 0
 0
 0
 0
 0
 0
 0
 0
 0
 0
 0
 0

 0
 0
 0
 0
 0
 0
 0
 0
 0
 0
 0
 0
 0
 0
 0
 0
 0
 0
 0
 0
 0
 0
 0
 0
 0
 2.3847127792981E-04
 0
 0
 0
 0
 1.41494222753645E-04
 0

 0
 0
 0
 0
 0
 0
 0
 0
 0
 0
 0
 0
 0
 0
 0
 0
 0
 0
 0
 0
 0
 0
 0
 0
 0
 2.3847127792981E-04
 0
 0
 0
 0
 1.41494222753645E-04
 0

 0
 0
 0
 0
 0
 0
 0
 0
 0
 0
 0
 0
 0
 0
 0
 0
 0
 0
 0
 0
 0
 0
 0
 0
 0
 0
 0
 0
 0
 1.96614639714014E-03
 0
 0

 0
 0
 0
 0
 0
 0
 0
 0
 0
 0
 0
 0
 0
 0
 0
 0
 0
 0
 0
 0
 0
 0
 0
 0
 0
 0
 0
 0
 0
 1.96614639714014E-03
 0
 0

 0
 0
 0
 0
 0
 0
 0
 0
 0
 0
 0
 0
 0
 0
 0
 0
 0
 0
 0
 0
 0
 0
 4.44952372443924E-04
 0
 0
 0
 2.07257750118031E-04
 0
 0
 0
 1.8747984514858E-04
 2.03789105808962E-04

 0
 0
 0
 0
 0
 0
 0
 0
 0
 0
 0
 0
 0
 0
 0
 0
 0
 0
 0
 0
 0
 0
 4.44952372443924E-04
 0
 0
 0
 2.07257750118031E-04
 0
 0
 0
 1.8747984514858E-04
 2.03789105808962E-04

 0
 0
 0
 0
 0
 0
 0
 0
 0
 0
 0
 0
 0
 0
 0
 0
 0
 0
 0
 0
 0
 0
 5.80372659708265E-04
 0
 0
 0
 0
 0
 0
 0
 0
 0

 0
 0
 0
 0
 0
 0
 0
 0
 0
 0
 0
 0
 0
 0
 0
 0
 0
 0
 0
 0
 0
 0
 5.80372659708265E-04
 0
 0
 0
 0
 0
 0
 0
 0
 0

 0
 0
 1.0608348486529E-04
 0
 1.48919815502848E-04
 0
 0
 0
 2.85120282131307E-04
 0
 0
 2.83320046230066E-04
 0
 0
 0
 0
 6.4150395708327E-04
 0
 0
 0
 0
 0
 0
 2.56112966395596E-03
 0
 2.15330731701201E-03
 2.7634366682276E-04
 0
 4.6598361219825E-04
 0
 1.66648751243182E-03
 9.96302295063936E-04

 0
 0
 1.0608348486529E-04
 0
 1.48919815502848E-04
 0
 0
 0
 2.85120282131307E-04
 0
 0
 2.83320046230066E-04
 0
 0
 0
 0
 6.4150395708327E-04
 0
 0
 0
 0
 0
 0
 2.56112966395596E-03
 0
 2.15330731701201E-03
 2.7634366682276E-04
 0
 4.6598361219825E-04
 0
 1.66648751243182E-03
 9.96302295063936E-04

 0
 0
 1.0608348486529E-04
 0
 0
 0
 0
 0
 2.85120282131307E-04
 0
 0
 0
 0
 0
 0
 0
 0
 0
 0
 0
 0
 0
 0
 0
 0
 9.83031601244732E-04
 2.7634366682276E-04
 0
 4.6598361219825E-04
 0
 6.24932817161934E-04
 1.35859403872641E-04

 0
 0
 0
 0
 1.48919815502848E-04
 0
 0
 0
 0
 0
 0
 2.83320046230066E-04
 0
 0
 0
 0
 6.4150395708327E-04
 0
 0
 0
 0
 0
 0
 0
 0
 1.17027571576727E-03
 0
 0
 0
 0
 1.04155469526989E-03
 4.52864679573371E-04

 0
 0
 0
 0
 0
 0
 0
 0
 0
 0
 0
 0
 0
 0
 0
 0
 0
 0
 0
 0
 0
 0
 0
 2.56112966395596E-03
 0
 0
 0
 0
 0
 0
 0
 4.07578211617924E-04

 2.68480961800994E-04
 0
 2.58040909131502E-04
 1.08281935403255E-04
 1.44894955624154E-04
 0
 2.50323756935026E-04
 0
 0
 0
 0
 0
 0
 0
 0
 0
 0
 0
 0
 0
 0
 0
 0
 0
 0
 0
 1.12031216280017E-04
 0
 0
 0
 8.10723654696563E-04
 2.20312546819478E-04

 2.68480961800994E-04
 0
 2.58040909131502E-04
 1.08281935403255E-04
 1.44894955624154E-04
 0
 2.50323756935026E-04
 0
 0
 0
 0
 0
 0
 0
 0
 0
 0
 0
 0
 0
 0
 0
 0
 0
 0
 0
 1.12031216280017E-04
 0
 0
 0
 8.10723654696563E-04
 2.20312546819478E-04

 2.68480961800994E-04
 0
 2.58040909131502E-04
 1.08281935403255E-04
 1.44894955624154E-04
 0
 2.50323756935026E-04
 0
 0
 0
 0
 0
 0
 0
 0
 0
 0
 0
 0
 0
 0
 0
 0
 0
 0
 0
 1.12031216280017E-04
 0
 0
 0
 8.10723654696563E-04
 2.20312546819478E-04

 0
 0
 0
 0
 0
 0
 0
 0
 0
 0
 0
 0
 0
 0
 0
 0
 0
 0
 0
 0
 0
 0
 0
 0
 0
 0
 5.05506707603079E-04
 0
 0
 0
 0
 9.94093199068106E-05

 0
 0
 0
 0
 0
 0
 0
 0
 0
 0
 0
 0
 0
 0
 0
 0
 0
 0
 0
 0
 0
 0
 0
 0
 0
 0
 5.05506707603079E-04
 0
 0
 0
 0
 9.94093199068106E-05

 0
 0
 0
 0
 0
 0
 0
 0
 0
 0
 0
 0
 0
 0
 0
 0
 0
 0
 0
 0
 0
 0
 0
 0
 0
 0
 5.05506707603079E-04
 0
 0
 0
 0
 9.94093199068106E-05

 0
 0
 0
 0
 0
 0
 0
 0
 0
 0
 0
 0
 0
 0
 0
 0
 0
 0
 0
 0
 0
 0
 0
 0
 0
 4.21299257676219E-04
 0
 0
 0
 0
 0
 0

 0
 0
 0
 0
 0
 0
 0
 0
 0
 0
 0
 0
 0
 0
 0
 0
 0
 0
 0
 0
 0
 0
 0
 0
 0
 4.21299257676219E-04
 0
 0
 0
 0
 0
 0

 0
 0
 0
 0
 0
 0
 0
 0
 0
 0
 0
 0
 0
 0
 0
 0
 0
 0
 0
 0
 0
 0
 0
 0
 0
 4.21299257676219E-04
 0
 0
 0
 0
 0
 0

 6.62253039109847E-04
 7.67195462878323E-04
 4.77375681893279E-04
 4.00643160991381E-04
 2.68055667904685E-04
 0
 0
 5.50819221907314E-04
 1.4256014106581E-04
 4.38589832159061E-04
 0
 0
 0
 0
 0
 0
 3.8490237424975E-04
 0
 0
 0
 4.92984033502473E-03
 0
 0
 0
 0
 4.0023429479314E-03
 8.4975677548066E-03
 0
 0
 0
 3.7495969029716E-04
 1.01894552904103E-03

 6.62253039109847E-04
 7.67195462878323E-04
 4.77375681893279E-04
 4.00643160991381E-04
 2.68055667904685E-04
 0
 0
 5.50819221907314E-04
 1.4256014106581E-04
 4.38589832159061E-04
 0
 0
 0
 0
 0
 0
 3.8490237424975E-04
 0
 0
 0
 4.92984033502473E-03
 0
 0
 0
 0
 4.0023429479314E-03
 8.4975677548066E-03
 0
 0
 0
 3.7495969029716E-04
 1.01894552904103E-03

 6.62253039109847E-04
 7.67195462878323E-04
 4.77375681893279E-04
 4.00643160991381E-04
 2.68055667904685E-04
 0
 0
 5.50819221907314E-04
 1.4256014106581E-04
 4.38589832159061E-04
 0
 0
 0
 0
 0
 0
 3.8490237424975E-04
 0
 0
 0
 4.92984033502473E-03
 0
 0
 0
 0
 4.0023429479314E-03
 8.4975677548066E-03
 0
 0
 0
 3.7495969029716E-04
 1.01894552904103E-03

 6.62253039109847E-04
 7.67195462878323E-04
 4.77375681893279E-04
 4.00643160991381E-04
 2.68055667904685E-04
 0
 0
 5.50819221907314E-04
 1.4256014106581E-04
 4.38589832159061E-04
 0
 0
 0
 0
 0
 0
 3.8490237424975E-04
 0
 0
 0
 4.92984033502473E-03
 0
 0
 0
 0
 4.0023429479314E-03
 8.4975677548066E-03
 0
 0
 0
 3.7495969029716E-04
 1.01894552904103E-03

 1.03477037360914E-04
 0
 0
 0
 0
 0
 0
 0
 0
 0
 0
 0
 0
 0
 0
 0
 0
 0
 0
 0
 0

 1.03477037360914E-04
 0
 0
 0
 0
 0
 0
 0
 0
 0
 0
 0
 0
 0
 0
 0
 0
 0
 0
 0
 0

 1.03477037360914E-04
 0
 0
 0
 0
 0
 0
 0
 0
 0
 0
 0
 0
 0
 0
 0
 0
 0
 0
 0
 0

 1.03477037360914E-04
 0
 0
 0
 0
 0
 0
 0
 0
 0
 0
 0
 0
 0
 0
 0
 0
 0
 0
 0
 0

 0
 0
 0
 0
 0
 0
 0
 0
 0
 0
 0
 0
 0
 0
 0
 0
 0
 0
 0
 0
 0
 0

 0
 0
 0
 0
 0
 0
 0
 0
 0
 0
 0
 0
 0
 0
 0
 0
 0
 0
 0
 0
 0
 0

 0
 0
 0
 0
 0
 0
 0
 0
 0
 0
 0
 0
 0
 0
 0
 0
 0
 0
 0
 0
 0
 0

 0
 0
 0
 0
 0
 0
 0
 0
 0
 0
 0
 0
 0
 0
 0
 0
 0
 0
 0
 0
 0
 0

 0
 0
 0
 0
 0
 0
 0
 0
 0
 0
 0
 0
 0
 0
 0
 0
 0
 0
 0
 0
 0
 0
 0

 0
 0
 0
 0
 0
 0
 0
 0
 0
 0
 0
 0
 0
 0
 0
 0
 0
 0
 0
 0
 0
 0
 0

 0
 0
 0
 0
 0
 0
 0
 0
 0
 0
 0
 0
 0
 0
 0
 0
 0
 0
 0
 0
 0
 0
 0

 0
 0
 0
 0
 0
 0
 0
 0
 0
 0
 0
 0
 0
 0
 0
 0
 0
 0
 0
 0
 0
 0
 0

 0
 0
 0
 0
 0
 0
 0
 0
 0
 0
 0
 0
 0
 0
 0
 0
 0
 0
 0
 0
 0
 0
 0
 0

 0
 0
 0
 0
 0
 0
 0
 0
 0
 0
 0
 0
 0
 0
 0
 0
 0
 0
 0
 0
 0
 0
 0
 0

 0
 0
 0
 0
 0
 0
 0
 0
 0
 0
 0
 0
 0
 0
 0
 0
 0
 0
 0
 0
 0
 0
 0
 0

 0
 0
 0
 0
 0
 0
 0
 0
 0
 0
 0
 0
 0
 0
 0
 0
 0
 0
 0
 0
 0
 0
 0
 0

 0
 0
 0
 0
 8.37673962203522E-05
 0
 0
 0
 0
 0
 0
 0
 0
 0
 0
 0
 0
 0
 0
 0
 0
 0
 0
 0
 2.85133505815079E-04

 0
 0
 0
 0
 8.37673962203522E-05
 0
 0
 0
 0
 0
 0
 0
 0
 0
 0
 0
 0
 0
 0
 0
 0
 0
 0
 0
 2.85133505815079E-04

 0
 0
 0
 0
 8.37673962203522E-05
 0
 0
 0
 0
 0
 0
 0
 0
 0
 0
 0
 0
 0
 0
 0
 0
 0
 0
 0
 2.85133505815079E-04

 0
 0
 0
 0
 8.37673962203522E-05
 0
 0
 0
 0
 0
 0
 0
 0
 0
 0
 0
 0
 0
 0
 0
 0
 0
 0
 0
 2.85133505815079E-04

 0
 0
 0
 0
 0
 0
 0
 0
 0
 0
 0
 0
 0
 0
 0
 0
 0
 0
 0
 0
 0
 0
 0
 0
 0
 0

 0
 0
 0
 0
 0
 0
 0
 0
 0
 0
 0
 0
 0
 0
 0
 0
 0
 0
 0
 0
 0
 0
 0
 0
 0
 0

 0
 0
 0
 0
 0
 0
 0
 0
 0
 0
 0
 0
 0
 0
 0
 0
 0
 0
 0
 0
 0
 0
 0
 0
 0
 0

 0
 0
 0
 0
 0
 0
 0
 0
 0
 0
 0
 0
 0
 0
 0
 0
 0
 0
 0
 0
 0
 0
 0
 0
 0
 0

 0
 0
 0
 0
 0
 0
 1.44718421978301E-04
 0
 0
 0
 0
 0
 0
 0
 0
 0
 0
 0
 0
 0
 0
 0
 0
 0
 0
 0
 0

 0
 0
 0
 0
 0
 0
 1.44718421978301E-04
 0
 0
 0
 0
 0
 0
 0
 0
 0
 0
 0
 0
 0
 0
 0
 0
 0
 0
 0
 0

 0
 0
 0
 0
 0
 0
 1.44718421978301E-04
 0
 0
 0
 0
 0
 0
 0
 0
 0
 0
 0
 0
 0
 0
 0
 0
 0
 0
 0
 0

 0
 0
 0
 0
 0
 0
 1.44718421978301E-04
 0
 0
 0
 0
 0
 0
 0
 0
 0
 0
 0
 0
 0
 0
 0
 0
 0
 0
 0
 0

 0
 0
 0
 0
 0
 0
 0
 0
 0
 0
 0
 0
 0
 0
 0
 0
 0
 0
 0
 0
 0
 0
 0
 0
 0
 0
 0
 0

 0
 0
 0
 0
 0
 0
 0
 0
 0
 0
 0
 0
 0
 0
 0
 0
 0
 0
 0
 0
 0
 0
 0
 0
 0
 0
 0
 0

 0
 0
 0
 0
 0
 0
 0
 0
 0
 0
 0
 0
 0
 0
 0
 0
 0
 0
 0
 0
 0
 0
 0
 0
 0
 0
 0
 0

 0
 0
 0
 0
 0
 0
 0
 0
 0
 0
 0
 0
 0
 0
 0
 0
 0
 0
 0
 0
 0
 0
 0
 0
 0
 0
 0
 0

 0
 0
 0
 0
 0
 0
 0
 0
 0
 0
 0
 0
 0
 0
 0
 0
 0
 0
 0
 0
 0
 0
 0
 0
 0
 0
 0
 0
 0

 0
 0
 0
 0
 0
 0
 0
 0
 0
 0
 0
 0
 0
 0
 0
 0
 0
 0
 0
 0
 0
 0
 0
 0
 0
 0
 0
 0
 0

 0
 0
 0
 0
 0
 0
 0
 0
 0
 0
 0
 0
 0
 0
 0
 0
 0
 0
 0
 0
 0
 0
 0
 0
 0
 0
 0
 0
 0

 0
 0
 0
 0
 0
 0
 0
 0
 0
 0
 0
 0
 0
 0
 0
 0
 0
 0
 0
 0
 0
 0
 0
 0
 0
 0
 0
 0
 0

 0
 0
 0
 0
 0
 0
 0
 0
 0
 0
 0
 0
 0
 0
 0
 0
 0
 0
 0
 0
 0
 0
 0
 0
 0
 0
 0
 0
 0
 0

 0
 0
 0
 0
 0
 0
 0
 0
 0
 0
 0
 0
 0
 0
 0
 0
 0
 0
 0
 0
 0
 0
 0
 0
 0
 0
 0
 0
 0
 0

 0
 0
 0
 0
 0
 0
 0
 0
 0
 0
 0
 0
 0
 0
 0
 0
 0
 0
 0
 0
 0
 0
 0
 0
 0
 0
 0
 0
 0
 0

 0
 0
 0
 0
 0
 0
 0
 0
 0
 0
 0
 0
 0
 0
 0
 0
 0
 0
 0
 0
 0
 0
 0
 0
 0
 0
 0
 0
 0
 0

 0
 0
 0
 0
 0
 0
 0
 0
 0
 0
 0
 0
 0
 0
 0
 0
 0
 0
 0
 0
 0
 0
 0
 0
 0
 0
 0
 0
 0
 0
 0

 0
 0
 0
 0
 0
 0
 0
 0
 0
 0
 0
 0
 0
 0
 0
 0
 0
 0
 0
 0
 0
 0
 0
 0
 0
 0
 0
 0
 0
 0
 0

 0
 0
 0
 0
 0
 0
 0
 0
 0
 0
 0
 0
 0
 0
 0
 0
 0
 0
 0
 0
 0
 0
 0
 0
 0
 0
 0
 0
 0
 0
 0

 0
 0
 0
 0
 0
 0
 0
 0
 0
 0
 0
 0
 0
 0
 0
 0
 0
 0
 0
 0
 0
 0
 0
 0
 0
 0
 0
 0
 0
 0
 0

 0
 0
 0
 0
 0
 0
 0
 0
 0
 0
 0
 0
 0
 0
 0
 0
 0
 0
 0
 0
 0
 0
 0
 0
 0
 0
 0
 0
 0
 0
 0
 0

 0
 0
 0
 0
 0
 0
 0
 0
 0
 0
 0
 0
 0
 0
 0
 0
 0
 0
 0
 0
 0
 0
 0
 0
 0
 0
 0
 0
 0
 0
 0
 0

 0
 0
 0
 0
 0
 0
 0
 0
 0
 0
 0
 0
 0
 0
 0
 0
 0
 0
 0
 0
 0
 0
 0
 0
 0
 0
 0
 0
 0
 0
 0
 0

 0
 0
 0
 0
 0
 0
 0
 0
 0
 0
 0
 0
 0
 0
 0
 0
 0
 0
 0
 0
 0
 0
 0
 0
 0
 0
 0
 0
 0
 0
 0
 0

 0
 0
 0
 0
 0
 0
 0
 0
 0
 0
 0
 0
 9.41274907271645E-05

 0
 0
 0
 0
 0
 0
 0
 0
 0
 0
 0
 0
 9.41274907271645E-05

 0
 0
 0
 0
 0
 0
 0
 0
 0
 0
 0
 0
 9.41274907271645E-05

 0
 0
 0
 0
 0
 0
 0
 0
 0
 0
 0
 0
 9.41274907271645E-05

 0
 0
 0
 0
 0
 0
 0
 0
 0
 0
 0
 0
 0
 0

 0
 0
 0
 0
 0
 0
 0
 0
 0
 0
 0
 0
 0
 0

 0
 0
 0
 0
 0
 0
 0
 0
 0
 0
 0
 0
 0
 0

 0
 0
 0
 0
 0
 0
 0
 0
 0
 0
 0
 0
 0
 0

 0
 0
 0
 0
 0
 0
 0
 0
 0
 0
 0
 0
 0
 0
 0

 0
 0
 0
 0
 0
 0
 0
 0
 0
 0
 0
 0
 0
 0
 0

 0
 0
 0
 0
 0
 0
 0
 0
 0
 0
 0
 0
 0
 0
 0

 0
 0
 0
 0
 0
 0
 0
 0
 0
 0
 0
 0
 0
 0
 0

 0
 0
 0
 0
 0
 0
 0
 0
 0
 0
 0
 0
 0
 0
 0
 0

 0
 0
 0
 0
 0
 0
 0
 0
 0
 0
 0
 0
 0
 0
 0
 0

 0
 0
 0
 0
 0
 0
 0
 0
 0
 0
 0
 0
 0
 0
 0
 0

 0
 0
 0
 0
 0
 0
 0
 0
 0
 0
 0
 0
 0
 0
 0
 0

 0
 0
 0
 0
 0
 0
 0
 0
 0
 0
 0
 0
 0
 0
 0
 0
 0

 0
 0
 0
 0
 0
 0
 0
 0
 0
 0
 0
 0
 0
 0
 0
 0
 0

 0
 0
 0
 0
 0
 0
 0
 0
 0
 0
 0
 0
 0
 0
 0
 0
 0

 0
 0
 0
 0
 0
 0
 0
 0
 0
 0
 0
 0
 0
 0
 0
 0
 0

 0
 0
 0
 0
 0
 0
 0
 0
 0
 0
 0
 0
 0
 0
 0
 0
 0
 0

 0
 0
 0
 0
 0
 0
 0
 0
 0
 0
 0
 0
 0
 0
 0
 0
 0
 0

 0
 0
 0
 0
 0
 0
 0
 0
 0
 0
 0
 0
 0
 0
 0
 0
 0
 0

 0
 0
 0
 0
 0
 0
 0
 0
 0
 0
 0
 0
 0
 0
 0
 0
 0
 0

 0
 0
 0
 0
 0
 0
 0
 0
 0
 0
 0
 0
 0
 0
 0
 0
 0
 0
 0

 0
 0
 0
 0
 0
 0
 0
 0
 0
 0
 0
 0
 0
 0
 0
 0
 0
 0
 0

 0
 0
 0
 0
 0
 0
 0
 0
 0
 0
 0
 0
 0
 0
 0
 0
 0
 0
 0

 0
 0
 0
 0
 0
 0
 0
 0
 0
 0
 0
 0
 0
 0
 0
 0
 0
 0
 0

 0
 0
 0
 0
 0
 0
 0
 0
 0
 0
 0
 0
 0
 0
 0
 0
 0
 0
 0
 0

 0
 0
 0
 0
 0
 0
 0
 0
 0
 0
 0
 0
 0
 0
 0
 0
 0
 0
 0
 0

 0
 0
 0
 0
 0
 0
 0
 0
 0
 0
 0
 0
 0
 0
 0
 0
 0
 0
 0
 0

 0
 0
 0
 0
 0
 0
 0
 0
 0
 0
 0
 0
 0
 0
 0
 0
 0
 0
 0
 0

 1.65563259777462E-04
 1.91798865719581E-04
 4.77375681893279E-04
 6.00964741487402E-04
 2.68055667904685E-04
 0
 0
 0
 0
 0
 0
 0
 0
 0
 0
 0
 0
 0
 0
 0
 4.10820027919462E-04
 0
 0
 1.28056483197798E-03
 0
 0
 0
 0
 0
 0
 0
 0

 1.65563259777462E-04
 1.91798865719581E-04
 4.77375681893279E-04
 6.00964741487402E-04
 2.68055667904685E-04
 0
 0
 0
 0
 0
 0
 0
 0
 0
 0
 0
 0
 0
 0
 0
 4.10820027919462E-04
 0
 0
 1.28056483197798E-03
 0
 0
 0
 0
 0
 0
 0
 0

 1.65563259777462E-04
 1.91798865719581E-04
 4.77375681893279E-04
 6.00964741487402E-04
 2.68055667904685E-04
 0
 0
 0
 0
 0
 0
 0
 0
 0
 0
 0
 0
 0
 0
 0
 4.10820027919462E-04
 0
 0
 1.28056483197798E-03
 0
 0
 0
 0
 0
 0
 0
 0

 1.65563259777462E-04
 1.91798865719581E-04
 4.77375681893279E-04
 6.00964741487402E-04
 2.68055667904685E-04
 0
 0
 0
 0
 0
 0
 0
 0
 0
 0
 0
 0
 0
 0
 0
 4.10820027919462E-04
 0
 0
 1.28056483197798E-03
 0
 0
 0
 0
 0
 0
 0
 0

 1.65563259777462E-04
 1.91798865719581E-04
 4.77375681893279E-04
 6.00964741487402E-04
 2.68055667904685E-04
 0
 0
 0
 0
 0
 0
 0
 0
 0
 0
 0
 0
 0
 0
 0
 4.10820027919462E-04
 0
 0
 1.28056483197798E-03
 0
 0
 0
 0
 0
 0
 0
 0

 2.81809803876298E-04
 5.71315770228539E-04
 5.41702901439778E-04
 1.02291870891445E-03
 7.41430570800474E-04
 3.28294364988293E-04
 4.92658457796845E-04
 3.12521544344919E-04
 6.06638898150382E-04
 0
 9.30616905914891E-05
 4.34022198480101E-04
 7.04954824168344E-04
 4.26680810275331E-04
 2.10646013562051E-04
 4.95443751587199E-04
 2.45682366542529E-04
 1.34515824801831E-04
 4.30469488180464E-04
 1.69783334725938E-04
 1.74817033157218E-04
 4.59289829360559E-04
 0
 1.08984241019402E-03
 9.70667253838567E-04
 8.96381399311812E-04
 6.17363510987426E-04
 5.66354312674866E-04
 0
 4.18329020668116E-04
 0
 1.73437536857887E-04

 7.04524509691326E-05
 8.1616538604077E-05
 0
 8.52432257429877E-05
 0
 0
 0
 0
 3.03319449075191E-04
 0
 0
 0
 0
 0
 0
 0
 0
 0
 0
 0
 0
 2.29644914680279E-04
 0
 5.44921205097012E-04
 1.94133450767713E-04
 3.58552559724725E-04
 2.64584361851988E-04
 4.24765734506149E-04
 0
 0
 0
 0

 7.04524509691326E-05
 8.1616538604077E-05
 0
 8.52432257429877E-05
 0
 0
 0
 0
 3.03319449075191E-04
 0
 0
 0
 0
 0
 0
 0
 0
 0
 0
 0
 0
 2.29644914680279E-04
 0
 5.44921205097012E-04
 1.94133450767713E-04
 3.58552559724725E-04
 2.64584361851988E-04
 4.24765734506149E-04
 0
 0
 0
 0

 7.04524509691326E-05
 8.1616538604077E-05
 0
 8.52432257429877E-05
 0
 0
 0
 0
 3.03319449075191E-04
 0
 0
 0
 0
 0
 0
 0
 0
 0
 0
 0
 0
 2.29644914680279E-04
 0
 5.44921205097012E-04
 1.94133450767713E-04
 3.58552559724725E-04
 2.64584361851988E-04
 4.24765734506149E-04
 0
 0
 0
 0

 7.04524509691326E-05
 8.1616538604077E-05
 0
 8.52432257429877E-05
 0
 0
 0
 0
 3.03319449075191E-04
 0
 0
 0
 0
 0
 0
 0
 0
 0
 0
 0
 0
 2.29644914680279E-04
 0
 5.44921205097012E-04
 1.94133450767713E-04
 3.58552559724725E-04
 2.64584361851988E-04
 4.24765734506149E-04
 0
 0
 0
 0

 2.11357352907165E-04
 4.89699231624462E-04
 5.41702901439778E-04
 9.37675483171458E-04
 7.41430570800474E-04
 3.28294364988293E-04
 4.92658457796845E-04
 3.12521544344919E-04
 3.03319449075191E-04
 0
 9.30616905914891E-05
 4.34022198480101E-04
 7.04954824168344E-04
 4.26680810275331E-04
 2.10646013562051E-04
 4.95443751587199E-04
 2.45682366542529E-04
 1.34515824801831E-04
 4.30469488180464E-04
 1.69783334725938E-04
 1.74817033157218E-04
 2.29644914680279E-04
 0
 5.44921205097012E-04
 7.76533803070853E-04
 5.37828839587087E-04
 3.52779149135438E-04
 1.41588578168716E-04
 0
 4.18329020668116E-04
 0
 1.73437536857887E-04

 2.11357352907165E-04
 4.89699231624462E-04
 5.41702901439778E-04
 9.37675483171458E-04
 7.41430570800474E-04
 3.28294364988293E-04
 4.92658457796845E-04
 3.12521544344919E-04
 3.03319449075191E-04
 0
 9.30616905914891E-05
 4.34022198480101E-04
 7.04954824168344E-04
 4.26680810275331E-04
 2.10646013562051E-04
 4.95443751587199E-04
 2.45682366542529E-04
 1.34515824801831E-04
 4.30469488180464E-04
 1.69783334725938E-04
 1.74817033157218E-04
 2.29644914680279E-04
 0
 5.44921205097012E-04
 7.76533803070853E-04
 5.37828839587087E-04
 3.52779149135438E-04
 1.41588578168716E-04
 0
 4.18329020668116E-04
 0
 1.73437536857887E-04

 2.11357352907165E-04
 4.89699231624462E-04
 5.41702901439778E-04
 9.37675483171458E-04
 7.41430570800474E-04
 3.28294364988293E-04
 4.92658457796845E-04
 3.12521544344919E-04
 3.03319449075191E-04
 0
 9.30616905914891E-05
 4.34022198480101E-04
 7.04954824168344E-04
 4.26680810275331E-04
 2.10646013562051E-04
 4.95443751587199E-04
 2.45682366542529E-04
 1.34515824801831E-04
 4.30469488180464E-04
 1.69783334725938E-04
 1.74817033157218E-04
 2.29644914680279E-04
 0
 5.44921205097012E-04
 7.76533803070853E-04
 5.37828839587087E-04
 3.52779149135438E-04
 1.41588578168716E-04
 0
 4.18329020668116E-04
 0
 1.73437536857887E-04

 2.11357352907165E-04
 4.89699231624462E-04
 5.41702901439778E-04
 9.37675483171458E-04
 7.41430570800474E-04
 3.28294364988293E-04
 4.92658457796845E-04
 3.12521544344919E-04
 3.03319449075191E-04
 0
 9.30616905914891E-05
 4.34022198480101E-04
 7.04954824168344E-04
 4.26680810275331E-04
 2.10646013562051E-04
 4.95443751587199E-04
 2.45682366542529E-04
 1.34515824801831E-04
 4.30469488180464E-04
 1.69783334725938E-04
 1.74817033157218E-04
 2.29644914680279E-04
 0
 5.44921205097012E-04
 7.76533803070853E-04
 5.37828839587087E-04
 3.52779149135438E-04
 1.41588578168716E-04
 0
 4.18329020668116E-04
 0
 1.73437536857887E-04

 .265415578886605
 .347122745577585
 .290826807664895
 8.19966200176343E-02
 .155539548031504
 .406549664229213
 .352583376657554
 .134605233213525
 7.37454642876624E-02
 .137779843587775
 9.93279766327493E-02
 9.12427946971893E-02
 .189022041662046
 9.49170654153477E-02
 .117260829655245
 5.84169384265679E-02
 .117593302361884
 3.32711342518051E-02
 .103164688627064
 5.88651373292615E-02
 4.00699620365514E-02
 1.41265270134899E-02
 1.12541982846876E-02
 7.41418248764062E-02
 .062517720416938
 3.42743780319392E-02
 1.42976048212918E-02
 .104957681414899
 5.69973599189094E-02
 .201924120637479
 1.58428278774033E-02
 2.33918333481268E-02

 .257348377412013
 .340650561181957
 .279560292186544
 6.41167808464432E-02
 .14078492552901
 .399903921543357
 .335934183802676
 .124196619796745
 5.62841014084257E-02
 .13061622906314
 8.91546506147146E-02
 8.55302745276739E-02
 .179089653214241
 .093569408515627
 .10726178600248
 5.35574695466291E-02
 .106617957667212
 3.12499477560697E-02
 .095439953934307
 5.22962989408911E-02
 3.10462563956063E-02
 1.36201305349641E-02
 9.42875265415689E-03
 .042197660177595
 2.51292725748228E-02
 1.45849790633719E-02
 4.68797291931564E-03
 5.97335242041054E-02
 4.22380859914223E-02
 .164992451826346
 5.14230432406085E-03
 2.26205907447092E-02

 .257190698116987
 .340559228388758
 .279332970433261
 6.41167808464432E-02
 .14078492552901
 .399903921543357
 .335934183802676
 .12410918817422
 5.59446725011273E-02
 .13061622906314
 8.87380887615908E-02
 8.54088516507181E-02
 .178587639930364
 9.34102498006828E-02
 .107026063082541
 5.35574695466291E-02
 .106434670822331
 .031099418142601
 9.51990960063969E-02
 .052106303304412
 3.08506278108827E-02
 1.36201305349641E-02
 9.21687057204073E-03
 .040917095345617
 2.46730589655186E-02
 1.38727826992056E-02
 4.58927875259277E-03
 5.95750798428213E-02
 4.22380859914223E-02
 .163026305429211
 5.05302820732343E-03
 2.26205907447092E-02

 1.57679295025894E-04
 0
 7.57739177609213E-05
 2.86173686422572E-04
 3.89318946241791E-03
 6.61278649477388E-03
 0
 7.33551312984919E-02
 3.42823196372936E-02
 .114868765565641
 4.57176633802916E-02
 6.31398960170662E-02
 8.57725553597297E-02
 7.94201987571114E-02
 4.23515512821631E-02
 .028386567710029
 5.04955257646879E-02
 1.43003132794907E-02
 5.52768944552862E-02
 2.83093498353997E-02
 3.91257169446407E-03
 7.70950784997089E-04
 3.39011331385583E-03
 6.09792777132371E-04
 0
 0
 1.38171833411288E-03
 5.22866392237761E-03
 0
 0
 0
 9.70424313376009E-04

 1.57679295025894E-04
 0
 7.57739177609213E-05
 2.86173686422572E-04
 3.89318946241791E-03
 6.61278649477388E-03
 0
 7.33551312984919E-02
 3.42823196372936E-02
 .114868765565641
 4.57176633802916E-02
 6.31398960170662E-02
 8.57725553597297E-02
 7.94201987571114E-02
 4.23515512821631E-02
 .028386567710029
 5.04955257646879E-02
 1.43003132794907E-02
 5.52768944552862E-02
 2.83093498353997E-02
 3.91257169446407E-03
 7.70950784997089E-04
 3.39011331385583E-03
 6.09792777132371E-04
 0
 0
 1.38171833411288E-03
 5.22866392237761E-03
 0
 0
 0
 9.70424313376009E-04

 0
 0
 0
 0
 0
 0
 0
 0
 6.78857814599097E-05
 0
 5.41530409061639E-03
 2.54988041606859E-03
 9.39482002686011E-03
 3.34233301382448E-03
 1.80720905286242E-03
 1.77416048187636E-03
 1.28300791416493E-03
 1.05370729427852E-03
 7.22573783731493E-04
 6.64984727676121E-04
 0
 3.08380313999232E-03
 3.17823123173573E-03
 0
 0
 6.01856082395074E-04
 9.86941667228721E-05
 6.33777445136159E-04
 6.6569087456971E-04
 0
 0
 0

 0
 0
 0
 0
 0
 0
 0
 0
 6.78857814599097E-05
 0
 5.41530409061639E-03
 2.54988041606859E-03
 9.39482002686011E-03
 3.34233301382448E-03
 1.80720905286242E-03
 1.77416048187636E-03
 1.28300791416493E-03
 1.05370729427852E-03
 7.22573783731493E-04
 6.64984727676121E-04
 0
 3.08380313999232E-03
 3.17823123173573E-03
 0
 0
 6.01856082395074E-04
 9.86941667228721E-05
 6.33777445136159E-04
 6.6569087456971E-04
 0
 0
 0

 7.8839647513077E-05
 0
 1.51547835521592E-04
 2.86173686422572E-04
 6.38227780726493E-04
 7.34754054976224E-05
 8.82093238724882E-04
 2.62294867574911E-04
 6.78857814599097E-05
 8.35409204112498E-04
 2.0828092656156E-04
 3.39984055475678E-03
 2.86864733644692E-04
 9.54952289664887E-04
 0
 2.43947066258136E-03
 0
 1.20423690774972E-03
 0
 1.89995636479026E-04
 7.82514338892814E-04
 7.70950784997089E-04
 4.23764164232308E-04
 6.09792777132371E-04
 2.17244575859108E-04
 0
 0
 3.80266467081695E-03
 3.32845437284464E-04
 0
 1.78552233474838E-04
 0

 7.8839647513077E-05
 0
 1.51547835521592E-04
 2.86173686422572E-04
 6.38227780726493E-04
 7.34754054976224E-05
 8.82093238724882E-04
 2.62294867574911E-04
 6.78857814599097E-05
 8.35409204112498E-04
 2.0828092656156E-04
 3.39984055475678E-03
 2.86864733644692E-04
 9.54952289664887E-04
 0
 2.43947066258136E-03
 0
 1.20423690774972E-03
 0
 1.89995636479026E-04
 7.82514338892814E-04
 7.70950784997089E-04
 4.23764164232308E-04
 6.09792777132371E-04
 2.17244575859108E-04
 0
 0
 3.80266467081695E-03
 3.32845437284464E-04
 0
 1.78552233474838E-04
 0

 1.65563259777462E-03
 2.03423039399555E-03
 2.38687840946639E-03
 3.44795932489751E-03
 2.24638772603281E-02
 1.30265214364635E-02
 1.85239580132225E-03
 8.81310755051459E-03
 1.02643301567313E-02
 1.44734644612273E-02
 3.56472805810013E-02
 1.58092585796124E-02
 7.87658842403943E-02
 9.69276574008206E-03
 5.57720428573758E-02
 2.07244121288956E-02
 4.94599550911078E-02
 1.35928240962302E-02
 3.21184046868189E-02
 1.75555968106778E-02
 3.69738025127222E-03
 7.55531769298119E-03
 1.77980948977293E-03
 1.28056483197798E-03
 2.55756114307571E-03
 5.68753997863761E-03
 1.65806200093656E-03
 3.32733158696484E-02
 0
 2.94921959571284E-03
 2.4372379869243E-03
 1.12763305213807E-02

 1.65563259777462E-03
 1.91798865719581E-03
 2.38687840946639E-03
 3.00482370743701E-03
 2.23826482700538E-02
 1.24981664751125E-02
 1.85239580132225E-03
 8.81310755051459E-03
 1.01217700156655E-02
 1.44734644612273E-02
 3.56472805810013E-02
 1.58092585796124E-02
 7.87658842403943E-02
 9.69276574008206E-03
 5.54420307694621E-02
 2.07244121288956E-02
 4.94599550911078E-02
 1.35928240962302E-02
 3.21184046868189E-02
 1.75555968106778E-02
 3.69738025127222E-03
 7.01565214348253E-03
 1.77980948977293E-03
 1.28056483197798E-03
 2.28106804652775E-03
 4.8449414632845E-03
 1.24354650070242E-03
 3.32733158696484E-02
 0
 2.45768299642846E-03
 2.24975814177572E-03
 1.03932443962117E-02

 0
 0
 0
 2.00321580496021E-04
 0
 1.54298351545007E-04
 0
 0
 1.4256014106581E-04
 0
 0
 0
 0
 0
 3.30012087913698E-04
 0
 0
 0
 0
 0
 0
 5.39665549498656E-04
 0
 0
 0
 8.42598515353103E-04
 4.1451550023414E-04
 0
 0
 4.91536599284381E-04
 1.8747984514858E-04
 6.11367317424996E-04

 0
 1.16241736799746E-04
 0
 2.42814036964473E-04
 8.12289902742809E-05
 3.74056609806078E-04
 0
 0
 0
 0
 0
 0
 0
 0
 0
 0
 0
 0
 0
 0
 0
 0
 0
 0
 2.76493096547955E-04
 0
 0
 0
 0
 0
 0
 0

 0
 0
 0
 0
 0
 0
 0
 0
 0
 0
 0
 0
 0
 0
 0
 0
 0
 0
 0
 0
 0
 0
 0
 0
 0
 0
 0
 0
 0
 0
 0
 2.71718807744023E-04

 .255298546576673
 .338524997994762
 .276718770270512
 6.00964741487005E-02
 .113789631025538
 .380191138206622
 .333199694762628
 4.16786544576386E-02
 1.12622511441825E-02
 4.38589832159061E-04
 .00174955978312
 5.09976083214118E-04
 4.36751556973546E-03
 0
 7.09525989013988E-03
 2.32858563246752E-04
 5.19618205237067E-03
 9.48336564851862E-04
 7.08122308056028E-03
 5.3863762941794E-03
 2.21842815076406E-02
 1.07933109899731E-03
 4.44952372443924E-04
 3.84169449593743E-02
 2.18982532465838E-02
 7.58338663817294E-03
 1.45080425082045E-03
 1.66366579348422E-02
 4.12395496795681E-02
 .159749394767308
 2.4372379869243E-03
 9.57808797298719E-03

 .253974040498457
 .337182405934726
 .274491017088346
 5.80932583437469E-02
 .111377130014397
 .376796574472637
 .332273496861969
 4.03934096065202E-02
 8.83872874606613E-03
 4.38589832159061E-04
 0
 2.54988041607059E-04
 0
 0
 1.65006043957122E-04
 0
 0
 0
 0
 0
 1.97193613401283E-02
 0
 0
 1.28056483197798E-03
 1.68799035442313E-02
 6.95143775165894E-03
 8.2903100046828E-04
 0
 4.19385250979411E-03
 .158766321568738
 2.24975814177572E-03
 0

 4.96689779331838E-04
 7.67195462878323E-04
 7.95626136487047E-04
 1.0016079024768E-03
 2.68055667904685E-04
 7.71491757722488E-04
 4.63098950329799E-04
 1.8360640730264E-04
 1.56816155172156E-03
 0
 .00174955978312
 2.54988041607059E-04
 .004216911584572
 0
 6.93025384618276E-03
 2.32858563246752E-04
 5.19618205237067E-03
 9.48336564851862E-04
 7.08122308056028E-03
 5.3863762941794E-03
 8.21640055837455E-04
 5.39665549498656E-04
 4.44952372443924E-04
 5.12225932790526E-03
 2.28106804652775E-03
 0
 4.1451550023414E-04
 1.43075258239578E-02
 3.49487709148687E-03
 0
 0
 9.57808797298719E-03

 8.27816298884575E-04
 5.75396597158109E-04
 1.43212704567879E-03
 1.0016079024768E-03
 2.14444534323659E-03
 2.62307197626257E-03
 4.63098950329799E-04
 1.10163844381584E-03
 8.55360846394863E-04
 0
 0
 0
 1.50603985163463E-04
 0
 0
 0
 0
 0
 0
 0
 1.64328011167491E-03
 5.39665549498656E-04
 0
 3.20141207994911E-02
 2.73728165582476E-03
 6.31948886513995E-04
 2.07257750118031E-04
 2.32913211088441E-03
 3.35508200782871E-02
 9.83073198570072E-04
 1.8747984514858E-04
 0

 0
 0
 0
 0
 0
 0
 0
 0
 0
 0
 0
 0
 0
 0
 0
 0
 0
 0
 0
 0
 0

 0
 0
 0
 0
 0
 0
 0
 0
 0
 0
 0
 0
 0
 0
 0
 0
 0
 0
 0
 0
 0

 0
 0
 0
 0
 0
 0
 0
 0
 0
 0
 0
 0
 0
 0
 0
 0
 0
 0
 0
 0
 2.73880018612974E-04

 0
 0
 0
 0
 0
 0
 0
 0
 0
 0
 0
 0
 0
 0
 0
 0
 0
 0
 0
 0
 2.73880018612974E-04

 0
 0
 0
 0
 0
 0
 0
 0
 0
 0
 0
 0
 0
 0
 0
 0
 0
 0
 0
 0
 0

 0
 0
 0
 0
 0
 0
 0
 0
 0
 0
 0
 0
 0
 0
 0
 0
 0
 0
 0
 0
 0

 0
 0
 0
 0
 0
 0
 0
 0
 0
 0
 0
 0
 0
 0
 0
 0
 0
 0
 0
 0
 0
 3.59777032999104E-04
 0
 0
 0
 0
 0
 0
 0
 0
 0
 2.71718807744023E-04

 0
 0
 0
 0
 0
 0
 0
 0
 0
 0
 0
 0
 0
 0
 0
 0
 0
 0
 0
 0
 0

 0
 0
 0
 0
 0
 0
 0
 0
 0
 0
 0
 0
 0
 0
 0
 0
 0
 0
 0
 0
 0
 3.59777032999104E-04

 0
 0
 0
 0
 0
 0
 0
 0
 0
 0
 0
 0
 0
 0
 0
 0
 0
 0
 0
 0
 0
 0
 0

 0
 0
 0
 0
 0
 0
 0
 0
 0
 0
 0
 0
 0
 0
 0
 0
 0
 0
 0
 0
 0
 0
 0
 0

 0
 0
 0
 0
 0
 0
 0
 0
 0
 0
 0
 0
 0
 0
 0
 0
 0
 0
 0
 0
 0
 0
 0
 0
 0

 0
 0
 0
 0
 0
 0
 0
 0
 0
 0
 0
 0
 0
 0
 0
 0
 0
 0
 0
 0
 0
 0
 0
 0
 0
 0

 0
 0
 0
 0
 0
 0
 0
 0
 0
 0
 0
 0
 0
 0
 0
 0
 0
 0
 0
 0
 0
 0
 0
 0
 0
 0
 0

 0
 0
 0
 0
 0
 0
 0
 0
 0
 0
 0
 0
 0
 0
 0
 0
 0
 0
 0
 0
 0
 0
 0
 0
 0
 0
 0
 0

 0
 0
 0
 0
 0
 0
 0
 0
 0
 0
 0
 0
 0
 0
 0
 0
 0
 0
 0
 0
 0
 0
 0
 0
 0
 0
 0
 0
 0

 0
 0
 0
 0
 0
 0
 0
 0
 0
 0
 0
 0
 0
 0
 0
 0
 0
 0
 0
 0
 0
 0
 0
 0
 0
 0
 0
 0
 0
 0

 0
 0
 0
 0
 0
 0
 0
 0
 0
 0
 0
 0
 0
 0
 0
 0
 0
 0
 0
 0
 0
 0
 0
 0
 0
 0
 0
 0
 0
 0
 0

 0
 0
 0
 0
 0
 0
 0
 0
 0
 0
 0
 0
 0
 0
 0
 0
 0
 0
 0
 0
 0
 0
 0
 0
 0
 0
 0
 0
 0
 0
 0
 2.71718807744023E-04

 0
 0
 0
 0
 0
 0
 0
 0
 0
 0
 0
 0
 0
 0
 0
 0
 0
 0
 0
 0
 0
 0

 0
 0
 0
 0
 0
 0
 0
 0
 0
 0
 0
 0
 0
 0
 0
 0
 0
 0
 0
 0
 0
 0

 0
 0
 0
 0
 0
 0
 0
 0
 0
 0
 0
 0
 0
 0
 0
 0
 0
 0
 0
 0
 0
 0

 0
 0
 0
 0
 0
 0
 0
 0
 0
 0
 0
 0
 0
 0
 0
 0
 0
 0
 0
 0
 0
 0

 0
 0
 0
 0
 0
 0
 0
 0
 0
 0
 0
 0
 0
 0
 0
 0
 0
 0
 0
 0
 0
 0

 0
 0
 0
 0
 0
 0
 0
 0
 0
 0
 0
 0
 0
 0
 0
 0
 0
 0
 0
 0
 0
 0

 0
 0
 0
 0
 0
 0
 0
 0
 0
 0
 0
 0
 0
 0
 0
 0
 0
 0
 0
 0
 0
 0
 0

 0
 0
 0
 0
 0
 0
 0
 0
 0
 0
 0
 0
 0
 0
 0
 0
 0
 0
 0
 0
 0
 0
 0

 0
 0
 0
 0
 0
 0
 0
 0
 0
 0
 0
 0
 0
 0
 0
 0
 0
 0
 0
 0
 0
 0
 0

 0
 0
 0
 0
 0
 0
 0
 0
 0
 0
 0
 0
 0
 0
 0
 0
 0
 0
 0
 0
 0
 0
 0

 0
 0
 0
 0
 0
 0
 0
 0
 0
 0
 0
 0
 0
 0
 0
 0
 0
 0
 0
 0
 0
 0
 0

 0
 0
 0
 0
 0
 0
 0
 0
 0
 0
 0
 0
 0
 0
 0
 0
 0
 0
 0
 0
 0
 0
 0

 0
 0
 0
 0
 0
 0
 0
 0
 0
 0
 0
 0
 0
 0
 0
 0
 0
 0
 0
 0
 0
 0
 0
 0

 0
 0
 0
 0
 0
 0
 0
 0
 0
 0
 0
 0
 0
 0
 0
 0
 0
 0
 0
 0
 0
 0
 0
 0

 0
 0
 0
 0
 0
 0
 0
 0
 0
 0
 0
 0
 0
 0
 0
 0
 0
 0
 0
 0
 0
 0
 0
 0

 0
 0
 0
 0
 0
 0
 0
 0
 0
 0
 0
 0
 0
 0
 0
 0
 0
 0
 0
 0
 0
 0
 0
 0

 0
 0
 0
 0
 0
 0
 0
 0
 0
 0
 0
 0
 0
 0
 0
 0
 0
 0
 0
 0
 0
 0
 0
 0

 0
 0
 0
 0
 0
 0
 0
 0
 0
 0
 0
 0
 0
 0
 0
 0
 0
 0
 0
 0
 0
 0
 0
 0

 0
 0
 0
 0
 0
 0
 0
 0
 0
 0
 0
 0
 0
 0
 0
 0
 0
 0
 0
 0
 0
 0
 0
 0
 0

 0
 0
 0
 0
 0
 0
 0
 0
 0
 0
 0
 0
 0
 0
 0
 0
 0
 0
 0
 0
 0
 0
 0
 0
 0

 0
 0
 0
 0
 0
 0
 0
 0
 0
 0
 0
 0
 0
 0
 0
 0
 0
 0
 0
 0
 0
 0
 0
 0
 0

 0
 0
 0
 0
 0
 0
 0
 0
 0
 0
 0
 0
 0
 0
 0
 0
 0
 0
 0
 0
 0
 0
 0
 0
 0

 0
 0
 0
 0
 0
 0
 0
 0
 0
 0
 0
 0
 0
 0
 0
 0
 0
 0
 0
 0
 0
 0
 0
 0
 0

 0
 0
 0
 0
 0
 0
 0
 0
 0
 0
 0
 0
 0
 0
 0
 0
 0
 0
 0
 0
 0
 0
 0
 0
 0

 0
 0
 0
 0
 0
 0
 0
 0
 0
 0
 0
 0
 0
 0
 0
 0
 0
 0
 0
 0
 0
 0
 0
 0
 0
 0

 0
 0
 0
 0
 0
 0
 0
 0
 0
 0
 0
 0
 0
 0
 0
 0
 0
 0
 0
 0
 0
 0
 0
 0
 0
 0

 0
 0
 0
 0
 0
 0
 0
 0
 0
 0
 0
 0
 0
 0
 0
 0
 0
 0
 0
 0
 0
 0
 0
 0
 0
 0

 0
 0
 0
 0
 0
 0
 0
 0
 0
 0
 0
 0
 0
 0
 0
 0
 0
 0
 0
 0
 0
 0
 0
 0
 0
 0

 0
 0
 0
 0
 0
 0
 0
 0
 0
 0
 0
 0
 0
 0
 0
 0
 0
 0
 0
 0
 0
 0
 0
 0
 0
 0

 0
 0
 0
 0
 0
 0
 0
 0
 0
 0
 0
 0
 0
 0
 0
 0
 0
 0
 0
 0
 0
 0
 0
 0
 0
 0

 0
 0
 0
 0
 0
 0
 0
 0
 0
 0
 0
 0
 0
 0
 0
 0
 0
 0
 0
 0
 0
 0
 0
 0
 0
 0
 0

 0
 0
 0
 0
 0
 0
 0
 0
 0
 0
 0
 0
 0
 0
 0
 0
 0
 0
 0
 0
 0
 0
 0
 0
 0
 0
 0

 0
 0
 0
 0
 0
 0
 0
 0
 0
 0
 0
 0
 0
 0
 0
 0
 0
 0
 0
 0
 0
 0
 0
 0
 0
 0
 0

 0
 0
 0
 0
 0
 0
 0
 0
 0
 0
 0
 0
 0
 0
 0
 0
 0
 0
 0
 0
 0
 0
 0
 0
 0
 0
 0

 0
 0
 0
 0
 0
 0
 0
 0
 0
 0
 0
 0
 0
 0
 0
 0
 0
 0
 0
 0
 0
 0
 0
 0
 0
 0
 0

 0
 0
 0
 0
 0
 0
 0
 0
 0
 0
 0
 0
 0
 0
 0
 0
 0
 0
 0
 0
 0
 0
 0
 0
 0
 0
 0

 0
 0
 0
 0
 0
 0
 0
 0
 0
 0
 0
 0
 0
 0
 0
 0
 0
 0
 0
 0
 0
 0
 0
 0
 0
 0
 0
 0

 0
 0
 0
 0
 0
 0
 0
 0
 0
 0
 0
 0
 0
 0
 0
 0
 0
 0
 0
 0
 0
 0
 0
 0
 0
 0
 0
 0

 0
 0
 0
 0
 0
 0
 0
 0
 0
 0
 0
 0
 0
 0
 0
 0
 0
 0
 0
 0
 0
 0
 0
 0
 0
 0
 0
 0

 0
 0
 0
 0
 0
 0
 0
 0
 0
 0
 0
 0
 0
 0
 0
 0
 0
 0
 0
 0
 0
 0
 0
 0
 0
 0
 0
 0

 0
 0
 0
 0
 0
 0
 0
 0
 0
 0
 0
 0
 0
 0
 0
 0
 0
 0
 0
 0
 0
 0
 0
 0
 0
 0
 0
 0

 0
 0
 0
 0
 0
 0
 0
 0
 0
 0
 0
 0
 0
 0
 0
 0
 0
 0
 0
 0
 0
 0
 0
 0
 0
 0
 0
 0

 0
 0
 0
 0
 0
 0
 0
 0
 0
 0
 0
 0
 0
 0
 0
 0
 0
 0
 0
 0
 0
 0
 0
 0
 0
 0
 0
 0
 0

 0
 0
 0
 0
 0
 0
 0
 0
 0
 0
 0
 0
 0
 0
 0
 0
 0
 0
 0
 0
 0
 0
 0
 0
 0
 0
 0
 0
 0

 0
 0
 0
 0
 0
 0
 0
 0
 0
 0
 0
 0
 0
 0
 0
 0
 0
 0
 0
 0
 0
 0
 0
 0
 0
 0
 0
 0
 0

 0
 0
 0
 0
 0
 0
 0
 0
 0
 0
 0
 0
 0
 0
 0
 0
 0
 0
 0
 0
 0
 0
 0
 0
 0
 0
 0
 0
 0

 0
 0
 0
 0
 0
 0
 0
 0
 0
 0
 0
 0
 0
 0
 0
 0
 0
 0
 0
 0
 0
 0
 0
 0
 0
 0
 0
 0
 0

 0
 0
 0
 0
 0
 0
 0
 0
 0
 0
 0
 0
 0
 0
 0
 0
 0
 0
 0
 0
 0
 0
 0
 0
 0
 0
 0
 0
 0

 0
 0
 0
 0
 0
 0
 0
 0
 0
 0
 0
 0
 0
 0
 0
 0
 0
 0
 0
 0
 0
 0
 0
 0
 0
 0
 0
 0
 0
 0

 0
 0
 0
 0
 0
 0
 0
 0
 0
 0
 0
 0
 0
 0
 0
 0
 0
 0
 0
 0
 0
 0
 0
 0
 0
 0
 0
 0
 0
 0

 0
 0
 0
 0
 0
 0
 0
 0
 0
 0
 0
 0
 0
 0
 0
 0
 0
 0
 0
 0
 0
 0
 0
 0
 0
 0
 0
 0
 0
 3.27691066189587E-04

 0
 0
 0
 0
 0
 0
 0
 0
 0
 0
 0
 0
 0
 0
 0
 0
 0
 0
 0
 0
 0
 0
 0
 0
 0
 0
 0
 0
 0
 3.27691066189587E-04

 0
 0
 0
 0
 0
 0
 0
 0
 0
 0
 0
 0
 0
 0
 0
 0
 0
 0
 0
 0
 0
 0
 0
 0
 0
 0
 0
 0
 0
 0

 0
 0
 0
 0
 0
 0
 0
 0
 0
 0
 0
 0
 0
 0
 0
 0
 0
 0
 0
 0
 0
 0
 0
 0
 0
 0
 0
 0
 0
 0

 0
 0
 0
 0
 0
 0
 0
 0
 0
 0
 0
 0
 0
 0
 0
 0
 0
 0
 0
 0
 0
 0
 0
 0
 0
 0
 0
 0
 0
 0
 0

 0
 0
 0
 0
 0
 0
 0
 0
 0
 0
 0
 0
 0
 0
 0
 0
 0
 0
 0
 0
 0
 0
 0
 0
 0
 0
 0
 0
 0
 0
 0

 0
 0
 0
 0
 0
 0
 0
 0
 0
 0
 0
 0
 0
 0
 0
 0
 0
 0
 0
 0
 0
 0
 0
 0
 0
 0
 0
 0
 0
 0
 0

 0
 0
 0
 0
 0
 0
 0
 0
 0
 0
 0
 0
 0
 0
 0
 0
 0
 0
 0
 0
 0
 0
 0
 0
 0
 0
 0
 0
 0
 0
 0

 0
 0
 0
 0
 0
 0
 0
 0
 0
 0
 0
 0
 0
 0
 0
 0
 0
 0
 0
 0
 0
 0
 0
 0
 0
 0
 0
 0
 0
 0
 0

 0
 0
 0
 0
 0
 0
 0
 0
 0
 0
 0
 0
 0
 0
 0
 0
 0
 0
 0
 0
 0
 0
 0
 0
 0
 0
 0
 0
 0
 0
 0

 0
 0
 0
 0
 0
 0
 0
 0
 0
 0
 0
 0
 0
 0
 0
 0
 0
 0
 0
 0
 0
 0
 0
 0
 0
 0
 0
 0
 0
 0
 0
 3.88169725348604E-04

 0
 0
 0
 0
 0
 0
 0
 0
 0
 0
 0
 0
 0
 0
 0
 0
 0
 0
 0
 0
 0
 0
 0
 0
 0
 0
 0
 0
 0
 0
 0
 3.88169725348604E-04

 0
 0
 0
 0
 0
 0
 0
 0
 0
 0
 0
 0
 0
 0
 0
 0
 0
 0
 0
 0
 0
 0
 0
 0
 0
 0
 0
 0
 0
 0
 0
 0

 0
 0
 0
 0
 0
 0
 0
 0
 0
 0
 0
 0
 0
 0
 0
 0
 0
 0
 0
 0
 0
 0
 0
 0
 0
 0
 0
 0
 0
 0
 0
 0

 0
 0
 0
 0
 0
 0
 0
 0
 0
 0
 0
 0
 0
 0
 0
 0
 0
 0
 0
 0
 0
 0
 0
 0
 0
 0
 0
 0
 0
 0
 0
 1.35859403872641E-04

 0
 0
 0
 0
 0
 0
 0
 0
 0
 0
 0
 0
 0
 0
 0
 0
 0
 0
 0
 0
 0
 0
 0
 0
 0
 0
 0
 0
 0
 0
 0
 1.35859403872641E-04

 1.57679295025894E-04
 9.13327931998004E-05
 2.27321753282514E-04
 0
 0
 0
 0
 8.74316225250666E-05
 3.39428907298428E-04
 0
 4.16561853123808E-04
 1.21422876955742E-04
 5.02013283877027E-04
 1.59158714944148E-04
 2.35722919938486E-04
 0
 1.83286844880833E-04
 1.50529613468715E-04
 2.408579279101E-04
 1.89995636479026E-04
 1.95628584723553E-04
 0
 2.11882082116154E-04
 1.28056483197798E-03
 4.56213609304126E-04
 7.12196364166276E-04
 9.86941667228721E-05
 1.5844436128404E-04
 0
 1.9661463971349E-03
 8.92761167374191E-05
 0

 1.57679295025894E-04
 9.13327931998004E-05
 2.27321753282514E-04
 0
 0
 0
 0
 8.74316225250666E-05
 3.39428907298428E-04
 0
 4.16561853123808E-04
 1.21422876955742E-04
 5.02013283877027E-04
 1.59158714944148E-04
 2.35722919938486E-04
 0
 1.83286844880833E-04
 1.50529613468715E-04
 2.408579279101E-04
 1.89995636479026E-04
 1.95628584723553E-04
 0
 2.11882082116154E-04
 1.28056483197798E-03
 4.56213609304126E-04
 7.12196364166276E-04
 9.86941667228721E-05
 1.5844436128404E-04
 0
 1.9661463971349E-03
 8.92761167374191E-05
 0

 1.57679295025894E-04
 9.13327931998004E-05
 2.27321753282514E-04
 0
 0
 0
 0
 8.74316225250666E-05
 3.39428907298428E-04
 0
 4.16561853123808E-04
 1.21422876955742E-04
 5.02013283877027E-04
 1.59158714944148E-04
 2.35722919938486E-04
 0
 1.83286844880833E-04
 1.50529613468715E-04
 2.408579279101E-04
 1.89995636479026E-04
 1.95628584723553E-04
 0
 2.11882082116154E-04
 0
 0
 5.01546735328832E-04
 9.86941667228721E-05
 1.5844436128404E-04
 0
 0
 8.92761167374191E-05
 0

 0
 0
 0
 0
 0
 0
 0
 0
 0
 0
 0
 0
 0
 0
 0
 0
 0
 0
 0
 0
 0

 0
 0
 0
 0
 0
 0
 0
 0
 0
 0
 0
 0
 0
 0
 0
 0
 0
 0
 0
 0
 0
 0

 0
 0
 0
 0
 0
 0
 0
 0
 0
 0
 0
 0
 0
 0
 0
 0
 0
 0
 0
 0
 0
 0
 0

 0
 0
 0
 0
 0
 0
 0
 0
 0
 0
 0
 0
 0
 0
 0
 0
 0
 0
 0
 0
 0
 0
 0
 1.28056483197798E-03

 0
 0
 0
 0
 0
 0
 0
 0
 0
 0
 0
 0
 0
 0
 0
 0
 0
 0
 0
 0
 0
 0
 0
 0
 4.56213609304126E-04

 0
 0
 0
 0
 0
 0
 0
 0
 0
 0
 0
 0
 0
 0
 0
 0
 0
 0
 0
 0
 0
 0
 0
 0
 0
 2.10649628837444E-04

 0
 0
 0
 0
 0
 0
 0
 0
 0
 0
 0
 0
 0
 0
 0
 0
 0
 0
 0
 0
 0
 0
 0
 0
 0
 0
 0

 0
 0
 0
 0
 0
 0
 0
 0
 0
 0
 0
 0
 0
 0
 0
 0
 0
 0
 0
 0
 0
 0
 0
 0
 0
 0
 0
 0

 0
 0
 0
 0
 0
 0
 0
 0
 0
 0
 0
 0
 0
 0
 0
 0
 0
 0
 0
 0
 0
 0
 0
 0
 0
 0
 0
 0
 0

 0
 0
 0
 0
 0
 0
 0
 0
 0
 0
 0
 0
 0
 0
 0
 0
 0
 0
 0
 0
 0
 0
 0
 0
 0
 0
 0
 0
 0
 1.9661463971349E-03

 0
 0
 0
 0
 0
 0
 0
 0
 0
 0
 0
 0
 0
 0
 0
 0
 0
 0
 0
 0
 0
 0
 0
 0
 0
 0
 0
 0
 0
 0
 0

 0
 0
 0
 0
 0
 0
 0
 0
 0
 0
 0
 0
 0
 0
 0
 0
 0
 0
 0
 0
 0
 0
 0
 0
 0
 0
 0
 0
 0
 0
 0
 0

 1.83959177530209E-04
 0
 8.84029040544082E-05
 3.25308549523086E-04
 0
 0
 0
 1.88314263899833E-04
 0
 4.49835725290602E-04
 3.36453804445783E-04
 6.53815491297993E-04
 1.62188907098987E-03
 1.71401693016775E-04
 3.30012087913265E-03
 7.16487886913084E-04
 2.96078749423048E-03
 8.10544072521175E-04
 1.94539095619803E-03
 1.73919082623227E-03
 6.32030812183034E-04
 2.76751563845465E-04
 1.82544563053067E-03
 0
 3.50933545619107E-04
 1.44213976666078E-03
 5.20801525935695E-04
 0
 0
 0
 3.55730988230639E-04
 0

 1.83959177530209E-04
 0
 8.84029040544082E-05
 3.25308549523086E-04
 0
 0
 0
 1.88314263899833E-04
 0
 4.49835725290602E-04
 3.36453804445783E-04
 6.53815491297993E-04
 1.62188907098987E-03
 1.71401693016775E-04
 3.30012087913265E-03
 7.16487886913084E-04
 2.96078749423048E-03
 8.10544072521175E-04
 1.94539095619803E-03
 1.73919082623227E-03
 6.32030812183034E-04
 2.76751563845465E-04
 1.82544563053067E-03
 0
 3.50933545619107E-04
 1.44213976666078E-03
 5.20801525935695E-04
 0
 0
 0
 3.55730988230639E-04
 0

 0
 0
 0
 1.02729015638985E-04
 0
 0
 0
 1.88314263899833E-04
 0
 4.49835725290602E-04
 3.36453804445783E-04
 6.53815491297993E-04
 1.62188907098987E-03
 1.71401693016775E-04
 3.30012087913265E-03
 7.16487886913084E-04
 2.96078749423048E-03
 8.10544072521175E-04
 1.94539095619803E-03
 1.73919082623227E-03
 6.32030812183034E-04
 2.76751563845465E-04
 1.82544563053067E-03
 0
 3.50933545619107E-04
 1.44213976666078E-03
 5.20801525935695E-04
 0
 0
 0
 3.55730988230639E-04
 0

 0
 0
 0
 1.02729015638985E-04
 0
 0
 0
 1.88314263899833E-04
 0
 4.49835725290602E-04
 3.36453804445783E-04
 6.53815491297993E-04
 1.62188907098987E-03
 1.71401693016775E-04
 3.30012087913265E-03
 7.16487886913084E-04
 2.96078749423048E-03
 8.10544072521175E-04
 1.94539095619803E-03
 1.73919082623227E-03
 6.32030812183034E-04
 2.76751563845465E-04
 1.82544563053067E-03
 0
 0
 0
 0
 0
 0
 0
 0
 0

 0
 0
 0
 0
 0
 0
 0
 0
 0
 0
 0
 0
 0
 0
 0
 0
 0
 0
 0
 0
 0
 0
 0
 0
 0
 1.08025450685869E-04
 1.06286025701555E-04
 0
 0
 0
 9.61435103326052E-05
 0

 0
 0
 0
 0
 0
 0
 0
 0
 0
 0
 0
 0
 0
 0
 0
 0
 0
 0
 0
 0
 0
 0
 0
 0
 3.50933545619107E-04
 0
 0
 0
 0
 0
 7.21076327494539E-05
 0

 0
 0
 0
 0
 0
 0
 0
 0
 0
 0
 0
 0
 0
 0
 0
 0
 0
 0
 0
 0
 0
 0
 0
 0
 0
 1.12346468713747E-03
 0
 0
 0
 0
 0
 0

 0
 0
 0
 0
 0
 0
 0
 0
 0
 0
 0
 0
 0
 0
 0
 0
 0
 0
 0
 0
 0
 0
 0
 0
 0
 2.10649628837444E-04
 4.1451550023414E-04
 0
 0
 0
 1.8747984514858E-04
 0

 1.83959177530209E-04
 0
 8.84029040544082E-05
 2.22579533884101E-04
 0
 0
 0
 0
 0
 0
 0
 0
 0
 0
 0
 0
 0
 0
 0
 0

 1.83959177530209E-04

 0
 0

 0
 0
 8.84029040544082E-05

 0
 0
 0
 2.22579533884101E-04

 0
 0
 0
 0
 0

 0
 0
 0
 0
 0
 0

 0
 0
 0
 0
 0
 0
 0

 0
 0
 0
 0
 0
 0
 0
 0

 0
 0
 0
 0
 0
 0
 0
 0
 0

 0
 0
 0
 0
 0
 0
 0
 0
 0
 0

 0
 0
 0
 0
 0
 0
 0
 0
 0
 0
 0

 0
 0
 0
 0
 0
 0
 0
 0
 0
 0
 0
 0

 0
 0
 0
 0
 0
 0
 0
 0
 0
 0
 0
 0
 0

 0
 0
 0
 0
 0
 0
 0
 0
 0
 0
 0
 0
 0
 0

 0
 0
 0
 0
 0
 0
 0
 0
 0
 0
 0
 0
 0
 0
 0

 0
 0
 0
 0
 0
 0
 0
 0
 0
 0
 0
 0
 0
 0
 0
 0

 0
 0
 0
 0
 0
 0
 0
 0
 0
 0
 0
 0
 0
 0
 0
 0
 0

 0
 0
 0
 0
 0
 0
 0
 0
 0
 0
 0
 0
 0
 0
 0
 0
 0
 0

 0
 0
 0
 0
 0
 0
 0
 0
 0
 0
 0
 0
 0
 0
 0
 0
 0
 0
 0

 0
 0
 0
 0
 0
 0
 0
 0
 0
 0
 0
 0
 0
 0
 0
 0
 0
 0
 0
 0

 5.10893902511971E-03
 5.02073351991446E-03
 7.65155348283328E-03
 1.44143544949758E-02
 8.66903436628782E-03
 1.64147182494472E-03
 1.52724121917331E-02
 6.25043088688007E-03
 9.52423070098443E-03
 4.10594736488718E-03
 8.65473722499466E-03
 4.23171643516844E-03
 6.60094062631455E-03
 9.95588557307854E-04
 4.91507364977501E-03
 3.76537251208496E-03
 6.1420591635497E-03
 1.21064242321426E-03
 4.41231225382666E-03
 4.07480003342364E-03
 3.06211770981649E-03
 2.29644914680279E-04
 0
 2.77909814599682E-02
 3.35850869828387E-02
 2.16577312284624E-03
 7.40836213184748E-03
 4.16270419816372E-02
 1.24923266249046E-02
 2.94921959570946E-02
 1.60586717052456E-03
 3.04914379315282E-04

 5.10893902511971E-03
 5.02073351991446E-03
 7.65155348283328E-03
 1.44143544949758E-02
 8.66903436628782E-03
 1.64147182494472E-03
 1.52724121917331E-02
 6.25043088688007E-03
 9.52423070098443E-03
 4.10594736488718E-03
 8.65473722499466E-03
 4.23171643516844E-03
 6.60094062631455E-03
 9.95588557307854E-04
 4.91507364977501E-03
 3.76537251208496E-03
 6.1420591635497E-03
 1.21064242321426E-03
 4.41231225382666E-03
 4.07480003342364E-03
 3.06211770981649E-03
 2.29644914680279E-04
 0
 2.77909814599682E-02
 3.35850869828387E-02
 2.16577312284624E-03
 7.40836213184748E-03
 4.16270419816372E-02
 1.24923266249046E-02
 2.94921959570946E-02
 1.60586717052456E-03
 3.04914379315282E-04

 1.0681500630804E-04
 1.23741203690052E-04
 0
 7.75438376113204E-04
 0
 0
 0
 0
 0
 0
 0
 0
 0
 0
 0
 0
 0
 0
 0
 0
 2.65045179302878E-04
 0
 0
 0
 0
 1.35902986346738E-03
 0
 0
 0
 0
 9.67637910444284E-04
 1.31476842457395E-04

 0
 1.23741203690052E-04
 0
 2.58479458704117E-04
 0
 0
 0
 0
 0
 0
 0
 0
 0
 0
 0
 0
 0
 0
 0
 0

 1.0681500630804E-04
 0
 0
 5.16958917409087E-04
 0
 0
 0
 0
 0
 0
 0
 0
 0
 0
 0
 0
 0
 0
 0
 0

 0
 0
 0
 0
 0
 0
 0
 0
 0
 0
 0
 0
 0
 0
 0
 0
 0
 0
 0
 0
 2.65045179302878E-04
 0
 0
 0
 0
 1.35902986346738E-03
 0
 0
 0
 0
 8.46683171638749E-04
 0

 0
 0
 0
 0
 0
 0
 0
 0
 0
 0
 0
 0
 0
 0
 0
 0
 0
 0
 0
 0
 0
 0
 0
 0
 0
 0
 0
 0
 0
 0
 1.20954738805536E-04
 1.31476842457395E-04

 1.40904901938033E-04
 0
 0
 0
 0
 0
 0
 0
 0
 0
 0
 0
 0
 0
 0
 0
 0
 0
 0
 0
 2.4474384641998E-03

 1.40904901938033E-04
 0
 0
 0
 0
 0
 0
 0
 0
 0
 0
 0
 0
 0
 0
 0
 0
 0
 0
 0
 2.4474384641998E-03

 4.86121911687364E-03
 4.81537577762033E-03
 7.44841489479359E-03
 1.27012406356911E-02
 7.81353755382559E-03
 1.11620084096171E-03
 1.47797537339362E-02
 1.56260772172202E-04
 6.06638898152385E-05
 0
 0
 0
 0
 0
 0
 0
 0
 0
 0
 0
 3.49634066313811E-04
 2.29644914680279E-04
 0
 5.44921205097012E-03
 1.16480070460931E-03
 0
 0
 1.41588578168716E-04
 5.94872696423996E-03
 1.04582255167168E-03
 0
 0

 0
 0
 6.77128626799722E-05
 8.52432257429877E-05
 0
 6.56588729978753E-05
 9.85316915596943E-05
 0
 0
 0
 0
 0
 0
 0
 0
 0
 0
 0
 0
 0
 0
 0
 0
 0
 1.94133450767713E-04
 0
 0
 0
 0
 1.04582255167168E-03
 0
 0

 3.66352745039955E-03
 0
 0
 0
 0
 0
 0
 0
 0
 0
 0
 0
 0
 0
 0
 0
 0
 0
 0
 0
 3.49634066313811E-04

 1.19769166647409E-03
 0
 0
 0
 0
 0
 0
 0
 0
 0
 0
 0
 0
 0
 0
 0
 0
 0
 0
 0
 0

 0
 3.34627808274964E-03
 0
 0
 0
 0
 0
 0
 0
 0
 0
 0
 0
 0
 0
 0
 0
 0
 0
 0
 0
 2.29644914680279E-04

 0
 1.46909769487069E-03
 0
 0
 0
 0
 0
 0
 0
 0
 0
 0
 0
 0
 0
 0
 0
 0
 0
 0
 0
 0

 0
 0
 5.41702901439778E-03
 0
 0
 0
 0
 0
 0
 0
 0
 0
 0
 0
 0
 0
 0
 0
 0
 0
 0
 0
 0

 0
 0
 1.96367301771584E-03
 0
 0
 0
 0
 0
 0
 0
 0
 0
 0
 0
 0
 0
 0
 0
 0
 0
 0
 0
 0

 0
 0
 0
 1.13373490238047E-02
 0
 0
 0
 0
 0
 0
 0
 0
 0
 0
 0
 0
 0
 0
 0
 0
 0
 0
 0
 0

 0
 0
 0
 1.27864838614341E-03
 0
 0
 0
 0
 0
 0
 0
 0
 0
 0
 0
 0
 0
 0
 0
 0
 0
 0
 0
 5.44921205097012E-03

 0
 0
 0
 0
 7.12914010385505E-03
 0
 0
 0
 0
 0
 0
 0
 0
 0
 0
 0
 0
 0
 0
 0
 0
 0
 0
 0
 0

 0
 0
 0
 0
 6.84397449970537E-04
 0
 0
 0
 0
 0
 0
 0
 0
 0
 0
 0
 0
 0
 0
 0
 0
 0
 0
 0
 9.70667253841597E-04

 0
 0
 0
 0
 0
 3.28294364988293E-04
 0
 0
 0
 0
 0
 0
 0
 0
 0
 0
 0
 0
 0
 0
 0
 0
 0
 0
 0
 0

 0
 0
 0
 0
 0
 7.22247602975545E-04
 0
 0
 0
 0
 0
 0
 0
 0
 0
 0
 0
 0
 0
 0
 0
 0
 0
 0
 0
 0

 0
 0
 0
 0
 0
 0
 .012809119902744
 0
 0
 0
 0
 0
 0
 0
 0
 0
 0
 0
 0
 0
 0
 0
 0
 0
 0
 0
 0

 0
 0
 0
 0
 0
 0
 1.87210213963256E-03
 0
 0
 0
 0
 0
 0
 0
 0
 0
 0
 0
 0
 0
 0
 0
 0
 0
 0
 0
 0

 0
 0
 0
 0
 0
 0
 0
 1.56260772172202E-04
 0
 0
 0
 0
 0
 0
 0
 0
 0
 0
 0
 0
 0
 0
 0
 0
 0
 0
 0
 1.41588578168716E-04

 0
 0
 0
 0
 0
 0
 0
 0
 0
 0
 0
 0
 0
 0
 0
 0
 0
 0
 0
 0
 0
 0
 0
 0
 0
 0
 0
 0

 0
 0
 0
 0
 0
 0
 0
 0
 6.06638898152385E-05
 0
 0
 0
 0
 0
 0
 0
 0
 0
 0
 0
 0
 0
 0
 0
 0
 0
 0
 0
 0

 0
 0
 0
 0
 0
 0
 0
 0
 0
 0
 0
 0
 0
 0
 0
 0
 0
 0
 0
 0
 0
 0
 0
 0
 0
 0
 0
 0
 5.94872696423996E-03

 0
 0
 0
 0
 0
 0
 0
 0
 0
 0
 0
 0
 0
 0
 0
 0
 0
 0
 0
 0
 0
 0
 0
 0
 0
 0
 0
 0
 0
 0

 0
 0
 0
 0
 0
 0
 0
 0
 0
 0
 0
 0
 0
 0
 0
 0
 0
 0
 0
 0
 0
 0
 0
 0
 0
 0
 0
 0
 0
 0

 0
 0
 0
 0
 0
 0
 0
 0
 0
 0
 0
 0
 0
 0
 0
 0
 0
 0
 0
 0
 0
 0
 0
 0
 0
 0
 0
 0
 0
 0
 0

 0
 0
 0
 0
 0
 0
 0
 0
 0
 0
 0
 0
 0
 0
 0
 0
 0
 0
 0
 0
 0
 0
 0
 0
 0
 0
 0
 0
 0
 0
 0

 0
 0
 0
 0
 0
 0
 0
 0
 0
 0
 0
 0
 0
 0
 0
 0
 0
 0
 0
 0
 0
 0
 0
 0
 0
 0
 0
 0
 0
 0
 0
 0

 0
 0
 0
 0
 0
 0
 0
 0
 0
 0
 0
 0
 0
 0
 0
 0
 0
 0
 0
 0
 0
 0
 0
 0
 0
 0
 0
 0
 0
 0
 0
 0

 0
 0
 0
 0
 0
 0
 0
 0
 0
 0
 0
 0
 0

 0
 0
 0
 0
 0
 0
 0
 0
 0
 0
 0
 0
 0

 0
 0
 0
 0
 0
 0
 0
 0
 0
 0
 0
 0
 0
 0

 0
 0
 0
 0
 0
 0
 0
 0
 0
 0
 0
 0
 0
 0

 0
 0
 0
 0
 0
 0
 0
 0
 0
 0
 0
 0
 0
 0
 0

 0
 0
 0
 0
 0
 0
 0
 0
 0
 0
 0
 0
 0
 0
 0

 0
 0
 0
 0
 0
 0
 0
 0
 0
 0
 0
 0
 0
 0
 0
 0

 0
 0
 0
 0
 0
 0
 0
 0
 0
 0
 0
 0
 0
 0
 0
 0

 0
 0
 0
 0
 0
 0
 0
 0
 0
 0
 0
 0
 0
 0
 0
 0
 0

 0
 0
 0
 0
 0
 0
 0
 0
 0
 0
 0
 0
 0
 0
 0
 0
 0

 0
 0
 0
 0
 0
 0
 0
 0
 0
 0
 0
 0
 0
 0
 0
 0
 0
 0

 0
 0
 0
 0
 0
 0
 0
 0
 0
 0
 0
 0
 0
 0
 0
 0
 0
 0

 0
 0
 0
 0
 0
 0
 0
 0
 0
 0
 0
 0
 0
 0
 0
 0
 0
 0
 0

 0
 0
 0
 0
 0
 0
 0
 0
 0
 0
 0
 0
 0
 0
 0
 0
 0
 0
 0

 0
 0
 0
 0
 0
 0
 0
 0
 0
 0
 0
 0
 0
 0
 0
 0
 0
 0
 0
 0

 0
 0
 0
 0
 0
 0
 0
 0
 0
 0
 0
 0
 0
 0
 0
 0
 0
 0
 0
 0

 0
 8.1616538604077E-05
 0
 0
 0
 0
 0
 0
 0
 0
 0
 0
 0
 0
 0
 0
 0
 0
 0
 0
 0
 0

 0
 8.1616538604077E-05
 0
 0
 0
 0
 0
 0
 0
 0
 0
 0
 0
 0
 0
 0
 0
 0
 0
 0
 0
 0

 0
 0
 2.03138588039693E-04
 0
 0
 0
 0
 0
 0
 0
 0
 0
 0
 0
 0
 0
 0
 0
 0
 0
 0
 0
 0

 0
 0
 2.03138588039693E-04
 0
 0
 0
 0
 0
 0
 0
 0
 0
 0
 0
 0
 0
 0
 0
 0
 0
 0
 0
 0

 0
 0
 0
 9.37675483171458E-04
 0
 0
 0
 0
 0
 0
 0
 0
 0
 0
 0
 0
 0
 0
 0
 0
 0
 0
 0
 .022341769408998

 0
 0
 0
 9.37675483171458E-04
 0
 0
 0
 0
 0
 0
 0
 0
 0
 0
 0
 0
 0
 0
 0
 0
 0
 0
 0
 .022341769408998

 0
 0
 0
 0
 8.5549681246223E-04
 0
 0
 0
 0
 0
 0
 0
 0
 0
 0
 0
 0
 0
 0
 0
 0
 0
 0
 0
 3.24202862782293E-02

 0
 0
 0
 0
 8.5549681246223E-04
 0
 0
 0
 0
 0
 0
 0
 0
 0
 0
 0
 0
 0
 0
 0
 0
 0
 0
 0
 3.24202862782293E-02

 0
 0
 0
 0
 0
 5.25270983983002E-04
 0
 0
 0
 0
 0
 0
 0
 0
 0
 0
 0
 0
 0
 0
 0
 0
 0
 0
 0
 8.06743259378861E-04

 0
 0
 0
 0
 0
 5.25270983983002E-04
 0
 0
 0
 0
 0
 0
 0
 0
 0
 0
 0
 0
 0
 0
 0
 0
 0
 0
 0
 8.06743259378861E-04

 0
 0
 0
 0
 0
 0
 4.92658457796845E-04
 0
 0
 0
 0
 0
 0
 0
 0
 0
 0
 0
 0
 0
 0
 0
 0
 0
 0
 0
 7.40836213184748E-03

 0
 0
 0
 0
 0
 0
 4.92658457796845E-04
 0
 0
 0
 0
 0
 0
 0
 0
 0
 0
 0
 0
 0
 0
 0
 0
 0
 0
 0
 7.40836213184748E-03

 0
 0
 0
 0
 0
 0
 0
 6.09417011470787E-03
 0
 0
 0
 0
 0
 0
 0
 0
 0
 0
 0
 0
 0
 0
 0
 0
 0
 0
 0
 4.14854534034685E-02

 0
 0
 0
 0
 0
 0
 0
 6.09417011470787E-03
 0
 0
 0
 0
 0
 0
 0
 0
 0
 0
 0
 0
 0
 0
 0
 0
 0
 0
 0
 4.14854534034685E-02

 0
 0
 0
 0
 0
 0
 0
 0
 9.46356681116919E-03
 0
 0
 0
 0
 0
 0
 0
 0
 0
 0
 0
 0
 0
 0
 0
 0
 0
 0
 0
 6.54359966066466E-03

 0
 0
 0
 0
 0
 0
 0
 0
 9.46356681116919E-03
 0
 0
 0
 0
 0
 0
 0
 0
 0
 0
 0
 0
 0
 0
 0
 0
 0
 0
 0
 6.54359966066466E-03

 0
 0
 0
 0
 0
 0
 0
 0
 0
 4.10594736488718E-03
 0
 0
 0
 0
 0
 0
 0
 0
 0
 0
 0
 0
 0
 0
 0
 0
 0
 0
 0
 .028446373405423

 0
 0
 0
 0
 0
 0
 0
 0
 0
 4.10594736488718E-03
 0
 0
 0
 0
 0
 0
 0
 0
 0
 0
 0
 0
 0
 0
 0
 0
 0
 0
 0
 .028446373405423

 0
 0
 0
 0
 0
 0
 0
 0
 0
 0
 8.65473722499466E-03
 0
 0
 0
 0
 0
 0
 0
 0
 0
 0
 0
 0
 0
 0
 0
 0
 0
 0
 0
 6.38229260080273E-04

 0
 0
 0
 0
 0
 0
 0
 0
 0
 0
 8.65473722499466E-03
 0
 0
 0
 0
 0
 0
 0
 0
 0
 0
 0
 0
 0
 0
 0
 0
 0
 0
 0
 6.38229260080273E-04

 0
 0
 0
 0
 0
 0
 0
 0
 0
 0
 0
 4.23171643516844E-03
 0
 0
 0
 0
 0
 0
 0
 0
 0
 0
 0
 0
 0
 0
 0
 0
 0
 0
 0
 1.73437536857887E-04

 0
 0
 0
 0
 0
 0
 0
 0
 0
 0
 0
 4.23171643516844E-03
 0
 0
 0
 0
 0
 0
 0
 0
 0
 0
 0
 0
 0
 0
 0
 0
 0
 0
 0
 1.73437536857887E-04

 0
 0
 0
 0
 0
 0
 0
 0
 0
 0
 0
 0
 6.60094062631455E-03

 0
 0
 0
 0
 0
 0
 0
 0
 0
 0
 0
 0
 6.60094062631455E-03

 0
 0
 0
 0
 0
 0
 0
 0
 0
 0
 0
 0
 0
 9.95588557307854E-04

 0
 0
 0
 0
 0
 0
 0
 0
 0
 0
 0
 0
 0
 9.95588557307854E-04

 0
 0
 0
 0
 0
 0
 0
 0
 0
 0
 0
 0
 0
 0
 4.91507364977501E-03

 0
 0
 0
 0
 0
 0
 0
 0
 0
 0
 0
 0
 0
 0
 4.91507364977501E-03

 0
 0
 0
 0
 0
 0
 0
 0
 0
 0
 0
 0
 0
 0
 0
 3.76537251208496E-03

 0
 0
 0
 0
 0
 0
 0
 0
 0
 0
 0
 0
 0
 0
 0
 3.76537251208496E-03

 0
 0
 0
 0
 0
 0
 0
 0
 0
 0
 0
 0
 0
 0
 0
 0
 6.1420591635497E-03

 0
 0
 0
 0
 0
 0
 0
 0
 0
 0
 0
 0
 0
 0
 0
 0
 6.1420591635497E-03

 0
 0
 0
 0
 0
 0
 0
 0
 0
 0
 0
 0
 0
 0
 0
 0
 0
 1.21064242321426E-03

 0
 0
 0
 0
 0
 0
 0
 0
 0
 0
 0
 0
 0
 0
 0
 0
 0
 1.21064242321426E-03

 0
 0
 0
 0
 0
 0
 0
 0
 0
 0
 0
 0
 0
 0
 0
 0
 0
 0
 4.41231225382666E-03

 0
 0
 0
 0
 0
 0
 0
 0
 0
 0
 0
 0
 0
 0
 0
 0
 0
 0
 4.41231225382666E-03

 0
 0
 0
 0
 0
 0
 0
 0
 0
 0
 0
 0
 0
 0
 0
 0
 0
 0
 0
 4.07480003342364E-03

 0
 0
 0
 0
 0
 0
 0
 0
 0
 0
 0
 0
 0
 0
 0
 0
 0
 0
 0
 4.07480003342364E-03

 2.77430327194222E-03
 1.45145087571369E-03
 3.52655909146348E-03
 3.14017612669224E-03
 6.08558813620537E-03
 5.00427086091088E-03
 1.37678066314492E-03
 3.96986826599975E-03
 7.93713217825227E-03
 2.60783143445772E-03
 1.1821349885942E-03
 8.26988243049011E-04
 1.70955875050015E-03
 1.8066664939606E-04
 1.78384912385783E-03
 3.77608480940679E-04
 1.87249803689171E-03
 0
 1.3670314827321E-03
 7.54847528714503E-04
 5.32955711894565E-03
 0
 0
 .004153183238843
 3.45242731365747E-03
 1.59410529931686E-02
 1.68046824419298E-03
 3.59711522915605E-03
 2.26694730258253E-03
 7.43947285403812E-03
 8.61393883115489E-03
 3.30468820229728E-04

 2.77430327194222E-03
 1.45145087571369E-03
 3.52655909146348E-03
 3.14017612669224E-03
 6.08558813620537E-03
 5.00427086091088E-03
 1.37678066314492E-03
 3.96986826599975E-03
 7.93713217825227E-03
 2.60783143445772E-03
 1.1821349885942E-03
 8.26988243049011E-04
 1.70955875050015E-03
 1.8066664939606E-04
 1.78384912385783E-03
 3.77608480940679E-04
 1.87249803689171E-03
 0
 1.3670314827321E-03
 7.54847528714503E-04
 5.32955711894565E-03
 0
 0
 .004153183238843
 3.45242731365747E-03
 1.59410529931686E-02
 1.68046824419298E-03
 3.59711522915605E-03
 2.26694730258253E-03
 7.43947285403812E-03
 8.61393883115489E-03
 3.30468820229728E-04

 1.34240480900497E-03
 1.14042568806066E-03
 2.4083818185605E-03
 2.27392064346656E-03
 2.3183192899879E-03
 2.58553994480409E-03
 1.2516187846772E-03
 1.98493413299824E-03
 2.69708374990007E-03
 4.74151169900905E-04
 1.18213498859459E-04
 4.13494121524506E-04
 4.07037797737746E-04
 0
 8.02732105735876E-04
 1.25869493646893E-04
 1.0402766871632E-03
 0
 4.10109444820126E-04
 1.07835361245031E-04
 1.77651903964855E-03
 0
 0
 6.92197206474583E-04
 9.86407803900814E-04
 1.13864664236456E-04
 5.60156081398006E-04
 2.33812489895314E-03
 1.13347365129126E-03
 2.12556367258536E-03
 5.06702284185352E-04
 1.1015627341025E-04

 1.34240480900497E-03
 1.14042568806066E-03
 2.4083818185605E-03
 2.27392064346656E-03
 2.3183192899879E-03
 2.58553994480409E-03
 1.2516187846772E-03
 1.98493413299824E-03
 2.69708374990007E-03
 4.74151169900905E-04
 1.18213498859459E-04
 4.13494121524506E-04
 4.07037797737746E-04
 0
 8.02732105735876E-04
 1.25869493646893E-04
 1.0402766871632E-03
 0
 4.10109444820126E-04
 1.07835361245031E-04
 1.77651903964855E-03
 0
 0
 6.92197206474583E-04
 9.86407803900814E-04
 1.13864664236456E-04
 5.60156081398006E-04
 2.33812489895314E-03
 1.13347365129126E-03
 2.12556367258536E-03
 5.06702284185352E-04
 1.1015627341025E-04

 6.26455577534864E-04
 0
 0
 0
 0
 0
 0
 0
 0
 0
 0
 0
 0
 0
 0
 0
 0
 0
 0
 0
 2.44271367951477E-03

 6.26455577534864E-04
 0
 0
 0
 0
 0
 0
 0
 0
 0
 0
 0
 0
 0
 0
 0
 0
 0
 0
 0
 2.44271367951477E-03

 8.0544288540239E-04
 0
 0
 0
 0
 0
 0
 0
 0
 0
 0
 0
 0
 0
 0
 0
 0
 0
 0
 0
 1.11032439978233E-03

 8.0544288540239E-04
 0
 0
 0
 0
 0
 0
 0
 0
 0
 0
 0
 0
 0
 0
 0
 0
 0
 0
 0
 1.11032439978233E-03

 0
 2.07350125101907E-04
 0
 0
 0
 0
 0
 0
 0
 0
 0
 0
 0
 0
 0
 0
 0
 0
 0
 0
 0
 0

 0
 2.07350125101907E-04
 0
 0
 0
 0
 0
 0
 0
 0
 0
 0
 0
 0
 0
 0
 0
 0
 0
 0
 0
 0

 0
 1.03675062551125E-04
 0
 0
 0
 0
 0
 0
 0
 0
 0
 0
 0
 0
 0
 0
 0
 0
 0
 0
 0
 0

 0
 1.03675062551125E-04
 0
 0
 0
 0
 0
 0
 0
 0
 0
 0
 0
 0
 0
 0
 0
 0
 0
 0
 0
 0

 0
 0
 5.16081818263572E-04
 0
 0
 0
 0
 0
 0
 0
 0
 0
 0
 0
 0
 0
 0
 0
 0
 0
 0
 0
 0

 0
 0
 5.16081818263572E-04
 0
 0
 0
 0
 0
 0
 0
 0
 0
 0
 0
 0
 0
 0
 0
 0
 0
 0
 0
 0

 0
 0
 6.02095454639414E-04
 0
 0
 0
 0
 0
 0
 0
 0
 0
 0
 0
 0
 0
 0
 0
 0
 0
 0
 0
 0

 0
 0
 6.02095454639414E-04
 0
 0
 0
 0
 0
 0
 0
 0
 0
 0
 0
 0
 0
 0
 0
 0
 0
 0
 0
 0

 0
 0
 0
 6.49691612419528E-04
 0
 0
 0
 0
 0
 0
 0
 0
 0
 0
 0
 0
 0
 0
 0
 0
 0
 0
 0
 2.07659161942105E-03

 0
 0
 0
 6.49691612419528E-04
 0
 0
 0
 0
 0
 0
 0
 0
 0
 0
 0
 0
 0
 0
 0
 0
 0
 0
 0
 2.07659161942105E-03

 0
 0
 0
 2.16563870806152E-04
 0
 0
 0
 0
 0
 0
 0
 0
 0
 0
 0
 0
 0
 0
 0
 0
 0
 0
 0
 1.38439441294737E-03

 0
 0
 0
 2.16563870806152E-04
 0
 0
 0
 0
 0
 0
 0
 0
 0
 0
 0
 0
 0
 0
 0
 0
 0
 0
 0
 1.38439441294737E-03

 0
 0
 0
 0
 2.97034659028451E-03
 0
 0
 0
 0
 0
 0
 0
 0
 0
 0
 0
 0
 0
 0
 0
 0
 0
 0
 0
 1.72621365683027E-03

 0
 0
 0
 0
 2.97034659028451E-03
 0
 0
 0
 0
 0
 0
 0
 0
 0
 0
 0
 0
 0
 0
 0
 0
 0
 0
 0
 1.72621365683027E-03

 0
 0
 0
 0
 7.96922255932966E-04
 0
 0
 0
 0
 0
 0
 0
 0
 0
 0
 0
 0
 0
 0
 0
 0
 0
 0
 0
 7.3980585292638E-04

 0
 0
 0
 0
 7.96922255932966E-04
 0
 0
 0
 0
 0
 0
 0
 0
 0
 0
 0
 0
 0
 0
 0
 0
 0
 0
 0
 7.3980585292638E-04

 0
 0
 0
 0
 0
 1.50128125827299E-03
 0
 0
 0
 0
 0
 0
 0
 0
 0
 0
 0
 0
 0
 0
 0
 0
 0
 0
 0
 1.44608123580893E-02

 0
 0
 0
 0
 0
 1.50128125827299E-03
 0
 0
 0
 0
 0
 0
 0
 0
 0
 0
 0
 0
 0
 0
 0
 0
 0
 0
 0
 1.44608123580893E-02

 0
 0
 0
 0
 0
 9.174496578338E-04
 0
 0
 0
 0
 0
 0
 0
 0
 0
 0
 0
 0
 0
 0
 0
 0
 0
 0
 0
 1.36637597084287E-03

 0
 0
 0
 0
 0
 9.174496578338E-04
 0
 0
 0
 0
 0
 0
 0
 0
 0
 0
 0
 0
 0
 0
 0
 0
 0
 0
 0
 1.36637597084287E-03

 0
 0
 0
 0
 0
 0
 1.2516187846772E-04
 0
 0
 0
 0
 0
 0
 0
 0
 0
 0
 0
 0
 0
 0
 0
 0
 0
 0
 0
 2.24062432558995E-04

 0
 0
 0
 0
 0
 0
 1.2516187846772E-04
 0
 0
 0
 0
 0
 0
 0
 0
 0
 0
 0
 0
 0
 0
 0
 0
 0
 0
 0
 2.24062432558995E-04

 0
 0
 0
 0
 0
 0
 0
 0
 0
 0
 0
 0
 0
 0
 0
 0
 0
 0
 0
 0
 0
 0
 0
 0
 0
 0
 8.96249730235979E-04

 0
 0
 0
 0
 0
 0
 0
 0
 0
 0
 0
 0
 0
 0
 0
 0
 0
 0
 0
 0
 0
 0
 0
 0
 0
 0
 8.96249730235979E-04

 0
 0
 0
 0
 0
 0
 0
 9.92467066500756E-04
 0
 0
 0
 0
 0
 0
 0
 0
 0
 0
 0
 0
 0
 0
 0
 0
 0
 0
 0
 8.99278807287793E-04

 0
 0
 0
 0
 0
 0
 0
 9.92467066500756E-04
 0
 0
 0
 0
 0
 0
 0
 0
 0
 0
 0
 0
 0
 0
 0
 0
 0
 0
 0
 8.99278807287793E-04

 0
 0
 0
 0
 0
 0
 0
 9.92467066500756E-04
 0
 0
 0
 0
 0
 0
 0
 0
 0
 0
 0
 0
 0
 0
 0
 0
 0
 0
 0
 3.59711522915117E-04

 0
 0
 0
 0
 0
 0
 0
 9.92467066500756E-04
 0
 0
 0
 0
 0
 0
 0
 0
 0
 0
 0
 0
 0
 0
 0
 0
 0
 0
 0
 3.59711522915117E-04

 0
 0
 0
 0
 0
 0
 0
 0
 3.0053218927298E-03
 0
 0
 0
 0
 0
 0
 0
 0
 0
 0
 0
 0
 0
 0
 0
 0
 0
 0
 0
 1.13347365129126E-03

 0
 0
 0
 0
 0
 0
 0
 0
 3.0053218927298E-03
 0
 0
 0
 0
 0
 0
 0
 0
 0
 0
 0
 0
 0
 0
 0
 0
 0
 0
 0
 1.13347365129126E-03

 0
 0
 0
 0
 0
 0
 0
 0
 2.2347265356224E-03
 0
 0
 0
 0
 0
 0
 0
 0
 0
 0
 0
 0
 0
 0
 0
 0
 0
 0
 0
 0

 0
 0
 0
 0
 0
 0
 0
 0
 2.2347265356224E-03
 0
 0
 0
 0
 0
 0
 0
 0
 0
 0
 0
 0
 0
 0
 0
 0
 0
 0
 0
 0

 0
 0
 0
 0
 0
 0
 0
 0
 0
 7.11226754851749E-04
 0
 0
 0
 0
 0
 0
 0
 0
 0
 0
 0
 0
 0
 0
 0
 0
 0
 0
 0
 4.25112734516363E-03

 0
 0
 0
 0
 0
 0
 0
 0
 0
 7.11226754851749E-04
 0
 0
 0
 0
 0
 0
 0
 0
 0
 0
 0
 0
 0
 0
 0
 0
 0
 0
 0
 4.25112734516363E-03

 0
 0
 0
 0
 0
 0
 0
 0
 0
 1.42245350970506E-03
 0
 0
 0
 0
 0
 0
 0
 0
 0
 0
 0
 0
 0
 0
 0
 0
 0
 0
 0
 1.06278183628914E-03

 0
 0
 0
 0
 0
 0
 0
 0
 0
 1.42245350970506E-03
 0
 0
 0
 0
 0
 0
 0
 0
 0
 0
 0
 0
 0
 0
 0
 0
 0
 0
 0
 1.06278183628914E-03

 0
 0
 0
 0
 0
 0
 0
 0
 0
 0
 3.54640496577987E-04
 0
 0
 0
 0
 0
 0
 0
 0
 0
 0
 0
 0
 0
 0
 0
 0
 0
 0
 0
 7.9045556332954E-03

 0
 0
 0
 0
 0
 0
 0
 0
 0
 0
 3.54640496577987E-04
 0
 0
 0
 0
 0
 0
 0
 0
 0
 0
 0
 0
 0
 0
 0
 0
 0
 0
 0
 7.9045556332954E-03

 0
 0
 0
 0
 0
 0
 0
 0
 0
 0
 7.09280993156755E-04
 0
 0
 0
 0
 0
 0
 0
 0
 0
 0
 0
 0
 0
 0
 0
 0
 0
 0
 0
 2.02680913674141E-04

 0
 0
 0
 0
 0
 0
 0
 0
 0
 0
 7.09280993156755E-04
 0
 0
 0
 0
 0
 0
 0
 0
 0
 0
 0
 0
 0
 0
 0
 0
 0
 0
 0
 2.02680913674141E-04

 0
 0
 0
 0
 0
 0
 0
 0
 0
 0
 0
 0
 0
 0
 0
 0
 0
 0
 0
 0
 0
 0
 0
 0
 0
 0
 0
 0
 0
 0
 0
 2.20312546819478E-04

 0
 0
 0
 0
 0
 0
 0
 0
 0
 0
 0
 0
 0
 0
 0
 0
 0
 0
 0
 0
 0
 0
 0
 0
 0
 0
 0
 0
 0
 0
 0
 2.20312546819478E-04

 0
 0
 0
 0
 0
 0
 0
 0
 0
 0
 0
 4.13494121524506E-04
 0
 0
 0
 0
 0
 0
 0
 0
 0
 0
 0
 0
 0
 0
 0
 0
 0
 0
 0
 0

 0
 0
 0
 0
 0
 0
 0
 0
 0
 0
 0
 4.13494121524506E-04
 0
 0
 0
 0
 0
 0
 0
 0
 0
 0
 0
 0
 0
 0
 0
 0
 0
 0
 0
 0

 0
 0
 0
 0
 0
 0
 0
 0
 0
 0
 0
 0
 4.07037797737746E-04

 0
 0
 0
 0
 0
 0
 0
 0
 0
 0
 0
 0
 4.07037797737746E-04

 0
 0
 0
 0
 0
 0
 0
 0
 0
 0
 0
 0
 8.95483155024654E-04

 0
 0
 0
 0
 0
 0
 0
 0
 0
 0
 0
 0
 8.95483155024654E-04

 0
 0
 0
 0
 0
 0
 0
 0
 0
 0
 0
 0
 0
 0

 0
 0
 0
 0
 0
 0
 0
 0
 0
 0
 0
 0
 0
 0

 0
 0
 0
 0
 0
 0
 0
 0
 0
 0
 0
 0
 0
 1.8066664939606E-04

 0
 0
 0
 0
 0
 0
 0
 0
 0
 0
 0
 0
 0
 1.8066664939606E-04

 0
 0
 0
 0
 0
 0
 0
 0
 0
 0
 0
 0
 0
 0
 4.45962280963721E-04

 0
 0
 0
 0
 0
 0
 0
 0
 0
 0
 0
 0
 0
 0
 4.45962280963721E-04

 0
 0
 0
 0
 0
 0
 0
 0
 0
 0
 0
 0
 0
 0
 5.35154737158232E-04

 0
 0
 0
 0
 0
 0
 0
 0
 0
 0
 0
 0
 0
 0
 5.35154737158232E-04

 0
 0
 0
 0
 0
 0
 0
 0
 0
 0
 0
 0
 0
 0
 0
 1.25869493646893E-04

 0
 0
 0
 0
 0
 0
 0
 0
 0
 0
 0
 0
 0
 0
 0
 1.25869493646893E-04

 0
 0
 0
 0
 0
 0
 0
 0
 0
 0
 0
 0
 0
 0
 0
 1.25869493646893E-04

 0
 0
 0
 0
 0
 0
 0
 0
 0
 0
 0
 0
 0
 0
 0
 1.25869493646893E-04

 0
 0
 0
 0
 0
 0
 0
 0
 0
 0
 0
 0
 0
 0
 0
 0
 3.12083006148618E-04

 0
 0
 0
 0
 0
 0
 0
 0
 0
 0
 0
 0
 0
 0
 0
 0
 3.12083006148618E-04

 0
 0
 0
 0
 0
 0
 0
 0
 0
 0
 0
 0
 0
 0
 0
 0
 5.20138343579885E-04

 0
 0
 0
 0
 0
 0
 0
 0
 0
 0
 0
 0
 0
 0
 0
 0
 5.20138343579885E-04

 0
 0
 0
 0
 0
 0
 0
 0
 0
 0
 0
 0
 0
 0
 0
 0
 0
 0

 0
 0
 0
 0
 0
 0
 0
 0
 0
 0
 0
 0
 0
 0
 0
 0
 0
 0

 0
 0
 0
 0
 0
 0
 0
 0
 0
 0
 0
 0
 0
 0
 0
 0
 0
 0

 0
 0
 0
 0
 0
 0
 0
 0
 0
 0
 0
 0
 0
 0
 0
 0
 0
 0

 0
 0
 0
 0
 0
 0
 0
 0
 0
 0
 0
 0
 0
 0
 0
 0
 0
 0
 2.734062965466E-04

 0
 0
 0
 0
 0
 0
 0
 0
 0
 0
 0
 0
 0
 0
 0
 0
 0
 0
 2.734062965466E-04

 0
 0
 0
 0
 0
 0
 0
 0
 0
 0
 0
 0
 0
 0
 0
 0
 0
 0
 6.83515741365372E-04

 0
 0
 0
 0
 0
 0
 0
 0
 0
 0
 0
 0
 0
 0
 0
 0
 0
 0
 6.83515741365372E-04

 0
 0
 0
 0
 0
 0
 0
 0
 0
 0
 0
 0
 0
 0
 0
 0
 0
 0
 0
 3.23506083734736E-04

 0
 0
 0
 0
 0
 0
 0
 0
 0
 0
 0
 0
 0
 0
 0
 0
 0
 0
 0
 3.23506083734736E-04

 0
 0
 0
 0
 0
 0
 0
 0
 0
 0
 0
 0
 0
 0
 0
 0
 0
 0
 0
 3.23506083734736E-04

 0
 0
 0
 0
 0
 0
 0
 0
 0
 0
 0
 0
 0
 0
 0
 0
 0
 0
 0
 3.23506083734736E-04

 0
 0
 0
 0
 0
 0
 0
 0
 0
 0
 0
 0
 0
 0
 0
 0
 0
 0
 0
 0
 0
 0
 0
 0
 0
 1.40433085891629E-04
 0
 0
 0
 0
 1.24986563432387E-04
 1.35859403872641E-04

 0
 0
 0
 0
 0
 0
 0
 0
 0
 0
 0
 0
 0
 0
 0
 0
 0
 0
 0
 0
 0
 0
 0
 0
 0
 1.40433085891629E-04
 0
 0
 0
 0
 1.24986563432387E-04
 1.35859403872641E-04

 0
 0
 0
 0
 0
 0
 0
 0
 0
 0
 0
 0
 0
 0
 0
 0
 0
 0
 0
 0
 0
 0
 0
 0
 0
 1.40433085891629E-04
 0
 0
 0
 0
 1.24986563432387E-04
 1.35859403872641E-04

 0
 0
 0
 0
 0
 0
 0
 0
 0
 0
 0
 0
 0
 0
 0
 0
 0
 0
 0
 0
 0
 0
 0
 0
 0
 1.40433085891629E-04
 0
 0
 0
 0
 1.24986563432387E-04
 1.35859403872641E-04

 0
 0
 0
 0
 0
 0
 0
 0
 0
 0
 0
 0
 0
 0
 0
 0
 0
 0
 0
 0
 1.74817033157218E-04

 0
 0
 0
 0
 0
 0
 0
 0
 0
 0
 0
 0
 0
 0
 0
 0
 0
 0
 0
 0
 1.74817033157218E-04

 0
 0
 0
 0
 0
 0
 0
 0
 0
 0
 0
 0
 0
 0
 0
 0
 0
 0
 0
 0
 1.74817033157218E-04

 0
 0
 0
 0
 0
 0
 0
 0
 0
 0
 0
 0
 0
 0
 0
 0
 0
 0
 0
 0
 1.74817033157218E-04

 0
 0
 0
 0
 0
 0
 0
 0
 0
 0
 0
 0
 0
 0
 0
 0
 0
 0
 0
 0
 1.74817033157218E-04

 1.83959177530513E-04
 2.13109850799534E-04
 1.76805808108816E-04
 4.45159067768201E-04
 0
 1.71442612827786E-04
 7.71831583883422E-04
 0
 0
 0
 0
 0
 0
 0
 0
 0
 0
 0
 0
 0
 0
 0
 0
 0
 0
 0
 0
 0
 7.76639353663749E-04
 0
 0
 0

 1.83959177530513E-04
 2.13109850799534E-04
 1.76805808108816E-04
 4.45159067768201E-04
 0
 1.71442612827786E-04
 7.71831583883422E-04
 0
 0
 0
 0
 0
 0
 0
 0
 0
 0
 0
 0
 0
 0
 0
 0
 0
 0
 0
 0
 0
 7.76639353663749E-04
 0
 0
 0

 1.83959177530513E-04
 2.13109850799534E-04
 1.76805808108816E-04
 4.45159067768201E-04
 0
 1.71442612827786E-04
 7.71831583883422E-04
 0
 0
 0
 0
 0
 0
 0
 0
 0
 0
 0
 0
 0
 0
 0
 0
 0
 0
 0
 0
 0
 7.76639353663749E-04
 0
 0
 0

 1.83959177530513E-04
 0
 0
 0
 0
 0
 0
 0
 0
 0
 0
 0
 0
 0
 0
 0
 0
 0
 0
 0
 0

 1.83959177530513E-04
 0
 0
 0
 0
 0
 0
 0
 0
 0
 0
 0
 0
 0
 0
 0
 0
 0
 0
 0
 0

 0
 2.13109850799534E-04
 0
 0
 0
 0
 0
 0
 0
 0
 0
 0
 0
 0
 0
 0
 0
 0
 0
 0
 0
 0

 0
 2.13109850799534E-04
 0
 0
 0
 0
 0
 0
 0
 0
 0
 0
 0
 0
 0
 0
 0
 0
 0
 0
 0
 0

 0
 0
 1.76805808108816E-04
 0
 0
 0
 0
 0
 0
 0
 0
 0
 0
 0
 0
 0
 0
 0
 0
 0
 0
 0
 0

 0
 0
 1.76805808108816E-04
 0
 0
 0
 0
 0
 0
 0
 0
 0
 0
 0
 0
 0
 0
 0
 0
 0
 0
 0
 0

 0
 0
 0
 4.45159067768201E-04
 0
 0
 0
 0
 0
 0
 0
 0
 0
 0
 0
 0
 0
 0
 0
 0
 0
 0
 0
 0

 0
 0
 0
 4.45159067768201E-04
 0
 0
 0
 0
 0
 0
 0
 0
 0
 0
 0
 0
 0
 0
 0
 0
 0
 0
 0
 0

 0
 0
 0
 0
 0
 0
 0
 0
 0
 0
 0
 0
 0
 0
 0
 0
 0
 0
 0
 0
 0
 0
 0
 0
 0

 0
 0
 0
 0
 0
 0
 0
 0
 0
 0
 0
 0
 0
 0
 0
 0
 0
 0
 0
 0
 0
 0
 0
 0
 0

 0
 0
 0
 0
 0
 1.71442612827786E-04
 0
 0
 0
 0
 0
 0
 0
 0
 0
 0
 0
 0
 0
 0
 0
 0
 0
 0
 0
 0

 0
 0
 0
 0
 0
 1.71442612827786E-04
 0
 0
 0
 0
 0
 0
 0
 0
 0
 0
 0
 0
 0
 0
 0
 0
 0
 0
 0
 0

 0
 0
 0
 0
 0
 0
 7.71831583883422E-04
 0
 0
 0
 0
 0
 0
 0
 0
 0
 0
 0
 0
 0
 0
 0
 0
 0
 0
 0
 0

 0
 0
 0
 0
 0
 0
 7.71831583883422E-04
 0
 0
 0
 0
 0
 0
 0
 0
 0
 0
 0
 0
 0
 0
 0
 0
 0
 0
 0
 0

 0
 0
 0
 0
 0
 0
 0
 0
 0
 0
 0
 0
 0
 0
 0
 0
 0
 0
 0
 0
 0
 0
 0
 0
 0
 0
 0
 0

 0
 0
 0
 0
 0
 0
 0
 0
 0
 0
 0
 0
 0
 0
 0
 0
 0
 0
 0
 0
 0
 0
 0
 0
 0
 0
 0
 0

 0
 0
 0
 0
 0
 0
 0
 0
 0
 0
 0
 0
 0
 0
 0
 0
 0
 0
 0
 0
 0
 0
 0
 0
 0
 0
 0
 0
 7.76639353663749E-04

 0
 0
 0
 0
 0
 0
 0
 0
 0
 0
 0
 0
 0
 0
 0
 0
 0
 0
 0
 0
 0
 0
 0
 0
 0
 0
 0
 0
 7.76639353663749E-04

 0
 0
 0
 0
 0
 0
 0
 0
 0
 0
 0
 0
 0
 0
 0
 0
 0
 0
 0
 0
 0
 0
 0
 0
 0
 0
 0
 0
 0
 0

 0
 0
 0
 0
 0
 0
 0
 0
 0
 0
 0
 0
 0
 0
 0
 0
 0
 0
 0
 0
 0
 0
 0
 0
 0
 0
 0
 0
 0
 0

 0
 0
 0
 0
 0
 0
 0
 0
 0
 0
 0
 0
 0
 0
 0
 0
 0
 0
 0
 0
 0
 0
 0
 0
 0
 0
 0
 0
 0
 0
 0

 0
 0
 0
 0
 0
 0
 0
 0
 0
 0
 0
 0
 0
 0
 0
 0
 0
 0
 0
 0
 0
 0
 0
 0
 0
 0
 0
 0
 0
 0
 0

 0
 0
 0
 0
 0
 0
 0
 0
 0
 0
 0
 0
 0
 0
 0
 0
 0
 0
 0
 0
 0
 0
 0
 0
 0
 0
 0
 0
 0
 0
 0
 0

 0
 0
 0
 0
 0
 0
 0
 0
 0
 0
 0
 0
 0
 0
 0
 0
 0
 0
 0
 0
 0
 0
 0
 0
 0
 0
 0
 0
 0
 0
 0
 0

 0
 0
 0
 0
 0
 0
 0
 0
 0
 0
 0
 0
 0

 0
 0
 0
 0
 0
 0
 0
 0
 0
 0
 0
 0
 0

 0
 0
 0
 0
 0
 0
 0
 0
 0
 0
 0
 0
 0
 0

 0
 0
 0
 0
 0
 0
 0
 0
 0
 0
 0
 0
 0
 0

 0
 0
 0
 0
 0
 0
 0
 0
 0
 0
 0
 0
 0
 0
 0

 0
 0
 0
 0
 0
 0
 0
 0
 0
 0
 0
 0
 0
 0
 0

 0
 0
 0
 0
 0
 0
 0
 0
 0
 0
 0
 0
 0
 0
 0
 0

 0
 0
 0
 0
 0
 0
 0
 0
 0
 0
 0
 0
 0
 0
 0
 0

 0
 0
 0
 0
 0
 0
 0
 0
 0
 0
 0
 0
 0
 0
 0
 0
 0

 0
 0
 0
 0
 0
 0
 0
 0
 0
 0
 0
 0
 0
 0
 0
 0
 0

 0
 0
 0
 0
 0
 0
 0
 0
 0
 0
 0
 0
 0
 0
 0
 0
 0
 0

 0
 0
 0
 0
 0
 0
 0
 0
 0
 0
 0
 0
 0
 0
 0
 0
 0
 0

 0
 0
 0
 0
 0
 0
 0
 0
 0
 0
 0
 0
 0
 0
 0
 0
 0
 0
 0

 0
 0
 0
 0
 0
 0
 0
 0
 0
 0
 0
 0
 0
 0
 0
 0
 0
 0
 0

 0
 0
 0
 0
 0
 0
 0
 0
 0
 0
 0
 0
 0
 0
 0
 0
 0
 0
 0
 0

 0
 0
 0
 0
 0
 0
 0
 0
 0
 0
 0
 0
 0
 0
 0
 0
 0
 0
 0
 0

 1.40691313059578E-02
 8.19267553117444E-03
 1.17795869619448E-02
 1.06596460933541E-02
 2.05468914674764E-02
 1.57789264475396E-02
 9.89062011564289E-03
 2.64858473520422E-02
 2.47067690631636E-02
 9.80377271884961E-03
 7.69885470172459E-03
 2.86613481518496E-02
 .019286169158841
 1.09510558158859E-02
 1.34244736214729E-02
 1.39504406216821E-02
 1.71551510695109E-02
 9.24163279865232E-03
 9.81850259069458E-03
 1.24761365446451E-02
 4.05615068289483E-02
 4.83501261541462E-03
 4.44952372443924E-04
 5.72545751346139E-02
 2.36983359041512E-02
 .02135091260624
 3.33675599510842E-02
 .010689617315599
 6.69245242135057E-02
 2.57556281435806E-02
 6.5151367001966E-03
 3.50406607272148E-03

 5.09425414699041E-04
 0
 0
 9.2456114074985E-04
 0
 0
 3.56229961792741E-04
 0
 0
 0
 0
 0
 0
 0
 2.53855452241726E-04
 0
 0
 0
 0
 0
 1.26406162436532E-03
 0
 0
 5.91029922450606E-03
 2.80746836494847E-03
 2.26853446441092E-03
 1.27543230841274E-03
 5.11897167225359E-04
 5.37673398690288E-03
 0
 8.65291592993446E-04
 0

 5.09425414699041E-04
 0
 0
 9.2456114074985E-04
 0
 0
 3.56229961792741E-04
 0
 0
 0
 0
 0
 0
 0
 2.53855452241726E-04
 0
 0
 0
 0
 0
 1.26406162436532E-03
 0
 0
 5.91029922450606E-03
 2.80746836494847E-03
 2.26853446441092E-03
 1.27543230841274E-03
 5.11897167225359E-04
 5.37673398690288E-03
 0
 8.65291592993446E-04
 0

 5.09425414699041E-04
 0
 0
 9.2456114074985E-04
 0
 0
 3.56229961792741E-04
 0
 0
 0
 0
 0
 0
 0
 2.53855452241726E-04
 0
 0
 0
 0
 0
 1.26406162436532E-03
 0
 0
 5.91029922450606E-03
 2.80746836494847E-03
 2.26853446441092E-03
 1.27543230841274E-03
 5.11897167225359E-04
 5.37673398690288E-03
 0
 8.65291592993446E-04
 0

 5.09425414699041E-04
 0
 0
 9.2456114074985E-04
 0
 0
 3.56229961792741E-04
 0
 0
 0
 0
 0
 0
 0
 2.53855452241726E-04
 0
 0
 0
 0
 0
 1.26406162436532E-03
 0
 0
 5.91029922450606E-03
 2.80746836494847E-03
 2.26853446441092E-03
 1.27543230841274E-03
 5.11897167225359E-04
 5.37673398690288E-03
 0
 8.65291592993446E-04
 0

 1.30502804765597E-02
 7.89760035314431E-03
 1.10451628359551E-02
 9.42689790568733E-03
 .020340694799857
 1.50667802096404E-02
 9.53439015385014E-03
 2.59209045603427E-02
 2.28096225705186E-02
 9.80377271884961E-03
 6.68949328838724E-03
 2.36988885728815E-02
 1.61234854704121E-02
 1.06168225145031E-02
 1.16474854557808E-02
 1.28757087913136E-02
 1.44904423247054E-02
 8.92552061036801E-03
 9.81850259069458E-03
 1.19697250981829E-02
 3.86654143923992E-02
 3.17450323234504E-03
 0
 4.21833121121218E-02
 1.87852662654892E-02
 1.90823781418291E-02
 3.14544114884651E-02
 1.01777201483736E-02
 5.50957094423042E-02
 2.19745773798587E-02
 5.07298404520753E-03
 2.87702267023527E-03

 1.30502804765597E-02
 7.89760035314431E-03
 1.10451628359551E-02
 9.42689790568733E-03
 .020340694799857
 1.50667802096404E-02
 9.53439015385014E-03
 2.59209045603427E-02
 2.28096225705186E-02
 9.80377271884961E-03
 6.68949328838724E-03
 2.36988885728815E-02
 1.61234854704121E-02
 1.06168225145031E-02
 1.16474854557808E-02
 1.28757087913136E-02
 1.44904423247054E-02
 8.92552061036801E-03
 9.81850259069458E-03
 1.19697250981829E-02
 3.86654143923992E-02
 3.17450323234504E-03
 0
 4.21833121121218E-02
 1.87852662654892E-02
 1.90823781418291E-02
 3.14544114884651E-02
 1.01777201483736E-02
 5.50957094423042E-02
 2.19745773798587E-02
 5.07298404520753E-03
 2.87702267023527E-03

 1.30502804765597E-02
 7.89760035314431E-03
 1.10451628359551E-02
 9.42689790568733E-03
 .020340694799857
 1.50667802096404E-02
 9.53439015385014E-03
 2.59209045603427E-02
 2.28096225705186E-02
 9.80377271884961E-03
 6.68949328838724E-03
 2.36988885728815E-02
 1.61234854704121E-02
 1.06168225145031E-02
 1.16474854557808E-02
 1.28757087913136E-02
 1.44904423247054E-02
 8.92552061036801E-03
 9.81850259069458E-03
 1.19697250981829E-02
 3.86654143923992E-02
 3.17450323234504E-03
 0
 4.21833121121218E-02
 1.87852662654892E-02
 1.90823781418291E-02
 3.14544114884651E-02
 1.01777201483736E-02
 5.50957094423042E-02
 2.19745773798587E-02
 5.07298404520753E-03
 2.87702267023527E-03

 0
 0
 0
 0
 0
 0
 0
 0
 0
 0
 0
 0
 0
 0
 0
 0
 0
 0
 0
 0
 0

 9.739015280995E-04
 1.57952007062812E-03
 9.36030748808291E-04
 1.41403468585427E-03
 9.46078827900448E-04
 5.44582417217072E-04
 1.36205573626187E-03
 4.32015076005498E-04
 6.70871252074402E-04
 0
 0
 0
 0
 0
 0
 2.73951250878532E-04
 0
 0
 0
 0
 4.83317679905249E-04
 0
 0
 0
 0
 4.95646185501825E-04
 0
 0
 1.64464804305458E-03
 0
 0
 0

 3.89560611240443E-04
 0
 0
 0
 0
 0
 0
 0
 0
 0
 0
 0
 0
 0
 0
 0
 0
 0
 0
 0
 0

 5.45384855736878E-03
 0
 0
 0
 0
 0
 0
 0
 0
 0
 0
 0
 0
 0
 0
 0
 0
 0
 0
 0
 1.40162127172315E-02

 6.23296977985095E-03
 3.61033159000819E-03
 5.05456604357218E-03
 4.47777650520129E-03
 8.98774886505166E-03
 5.80887911698251E-03
 4.08616720879459E-03
 8.64030152012423E-03
 4.52838095149391E-03
 3.09592822700514E-03
 2.83017023739575E-03
 6.29970455734592E-03
 5.49261592948224E-03
 1.57286259474217E-03
 6.40611700067864E-03
 2.73951250878532E-03
 7.69804748500024E-03
 2.97517353679343E-03
 6.54566839379311E-03
 7.74511623999911E-03
 2.41658839952624E-02
 2.53960258587603E-03
 0
 3.61571246675273E-02
 1.66383786922933E-02
 5.69993113327588E-03
 6.82731412149896E-03
 3.52305697444397E-03
 .039471553033279
 3.46967011259871E-03
 1.54395166592948E-03
 7.19255667558819E-04

 0
 0
 0
 0
 0
 0
 0
 0
 0
 0
 0
 0
 0
 0
 0
 0
 0
 0
 0
 0
 0
 0

 0
 2.25645724375978E-04
 0
 0
 0
 0
 0
 0
 0
 0
 0
 0
 0
 0
 0
 0
 0
 0
 0
 0
 0
 0

 0
 2.48210296813203E-03
 0
 0
 0
 0
 0
 0
 0
 0
 0
 0
 0
 0
 0
 0
 0
 0
 0
 0
 0
 6.34900646469007E-04

 0
 0
 0
 0
 0
 0
 0
 0
 0
 0
 0
 0
 0
 0
 0
 0
 0
 0
 0
 0
 0
 0
 0

 0
 0
 5.6161844928621E-04
 0
 0
 0
 0
 0
 0
 0
 0
 0
 0
 0
 0
 0
 0
 0
 0
 0
 0
 0
 0

 0
 0
 4.49294759428845E-03
 0
 0
 0
 0
 0
 0
 0
 0
 0
 0
 0
 0
 0
 0
 0
 0
 0
 0
 0
 0

 0
 0
 0
 0
 0
 0
 0
 0
 0
 0
 0
 0
 0
 0
 0
 0
 0
 0
 0
 0
 0
 0
 0
 0

 0
 0
 0
 0
 0
 0
 0
 0
 0
 0
 0
 0
 0
 0
 0
 0
 0
 0
 0
 0
 0
 0
 0
 0

 0
 0
 0
 3.53508671463178E-03
 0
 0
 0
 0
 0
 0
 0
 0
 0
 0
 0
 0
 0
 0
 0
 0
 0
 0
 0
 6.02618744459442E-03

 0
 0
 0
 0
 0
 0
 0
 0
 0
 0
 0
 0
 0
 0
 0
 0
 0
 0
 0
 0
 0
 0
 0
 0
 0

 0
 0
 0
 0
 9.46078827900448E-04
 0
 0
 0
 0
 0
 0
 0
 0
 0
 0
 0
 0
 0
 0
 0
 0
 0
 0
 0
 0

 0
 0
 0
 0
 9.46078827900448E-03
 0
 0
 0
 0
 0
 0
 0
 0
 0
 0
 0
 0
 0
 0
 0
 0
 0
 0
 0
 2.14688757319589E-03

 0
 0
 0
 0
 0
 0
 0
 0
 0
 0
 0
 0
 0
 0
 0
 0
 0
 0
 0
 0
 0
 0
 0
 0
 0
 0

 0
 0
 0
 0
 0
 1.27069230683824E-03
 0
 0
 0
 0
 0
 0
 0
 0
 0
 0
 0
 0
 0
 0
 0
 0
 0
 0
 0
 2.47823092749934E-04

 0
 0
 0
 0
 0
 7.44262636860256E-03
 0
 0
 0
 0
 0
 0
 0
 0
 0
 0
 0
 0
 0
 0
 0
 0
 0
 0
 0
 1.26389777303014E-02

 0
 0
 0
 0
 0
 0
 0
 0
 0
 0
 0
 0
 0
 0
 0
 0
 0
 0
 0
 0
 0
 0
 0
 0
 0
 0
 2.43832647197684E-04

 0
 0
 0
 0
 0
 0
 5.44822294505645E-04
 0
 0
 0
 0
 0
 0
 0
 0
 0
 0
 0
 0
 0
 0
 0
 0
 0
 0
 0
 0

 0
 0
 0
 0
 0
 0
 3.54134491428804E-03
 0
 0
 0
 0
 0
 0
 0
 0
 0
 0
 0
 0
 0
 0
 0
 0
 0
 0
 0
 2.43832647197684E-02

 0
 0
 0
 0
 0
 0
 0
 0
 0
 0
 0
 0
 0
 0
 0
 0
 0
 0
 0
 0
 0
 0
 0
 0
 0
 0
 0
 0

 0
 0
 0
 0
 0
 0
 0
 4.32015076005498E-04
 0
 0
 0
 0
 0
 0
 0
 0
 0
 0
 0
 0
 0
 0
 0
 0
 0
 0
 0
 1.17435232481112E-03

 0
 0
 0
 0
 0
 0
 0
 1.64165728882075E-02
 0
 0
 0
 0
 0
 0
 0
 0
 0
 0
 0
 0
 0
 0
 0
 0
 0
 0
 0
 5.48031084911855E-03

 0
 0
 0
 0
 0
 0
 0
 0
 0
 0
 0
 0
 0
 0
 0
 0
 0
 0
 0
 0
 0
 0
 0
 0
 0
 0
 0
 0
 0

 0
 0
 0
 0
 0
 0
 0
 0
 1.3417425041488E-03
 0
 0
 0
 0
 0
 0
 0
 0
 0
 0
 0
 0
 0
 0
 0
 0
 0
 0
 0
 0

 0
 0
 0
 0
 0
 0
 0
 0
 1.62686278628015E-02
 0
 0
 0
 0
 0
 0
 0
 0
 0
 0
 0
 0
 0
 0
 0
 0
 0
 0
 0
 1.39795083659707E-02

 0
 0
 0
 0
 0
 0
 0
 0
 0
 0
 0
 0
 0
 0
 0
 0
 0
 0
 0
 0
 0
 0
 0
 0
 0
 0
 0
 0
 0
 0

 0
 0
 0
 0
 0
 0
 0
 0
 0
 5.1598803783419E-04
 0
 0
 0
 0
 0
 0
 0
 0
 0
 0
 0
 0
 0
 0
 0
 0
 0
 0
 0
 0

 0
 0
 0
 0
 0
 0
 0
 0
 0
 6.19185645401028E-03
 0
 0
 0
 0
 0
 0
 0
 0
 0
 0
 0
 0
 0
 0
 0
 0
 0
 0
 0
 1.67700722109606E-02

 0
 0
 0
 0
 0
 0
 0
 0
 0
 0
 0
 0
 0
 0
 0
 0
 0
 0
 0
 0
 0
 0
 0
 0
 0
 0
 0
 0
 0
 0
 0

 0
 0
 0
 0
 0
 0
 0
 0
 0
 0
 2.57288203399999E-04
 0
 0
 0
 0
 0
 0
 0
 0
 0
 0
 0
 0
 0
 0
 0
 0
 0
 0
 0
 2.20564523704212E-04

 0
 0
 0
 0
 0
 0
 0
 0
 0
 0
 3.6020348475915E-03
 0
 0
 0
 0
 0
 0
 0
 0
 0
 0
 0
 0
 0
 0
 0
 0
 0
 0
 0
 3.30846785557383E-03

 0
 0
 0
 0
 0
 0
 0
 0
 0
 0
 0
 0
 0
 0
 0
 0
 0
 0
 0
 0
 0
 0
 0
 0
 0
 0
 0
 0
 0
 0
 0
 0

 0
 0
 0
 0
 0
 0
 0
 0
 0
 0
 0
 2.99985931302423E-04
 0
 0
 0
 0
 0
 0
 0
 0
 0
 0
 0
 0
 0
 0
 0
 0
 0
 0
 0
 0

 0
 0
 0
 0
 0
 0
 0
 0
 0
 0
 0
 1.70991980842331E-02
 0
 0
 0
 0
 0
 0
 0
 0
 0
 0
 0
 0
 0
 0
 0
 0
 0
 0
 0
 2.15776700267646E-03

 0
 0
 0
 0
 0
 0
 0
 0
 0
 0
 0
 0
 0

 0
 0
 0
 0
 0
 0
 0
 0
 0
 0
 0
 0
 1.77181159015839E-04

 0
 0
 0
 0
 0
 0
 0
 0
 0
 0
 0
 0
 .010453688381914

 0
 0
 0
 0
 0
 0
 0
 0
 0
 0
 0
 0
 0
 0

 0
 0
 0
 0
 0
 0
 0
 0
 0
 0
 0
 0
 0
 0

 0
 0
 0
 0
 0
 0
 0
 0
 0
 0
 0
 0
 0
 9.04395991976097E-03

 0
 0
 0
 0
 0
 0
 0
 0
 0
 0
 0
 0
 0
 0
 0

 0
 0
 0
 0
 0
 0
 0
 0
 0
 0
 0
 0
 0
 0
 1.94124757596614E-04

 0
 0
 0
 0
 0
 0
 0
 0
 0
 0
 0
 0
 0
 0
 5.04724369750554E-03

 0
 0
 0
 0
 0
 0
 0
 0
 0
 0
 0
 0
 0
 0
 0
 0

 0
 0
 0
 0
 0
 0
 0
 0
 0
 0
 0
 0
 0
 0
 0
 2.73951250878532E-04

 0
 0
 0
 0
 0
 0
 0
 0
 0
 0
 0
 0
 0
 0
 0
 9.58829378077124E-03

 0
 0
 0
 0
 0
 0
 0
 0
 0
 0
 0
 0
 0
 0
 0
 0
 0

 0
 0
 0
 0
 0
 0
 0
 0
 0
 0
 0
 0
 0
 0
 0
 0
 0

 0
 0
 0
 0
 0
 0
 0
 0
 0
 0
 0
 0
 0
 0
 0
 0
 6.79239483970521E-03

 0
 0
 0
 0
 0
 0
 0
 0
 0
 0
 0
 0
 0
 0
 0
 0
 0
 0

 0
 0
 0
 0
 0
 0
 0
 0
 0
 0
 0
 0
 0
 0
 0
 0
 0
 0

 0
 0
 0
 0
 0
 0
 0
 0
 0
 0
 0
 0
 0
 0
 0
 0
 0
 5.95034707357458E-03

 0
 0
 0
 0
 0
 0
 0
 0
 0
 0
 0
 0
 0
 0
 0
 0
 0
 0
 2.97530381536497E-04

 0
 0
 0
 0
 0
 0
 0
 0
 0
 0
 0
 0
 0
 0
 0
 0
 0
 0
 0

 0
 0
 0
 0
 0
 0
 0
 0
 0
 0
 0
 0
 0
 0
 0
 0
 0
 0
 2.97530381536497E-03

 0
 0
 0
 0
 0
 0
 0
 0
 0
 0
 0
 0
 0
 0
 0
 0
 0
 0
 0
 2.34700492121537E-04

 0
 0
 0
 0
 0
 0
 0
 0
 0
 0
 0
 0
 0
 0
 0
 0
 0
 0
 0
 0

 0
 0
 0
 0
 0
 0
 0
 0
 0
 0
 0
 0
 0
 0
 0
 0
 0
 0
 0
 3.98990836606226E-03

 0
 0
 0
 0
 0
 0
 0
 0
 0
 0
 0
 0
 0
 0
 0
 0
 0
 0
 0
 0
 0
 0
 0
 0
 0
 0
 0
 0
 0
 1.73483505629936E-03
 0
 0

 0
 0
 0
 0
 0
 0
 0
 0
 1.4256014106581E-04
 0
 0
 2.54988041607059E-04
 1.50603985163463E-04
 3.34233301382711E-04
 0
 0
 0
 3.16112188284302E-04
 0
 1.99495418303307E-04
 0
 0
 4.44952372443924E-04
 0
 0
 0
 0
 0
 0
 0
 0
 0

 0
 0
 0
 0
 0
 0
 0
 0
 0
 0
 0
 0
 0
 0
 0
 0
 0
 0
 0
 0
 0

 0
 0
 0
 0
 0
 0
 0
 0
 0
 0
 0
 0
 0
 0
 0
 0
 0
 0
 0
 0
 0

 0
 0
 0
 0
 0
 0
 0
 0
 0
 0
 0
 0
 0
 0
 0
 0
 0
 0
 0
 0
 0

 0
 0
 0
 0
 0
 0
 0
 0
 0
 0
 0
 0
 0
 0
 0
 0
 0
 0
 0
 0
 0
 0

 0
 0
 0
 0
 0
 0
 0
 0
 0
 0
 0
 0
 0
 0
 0
 0
 0
 0
 0
 0
 0
 0

 0
 0
 0
 0
 0
 0
 0
 0
 0
 0
 0
 0
 0
 0
 0
 0
 0
 0
 0
 0
 0
 0

 0
 0
 0
 0
 0
 0
 0
 0
 0
 0
 0
 0
 0
 0
 0
 0
 0
 0
 0
 0
 0
 0
 4.44952372443924E-04

 0
 0
 0
 0
 0
 0
 0
 0
 0
 0
 0
 0
 0
 0
 0
 0
 0
 0
 0
 0
 0
 0
 4.44952372443924E-04

 0
 0
 0
 0
 0
 0
 0
 0
 0
 0
 0
 0
 0
 0
 0
 0
 0
 0
 0
 0
 0
 0
 4.44952372443924E-04

 0
 0
 0
 0
 0
 0
 0
 0
 0
 0
 0
 0
 0
 0
 0
 0
 0
 0
 0
 0
 0
 0
 0
 0

 0
 0
 0
 0
 0
 0
 0
 0
 0
 0
 0
 0
 0
 0
 0
 0
 0
 0
 0
 0
 0
 0
 0
 0

 0
 0
 0
 0
 0
 0
 0
 0
 0
 0
 0
 0
 0
 0
 0
 0
 0
 0
 0
 0
 0
 0
 0
 0

 0
 0
 0
 0
 0
 0
 0
 0
 0
 0
 0
 0
 0
 0
 0
 0
 0
 0
 0
 0
 0
 0
 0
 0
 0

 0
 0
 0
 0
 0
 0
 0
 0
 0
 0
 0
 0
 0
 0
 0
 0
 0
 0
 0
 0
 0
 0
 0
 0
 0

 0
 0
 0
 0
 0
 0
 0
 0
 0
 0
 0
 0
 0
 0
 0
 0
 0
 0
 0
 0
 0
 0
 0
 0
 0

 0
 0
 0
 0
 0
 0
 0
 0
 0
 0
 0
 0
 0
 0
 0
 0
 0
 0
 0
 0
 0
 0
 0
 0
 0
 0

 0
 0
 0
 0
 0
 0
 0
 0
 0
 0
 0
 0
 0
 0
 0
 0
 0
 0
 0
 0
 0
 0
 0
 0
 0
 0

 0
 0
 0
 0
 0
 0
 0
 0
 0
 0
 0
 0
 0
 0
 0
 0
 0
 0
 0
 0
 0
 0
 0
 0
 0
 0

 0
 0
 0
 0
 0
 0
 0
 0
 0
 0
 0
 0
 0
 0
 0
 0
 0
 0
 0
 0
 0
 0
 0
 0
 0
 0
 0

 0
 0
 0
 0
 0
 0
 0
 0
 0
 0
 0
 0
 0
 0
 0
 0
 0
 0
 0
 0
 0
 0
 0
 0
 0
 0
 0

 0
 0
 0
 0
 0
 0
 0
 0
 0
 0
 0
 0
 0
 0
 0
 0
 0
 0
 0
 0
 0
 0
 0
 0
 0
 0
 0

 0
 0
 0
 0
 0
 0
 0
 0
 0
 0
 0
 0
 0
 0
 0
 0
 0
 0
 0
 0
 0
 0
 0
 0
 0
 0
 0
 0

 0
 0
 0
 0
 0
 0
 0
 0
 0
 0
 0
 0
 0
 0
 0
 0
 0
 0
 0
 0
 0
 0
 0
 0
 0
 0
 0
 0

 0
 0
 0
 0
 0
 0
 0
 0
 0
 0
 0
 0
 0
 0
 0
 0
 0
 0
 0
 0
 0
 0
 0
 0
 0
 0
 0
 0

 0
 0
 0
 0
 0
 0
 0
 0
 1.4256014106581E-04
 0
 0
 0
 0
 0
 0
 0
 0
 0
 0
 0
 0
 0
 0
 0
 0
 0
 0
 0
 0

 0
 0
 0
 0
 0
 0
 0
 0
 1.4256014106581E-04
 0
 0
 0
 0
 0
 0
 0
 0
 0
 0
 0
 0
 0
 0
 0
 0
 0
 0
 0
 0

 0
 0
 0
 0
 0
 0
 0
 0
 1.4256014106581E-04
 0
 0
 0
 0
 0
 0
 0
 0
 0
 0
 0
 0
 0
 0
 0
 0
 0
 0
 0
 0

 0
 0
 0
 0
 0
 0
 0
 0
 0
 0
 0
 0
 0
 0
 0
 0
 0
 0
 0
 0
 0
 0
 0
 0
 0
 0
 0
 0
 0
 0

 0
 0
 0
 0
 0
 0
 0
 0
 0
 0
 0
 0
 0
 0
 0
 0
 0
 0
 0
 0
 0
 0
 0
 0
 0
 0
 0
 0
 0
 0

 0
 0
 0
 0
 0
 0
 0
 0
 0
 0
 0
 0
 0
 0
 0
 0
 0
 0
 0
 0
 0
 0
 0
 0
 0
 0
 0
 0
 0
 0

 0
 0
 0
 0
 0
 0
 0
 0
 0
 0
 0
 0
 0
 0
 0
 0
 0
 0
 0
 0
 0
 0
 0
 0
 0
 0
 0
 0
 0
 0
 0

 0
 0
 0
 0
 0
 0
 0
 0
 0
 0
 0
 0
 0
 0
 0
 0
 0
 0
 0
 0
 0
 0
 0
 0
 0
 0
 0
 0
 0
 0
 0

 0
 0
 0
 0
 0
 0
 0
 0
 0
 0
 0
 0
 0
 0
 0
 0
 0
 0
 0
 0
 0
 0
 0
 0
 0
 0
 0
 0
 0
 0
 0

 0
 0
 0
 0
 0
 0
 0
 0
 0
 0
 0
 2.54988041607059E-04
 0
 0
 0
 0
 0
 0
 0
 0
 0
 0
 0
 0
 0
 0
 0
 0
 0
 0
 0
 0

 0
 0
 0
 0
 0
 0
 0
 0
 0
 0
 0
 2.54988041607059E-04
 0
 0
 0
 0
 0
 0
 0
 0
 0
 0
 0
 0
 0
 0
 0
 0
 0
 0
 0
 0

 0
 0
 0
 0
 0
 0
 0
 0
 0
 0
 0
 2.54988041607059E-04
 0
 0
 0
 0
 0
 0
 0
 0
 0
 0
 0
 0
 0
 0
 0
 0
 0
 0
 0
 0

 0
 0
 0
 0
 0
 0
 0
 0
 0
 0
 0
 0
 1.50603985163463E-04

 0
 0
 0
 0
 0
 0
 0
 0
 0
 0
 0
 0
 1.50603985163463E-04

 0
 0
 0
 0
 0
 0
 0
 0
 0
 0
 0
 0
 1.50603985163463E-04

 0
 0
 0
 0
 0
 0
 0
 0
 0
 0
 0
 0
 0
 3.34233301382711E-04

 0
 0
 0
 0
 0
 0
 0
 0
 0
 0
 0
 0
 0
 3.34233301382711E-04

 0
 0
 0
 0
 0
 0
 0
 0
 0
 0
 0
 0
 0
 3.34233301382711E-04

 0
 0
 0
 0
 0
 0
 0
 0
 0
 0
 0
 0
 0
 0
 0

 0
 0
 0
 0
 0
 0
 0
 0
 0
 0
 0
 0
 0
 0
 0

 0
 0
 0
 0
 0
 0
 0
 0
 0
 0
 0
 0
 0
 0
 0

 0
 0
 0
 0
 0
 0
 0
 0
 0
 0
 0
 0
 0
 0
 0
 0

 0
 0
 0
 0
 0
 0
 0
 0
 0
 0
 0
 0
 0
 0
 0
 0

 0
 0
 0
 0
 0
 0
 0
 0
 0
 0
 0
 0
 0
 0
 0
 0

 0
 0
 0
 0
 0
 0
 0
 0
 0
 0
 0
 0
 0
 0
 0
 0
 0

 0
 0
 0
 0
 0
 0
 0
 0
 0
 0
 0
 0
 0
 0
 0
 0
 0

 0
 0
 0
 0
 0
 0
 0
 0
 0
 0
 0
 0
 0
 0
 0
 0
 0

 0
 0
 0
 0
 0
 0
 0
 0
 0
 0
 0
 0
 0
 0
 0
 0
 0
 3.16112188284302E-04

 0
 0
 0
 0
 0
 0
 0
 0
 0
 0
 0
 0
 0
 0
 0
 0
 0
 3.16112188284302E-04

 0
 0
 0
 0
 0
 0
 0
 0
 0
 0
 0
 0
 0
 0
 0
 0
 0
 3.16112188284302E-04

 0
 0
 0
 0
 0
 0
 0
 0
 0
 0
 0
 0
 0
 0
 0
 0
 0
 0
 0

 0
 0
 0
 0
 0
 0
 0
 0
 0
 0
 0
 0
 0
 0
 0
 0
 0
 0
 0

 0
 0
 0
 0
 0
 0
 0
 0
 0
 0
 0
 0
 0
 0
 0
 0
 0
 0
 0

 0
 0
 0
 0
 0
 0
 0
 0
 0
 0
 0
 0
 0
 0
 0
 0
 0
 0
 0
 1.99495418303307E-04

 0
 0
 0
 0
 0
 0
 0
 0
 0
 0
 0
 0
 0
 0
 0
 0
 0
 0
 0
 1.99495418303307E-04

 0
 0
 0
 0
 0
 0
 0
 0
 0
 0
 0
 0
 0
 0
 0
 0
 0
 0
 0
 1.99495418303307E-04

 5.09425414699041E-04
 0
 0
 0
 0
 0
 0
 0
 0
 0
 0
 0
 0
 0
 0
 0
 0
 0
 0
 0
 6.32030812183787E-04

 5.09425414699041E-04
 0
 0
 0
 0
 0
 0
 0
 0
 0
 0
 0
 0
 0
 0
 0
 0
 0
 0
 0
 6.32030812183787E-04

 5.09425414699041E-04
 0
 0
 0
 0
 0
 0
 0
 0
 0
 0
 0
 0
 0
 0
 0
 0
 0
 0
 0
 6.32030812183787E-04

 5.09425414699041E-04
 0
 0
 0
 0
 0
 0
 0
 0
 0
 0
 0
 0
 0
 0
 0
 0
 0
 0
 0
 6.32030812183787E-04

 0
 2.95075178030124E-04
 0
 0
 0
 0
 0
 0
 0
 0
 0
 0
 0
 0
 0
 0
 0
 0
 0
 0
 0
 1.66050938306959E-03

 0
 2.95075178030124E-04
 0
 0
 0
 0
 0
 0
 0
 0
 0
 0
 0
 0
 0
 0
 0
 0
 0
 0
 0
 1.66050938306959E-03

 0
 2.95075178030124E-04
 0
 0
 0
 0
 0
 0
 0
 0
 0
 0
 0
 0
 0
 0
 0
 0
 0
 0
 0
 1.66050938306959E-03

 0
 2.95075178030124E-04
 0
 0
 0
 0
 0
 0
 0
 0
 0
 0
 0
 0
 0
 0
 0
 0
 0
 0
 0
 1.66050938306959E-03

 0
 0
 7.3442412598966E-04
 0
 0
 0
 0
 0
 0
 0
 0
 0
 0
 0
 0
 0
 0
 0
 0
 0
 0
 0
 0

 0
 0
 7.3442412598966E-04
 0
 0
 0
 0
 0
 0
 0
 0
 0
 0
 0
 0
 0
 0
 0
 0
 0
 0
 0
 0

 0
 0
 7.3442412598966E-04
 0
 0
 0
 0
 0
 0
 0
 0
 0
 0
 0
 0
 0
 0
 0
 0
 0
 0
 0
 0

 0
 0
 7.3442412598966E-04
 0
 0
 0
 0
 0
 0
 0
 0
 0
 0
 0
 0
 0
 0
 0
 0
 0
 0
 0
 0

 0
 0
 0
 3.08187046916956E-04
 0
 0
 0
 0
 0
 0
 0
 0
 0
 0
 0
 0
 0
 0
 0
 0
 0
 0
 0
 7.88039896600808E-03

 0
 0
 0
 3.08187046916956E-04
 0
 0
 0
 0
 0
 0
 0
 0
 0
 0
 0
 0
 0
 0
 0
 0
 0
 0
 0
 7.88039896600808E-03

 0
 0
 0
 3.08187046916956E-04
 0
 0
 0
 0
 0
 0
 0
 0
 0
 0
 0
 0
 0
 0
 0
 0
 0
 0
 0
 7.88039896600808E-03

 0
 0
 0
 3.08187046916956E-04
 0
 0
 0
 0
 0
 0
 0
 0
 0
 0
 0
 0
 0
 0
 0
 0
 0
 0
 0
 7.88039896600808E-03

 0
 0
 0
 0
 2.06196667619329E-04
 0
 0
 0
 0
 0
 0
 0
 0
 0
 0
 0
 0
 0
 0
 0
 0
 0
 0
 0
 2.10560127371354E-03

 0
 0
 0
 0
 2.06196667619329E-04
 0
 0
 0
 0
 0
 0
 0
 0
 0
 0
 0
 0
 0
 0
 0
 0
 0
 0
 0
 2.10560127371354E-03

 0
 0
 0
 0
 2.06196667619329E-04
 0
 0
 0
 0
 0
 0
 0
 0
 0
 0
 0
 0
 0
 0
 0
 0
 0
 0
 0
 2.10560127371354E-03

 0
 0
 0
 0
 2.06196667619329E-04
 0
 0
 0
 0
 0
 0
 0
 0
 0
 0
 0
 0
 0
 0
 0
 0
 0
 0
 0
 2.10560127371354E-03

 0
 0
 0
 0
 0
 7.12146237899249E-04
 0
 0
 0
 0
 0
 0
 0
 0
 0
 0
 0
 0
 0
 0
 0
 0
 0
 0
 0
 0

 0
 0
 0
 0
 0
 7.12146237899249E-04
 0
 0
 0
 0
 0
 0
 0
 0
 0
 0
 0
 0
 0
 0
 0
 0
 0
 0
 0
 0

 0
 0
 0
 0
 0
 7.12146237899249E-04
 0
 0
 0
 0
 0
 0
 0
 0
 0
 0
 0
 0
 0
 0
 0
 0
 0
 0
 0
 0

 0
 0
 0
 0
 0
 7.12146237899249E-04
 0
 0
 0
 0
 0
 0
 0
 0
 0
 0
 0
 0
 0
 0
 0
 0
 0
 0
 0
 0

 0
 0
 0
 0
 0
 0
 0
 0
 0
 0
 0
 0
 0
 0
 0
 0
 0
 0
 0
 0
 0
 0
 0
 0
 0
 0
 6.37716154206369E-04

 0
 0
 0
 0
 0
 0
 0
 0
 0
 0
 0
 0
 0
 0
 0
 0
 0
 0
 0
 0
 0
 0
 0
 0
 0
 0
 6.37716154206369E-04

 0
 0
 0
 0
 0
 0
 0
 0
 0
 0
 0
 0
 0
 0
 0
 0
 0
 0
 0
 0
 0
 0
 0
 0
 0
 0
 6.37716154206369E-04

 0
 0
 0
 0
 0
 0
 0
 0
 0
 0
 0
 0
 0
 0
 0
 0
 0
 0
 0
 0
 0
 0
 0
 0
 0
 0
 6.37716154206369E-04

 0
 0
 0
 0
 0
 0
 0
 5.64942791699498E-04
 0
 0
 0
 0
 0
 0
 0
 0
 0
 0
 0
 0
 0
 0
 0
 0
 0
 0
 0
 0

 0
 0
 0
 0
 0
 0
 0
 5.64942791699498E-04
 0
 0
 0
 0
 0
 0
 0
 0
 0
 0
 0
 0
 0
 0
 0
 0
 0
 0
 0
 0

 0
 0
 0
 0
 0
 0
 0
 5.64942791699498E-04
 0
 0
 0
 0
 0
 0
 0
 0
 0
 0
 0
 0
 0
 0
 0
 0
 0
 0
 0
 0

 0
 0
 0
 0
 0
 0
 0
 5.64942791699498E-04
 0
 0
 0
 0
 0
 0
 0
 0
 0
 0
 0
 0
 0
 0
 0
 0
 0
 0
 0
 0

 0
 0
 0
 0
 0
 0
 0
 0
 1.75458635157921E-03
 0
 0
 0
 0
 0
 0
 0
 0
 0
 0
 0
 0
 0
 0
 0
 0
 0
 0
 0
 6.45208078429862E-03

 0
 0
 0
 0
 0
 0
 0
 0
 1.75458635157921E-03
 0
 0
 0
 0
 0
 0
 0
 0
 0
 0
 0
 0
 0
 0
 0
 0
 0
 0
 0
 6.45208078429862E-03

 0
 0
 0
 0
 0
 0
 0
 0
 1.75458635157921E-03
 0
 0
 0
 0
 0
 0
 0
 0
 0
 0
 0
 0
 0
 0
 0
 0
 0
 0
 0
 6.45208078429862E-03

 0
 0
 0
 0
 0
 0
 0
 0
 1.75458635157921E-03
 0
 0
 0
 0
 0
 0
 0
 0
 0
 0
 0
 0
 0
 0
 0
 0
 0
 0
 0
 6.45208078429862E-03

 0
 0
 0
 0
 0
 0
 0
 0
 0
 0
 0
 0
 0
 0
 0
 0
 0
 0
 0
 0
 0
 0
 0
 0
 0
 0
 0
 0
 0
 3.02484061097677E-03

 0
 0
 0
 0
 0
 0
 0
 0
 0
 0
 0
 0
 0
 0
 0
 0
 0
 0
 0
 0
 0
 0
 0
 0
 0
 0
 0
 0
 0
 3.02484061097677E-03

 0
 0
 0
 0
 0
 0
 0
 0
 0
 0
 0
 0
 0
 0
 0
 0
 0
 0
 0
 0
 0
 0
 0
 0
 0
 0
 0
 0
 0
 3.02484061097677E-03

 0
 0
 0
 0
 0
 0
 0
 0
 0
 0
 0
 0
 0
 0
 0
 0
 0
 0
 0
 0
 0
 0
 0
 0
 0
 0
 0
 0
 0
 3.02484061097677E-03

 0
 0
 0
 0
 0
 0
 0
 0
 0
 0
 1.00936141333735E-03
 0
 0
 0
 0
 0
 0
 0
 0
 0
 0
 0
 0
 0
 0
 0
 0
 0
 0
 0
 5.76861061995631E-04

 0
 0
 0
 0
 0
 0
 0
 0
 0
 0
 1.00936141333735E-03
 0
 0
 0
 0
 0
 0
 0
 0
 0
 0
 0
 0
 0
 0
 0
 0
 0
 0
 0
 5.76861061995631E-04

 0
 0
 0
 0
 0
 0
 0
 0
 0
 0
 1.00936141333735E-03
 0
 0
 0
 0
 0
 0
 0
 0
 0
 0
 0
 0
 0
 0
 0
 0
 0
 0
 0
 5.76861061995631E-04

 0
 0
 0
 0
 0
 0
 0
 0
 0
 0
 1.00936141333735E-03
 0
 0
 0
 0
 0
 0
 0
 0
 0
 0
 0
 0
 0
 0
 0
 0
 0
 0
 0
 5.76861061995631E-04

 0
 0
 0
 0
 0
 0
 0
 0
 0
 0
 0
 4.70747153736109E-03
 0
 0
 0
 0
 0
 0
 0
 0
 0
 0
 0
 0
 0
 0
 0
 0
 0
 0
 0
 6.27043402486206E-04

 0
 0
 0
 0
 0
 0
 0
 0
 0
 0
 0
 4.70747153736109E-03
 0
 0
 0
 0
 0
 0
 0
 0
 0
 0
 0
 0
 0
 0
 0
 0
 0
 0
 0
 6.27043402486206E-04

 0
 0
 0
 0
 0
 0
 0
 0
 0
 0
 0
 4.70747153736109E-03
 0
 0
 0
 0
 0
 0
 0
 0
 0
 0
 0
 0
 0
 0
 0
 0
 0
 0
 0
 6.27043402486206E-04

 0
 0
 0
 0
 0
 0
 0
 0
 0
 0
 0
 4.70747153736109E-03
 0
 0
 0
 0
 0
 0
 0
 0
 0
 0
 0
 0
 0
 0
 0
 0
 0
 0
 0
 6.27043402486206E-04

 0
 0
 0
 0
 0
 0
 0
 0
 0
 0
 0
 0
 3.01207970326544E-03

 0
 0
 0
 0
 0
 0
 0
 0
 0
 0
 0
 0
 3.01207970326544E-03

 0
 0
 0
 0
 0
 0
 0
 0
 0
 0
 0
 0
 3.01207970326544E-03

 0
 0
 0
 0
 0
 0
 0
 0
 0
 0
 0
 0
 3.01207970326544E-03

 0
 0
 0
 0
 0
 0
 0
 0
 0
 0
 0
 0
 0
 0

 0
 0
 0
 0
 0
 0
 0
 0
 0
 0
 0
 0
 0
 0

 0
 0
 0
 0
 0
 0
 0
 0
 0
 0
 0
 0
 0
 0

 0
 0
 0
 0
 0
 0
 0
 0
 0
 0
 0
 0
 0
 0

 0
 0
 0
 0
 0
 0
 0
 0
 0
 0
 0
 0
 0
 0
 1.52313271345035E-03

 0
 0
 0
 0
 0
 0
 0
 0
 0
 0
 0
 0
 0
 0
 1.52313271345035E-03

 0
 0
 0
 0
 0
 0
 0
 0
 0
 0
 0
 0
 0
 0
 1.52313271345035E-03

 0
 0
 0
 0
 0
 0
 0
 0
 0
 0
 0
 0
 0
 0
 1.52313271345035E-03

 0
 0
 0
 0
 0
 0
 0
 0
 0
 0
 0
 0
 0
 0
 0
 1.07473183036844E-03

 0
 0
 0
 0
 0
 0
 0
 0
 0
 0
 0
 0
 0
 0
 0
 1.07473183036844E-03

 0
 0
 0
 0
 0
 0
 0
 0
 0
 0
 0
 0
 0
 0
 0
 1.07473183036844E-03

 0
 0
 0
 0
 0
 0
 0
 0
 0
 0
 0
 0
 0
 0
 0
 1.07473183036844E-03

 0
 0
 0
 0
 0
 0
 0
 0
 0
 0
 0
 0
 0
 0
 0
 0
 2.66470874480547E-03

 0
 0
 0
 0
 0
 0
 0
 0
 0
 0
 0
 0
 0
 0
 0
 0
 2.66470874480547E-03

 0
 0
 0
 0
 0
 0
 0
 0
 0
 0
 0
 0
 0
 0
 0
 0
 2.66470874480547E-03

 0
 0
 0
 0
 0
 0
 0
 0
 0
 0
 0
 0
 0
 0
 0
 0
 2.66470874480547E-03

 0
 0
 0
 0
 0
 0
 0
 0
 0
 0
 0
 0
 0
 0
 0
 0
 0
 0

 0
 0
 0
 0
 0
 0
 0
 0
 0
 0
 0
 0
 0
 0
 0
 0
 0
 0

 0
 0
 0
 0
 0
 0
 0
 0
 0
 0
 0
 0
 0
 0
 0
 0
 0
 0

 0
 0
 0
 0
 0
 0
 0
 0
 0
 0
 0
 0
 0
 0
 0
 0
 0
 0

 0
 0
 0
 0
 0
 0
 0
 0
 0
 0
 0
 0
 0
 0
 0
 0
 0
 0
 0

 0
 0
 0
 0
 0
 0
 0
 0
 0
 0
 0
 0
 0
 0
 0
 0
 0
 0
 0

 0
 0
 0
 0
 0
 0
 0
 0
 0
 0
 0
 0
 0
 0
 0
 0
 0
 0
 0

 0
 0
 0
 0
 0
 0
 0
 0
 0
 0
 0
 0
 0
 0
 0
 0
 0
 0
 0

 0
 0
 0
 0
 0
 0
 0
 0
 0
 0
 0
 0
 0
 0
 0
 0
 0
 0
 0
 3.06916028158933E-04

 0
 0
 0
 0
 0
 0
 0
 0
 0
 0
 0
 0
 0
 0
 0
 0
 0
 0
 0
 3.06916028158933E-04

 0
 0
 0
 0
 0
 0
 0
 0
 0
 0
 0
 0
 0
 0
 0
 0
 0
 0
 0
 3.06916028158933E-04

 0
 0
 0
 0
 0
 0
 0
 0
 0
 0
 0
 0
 0
 0
 0
 0
 0
 0
 0
 3.06916028158933E-04

 0
 0
 0
 0
 0
 0
 0
 0
 0
 0
 0
 0
 0
 0
 0
 0
 0
 0
 0
 0
 0
 0
 0
 1.28056483197798E-03
 0
 0
 0
 0
 0
 0
 0
 0

 0
 0
 0
 0
 0
 0
 0
 0
 0
 0
 0
 0
 0
 0
 0
 0
 0
 0
 0
 0
 0
 0
 0
 1.28056483197798E-03
 0
 0
 0
 0
 0
 0
 0
 0

 0
 0
 0
 0
 0
 0
 0
 0
 0
 0
 0
 0
 0
 0
 0
 0
 0
 0
 0
 0
 0
 0
 0
 1.28056483197798E-03
 0
 0
 0
 0
 0
 0
 0
 0

 0
 0
 0
 0
 0
 0
 0
 0
 0
 0
 0
 0
 0
 0
 0
 0
 0
 0
 0
 0
 0
 0
 0
 1.28056483197798E-03
 0
 0
 0
 0
 0
 0
 0
 0

 0
 0
 0
 0
 0
 0
 0
 0
 0
 0
 0
 0
 0
 0
 0
 0
 0
 0
 0
 0
 0
 0
 0
 0
 0
 0
 0
 0
 0
 7.56210152745201E-04
 0
 0

 0
 0
 0
 0
 0
 0
 0
 0
 0
 0
 0
 0
 0
 0
 0
 0
 0
 0
 0
 0
 0
 0
 0
 0
 0
 0
 0
 0
 0
 7.56210152745201E-04
 0
 0

 0
 0
 0
 0
 0
 0
 0
 0
 0
 0
 0
 0
 0
 0
 0
 0
 0
 0
 0
 0
 0
 0
 0
 0
 0
 0
 0
 0
 0
 7.56210152745201E-04
 0
 0

 0
 0
 0
 0
 0
 0
 0
 0
 0
 0
 0
 0
 0
 0
 0
 0
 0
 0
 0
 0
 0
 0
 0
 0
 0
 0
 0
 0
 0
 7.56210152745201E-04
 0
 0

 2.36755461481218E-02
 .023015863886299
 7.06516009204196E-02
 .055689399377841
 5.13326604035531E-02
 1.66642219668144E-02
 3.21853770479627E-02
 4.73704530840387E-02
 6.47223040438262E-02
 8.72793765996025E-02
 .154179955887359
 6.78268190673725E-02
 .207231083584654
 3.10836970285424E-02
 .249819150550668
 .112470686048108
 .156077912758141
 .1982023420542
 .172731263000667
 .143636701177926
 4.47793830432184E-02
 1.88882942324301E-02
 4.44952372443924E-04
 .147264955677411
 4.28840792745808E-02
 1.43241747610011E-02
 1.01556297557432E-02
 5.19063727567327E-02
 7.33924189212062E-02
 2.60514397620473E-02
 1.01239116380378E-02
 1.16159790310787E-02

 0
 0
 0
 0
 0
 0
 0
 0
 0
 0
 0
 0
 0
 0
 0
 0
 0
 0
 0
 0
 0

 0
 0
 0
 0
 0
 0
 0
 0
 0
 0
 0
 0
 0
 0
 0
 0
 0
 0
 0
 0
 0

 0
 0
 0
 0
 0
 0
 0
 0
 0
 0
 0
 0
 0
 0
 0
 0
 0
 0
 0
 0
 0

 0
 0
 0
 0
 0
 0
 0
 0
 0
 0
 0
 0
 0
 0
 0
 0
 0
 0
 0
 0
 0

 .02317885636879
 0
 0
 0
 0
 0
 0
 0
 0
 0
 0
 0
 0
 0
 0
 0
 0
 0
 0
 0
 3.69738025127222E-03

 .02317885636879
 0
 0
 0
 0
 0
 0
 0
 0
 0
 0
 0
 0
 0
 0
 0
 0
 0
 0
 0
 3.69738025127222E-03

 .02317885636879
 0
 0
 0
 0
 0
 0
 0
 0
 0
 0
 0
 0
 0
 0
 0
 0
 0
 0
 0
 3.69738025127222E-03

 .02317885636879
 0
 0
 0
 0
 0
 0
 0
 0
 0
 0
 0
 0
 0
 0
 0
 0
 0
 0
 0
 3.69738025127222E-03

 4.96689779331838E-04
 0
 0
 0
 0
 0
 0
 0
 0
 0
 0
 0
 0
 0
 0
 0
 0
 0
 0
 0
 2.38275616193523E-02

 4.96689779331838E-04
 0
 0
 0
 0
 0
 0
 0
 0
 0
 0
 0
 0
 0
 0
 0
 0
 0
 0
 0
 2.38275616193523E-02

 4.96689779331838E-04
 0
 0
 0
 0
 0
 0
 0
 0
 0
 0
 0
 0
 0
 0
 0
 0
 0
 0
 0
 2.38275616193523E-02

 4.96689779331838E-04
 0
 0
 0
 0
 0
 0
 0
 0
 0
 0
 0
 0
 0
 0
 0
 0
 0
 0
 0
 2.38275616193523E-02

 0
 0
 0
 0
 0
 0
 0
 0
 0
 0
 0
 0
 0
 0
 0
 0
 0
 0
 0
 0
 0
 1.07933109899523E-03

 0
 0
 0
 0
 0
 0
 0
 0
 0
 0
 0
 0
 0
 0
 0
 0
 0
 0
 0
 0
 0
 1.07933109899523E-03

 0
 0
 0
 0
 0
 0
 0
 0
 0
 0
 0
 0
 0
 0
 0
 0
 0
 0
 0
 0
 0
 1.07933109899523E-03

 0
 0
 0
 0
 0
 0
 0
 0
 0
 0
 0
 0
 0
 0
 0
 0
 0
 0
 0
 0
 0
 1.07933109899523E-03

 0
 2.20568695577043E-02
 0
 0
 0
 0
 0
 0
 0
 0
 0
 0
 0
 0
 0
 0
 0
 0
 0
 0
 0
 1.78089631334348E-02

 0
 2.20568695577043E-02
 0
 0
 0
 0
 0
 0
 0
 0
 0
 0
 0
 0
 0
 0
 0
 0
 0
 0
 0
 1.78089631334348E-02

 0
 2.20568695577043E-02
 0
 0
 0
 0
 0
 0
 0
 0
 0
 0
 0
 0
 0
 0
 0
 0
 0
 0
 0
 1.78089631334348E-02

 0
 2.20568695577043E-02
 0
 0
 0
 0
 0
 0
 0
 0
 0
 0
 0
 0
 0
 0
 0
 0
 0
 0
 0
 1.78089631334348E-02

 0
 9.58994328594738E-04
 0
 0
 0
 0
 0
 0
 0
 0
 0
 0
 0
 0
 0
 0
 0
 0
 0
 0
 0
 0

 0
 9.58994328594738E-04
 0
 0
 0
 0
 0
 0
 0
 0
 0
 0
 0
 0
 0
 0
 0
 0
 0
 0
 0
 0

 0
 9.58994328594738E-04
 0
 0
 0
 0
 0
 0
 0
 0
 0
 0
 0
 0
 0
 0
 0
 0
 0
 0
 0
 0

 0
 9.58994328594738E-04
 0
 0
 0
 0
 0
 0
 0
 0
 0
 0
 0
 0
 0
 0
 0
 0
 0
 0
 0
 0

 0
 0
 0
 0
 0
 0
 0
 0
 0
 0
 0
 0
 0
 0
 0
 0
 0
 0
 0
 0
 0
 0
 0

 0
 0
 0
 0
 0
 0
 0
 0
 0
 0
 0
 0
 0
 0
 0
 0
 0
 0
 0
 0
 0
 0
 0

 0
 0
 0
 0
 0
 0
 0
 0
 0
 0
 0
 0
 0
 0
 0
 0
 0
 0
 0
 0
 0
 0
 0

 0
 0
 0
 0
 0
 0
 0
 0
 0
 0
 0
 0
 0
 0
 0
 0
 0
 0
 0
 0
 0
 0
 0

 0
 0
 6.93785991020361E-02
 0
 0
 0
 0
 0
 0
 0
 0
 0
 0
 0
 0
 0
 0
 0
 0
 0
 0
 0
 4.44952372443924E-04

 0
 0
 6.93785991020361E-02
 0
 0
 0
 0
 0
 0
 0
 0
 0
 0
 0
 0
 0
 0
 0
 0
 0
 0
 0
 4.44952372443924E-04

 0
 0
 6.93785991020361E-02
 0
 0
 0
 0
 0
 0
 0
 0
 0
 0
 0
 0
 0
 0
 0
 0
 0
 0
 0
 4.44952372443924E-04

 0
 0
 6.93785991020361E-02
 0
 0
 0
 0
 0
 0
 0
 0
 0
 0
 0
 0
 0
 0
 0
 0
 0
 0
 0
 4.44952372443924E-04

 0
 0
 1.27300181838348E-03
 0
 0
 0
 0
 0
 0
 0
 0
 0
 0
 0
 0
 0
 0
 0
 0
 0
 0
 0
 0

 0
 0
 1.27300181838348E-03
 0
 0
 0
 0
 0
 0
 0
 0
 0
 0
 0
 0
 0
 0
 0
 0
 0
 0
 0
 0

 0
 0
 1.27300181838348E-03
 0
 0
 0
 0
 0
 0
 0
 0
 0
 0
 0
 0
 0
 0
 0
 0
 0
 0
 0
 0

 0
 0
 1.27300181838348E-03
 0
 0
 0
 0
 0
 0
 0
 0
 0
 0
 0
 0
 0
 0
 0
 0
 0
 0
 0
 0

 0
 0
 0
 0
 0
 0
 0
 0
 0
 0
 0
 0
 0
 0
 0
 0
 0
 0
 0
 0
 0
 0
 0
 0

 0
 0
 0
 0
 0
 0
 0
 0
 0
 0
 0
 0
 0
 0
 0
 0
 0
 0
 0
 0
 0
 0
 0
 0

 0
 0
 0
 0
 0
 0
 0
 0
 0
 0
 0
 0
 0
 0
 0
 0
 0
 0
 0
 0
 0
 0
 0
 0

 0
 0
 0
 0
 0
 0
 0
 0
 0
 0
 0
 0
 0
 0
 0
 0
 0
 0
 0
 0
 0
 0
 0
 0

 0
 0
 0
 5.10820030264358E-02
 0
 0
 0
 0
 0
 0
 0
 0
 0
 0
 0
 0
 0
 0
 0
 0
 0
 0
 0
 .115250834878035

 0
 0
 0
 5.10820030264358E-02
 0
 0
 0
 0
 0
 0
 0
 0
 0
 0
 0
 0
 0
 0
 0
 0
 0
 0
 0
 .115250834878035

 0
 0
 0
 5.10820030264358E-02
 0
 0
 0
 0
 0
 0
 0
 0
 0
 0
 0
 0
 0
 0
 0
 0
 0
 0
 0
 .115250834878035

 0
 0
 0
 5.10820030264358E-02
 0
 0
 0
 0
 0
 0
 0
 0
 0
 0
 0
 0
 0
 0
 0
 0
 0
 0
 0
 .115250834878035

 0
 0
 0
 4.60739635140518E-03
 0
 0
 0
 0
 0
 0
 0
 0
 0
 0
 0
 0
 0
 0
 0
 0
 0
 0
 0
 1.28056483197798E-03

 0
 0
 0
 4.60739635140518E-03
 0
 0
 0
 0
 0
 0
 0
 0
 0
 0
 0
 0
 0
 0
 0
 0
 0
 0
 0
 1.28056483197798E-03

 0
 0
 0
 4.60739635140518E-03
 0
 0
 0
 0
 0
 0
 0
 0
 0
 0
 0
 0
 0
 0
 0
 0
 0
 0
 0
 1.28056483197798E-03

 0
 0
 0
 4.60739635140518E-03
 0
 0
 0
 0
 0
 0
 0
 0
 0
 0
 0
 0
 0
 0
 0
 0
 0
 0
 0
 1.28056483197798E-03

 0
 0
 0
 0
 1.34027833952564E-04
 0
 0
 0
 0
 0
 0
 0
 0
 0
 0
 0
 0
 0
 0
 0
 0
 0
 0
 0
 0

 0
 0
 0
 0
 1.34027833952564E-04
 0
 0
 0
 0
 0
 0
 0
 0
 0
 0
 0
 0
 0
 0
 0
 0
 0
 0
 0
 0

 0
 0
 0
 0
 1.34027833952564E-04
 0
 0
 0
 0
 0
 0
 0
 0
 0
 0
 0
 0
 0
 0
 0
 0
 0
 0
 0
 0

 0
 0
 0
 0
 1.34027833952564E-04
 0
 0
 0
 0
 0
 0
 0
 0
 0
 0
 0
 0
 0
 0
 0
 0
 0
 0
 0
 0

 0
 0
 0
 0
 4.57034913775742E-02
 0
 0
 0
 0
 0
 0
 0
 0
 0
 0
 0
 0
 0
 0
 0
 0
 0
 0
 0
 3.51284479163964E-02

 0
 0
 0
 0
 4.57034913775742E-02
 0
 0
 0
 0
 0
 0
 0
 0
 0
 0
 0
 0
 0
 0
 0
 0
 0
 0
 0
 3.51284479163964E-02

 0
 0
 0
 0
 4.57034913775742E-02
 0
 0
 0
 0
 0
 0
 0
 0
 0
 0
 0
 0
 0
 0
 0
 0
 0
 0
 0
 3.51284479163964E-02

 0
 0
 0
 0
 4.57034913775742E-02
 0
 0
 0
 0
 0
 0
 0
 0
 0
 0
 0
 0
 0
 0
 0
 0
 0
 0
 0
 3.51284479163964E-02

 0
 0
 0
 0
 5.49514119202634E-03
 0
 0
 0
 0
 0
 0
 0
 0
 0
 0
 0
 0
 0
 0
 0
 0
 0
 0
 0
 4.56213609304126E-04

 0
 0
 0
 0
 5.49514119202634E-03
 0
 0
 0
 0
 0
 0
 0
 0
 0
 0
 0
 0
 0
 0
 0
 0
 0
 0
 0
 4.56213609304126E-04

 0
 0
 0
 0
 5.49514119202634E-03
 0
 0
 0
 0
 0
 0
 0
 0
 0
 0
 0
 0
 0
 0
 0
 0
 0
 0
 0
 4.56213609304126E-04

 0
 0
 0
 0
 5.49514119202634E-03
 0
 0
 0
 0
 0
 0
 0
 0
 0
 0
 0
 0
 0
 0
 0
 0
 0
 0
 0
 4.56213609304126E-04

 0
 0
 0
 0
 0
 0
 0
 0
 0
 0
 0
 0
 0
 0
 0
 0
 0
 0
 0
 0
 0
 0
 0
 0
 0
 0

 0
 0
 0
 0
 0
 0
 0
 0
 0
 0
 0
 0
 0
 0
 0
 0
 0
 0
 0
 0
 0
 0
 0
 0
 0
 0

 0
 0
 0
 0
 0
 0
 0
 0
 0
 0
 0
 0
 0
 0
 0
 0
 0
 0
 0
 0
 0
 0
 0
 0
 0
 0

 0
 0
 0
 0
 0
 0
 0
 0
 0
 0
 0
 0
 0
 0
 0
 0
 0
 0
 0
 0
 0
 0
 0
 0
 0
 0

 0
 0
 0
 0
 0
 1.63556252637249E-02
 0
 0
 0
 0
 0
 0
 0
 0
 0
 0
 0
 0
 0
 0
 0
 0
 0
 0
 0
 1.17963792149434E-02

 0
 0
 0
 0
 0
 1.63556252637249E-02
 0
 0
 0
 0
 0
 0
 0
 0
 0
 0
 0
 0
 0
 0
 0
 0
 0
 0
 0
 1.17963792149434E-02

 0
 0
 0
 0
 0
 1.63556252637249E-02
 0
 0
 0
 0
 0
 0
 0
 0
 0
 0
 0
 0
 0
 0
 0
 0
 0
 0
 0
 1.17963792149434E-02

 0
 0
 0
 0
 0
 1.63556252637249E-02
 0
 0
 0
 0
 0
 0
 0
 0
 0
 0
 0
 0
 0
 0
 0
 0
 0
 0
 0
 1.17963792149434E-02

 0
 0
 0
 0
 0
 3.08596703089505E-04
 0
 0
 0
 0
 0
 0
 0
 0
 0
 0
 0
 0
 0
 0
 0
 0
 0
 0
 0
 1.4745474018671E-03

 0
 0
 0
 0
 0
 3.08596703089505E-04
 0
 0
 0
 0
 0
 0
 0
 0
 0
 0
 0
 0
 0
 0
 0
 0
 0
 0
 0
 1.4745474018671E-03

 0
 0
 0
 0
 0
 3.08596703089505E-04
 0
 0
 0
 0
 0
 0
 0
 0
 0
 0
 0
 0
 0
 0
 0
 0
 0
 0
 0
 1.4745474018671E-03

 0
 0
 0
 0
 0
 3.08596703089505E-04
 0
 0
 0
 0
 0
 0
 0
 0
 0
 0
 0
 0
 0
 0
 0
 0
 0
 0
 0
 1.4745474018671E-03

 0
 0
 0
 0
 0
 0
 0
 0
 0
 0
 0
 0
 0
 0
 0
 0
 0
 0
 0
 0
 0
 0
 0
 0
 0
 0
 1.03628875058631E-03

 0
 0
 0
 0
 0
 0
 0
 0
 0
 0
 0
 0
 0
 0
 0
 0
 0
 0
 0
 0
 0
 0
 0
 0
 0
 0
 1.03628875058631E-03

 0
 0
 0
 0
 0
 0
 0
 0
 0
 0
 0
 0
 0
 0
 0
 0
 0
 0
 0
 0
 0
 0
 0
 0
 0
 0
 1.03628875058631E-03

 0
 0
 0
 0
 0
 0
 0
 0
 0
 0
 0
 0
 0
 0
 0
 0
 0
 0
 0
 0
 0
 0
 0
 0
 0
 0
 1.03628875058631E-03

 0
 0
 0
 0
 0
 0
 3.03329812466404E-02
 0
 0
 0
 0
 0
 0
 0
 0
 0
 0
 0
 0
 0
 0
 0
 0
 0
 0
 0
 7.25402125410226E-03

 0
 0
 0
 0
 0
 0
 3.03329812466404E-02
 0
 0
 0
 0
 0
 0
 0
 0
 0
 0
 0
 0
 0
 0
 0
 0
 0
 0
 0
 7.25402125410226E-03

 0
 0
 0
 0
 0
 0
 3.03329812466404E-02
 0
 0
 0
 0
 0
 0
 0
 0
 0
 0
 0
 0
 0
 0
 0
 0
 0
 0
 0
 7.25402125410226E-03

 0
 0
 0
 0
 0
 0
 3.03329812466404E-02
 0
 0
 0
 0
 0
 0
 0
 0
 0
 0
 0
 0
 0
 0
 0
 0
 0
 0
 0
 7.25402125410226E-03

 0
 0
 0
 0
 0
 0
 1.85239580132225E-03
 0
 0
 0
 0
 0
 0
 0
 0
 0
 0
 0
 0
 0
 0
 0
 0
 0
 0
 0
 1.03628875058631E-03

 0
 0
 0
 0
 0
 0
 1.85239580132225E-03
 0
 0
 0
 0
 0
 0
 0
 0
 0
 0
 0
 0
 0
 0
 0
 0
 0
 0
 0
 1.03628875058631E-03

 0
 0
 0
 0
 0
 0
 1.85239580132225E-03
 0
 0
 0
 0
 0
 0
 0
 0
 0
 0
 0
 0
 0
 0
 0
 0
 0
 0
 0
 1.03628875058631E-03

 0
 0
 0
 0
 0
 0
 1.85239580132225E-03
 0
 0
 0
 0
 0
 0
 0
 0
 0
 0
 0
 0
 0
 0
 0
 0
 0
 0
 0
 1.03628875058631E-03

 0
 0
 0
 0
 0
 0
 0
 0
 0
 0
 0
 0
 0
 0
 0
 0
 0
 0
 0
 0
 0
 0
 0
 0
 0
 0
 0
 2.66186526957187E-03

 0
 0
 0
 0
 0
 0
 0
 0
 0
 0
 0
 0
 0
 0
 0
 0
 0
 0
 0
 0
 0
 0
 0
 0
 0
 0
 0
 2.66186526957187E-03

 0
 0
 0
 0
 0
 0
 0
 0
 0
 0
 0
 0
 0
 0
 0
 0
 0
 0
 0
 0
 0
 0
 0
 0
 0
 0
 0
 2.66186526957187E-03

 0
 0
 0
 0
 0
 0
 0
 0
 0
 0
 0
 0
 0
 0
 0
 0
 0
 0
 0
 0
 0
 0
 0
 0
 0
 0
 0
 2.66186526957187E-03

 0
 0
 0
 0
 0
 0
 0
 1.61573638426202E-02
 0
 0
 0
 0
 0
 0
 0
 0
 0
 0
 0
 0
 0
 0
 0
 0
 0
 0
 0
 4.42535101067135E-02

 0
 0
 0
 0
 0
 0
 0
 1.61573638426202E-02
 0
 0
 0
 0
 0
 0
 0
 0
 0
 0
 0
 0
 0
 0
 0
 0
 0
 0
 0
 4.42535101067135E-02

 0
 0
 0
 0
 0
 0
 0
 1.61573638426202E-02
 0
 0
 0
 0
 0
 0
 0
 0
 0
 0
 0
 0
 0
 0
 0
 0
 0
 0
 0
 4.42535101067135E-02

 0
 0
 0
 0
 0
 0
 0
 1.61573638426202E-02
 0
 0
 0
 0
 0
 0
 0
 0
 0
 0
 0
 0
 0
 0
 0
 0
 0
 0
 0
 4.42535101067135E-02

 0
 0
 0
 0
 0
 0
 0
 3.12130892414185E-02
 0
 0
 0
 0
 0
 0
 0
 0
 0
 0
 0
 0
 0
 0
 0
 0
 0
 0
 0
 4.65826422175077E-03

 0
 0
 0
 0
 0
 0
 0
 3.12130892414185E-02
 0
 0
 0
 0
 0
 0
 0
 0
 0
 0
 0
 0
 0
 0
 0
 0
 0
 0
 0
 4.65826422175077E-03

 0
 0
 0
 0
 0
 0
 0
 3.12130892414185E-02
 0
 0
 0
 0
 0
 0
 0
 0
 0
 0
 0
 0
 0
 0
 0
 0
 0
 0
 0
 4.65826422175077E-03

 0
 0
 0
 0
 0
 0
 0
 3.12130892414185E-02
 0
 0
 0
 0
 0
 0
 0
 0
 0
 0
 0
 0
 0
 0
 0
 0
 0
 0
 0
 4.65826422175077E-03

 0
 0
 0
 0
 0
 0
 0
 0
 0
 0
 0
 0
 0
 0
 0
 0
 0
 0
 0
 0
 0
 0
 0
 0
 0
 0
 0
 0
 0

 0
 0
 0
 0
 0
 0
 0
 0
 0
 0
 0
 0
 0
 0
 0
 0
 0
 0
 0
 0
 0
 0
 0
 0
 0
 0
 0
 0
 0

 0
 0
 0
 0
 0
 0
 0
 0
 0
 0
 0
 0
 0
 0
 0
 0
 0
 0
 0
 0
 0
 0
 0
 0
 0
 0
 0
 0
 0

 0
 0
 0
 0
 0
 0
 0
 0
 0
 0
 0
 0
 0
 0
 0
 0
 0
 0
 0
 0
 0
 0
 0
 0
 0
 0
 0
 0
 0

 0
 0
 0
 0
 0
 0
 0
 0
 3.23611520219131E-02
 0
 0
 0
 0
 0
 0
 0
 0
 0
 0
 0
 0
 0
 0
 0
 0
 0
 0
 0
 5.17241809540123E-02

 0
 0
 0
 0
 0
 0
 0
 0
 3.23611520219131E-02
 0
 0
 0
 0
 0
 0
 0
 0
 0
 0
 0
 0
 0
 0
 0
 0
 0
 0
 0
 5.17241809540123E-02

 0
 0
 0
 0
 0
 0
 0
 0
 3.23611520219131E-02
 0
 0
 0
 0
 0
 0
 0
 0
 0
 0
 0
 0
 0
 0
 0
 0
 0
 0
 0
 5.17241809540123E-02

 0
 0
 0
 0
 0
 0
 0
 0
 3.23611520219131E-02
 0
 0
 0
 0
 0
 0
 0
 0
 0
 0
 0
 0
 0
 0
 0
 0
 0
 0
 0
 5.17241809540123E-02

 0
 0
 0
 0
 0
 0
 0
 0
 3.23611520219131E-02
 0
 0
 0
 0
 0
 0
 0
 0
 0
 0
 0
 0
 0
 0
 0
 0
 0
 0
 0
 1.39795083659639E-03

 0
 0
 0
 0
 0
 0
 0
 0
 3.23611520219131E-02
 0
 0
 0
 0
 0
 0
 0
 0
 0
 0
 0
 0
 0
 0
 0
 0
 0
 0
 0
 1.39795083659639E-03

 0
 0
 0
 0
 0
 0
 0
 0
 3.23611520219131E-02
 0
 0
 0
 0
 0
 0
 0
 0
 0
 0
 0
 0
 0
 0
 0
 0
 0
 0
 0
 1.39795083659639E-03

 0
 0
 0
 0
 0
 0
 0
 0
 3.23611520219131E-02
 0
 0
 0
 0
 0
 0
 0
 0
 0
 0
 0
 0
 0
 0
 0
 0
 0
 0
 0
 1.39795083659639E-03

 0
 0
 0
 0
 0
 0
 0
 0
 0
 0
 0
 0
 0
 0
 0
 0
 0
 0
 0
 0
 0
 0
 0
 0
 0
 0
 0
 0
 0
 0

 0
 0
 0
 0
 0
 0
 0
 0
 0
 0
 0
 0
 0
 0
 0
 0
 0
 0
 0
 0
 0
 0
 0
 0
 0
 0
 0
 0
 0
 0

 0
 0
 0
 0
 0
 0
 0
 0
 0
 0
 0
 0
 0
 0
 0
 0
 0
 0
 0
 0
 0
 0
 0
 0
 0
 0
 0
 0
 0
 0

 0
 0
 0
 0
 0
 0
 0
 0
 0
 0
 0
 0
 0
 0
 0
 0
 0
 0
 0
 0
 0
 0
 0
 0
 0
 0
 0
 0
 0
 0

 0
 0
 0
 0
 0
 0
 0
 0
 0
 7.19287324739992E-02
 0
 0
 0
 0
 0
 0
 0
 0
 0
 0
 0
 0
 0
 0
 0
 0
 0
 0
 0
 2.31022201663384E-02

 0
 0
 0
 0
 0
 0
 0
 0
 0
 7.19287324739992E-02
 0
 0
 0
 0
 0
 0
 0
 0
 0
 0
 0
 0
 0
 0
 0
 0
 0
 0
 0
 2.31022201663384E-02

 0
 0
 0
 0
 0
 0
 0
 0
 0
 7.19287324739992E-02
 0
 0
 0
 0
 0
 0
 0
 0
 0
 0
 0
 0
 0
 0
 0
 0
 0
 0
 0
 2.31022201663384E-02

 0
 0
 0
 0
 0
 0
 0
 0
 0
 7.19287324739992E-02
 0
 0
 0
 0
 0
 0
 0
 0
 0
 0
 0
 0
 0
 0
 0
 0
 0
 0
 0
 2.31022201663384E-02

 0
 0
 0
 0
 0
 0
 0
 0
 0
 1.53506441256033E-02
 0
 0
 0
 0
 0
 0
 0
 0
 0
 0
 0
 0
 0
 0
 0
 0
 0
 0
 0
 0

 0
 0
 0
 0
 0
 0
 0
 0
 0
 1.53506441256033E-02
 0
 0
 0
 0
 0
 0
 0
 0
 0
 0
 0
 0
 0
 0
 0
 0
 0
 0
 0
 0

 0
 0
 0
 0
 0
 0
 0
 0
 0
 1.53506441256033E-02
 0
 0
 0
 0
 0
 0
 0
 0
 0
 0
 0
 0
 0
 0
 0
 0
 0
 0
 0
 0

 0
 0
 0
 0
 0
 0
 0
 0
 0
 1.53506441256033E-02
 0
 0
 0
 0
 0
 0
 0
 0
 0
 0
 0
 0
 0
 0
 0
 0
 0
 0
 0
 0

 0
 0
 0
 0
 0
 0
 0
 0
 0
 0
 0
 0
 0
 0
 0
 0
 0
 0
 0
 0
 0
 0
 0
 0
 0
 0
 0
 0
 0
 0
 0

 0
 0
 0
 0
 0
 0
 0
 0
 0
 0
 0
 0
 0
 0
 0
 0
 0
 0
 0
 0
 0
 0
 0
 0
 0
 0
 0
 0
 0
 0
 0

 0
 0
 0
 0
 0
 0
 0
 0
 0
 0
 0
 0
 0
 0
 0
 0
 0
 0
 0
 0
 0
 0
 0
 0
 0
 0
 0
 0
 0
 0
 0

 0
 0
 0
 0
 0
 0
 0
 0
 0
 0
 0
 0
 0
 0
 0
 0
 0
 0
 0
 0
 0
 0
 0
 0
 0
 0
 0
 0
 0
 0
 0

 0
 0
 0
 0
 0
 0
 0
 0
 0
 0
 3.60846705268319E-02
 0
 0
 0
 0
 0
 0
 0
 0
 0
 0
 0
 0
 0
 0
 0
 0
 0
 0
 0
 5.99935504476543E-03

 0
 0
 0
 0
 0
 0
 0
 0
 0
 0
 3.60846705268319E-02
 0
 0
 0
 0
 0
 0
 0
 0
 0
 0
 0
 0
 0
 0
 0
 0
 0
 0
 0
 5.99935504476543E-03

 0
 0
 0
 0
 0
 0
 0
 0
 0
 0
 3.60846705268319E-02
 0
 0
 0
 0
 0
 0
 0
 0
 0
 0
 0
 0
 0
 0
 0
 0
 0
 0
 0
 5.99935504476543E-03

 0
 0
 0
 0
 0
 0
 0
 0
 0
 0
 3.60846705268319E-02
 0
 0
 0
 0
 0
 0
 0
 0
 0
 0
 0
 0
 0
 0
 0
 0
 0
 0
 0
 5.99935504476543E-03

 0
 0
 0
 0
 0
 0
 0
 0
 0
 0
 .118095285360528
 0
 0
 0
 0
 0
 0
 0
 0
 0
 0
 0
 0
 0
 0
 0
 0
 0
 0
 0
 2.62471783208374E-03

 0
 0
 0
 0
 0
 0
 0
 0
 0
 0
 .118095285360528
 0
 0
 0
 0
 0
 0
 0
 0
 0
 0
 0
 0
 0
 0
 0
 0
 0
 0
 0
 2.62471783208374E-03

 0
 0
 0
 0
 0
 0
 0
 0
 0
 0
 .118095285360528
 0
 0
 0
 0
 0
 0
 0
 0
 0
 0
 0
 0
 0
 0
 0
 0
 0
 0
 0
 2.62471783208374E-03

 0
 0
 0
 0
 0
 0
 0
 0
 0
 0
 .118095285360528
 0
 0
 0
 0
 0
 0
 0
 0
 0
 0
 0
 0
 0
 0
 0
 0
 0
 0
 0
 2.62471783208374E-03

 0
 0
 0
 0
 0
 0
 0
 0
 0
 0
 0
 2.54988041607059E-04
 0
 0
 0
 0
 0
 0
 0
 0
 0
 0
 0
 0
 0
 0
 0
 0
 0
 0
 0
 0

 0
 0
 0
 0
 0
 0
 0
 0
 0
 0
 0
 2.54988041607059E-04
 0
 0
 0
 0
 0
 0
 0
 0
 0
 0
 0
 0
 0
 0
 0
 0
 0
 0
 0
 0

 0
 0
 0
 0
 0
 0
 0
 0
 0
 0
 0
 2.54988041607059E-04
 0
 0
 0
 0
 0
 0
 0
 0
 0
 0
 0
 0
 0
 0
 0
 0
 0
 0
 0
 0

 0
 0
 0
 0
 0
 0
 0
 0
 0
 0
 0
 2.54988041607059E-04
 0
 0
 0
 0
 0
 0
 0
 0
 0
 0
 0
 0
 0
 0
 0
 0
 0
 0
 0
 0

 0
 0
 0
 0
 0
 0
 0
 0
 0
 0
 0
 2.60087802438948E-02
 0
 0
 0
 0
 0
 0
 0
 0
 0
 0
 0
 0
 0
 0
 0
 0
 0
 0
 0
 1.10046117136537E-02

 0
 0
 0
 0
 0
 0
 0
 0
 0
 0
 0
 2.60087802438948E-02
 0
 0
 0
 0
 0
 0
 0
 0
 0
 0
 0
 0
 0
 0
 0
 0
 0
 0
 0
 1.10046117136537E-02

 0
 0
 0
 0
 0
 0
 0
 0
 0
 0
 0
 2.60087802438948E-02
 0
 0
 0
 0
 0
 0
 0
 0
 0
 0
 0
 0
 0
 0
 0
 0
 0
 0
 0
 1.10046117136537E-02

 0
 0
 0
 0
 0
 0
 0
 0
 0
 0
 0
 2.60087802438948E-02
 0
 0
 0
 0
 0
 0
 0
 0
 0
 0
 0
 0
 0
 0
 0
 0
 0
 0
 0
 1.10046117136537E-02

 0
 0
 0
 0
 0
 0
 0
 0
 0
 0
 0
 4.15630507818707E-02
 0
 0
 0
 0
 0
 0
 0
 0
 0
 0
 0
 0
 0
 0
 0
 0
 0
 0
 0
 2.03789105808962E-04

 0
 0
 0
 0
 0
 0
 0
 0
 0
 0
 0
 4.15630507818707E-02
 0
 0
 0
 0
 0
 0
 0
 0
 0
 0
 0
 0
 0
 0
 0
 0
 0
 0
 0
 2.03789105808962E-04

 0
 0
 0
 0
 0
 0
 0
 0
 0
 0
 0
 4.15630507818707E-02
 0
 0
 0
 0
 0
 0
 0
 0
 0
 0
 0
 0
 0
 0
 0
 0
 0
 0
 0
 2.03789105808962E-04

 0
 0
 0
 0
 0
 0
 0
 0
 0
 0
 0
 4.15630507818707E-02
 0
 0
 0
 0
 0
 0
 0
 0
 0
 0
 0
 0
 0
 0
 0
 0
 0
 0
 0
 2.03789105808962E-04

 0
 0
 0
 0
 0
 0
 0
 0
 0
 0
 0
 0
 1.50603985163463E-04

 0
 0
 0
 0
 0
 0
 0
 0
 0
 0
 0
 0
 1.50603985163463E-04

 0
 0
 0
 0
 0
 0
 0
 0
 0
 0
 0
 0
 1.50603985163463E-04

 0
 0
 0
 0
 0
 0
 0
 0
 0
 0
 0
 0
 1.50603985163463E-04

 0
 0
 0
 0
 0
 0
 0
 0
 0
 0
 0
 0
 2.86147571810332E-03

 0
 0
 0
 0
 0
 0
 0
 0
 0
 0
 0
 0
 2.86147571810332E-03

 0
 0
 0
 0
 0
 0
 0
 0
 0
 0
 0
 0
 2.86147571810332E-03

 0
 0
 0
 0
 0
 0
 0
 0
 0
 0
 0
 0
 2.86147571810332E-03

 0
 0
 0
 0
 0
 0
 0
 0
 0
 0
 0
 0
 .204219003881388

 0
 0
 0
 0
 0
 0
 0
 0
 0
 0
 0
 0
 .204219003881388

 0
 0
 0
 0
 0
 0
 0
 0
 0
 0
 0
 0
 .204219003881388

 0
 0
 0
 0
 0
 0
 0
 0
 0
 0
 0
 0
 .204219003881388

 0
 0
 0
 0
 0
 0
 0
 0
 0
 0
 0
 0
 0
 0

 0
 0
 0
 0
 0
 0
 0
 0
 0
 0
 0
 0
 0
 0

 0
 0
 0
 0
 0
 0
 0
 0
 0
 0
 0
 0
 0
 0

 0
 0
 0
 0
 0
 0
 0
 0
 0
 0
 0
 0
 0
 0

 0
 0
 0
 0
 0
 0
 0
 0
 0
 0
 0
 0
 0
 1.57089651649267E-02

 0
 0
 0
 0
 0
 0
 0
 0
 0
 0
 0
 0
 0
 1.57089651649267E-02

 0
 0
 0
 0
 0
 0
 0
 0
 0
 0
 0
 0
 0
 1.57089651649267E-02

 0
 0
 0
 0
 0
 0
 0
 0
 0
 0
 0
 0
 0
 1.57089651649267E-02

 0
 0
 0
 0
 0
 0
 0
 0
 0
 0
 0
 0
 0
 1.53747318636157E-02

 0
 0
 0
 0
 0
 0
 0
 0
 0
 0
 0
 0
 0
 1.53747318636157E-02

 0
 0
 0
 0
 0
 0
 0
 0
 0
 0
 0
 0
 0
 1.53747318636157E-02

 0
 0
 0
 0
 0
 0
 0
 0
 0
 0
 0
 0
 0
 1.53747318636157E-02

 0
 0
 0
 0
 0
 0
 0
 0
 0
 0
 0
 0
 0
 0
 0

 0
 0
 0
 0
 0
 0
 0
 0
 0
 0
 0
 0
 0
 0
 0

 0
 0
 0
 0
 0
 0
 0
 0
 0
 0
 0
 0
 0
 0
 0

 0
 0
 0
 0
 0
 0
 0
 0
 0
 0
 0
 0
 0
 0
 0

 0
 0
 0
 0
 0
 0
 0
 0
 0
 0
 0
 0
 0
 0
 1.15504230769713E-03

 0
 0
 0
 0
 0
 0
 0
 0
 0
 0
 0
 0
 0
 0
 1.15504230769713E-03

 0
 0
 0
 0
 0
 0
 0
 0
 0
 0
 0
 0
 0
 0
 1.15504230769713E-03

 0
 0
 0
 0
 0
 0
 0
 0
 0
 0
 0
 0
 0
 0
 1.15504230769713E-03

 0
 0
 0
 0
 0
 0
 0
 0
 0
 0
 0
 0
 0
 0
 .248664108242971

 0
 0
 0
 0
 0
 0
 0
 0
 0
 0
 0
 0
 0
 0
 .248664108242971

 0
 0
 0
 0
 0
 0
 0
 0
 0
 0
 0
 0
 0
 0
 .248664108242971

 0
 0
 0
 0
 0
 0
 0
 0
 0
 0
 0
 0
 0
 0
 .248664108242971

 0
 0
 0
 0
 0
 0
 0
 0
 0
 0
 0
 0
 0
 0
 0
 0

 0
 0
 0
 0
 0
 0
 0
 0
 0
 0
 0
 0
 0
 0
 0
 0

 0
 0
 0
 0
 0
 0
 0
 0
 0
 0
 0
 0
 0
 0
 0
 0

 0
 0
 0
 0
 0
 0
 0
 0
 0
 0
 0
 0
 0
 0
 0
 0

 0
 0
 0
 0
 0
 0
 0
 0
 0
 0
 0
 0
 0
 0
 0
 8.26647899525394E-02

 0
 0
 0
 0
 0
 0
 0
 0
 0
 0
 0
 0
 0
 0
 0
 8.26647899525394E-02

 0
 0
 0
 0
 0
 0
 0
 0
 0
 0
 0
 0
 0
 0
 0
 8.26647899525394E-02

 0
 0
 0
 0
 0
 0
 0
 0
 0
 0
 0
 0
 0
 0
 0
 8.26647899525394E-02

 0
 0
 0
 0
 0
 0
 0
 0
 0
 0
 0
 0
 0
 0
 0
 2.98058960955689E-02

 0
 0
 0
 0
 0
 0
 0
 0
 0
 0
 0
 0
 0
 0
 0
 2.98058960955689E-02

 0
 0
 0
 0
 0
 0
 0
 0
 0
 0
 0
 0
 0
 0
 0
 2.98058960955689E-02

 0
 0
 0
 0
 0
 0
 0
 0
 0
 0
 0
 0
 0
 0
 0
 2.98058960955689E-02

 0
 0
 0
 0
 0
 0
 0
 0
 0
 0
 0
 0
 0
 0
 0
 0
 0

 0
 0
 0
 0
 0
 0
 0
 0
 0
 0
 0
 0
 0
 0
 0
 0
 0

 0
 0
 0
 0
 0
 0
 0
 0
 0
 0
 0
 0
 0
 0
 0
 0
 0

 0
 0
 0
 0
 0
 0
 0
 0
 0
 0
 0
 0
 0
 0
 0
 0
 0

 0
 0
 0
 0
 0
 0
 0
 0
 0
 0
 0
 0
 0
 0
 0
 0
 2.69431661974635E-03

 0
 0
 0
 0
 0
 0
 0
 0
 0
 0
 0
 0
 0
 0
 0
 0
 2.69431661974635E-03

 0
 0
 0
 0
 0
 0
 0
 0
 0
 0
 0
 0
 0
 0
 0
 0
 2.69431661974635E-03

 0
 0
 0
 0
 0
 0
 0
 0
 0
 0
 0
 0
 0
 0
 0
 0
 2.69431661974635E-03

 0
 0
 0
 0
 0
 0
 0
 0
 0
 0
 0
 0
 0
 0
 0
 0
 .153383596138394

 0
 0
 0
 0
 0
 0
 0
 0
 0
 0
 0
 0
 0
 0
 0
 0
 .153383596138394

 0
 0
 0
 0
 0
 0
 0
 0
 0
 0
 0
 0
 0
 0
 0
 0
 .153383596138394

 0
 0
 0
 0
 0
 0
 0
 0
 0
 0
 0
 0
 0
 0
 0
 0
 .153383596138394

 0
 0
 0
 0
 0
 0
 0
 0
 0
 0
 0
 0
 0
 0
 0
 0
 0
 0

 0
 0
 0
 0
 0
 0
 0
 0
 0
 0
 0
 0
 0
 0
 0
 0
 0
 0

 0
 0
 0
 0
 0
 0
 0
 0
 0
 0
 0
 0
 0
 0
 0
 0
 0
 0

 0
 0
 0
 0
 0
 0
 0
 0
 0
 0
 0
 0
 0
 0
 0
 0
 0
 0

 0
 0
 0
 0
 0
 0
 0
 0
 0
 0
 0
 0
 0
 0
 0
 0
 0
 .184925630146291

 0
 0
 0
 0
 0
 0
 0
 0
 0
 0
 0
 0
 0
 0
 0
 0
 0
 .184925630146291

 0
 0
 0
 0
 0
 0
 0
 0
 0
 0
 0
 0
 0
 0
 0
 0
 0
 .184925630146291

 0
 0
 0
 0
 0
 0
 0
 0
 0
 0
 0
 0
 0
 0
 0
 0
 0
 .184925630146291

 0
 0
 0
 0
 0
 0
 0
 0
 0
 0
 0
 0
 0
 0
 0
 0
 0
 1.32767119079094E-02

 0
 0
 0
 0
 0
 0
 0
 0
 0
 0
 0
 0
 0
 0
 0
 0
 0
 1.32767119079094E-02

 0
 0
 0
 0
 0
 0
 0
 0
 0
 0
 0
 0
 0
 0
 0
 0
 0
 1.32767119079094E-02

 0
 0
 0
 0
 0
 0
 0
 0
 0
 0
 0
 0
 0
 0
 0
 0
 0
 1.32767119079094E-02

 0
 0
 0
 0
 0
 0
 0
 0
 0
 0
 0
 0
 0
 0
 0
 0
 0
 0
 0

 0
 0
 0
 0
 0
 0
 0
 0
 0
 0
 0
 0
 0
 0
 0
 0
 0
 0
 0

 0
 0
 0
 0
 0
 0
 0
 0
 0
 0
 0
 0
 0
 0
 0
 0
 0
 0
 0

 0
 0
 0
 0
 0
 0
 0
 0
 0
 0
 0
 0
 0
 0
 0
 0
 0
 0
 0

 0
 0
 0
 0
 0
 0
 0
 0
 0
 0
 0
 0
 0
 0
 0
 0
 0
 0
 2.27610741875003E-03

 0
 0
 0
 0
 0
 0
 0
 0
 0
 0
 0
 0
 0
 0
 0
 0
 0
 0
 2.27610741875003E-03

 0
 0
 0
 0
 0
 0
 0
 0
 0
 0
 0
 0
 0
 0
 0
 0
 0
 0
 2.27610741875003E-03

 0
 0
 0
 0
 0
 0
 0
 0
 0
 0
 0
 0
 0
 0
 0
 0
 0
 0
 2.27610741875003E-03

 0
 0
 0
 0
 0
 0
 0
 0
 0
 0
 0
 0
 0
 0
 0
 0
 0
 0
 .170455155581917

 0
 0
 0
 0
 0
 0
 0
 0
 0
 0
 0
 0
 0
 0
 0
 0
 0
 0
 .170455155581917

 0
 0
 0
 0
 0
 0
 0
 0
 0
 0
 0
 0
 0
 0
 0
 0
 0
 0
 .170455155581917

 0
 0
 0
 0
 0
 0
 0
 0
 0
 0
 0
 0
 0
 0
 0
 0
 0
 0
 .170455155581917

 0
 0
 0
 0
 0
 0
 0
 0
 0
 0
 0
 0
 0
 0
 0
 0
 0
 0
 0
 0

 0
 0
 0
 0
 0
 0
 0
 0
 0
 0
 0
 0
 0
 0
 0
 0
 0
 0
 0
 0

 0
 0
 0
 0
 0
 0
 0
 0
 0
 0
 0
 0
 0
 0
 0
 0
 0
 0
 0
 0

 0
 0
 0
 0
 0
 0
 0
 0
 0
 0
 0
 0
 0
 0
 0
 0
 0
 0
 0
 0

 0
 0
 0
 0
 0
 0
 0
 0
 0
 0
 0
 0
 0
 0
 0
 0
 0
 0
 0
 2.19444960133308E-03

 0
 0
 0
 0
 0
 0
 0
 0
 0
 0
 0
 0
 0
 0
 0
 0
 0
 0
 0
 2.19444960133308E-03

 0
 0
 0
 0
 0
 0
 0
 0
 0
 0
 0
 0
 0
 0
 0
 0
 0
 0
 0
 2.19444960133308E-03

 0
 0
 0
 0
 0
 0
 0
 0
 0
 0
 0
 0
 0
 0
 0
 0
 0
 0
 0
 2.19444960133308E-03

 0
 0
 0
 0
 0
 0
 0
 0
 0
 0
 0
 0
 0
 0
 0
 0
 0
 0
 0
 .141442251576593

 0
 0
 0
 0
 0
 0
 0
 0
 0
 0
 0
 0
 0
 0
 0
 0
 0
 0
 0
 .141442251576593

 0
 0
 0
 0
 0
 0
 0
 0
 0
 0
 0
 0
 0
 0
 0
 0
 0
 0
 0
 .141442251576593

 0
 0
 0
 0
 0
 0
 0
 0
 0
 0
 0
 0
 0
 0
 0
 0
 0
 0
 0
 .141442251576593

 0
 0
 0
 0
 0
 0
 0
 0
 0
 0
 0
 0
 0
 0
 0
 0
 0
 0
 0
 0
 1.72544411725939E-02
 0
 0
 3.07335559673982E-02
 7.29941774888027E-03
 1.05324814419055E-03
 8.2903100046828E-04
 3.32733158696484E-04
 2.02702871305976E-02
 2.94921959570891E-03
 1.49983876118864E-03
 4.07578211616034E-04

 0
 0
 0
 0
 0
 0
 0
 0
 0
 0
 0
 0
 0
 0
 0
 0
 0
 0
 0
 0
 1.72544411725939E-02
 0
 0
 3.07335559673982E-02
 7.29941774888027E-03
 1.05324814419055E-03
 8.2903100046828E-04
 3.32733158696484E-04
 2.02702871305976E-02
 2.94921959570891E-03
 1.49983876118864E-03
 4.07578211616034E-04

 0
 0
 0
 0
 0
 0
 0
 0
 0
 0
 0
 0
 0
 0
 0
 0
 0
 0
 0
 0
 1.72544411725939E-02
 0
 0
 3.07335559673982E-02
 7.29941774888027E-03
 1.05324814419055E-03
 8.2903100046828E-04
 3.32733158696484E-04
 2.02702871305976E-02
 2.94921959570891E-03
 1.49983876118864E-03
 4.07578211616034E-04

 0
 0
 0
 0
 0
 0
 0
 0
 0
 0
 0
 0
 0
 0
 0
 0
 0
 0
 0
 0
 1.72544411725939E-02
 0
 0
 3.07335559673982E-02
 7.29941774888027E-03
 1.05324814419055E-03
 8.2903100046828E-04
 3.32733158696484E-04
 2.02702871305976E-02
 2.94921959570891E-03
 1.49983876118864E-03
 4.07578211616034E-04

 0
 0
 1.59125227297935E-04
 0
 1.34027833952564E-04
 0
 0
 0
 0
 0
 0
 0
 0
 0
 0
 0
 0
 0
 0
 0

 0
 0
 1.59125227297935E-04
 0
 1.34027833952564E-04
 0
 0
 0
 0
 0
 0
 0
 0
 0
 0
 0
 0
 0
 0
 0

 0
 0
 1.59125227297935E-04
 0
 1.34027833952564E-04
 0
 0
 0
 0
 0
 0
 0
 0
 0
 0
 0
 0
 0
 0
 0

 0
 0
 1.59125227297935E-04
 0
 1.34027833952564E-04
 0
 0
 0
 0
 0
 0
 0
 0
 0
 0
 0
 0
 0
 0
 0

 0
 0
 1.59125227297935E-04
 0
 1.34027833952564E-04
 0
 0
 0
 0
 0
 0
 0
 0
 0
 0
 0
 0
 0
 0
 0

 0
 0
 0
 0
 0
 0
 0
 0
 0
 0
 0
 1.27494020803109E-03
 0
 3.34233301382711E-04
 0
 0
 0
 3.16112188284302E-04
 2.52900824306023E-04
 1.99495418303307E-04
 0
 0
 0
 0
 0
 0
 2.07257750118031E-04
 3.32733158696484E-04
 0
 0
 1.8747984514858E-04
 2.03789105808962E-04

 0
 0
 0
 0
 0
 0
 0
 0
 0
 0
 0
 1.27494020803109E-03
 0
 3.34233301382711E-04
 0
 0
 0
 3.16112188284302E-04
 2.52900824306023E-04
 1.99495418303307E-04
 0
 0
 0
 0
 0
 0
 2.07257750118031E-04
 3.32733158696484E-04
 0
 0
 1.8747984514858E-04
 2.03789105808962E-04

 0
 0
 0
 0
 0
 0
 0
 0
 0
 0
 0
 1.27494020803109E-03
 0
 3.34233301382711E-04
 0
 0
 0
 3.16112188284302E-04
 2.52900824306023E-04
 1.99495418303307E-04
 0
 0
 0
 0
 0
 0
 2.07257750118031E-04
 3.32733158696484E-04
 0
 0
 1.8747984514858E-04
 2.03789105808962E-04

 0
 0
 0
 0
 0
 0
 0
 0
 0
 0
 0
 1.27494020803109E-03
 0
 3.34233301382711E-04
 0
 0
 0
 3.16112188284302E-04
 2.52900824306023E-04
 1.99495418303307E-04
 0
 0
 0
 0
 0
 0
 2.07257750118031E-04
 3.32733158696484E-04
 0
 0
 1.8747984514858E-04
 2.03789105808962E-04

 0
 0
 0
 0
 0
 0
 0
 0
 0
 0
 0
 1.27494020803109E-03
 0
 3.34233301382711E-04
 0
 0
 0
 3.16112188284302E-04
 2.52900824306023E-04
 1.99495418303307E-04
 0
 0
 0
 0
 0
 0
 2.07257750118031E-04
 3.32733158696484E-04
 0
 0
 1.8747984514858E-04
 2.03789105808962E-04

 8.60928950841707E-03
 4.21957504582445E-03
 4.45550636433167E-03
 6.81093373685811E-03
 4.39611295363877E-02
 1.32696582328757E-02
 2.77859370198338E-03
 7.45442013647566E-02
 5.75942969905592E-02
 .136840027633511
 2.14321073432416E-02
 .169312059626447
 2.92171731217069E-02
 9.15799245788517E-02
 1.81506648353106E-02
 5.26260352936968E-02
 3.11770923141986E-02
 2.59211994391771E-02
 3.74293219972746E-02
 1.83535784839174E-02
 .029579042010119
 0
 0
 0
 4.01467976188201E-02
 1.34815762456496E-02
 4.84983135275078E-02
 .045251709582794
 0
 4.91536599285691E-02
 1.98728635857459E-02
 0

 8.60928950841707E-03
 4.21957504582445E-03
 4.45550636433167E-03
 6.81093373685811E-03
 4.39611295363877E-02
 1.32696582328757E-02
 2.77859370198338E-03
 7.45442013647566E-02
 5.75942969905592E-02
 .136840027633511
 2.14321073432416E-02
 .169312059626447
 2.92171731217069E-02
 9.15799245788517E-02
 1.81506648353106E-02
 5.26260352936968E-02
 3.11770923141986E-02
 2.59211994391771E-02
 3.74293219972746E-02
 1.83535784839174E-02
 .029579042010119
 0
 0
 0
 4.01467976188201E-02
 1.34815762456496E-02
 4.84983135275078E-02
 .045251709582794
 0
 4.91536599285691E-02
 1.98728635857459E-02
 0

 8.60928950841707E-03
 4.21957504582445E-03
 4.45550636433167E-03
 6.81093373685811E-03
 4.39611295363877E-02
 1.32696582328757E-02
 2.77859370198338E-03
 7.45442013647566E-02
 5.75942969905592E-02
 .136840027633511
 2.14321073432416E-02
 .169312059626447
 2.92171731217069E-02
 9.15799245788517E-02
 1.81506648353106E-02
 5.26260352936968E-02
 3.11770923141986E-02
 2.59211994391771E-02
 3.74293219972746E-02
 1.83535784839174E-02
 .029579042010119
 0
 0
 0
 4.01467976188201E-02
 1.34815762456496E-02
 4.84983135275078E-02
 .045251709582794
 0
 4.91536599285691E-02
 1.98728635857459E-02
 0

 8.60928950841707E-03
 4.21957504582445E-03
 4.45550636433167E-03
 6.81093373685811E-03
 4.39611295363877E-02
 1.32696582328757E-02
 2.77859370198338E-03
 7.45442013647566E-02
 5.75942969905592E-02
 .136840027633511
 2.14321073432416E-02
 .169312059626447
 2.92171731217069E-02
 9.15799245788517E-02
 1.81506648353106E-02
 5.26260352936968E-02
 3.11770923141986E-02
 2.59211994391771E-02
 3.74293219972746E-02
 1.83535784839174E-02
 .029579042010119
 0
 0
 0
 4.01467976188201E-02
 1.34815762456496E-02
 4.84983135275078E-02
 .045251709582794
 0
 4.91536599285691E-02
 1.98728635857459E-02
 0

 8.60928950841707E-03
 4.21957504582445E-03
 4.45550636433167E-03
 6.81093373685811E-03
 4.39611295363877E-02
 1.32696582328757E-02
 2.77859370198338E-03
 7.45442013647566E-02
 5.75942969905592E-02
 .136840027633511
 2.14321073432416E-02
 .169312059626447
 2.92171731217069E-02
 9.15799245788517E-02
 1.81506648353106E-02
 5.26260352936968E-02
 3.11770923141986E-02
 2.59211994391771E-02
 3.74293219972746E-02
 1.83535784839174E-02
 .029579042010119
 0
 0
 0
 4.01467976188201E-02
 1.34815762456496E-02
 4.84983135275078E-02
 .045251709582794
 0
 4.91536599285691E-02
 1.98728635857459E-02
 0

 .125204618498817
 .116187015888716
 .127390072578251
 .30336010732037
 .170875384205412
 .105357169278399
 .275915699324343
 .495774305486524
 .343003378395976
 .483625660972539
 .382161226675514
 .435624183612124
 .331072138097874
 .619644352567472
 .396375459703112
 .454211977695598
 .478769353094132
 .549001052336339
 .525088156280802
 .630823348209207
 .329992759292005
 .728860351871704
 .867358392988573
 .354871834932248
 .538311151045814
 .207852739675698
 .420906146969178
 .52898152396629
 .436683086795138
 .16113536027288
 .186525864336727
 .550844627964218

 0
 0
 0
 0
 0
 8.81704865970013E-05
 0
 1.57376920544947E-04
 4.07314688759458E-05
 0
 0
 7.28537261734455E-05
 3.4423768037363E-04
 3.81980915865955E-04
 2.82867503926494E-04
 2.66124072282003E-04
 1.099721069285E-04
 0
 2.16772135119209E-04
 6.2698560038088E-04
 1.17377150834132E-04
 1.85028188399539E-03
 1.39842174196109E-03
 0
 0
 6.62041690635769E-04
 5.32948500301312E-04
 1.90133233540848E-04
 0
 2.80878056734306E-04
 4.8209103038258E-04
 3.60997844574364E-03

 0
 0
 0
 0
 0
 8.81704865970013E-05
 0
 1.57376920544947E-04
 4.07314688759458E-05
 0
 0
 7.28537261734455E-05
 3.4423768037363E-04
 3.81980915865955E-04
 2.82867503926494E-04
 2.66124072282003E-04
 1.099721069285E-04
 0
 2.16772135119209E-04
 6.2698560038088E-04
 1.17377150834132E-04
 1.85028188399539E-03
 1.39842174196109E-03
 0
 0
 6.62041690635769E-04
 5.32948500301312E-04
 1.90133233540848E-04
 0
 2.80878056734306E-04
 4.8209103038258E-04
 3.60997844574364E-03

 0
 0
 0
 0
 0
 8.81704865970013E-05
 0
 1.57376920544947E-04
 4.07314688759458E-05
 0
 0
 7.28537261734455E-05
 3.4423768037363E-04
 3.81980915865955E-04
 2.82867503926494E-04
 2.66124072282003E-04
 1.099721069285E-04
 0
 2.16772135119209E-04
 6.2698560038088E-04
 1.17377150834132E-04
 1.85028188399539E-03
 1.39842174196109E-03
 0
 0
 6.62041690635769E-04
 5.32948500301312E-04
 1.90133233540848E-04
 0
 2.80878056734306E-04
 4.8209103038258E-04
 3.60997844574364E-03

 0
 0
 0
 0
 0
 8.81704865970013E-05
 0
 1.57376920544947E-04
 4.07314688759458E-05
 0
 0
 7.28537261734455E-05
 3.4423768037363E-04
 3.81980915865955E-04
 2.82867503926494E-04
 2.66124072282003E-04
 1.099721069285E-04
 0
 2.16772135119209E-04
 6.2698560038088E-04
 1.17377150834132E-04
 1.85028188399539E-03
 1.39842174196109E-03
 0
 0
 6.62041690635769E-04
 5.32948500301312E-04
 1.90133233540848E-04
 0
 2.80878056734306E-04
 4.8209103038258E-04
 3.60997844574364E-03

 6.02048217372588E-05
 2.38355094677648E-04
 5.78637190174308E-05
 2.2832352185504E-03
 1.59405462546598E-04
 5.51515057059997E-04
 4.97955860570498E-05
 3.77152204840469E-04
 5.53099902140574E-03
 6.04951492633188E-04
 1.35741707310868E-03
 8.75487306370548E-04
 8.61072840644135E-04
 2.97426742053343E-04
 6.71159177681895E-03
 8.46130485657119E-04
 4.9935980997408E-03
 1.06660026450904E-03
 1.33717677219084E-03
 3.6688812561452E-04
 2.62303332619887E-03
 1.04131239139538E-02
 1.2468025321496E-03
 0
 4.19506767176208E-04
 1.9854332833031E-03
 9.14316373506655E-02
 7.00613510527104E-04
 1.60684004206293E-04
 0
 2.58592889860277E-03
 1.22026209780618E-02

 6.02048217372588E-05
 9.62818608115261E-05
 5.78637190174308E-05
 2.2832352185504E-03
 1.59405462546598E-04
 5.51515057059997E-04
 4.97955860570498E-05
 3.77152204840469E-04
 5.48779897865852E-03
 6.04951492633188E-04
 1.35741707310868E-03
 8.75487306370548E-04
 8.61072840644135E-04
 2.97426742053343E-04
 6.71159177681895E-03
 8.46130485657119E-04
 4.9935980997408E-03
 1.06660026450904E-03
 1.33717677219084E-03
 3.6688812561452E-04
 2.62303332619887E-03
 1.04131239139538E-02
 1.11196847989387E-03
 0
 4.19506767176208E-04
 1.9854332833031E-03
 9.14316373506655E-02
 7.00613510527104E-04
 1.60684004206293E-04
 0
 2.58592889860277E-03
 1.22026209780618E-02

 0
 4.40916932688692E-05
 0
 0
 9.24329889327007E-05
 3.19237968713222E-04
 0
 3.37666955958181E-04
 4.75200470219033E-03
 6.04951492633188E-04
 1.35741707310868E-03
 8.20651168390535E-04
 7.96296933047053E-04
 1.5367048339435E-04
 6.67610660607548E-03
 6.95899154530348E-04
 4.91082339560106E-03
 7.2669468570984E-04
 1.33717677219084E-03
 3.6688812561452E-04
 9.44413857284431E-04
 1.02970668065347E-02
 9.20591115401856E-04
 0
 4.19506767176208E-04
 1.9854332833031E-03
 9.00499190165524E-02
 7.64903813095365E-05
 1.60684004206293E-04
 0
 2.58592889860277E-03
 .011712017575189

 0
 0
 0
 0
 0
 0
 0
 4.22083694948598E-05
 9.8317338665968E-05
 6.04951492633188E-04
 5.02747064114941E-05
 4.68943524794591E-04
 0
 7.68352416971749E-05
 3.79324238981889E-05
 5.35307041946557E-04
 4.4241652212688E-05
 6.54025217138736E-04
 0
 4.58610157019096E-05
 1.88882771456886E-04
 3.72183137584802E-04
 2.04575803422494E-04
 0
 0
 3.874016162543E-04
 4.76454597972486E-04
 0
 1.60684004206293E-04
 0
 2.5859288986011E-04
 4.59111088949273E-03

 0
 4.40916932688692E-05
 0
 0
 9.24329889327007E-05
 3.19237968713222E-04
 0
 2.95458586463322E-04
 4.65368736352436E-03
 0
 1.30714236669719E-03
 3.51707643595944E-04
 7.61675327262349E-04
 7.68352416971749E-05
 6.56230933438104E-03
 1.6059211258379E-04
 4.86658174338838E-03
 7.26694685711039E-05
 1.27903865166072E-03
 3.2102710991261E-04
 7.55531085827545E-04
 9.67676157722686E-03
 0
 0
 4.19506767176208E-04
 1.35590565689005E-03
 8.95734644185799E-02
 7.64903813095365E-05
 0
 0
 2.06874311888255E-03
 .006980362474794

 0
 0
 0
 0
 0
 0
 0
 0
 0
 0
 0
 0
 3.46216057847042E-05
 0
 7.58648477962525E-05
 0
 0
 0
 5.81381205301201E-05
 0
 0
 2.48122091723042E-04
 7.16015311979363E-04
 0
 0
 2.42126010158746E-04
 0
 0
 0
 0
 2.5859288986011E-04
 1.40544210902298E-04

 0
 5.21901675426569E-05
 0
 0
 1.82350794493284E-05
 0
 0
 0
 0
 0
 0
 0
 0
 0
 0
 0
 0
 0
 0
 0
 0
 0
 0
 0
 0
 0
 0
 1.2323450322092E-04
 0
 0
 0
 4.90603402872727E-04

 0
 5.21901675426569E-05
 0
 0
 1.82350794493284E-05
 0
 0
 0
 0
 0
 0
 0
 0
 0
 0
 0
 0
 0
 0
 0

 0
 0
 0
 0
 0
 0
 0
 0
 0
 0
 0
 0
 0
 0
 0
 0
 0
 0
 0
 0
 0
 0
 0
 0
 0
 0
 0
 1.2323450322092E-04
 0
 0
 0
 4.90603402872727E-04

 0
 0
 0
 2.2832352185504E-03
 0
 2.32277088346775E-04
 4.97955860570498E-05
 3.94852488822882E-05
 7.35794276468193E-04
 0
 0
 5.48361379800127E-05
 6.47759075970815E-05
 1.43756258658993E-04
 3.5485170743467E-05
 1.50231331126772E-04
 8.27747041397312E-05
 3.39905578799203E-04
 0
 0
 1.67861946891444E-03
 1.16057107419066E-04
 1.9137736449201E-04
 0
 0
 0
 1.38171833411318E-03
 5.00888625996647E-04
 0
 0
 0
 0

 0
 0
 0
 0
 0
 0
 0
 3.94852488822882E-05
 7.0513618161533E-04
 0
 0
 5.48361379800127E-05
 6.47759075970815E-05
 1.43756258658993E-04
 3.5485170743467E-05
 1.50231331126772E-04
 8.27747041397312E-05
 3.39905578799203E-04
 0
 0
 1.67861946891444E-03
 0
 0
 0
 0
 0
 1.38171833411318E-03
 5.00888625996647E-04
 0
 0
 0
 0

 0
 0
 0
 2.2832352185504E-03
 0
 2.32277088346775E-04
 4.97955860570498E-05
 0
 3.06580948528625E-05
 0
 0
 0
 0
 0
 0
 0
 0
 0
 0
 0

 0
 0
 0
 0
 0
 0
 0
 0
 0
 0
 0
 0
 0
 0
 0
 0
 0
 0
 0
 0
 0
 1.16057107419066E-04
 1.9137736449201E-04
 0
 0
 0
 0
 0
 0
 0
 0
 0

 6.02048217372588E-05
 0
 5.78637190174308E-05
 0
 4.87373941645686E-05
 0
 0
 0
 0
 0
 0
 0
 0
 0
 0
 0
 0
 0
 0
 0

 6.02048217372588E-05
 0
 5.78637190174308E-05
 0
 4.87373941645686E-05
 0
 0
 0
 0
 0
 0
 0
 0
 0
 0
 0
 0
 0
 0
 0

 0
 1.42073233866122E-04
 0
 0
 0
 0
 0
 0
 4.32000427472153E-05
 0
 0
 0
 0
 0
 0
 0
 0
 0
 0
 0
 0
 0
 1.34834052255734E-04
 0
 0
 0
 0
 0
 0
 0
 0
 0

 0
 0
 0
 0
 0
 0
 0
 0
 4.32000427472153E-05
 0
 0
 0
 0
 0
 0
 0
 0
 0
 0
 0
 0
 0
 1.34834052255734E-04
 0
 0
 0
 0
 0
 0
 0
 0
 0

 0
 0
 0
 0
 0
 0
 0
 0
 4.32000427472153E-05
 0
 0
 0
 0
 0
 0
 0
 0
 0
 0
 0
 0
 0
 1.34834052255734E-04
 0
 0
 0
 0
 0
 0
 0
 0
 0

 0
 1.42073233866122E-04
 0
 0
 0
 0
 0
 0
 0
 0
 0
 0
 0
 0
 0
 0
 0
 0
 0
 0

 0
 1.42073233866122E-04
 0
 0
 0
 0
 0
 0
 0
 0
 0
 0
 0
 0
 0
 0
 0
 0
 0
 0

 .124009122752892
 .11512666565524
 .125922813988882
 .299531534195137
 .16761419172854
 .100176703674999
 .274211978915678
 .49034797708086
 .332716980383313
 .471241439701916
 .369406436976806
 .405437680819735
 .304701894649393
 .578546589249529
 .370723531309243
 .409889106343686
 .450686899659927
 .483305141489146
 .490134623785624
 .599312156126162
 .324083165742453
 .653378981704025
 .815895536994955
 .34901782427178
 .534893669131778
 .203013669559057
 .319551744748015
 .444692055921928
 .432150432993082
 .159784109081025
 .181955649925408
 .48761683431218

 0
 0
 0
 0
 0
 0
 0
 0
 0
 0
 0
 0
 1.72118840186815E-04
 0
 2.35722919937967E-04
 2.66124072282003E-04
 4.94874481178159E-04
 9.03177680812291E-04
 2.89029513492597E-04
 3.41992145662812E-04
 0
 5.55084565198023E-03
 1.30943126747933E-02
 0
 0
 0
 5.92165000337233E-05
 3.80266467081695E-04
 0
 0
 0
 0

 0
 0
 0
 0
 0
 0
 0
 0
 0
 0
 0
 0
 1.72118840186815E-04
 0
 2.35722919937967E-04
 2.66124072282003E-04
 4.94874481178159E-04
 9.03177680812291E-04
 2.89029513492597E-04
 3.41992145662812E-04
 0
 5.55084565198023E-03
 1.30943126747933E-02
 0
 0
 0
 5.92165000337233E-05
 3.80266467081695E-04
 0
 0
 0
 0

 0
 0
 0
 0
 0
 0
 0
 0
 0
 0
 0
 0
 1.72118840186815E-04
 0
 2.35722919937967E-04
 2.66124072282003E-04
 4.94874481178159E-04
 9.03177680812291E-04
 2.89029513492597E-04
 3.41992145662812E-04
 0
 5.55084565198023E-03
 1.30943126747933E-02
 0
 0
 0
 5.92165000337233E-05
 3.80266467081695E-04
 0
 0
 0
 0

 .109750394795852
 .107256416509517
 .113607462037762
 .289001329340621
 .153503140555614
 9.16408884639165E-02
 .266870833978947
 .442062318413544
 .290471512275439
 .435445163558229
 .340862972411699
 .353157909722699
 .264648282565606
 .523913439464003
 .322311835022149
 .327752262961491
 .365745534508949
 .394731440654055
 .415088017171161
 .498615369633465
 .319517599323805
 .511199706244681
 .548864932670962
 .223504334659872
 .510912958073748
 .196807677261171
 .305007968144981
 .422794567127619
 .283827332746262
 .156733192257878
 .178160799266704
 .411827364695929

 8.38294986213612E-05
 0
 8.05697353405935E-05
 1.52142972528456E-04
 1.35724388812723E-04
 1.17188621426459E-04
 5.8620120295008E-05
 1.11558323424235E-03
 6.49641149159464E-04
 9.99318604917547E-04
 2.7682907960668E-04
 5.16431476672525E-04
 3.81275911806236E-05
 5.07696153999054E-04
 1.25321046043246E-04
 3.53709209995067E-04
 1.94887278101461E-04
 4.00142010485137E-04
 6.40255251407652E-05
 0
 2.08010140718343E-04
 0
 2.25292340477936E-04
 3.24193628348855E-04
 2.30994232559412E-04
 3.73303139713189E-04
 0
 8.42362427079705E-05
 0
 1.24439645388451E-04
 0
 5.15921786858131E-05

 8.38294986213612E-05
 0
 8.05697353405935E-05
 1.52142972528456E-04
 1.35724388812723E-04
 1.17188621426459E-04
 5.8620120295008E-05
 1.11558323424235E-03
 6.49641149159464E-04
 9.99318604917547E-04
 2.7682907960668E-04
 5.16431476672525E-04
 3.81275911806236E-05
 5.07696153999054E-04
 1.25321046043246E-04
 3.53709209995067E-04
 1.94887278101461E-04
 4.00142010485137E-04
 6.40255251407652E-05
 0
 2.08010140718343E-04
 0
 2.25292340477936E-04
 3.24193628348855E-04
 2.30994232559412E-04
 3.73303139713189E-04
 0
 8.42362427079705E-05
 0
 1.24439645388451E-04
 0
 5.15921786858131E-05

 2.64062920657793E-03
 1.50525692083481E-03
 2.57823153089527E-03
 5.73071863190396E-03
 5.90401091334111E-03
 1.2109490880728E-03
 5.80339190919902E-03
 3.34210143925051E-02
 1.89478668505042E-02
 5.99591162951262E-03
 4.09707037819057E-03
 2.38849557960936E-03
 2.85956933853733E-03
 9.30776282330202E-04
 4.46978397553976E-03
 2.29910986496112E-03
 5.99278380160465E-03
 1.36048283565264E-03
 6.59462908949987E-03
 8.33335291646307E-03
 1.24806084431006E-03
 1.50286608721145E-03
 2.36556957501343E-03
 2.91774265514012E-03
 2.77193079071222E-03
 2.13316079836229E-04
 4.35503626828298E-03
 5.98077323227276E-03
 2.30042542731114E-03
 0
 1.89853007745398E-04
 1.13502793108406E-03

 2.64062920657793E-03
 1.50525692083481E-03
 2.57823153089527E-03
 5.73071863190396E-03
 5.90401091334111E-03
 1.2109490880728E-03
 5.80339190919902E-03
 3.34210143925051E-02
 1.89478668505042E-02
 5.99591162951262E-03
 4.09707037819057E-03
 2.38849557960936E-03
 2.85956933853733E-03
 9.30776282330202E-04
 4.46978397553976E-03
 2.29910986496112E-03
 5.99278380160465E-03
 1.36048283565264E-03
 6.59462908949987E-03
 8.33335291646307E-03
 1.24806084431006E-03
 1.50286608721145E-03
 2.36556957501343E-03
 2.91774265514012E-03
 2.77193079071222E-03
 2.13316079836229E-04
 4.35503626828298E-03
 5.98077323227276E-03
 2.30042542731114E-03
 0
 1.89853007745398E-04
 1.13502793108406E-03

 4.41502026072867E-04
 5.1146364191846E-04
 4.24333939460809E-04
 1.3354772033046E-03
 2.05509345393783E-03
 1.02865567696332E-03
 1.08056421743543E-03
 8.56829900743228E-04
 1.04544103448041E-03
 1.46196610719204E-03
 2.91593297186185E-04
 1.69992027738039E-04
 6.0241594065319E-04
 6.68466602764686E-04
 5.50020146523012E-04
 1.55239042164502E-04
 0
 0
 3.3720109907414E-04
 2.65993891071076E-04
 1.64328011167785E-03
 3.59777032999104E-04
 0
 8.53709887985319E-04
 1.52071203101375E-03
 1.6851970307051E-03
 1.10537466729232E-03
 0
 9.31967224397595E-04
 6.55382132379175E-04
 1.24986563432387E-04
 5.57023555876065E-03

 1.10375506518308E-04
 2.55731820959019E-04
 1.0608348486529E-04
 6.67738601651199E-04
 1.07222267162051E-03
 5.14327838481659E-04
 7.71831583881724E-04
 2.44808543069782E-04
 1.90080188087433E-04
 0
 0
 0
 0
 0
 0
 0
 0
 0
 0
 0
 1.36940009306487E-03
 0
 0
 8.53709887985319E-04
 .001216569624811
 7.02165429460365E-04
 8.2903100046828E-04
 0
 0
 3.27691066189587E-04
 0
 0

 1.10375506518308E-04
 1.27865910479721E-04
 2.12166969730229E-04
 1.33547720330681E-04
 3.57407557206836E-04
 5.14327838481659E-04
 1.54366316776854E-04
 6.12021357673446E-04
 6.65280658305547E-04
 1.46196610719204E-03
 2.91593297186185E-04
 1.69992027738039E-04
 3.01207970326595E-04
 2.22822200921807E-04
 2.20008058609132E-04
 0
 0
 0
 3.3720109907414E-04
 1.32996945535538E-04
 2.73880018612974E-04
 3.59777032999104E-04
 0
 0
 3.04142406202751E-04
 0
 1.38171833412021E-04
 0
 9.31967224397595E-04
 3.27691066189587E-04
 0
 5.57023555876065E-03

 2.20751013036251E-04
 1.27865910479721E-04
 1.0608348486529E-04
 5.34190881322723E-04
 6.25463225110488E-04
 0
 1.54366316776854E-04
 0
 1.90080188087433E-04
 0
 0
 0
 3.01207970326595E-04
 4.45644401842879E-04
 3.3001208791388E-04
 1.55239042164502E-04
 0
 0
 0
 1.32996945535538E-04
 0
 0
 0
 0
 0
 9.83031601244732E-04
 1.38171833412021E-04
 0
 0
 0
 1.24986563432387E-04
 0

 1.86026134581418E-04
 8.62017374019165E-05
 3.57584780444797E-05
 1.35048256514023E-04
 9.03558431139883E-05
 1.04021360592025E-04
 1.56100769774175E-04
 1.2377960042861E-04
 1.60179933781598E-04
 9.85595128447329E-05
 9.82898754560173E-05
 0
 6.76871843431295E-05
 0
 3.70800098780048E-05
 0
 4.32474577809422E-05
 1.42072893610688E-04
 2.27326583645675E-04
 8.96608621586415E-05
 0
 7.21188243293686E-04
 1.79980734920765E-03
 0
 9.70667253838567E-05
 2.64432512796896E-03
 2.64584361851579E-04
 0
 0
 3.13746765500947E-04
 4.78671945060204E-04
 8.67187684293454E-05

 3.72052269162835E-05
 0
 0
 0
 0
 0
 0
 0
 0
 0
 0
 0
 0
 0
 0
 0
 0
 0
 0
 0
 0
 1.1482245734014E-04
 0
 0
 9.70667253838567E-05
 1.97203907848599E-03
 1.76389574567719E-04
 0
 0
 0
 1.99446643775085E-04
 4.33593842146727E-05

 1.48820907665134E-04
 8.62017374019165E-05
 3.57584780444797E-05
 1.35048256514023E-04
 3.01186143713626E-05
 1.04021360592025E-04
 5.20335899247824E-05
 8.25197336190278E-05
 6.4071973512618E-05
 0
 9.82898754560173E-05
 0
 0
 0
 0
 0
 0
 0
 0
 0
 0
 0
 0
 0
 0
 4.0337162968943E-04
 8.81947872838596E-05
 0
 0
 3.13746765500947E-04
 2.79225301285119E-04
 0

 0
 0
 0
 0
 0
 0
 0
 0
 3.20359867563619E-05
 0
 0
 0
 3.38435921715648E-05
 0
 0
 0
 0
 1.42072893610688E-04
 5.68316459114657E-05
 0
 0
 6.06365785953546E-04
 1.79980734920765E-03
 0
 0
 0
 0
 0
 0
 0
 0
 0

 0
 0

 0
 0
 0

 0
 0
 0
 0

 0
 0
 0
 0
 6.02372287426257E-05

 0
 0
 0
 0
 0
 0

 0
 0
 0
 0
 0
 0
 1.04067179849393E-04

 0
 0
 0
 0
 0
 0
 0
 4.1259866809582E-05

 0
 0
 0
 0
 0
 0
 0
 0
 6.4071973512618E-05

 0
 0
 0
 0
 0
 0
 0
 0
 0
 9.85595128447329E-05

 0
 0
 0
 0
 0
 0
 0
 0
 0
 0
 0

 0
 0
 0
 0
 0
 0
 0
 0
 0
 0
 0
 0

 0
 0
 0
 0
 0
 0
 0
 0
 0
 0
 0
 0
 3.38435921715648E-05

 0
 0
 0
 0
 0
 0
 0
 0
 0
 0
 0
 0
 0
 0

 0
 0
 0
 0
 0
 0
 0
 0
 0
 0
 0
 0
 0
 0
 3.70800098780048E-05

 0
 0
 0
 0
 0
 0
 0
 0
 0
 0
 0
 0
 0
 0
 0
 0

 0
 0
 0
 0
 0
 0
 0
 0
 0
 0
 0
 0
 0
 0
 0
 0
 4.32474577809422E-05

 0
 0
 0
 0
 0
 0
 0
 0
 0
 0
 0
 0
 0
 0
 0
 0
 0
 0

 0
 0
 0
 0
 0
 0
 0
 0
 0
 0
 0
 0
 0
 0
 0
 0
 0
 0
 1.7049493773421E-04

 0
 0
 0
 0
 0
 0
 0
 0
 0
 0
 0
 0
 0
 0
 0
 0
 0
 0
 0
 8.96608621586415E-05

 0
 0
 0
 0
 0
 0
 0
 0
 0
 0
 0
 0
 0
 0
 0
 0
 0
 0
 0
 0
 0
 0
 0
 0
 0
 0
 0
 0
 0
 0
 0
 4.33593842146727E-05

 0
 0
 0
 0
 0
 0
 0
 0
 0
 0
 0
 0
 0
 0
 0
 0
 0
 0
 0
 0
 0
 0
 0
 0
 0
 2.68914419793543E-04
 0
 0
 0
 0
 0
 0

 0
 0
 1.20094511168054E-04
 0
 0
 0
 0
 0
 5.37962796474756E-05
 0
 0
 0
 0
 0
 6.22664316819327E-05
 8.78711559421707E-05
 7.26230894812048E-05
 1.19287618220491E-04
 9.54342733230274E-05
 0
 1.74817033156905E-04
 2.87056143349906E-03
 .264699326244957
 0
 0
 0
 4.40973936421344E-05
 0
 0
 0
 7.97786575100341E-05
 1.0839846053628E-03

 0
 0
 1.20094511168054E-04
 0
 0
 0
 0
 0
 5.37962796474756E-05
 0
 0
 0
 0
 0
 6.22664316819327E-05
 8.78711559421707E-05
 7.26230894812048E-05
 1.19287618220491E-04
 9.54342733230274E-05
 0
 1.74817033156905E-04
 2.87056143349906E-03
 .264699326244957
 0
 0
 0
 4.40973936421344E-05
 0
 0
 0
 7.97786575100341E-05
 1.0839846053628E-03

 1.67658997242999E-04
 4.85566748657167E-05
 8.05697353405935E-05
 5.07143241762079E-05
 1.0179329160943E-04
 1.5625149523545E-04
 0
 9.76135329962635E-03
 4.5835792190714E-03
 7.77247803824352E-03
 2.15926682093284E-03
 3.42135853294589E-03
 1.2200829177787E-03
 2.11540064166133E-03
 8.77247322302995E-04
 2.47596446995963E-03
 1.65654186386081E-03
 6.40227216778333E-04
 2.88114863132915E-03
 1.81818609085891E-03
 3.120152110777E-04
 2.32261122568515E-03
 4.50584680955173E-04
 0
 0
 1.27989647901737E-03
 4.82726911665172E-03
 0
 0
 0
 8.06875282918398E-04
 3.04393854245054E-03

 1.67658997242999E-04
 4.85566748657167E-05
 8.05697353405935E-05
 5.07143241762079E-05
 1.0179329160943E-04
 1.5625149523545E-04
 0
 9.76135329962635E-03
 4.5835792190714E-03
 7.77247803824352E-03
 2.15926682093284E-03
 3.42135853294589E-03
 1.2200829177787E-03
 2.11540064166133E-03
 8.77247322302995E-04
 2.47596446995963E-03
 1.65654186386081E-03
 6.40227216778333E-04
 2.88114863132915E-03
 1.81818609085891E-03
 3.120152110777E-04
 2.32261122568515E-03
 4.50584680955173E-04
 0
 0
 1.27989647901737E-03
 4.82726911665172E-03
 0
 0
 0
 8.06875282918398E-04
 3.04393854245054E-03

 5.61231389076141E-05
 0
 0
 1.35811241014027E-04
 0
 0
 0
 0
 4.83254715477323E-05
 0
 0
 0
 0
 0
 0
 0
 0
 0
 0
 0
 0
 1.82937474406324E-04
 0
 0
 0
 0
 7.02568644467903E-05
 0
 2.36940819761822E-04
 0
 0
 2.07243158449151E-04

 5.61231389076141E-05
 0
 0
 1.35811241014027E-04
 0
 0
 0
 0
 4.83254715477323E-05
 0
 0
 0
 0
 0
 0
 0
 0
 0
 0
 0
 0
 1.82937474406324E-04
 0
 0
 0
 0
 7.02568644467903E-05
 0
 2.36940819761822E-04
 0
 0
 2.07243158449151E-04

 7.96380236902724E-04
 5.34123423522082E-04
 8.86267088746395E-04
 3.55000269233455E-04
 1.42510608253347E-03
 8.64661990525258E-04
 8.79301804424152E-04
 2.11495988157922E-02
 9.67731207329211E-03
 9.54904444699422E-03
 5.59194740806043E-03
 9.16665871091707E-03
 5.94790422416721E-03
 4.66531600972663E-03
 6.06847407641722E-03
 .011082888579845
 5.65173106494205E-03
 4.48159051744543E-03
 5.57022068724551E-03
 5.65657894934441E-03
 3.03020177965428E-03
 1.22176265602113E-02
 2.72843576694533E-03
 .019451617700906
 1.38596539535431E-02
 5.18273870285881E-03
 2.08663234910083E-02
 1.31441789772963E-02
 1.59539625788845E-02
 1.24439645388451E-04
 7.17378287412286E-03
 2.54196058767628E-03

 0
 0
 0
 0
 6.78621944062493E-05
 0
 0
 2.78895808560972E-04
 1.22709994841338E-03
 2.22070801092829E-04
 2.7682907960668E-04
 4.51877542087394E-04
 6.10041458888719E-04
 1.10000833366322E-03
 4.59510502158383E-04
 2.71177060996413E-03
 2.92330917152192E-04
 9.603408251675E-04
 2.56102100563061E-04
 5.05051691907106E-05
 3.120152110777E-04
 3.8254773128966E-03
 1.80233872382209E-03
 0
 0
 3.30639923745944E-03
 9.54959760030989E-03
 5.05417456247823E-04
 0
 0
 5.98036974399745E-03
 9.28659216343679E-04

 0
 4.85566748657167E-05
 8.05697353405935E-05
 5.07143241762079E-05
 0
 3.90628738088625E-05
 0
 1.85930539040648E-04
 1.19100877345941E-03
 4.44141602186391E-04
 1.66097447764374E-04
 3.87323607504394E-04
 3.81275911806236E-05
 8.4616025666509E-05
 2.08868410071616E-04
 2.35806139996711E-04
 2.92330917152192E-04
 4.00142010485137E-04
 2.56102100563061E-04
 1.51515507571965E-04
 0
 0
 1.17092729590506E-04
 0
 0
 1.66302338556315E-03
 4.03607197596349E-03
 8.75613575517062E-05
 7.35763598209492E-04
 0
 9.37399225741471E-04
 0

 0
 0
 0
 0
 0
 0
 0
 0
 0
 0
 0
 6.45539345840656E-05
 3.81275911806236E-05
 0
 0
 0
 0
 0
 0
 0

 6.70635988970613E-04
 4.37010073790649E-04
 7.25127618065208E-04
 3.04285945057247E-04
 1.35724388812723E-03
 7.42194602367743E-04
 8.79301804424152E-04
 2.05453245639103E-02
 6.49641149159464E-03
 8.7717966431684E-03
 4.65072853739662E-03
 8.13379575757309E-03
 4.61343853284727E-03
 2.9615608983348E-03
 4.7204260676279E-03
 7.89950568988788E-03
 4.87218195253653E-03
 2.96105087759847E-03
 4.92996543583786E-03
 5.35354793420031E-03
 2.49612168862012E-03
 0
 0
 .019451617700906
 1.38596539535431E-02
 2.13316079836229E-04
 6.19149734526603E-03
 1.24669639207888E-02
 .015218198980675
 1.24439645388451E-04
 4.74632519363494E-05
 8.77067037654996E-04

 0
 0
 8.05697353405935E-05
 0
 0
 0
 0
 9.29652695201706E-05
 2.52638224672992E-04
 1.11035400546598E-04
 2.21463263686075E-04
 6.45539345840656E-05
 2.28765547083742E-04
 3.38464102666036E-04
 2.50642092086767E-04
 1.17903069998161E-04
 9.74436390505697E-05
 1.60056804194319E-04
 6.40255251407652E-05
 5.05051691907106E-05

 0
 0
 0
 0
 0
 8.34045143486524E-05
 0
 0
 7.70595357112489E-05
 0
 0
 0
 0
 1.8066664939606E-04
 1.78384912385783E-04
 0
 0
 0
 0
 0
 2.22064879956466E-04
 7.00106658807943E-03
 0
 0
 0
 0
 0
 0
 0
 0
 0
 2.20312546819478E-04

 1.25744247932111E-04
 4.85566748657167E-05
 0
 0
 0
 0
 0
 4.6482634760162E-05
 4.33094099440437E-04
 0
 2.7682907960668E-04
 6.45539345840656E-05
 4.1940350298623E-04
 0
 2.50642092086767E-04
 1.17903069998161E-04
 9.74436390505697E-05
 0
 6.40255251407652E-05
 5.05051691907106E-05
 0
 4.09872569238959E-04
 0
 0
 0
 0
 2.09881265941337E-04
 8.42362427079705E-05
 0
 0
 9.49265038726988E-05
 5.15921786858131E-04

 0
 0
 0
 0
 0
 0
 0
 0
 0
 0
 0
 0
 0
 0
 0
 0
 0
 0
 0
 0
 0

 0
 0
 0
 0
 0
 0
 0
 0
 0
 0
 0
 0
 0
 0
 0
 0
 0
 0
 0
 0
 0
 9.81210089996296E-04

 0
 0
 0
 0
 0
 0
 0
 0
 0
 0
 0
 0
 0
 0
 0
 0
 0
 0
 0
 0
 0
 0
 8.09004313532733E-04

 0
 0
 0
 0
 0
 0
 0
 0
 0
 0
 0
 0
 0
 0
 0
 0
 0
 0
 0
 0
 0
 0
 0
 0

 0
 0
 0
 0
 0
 0
 0
 0
 0
 0
 0
 0
 0
 0
 0
 0
 0
 0
 0
 0
 0
 0
 0
 0
 0

 0
 0
 0
 0
 0
 0
 0
 0
 0
 0
 0
 0
 0
 0
 0
 0
 0
 0
 0
 0
 0
 0
 0
 0
 0
 0

 0
 0
 0
 0
 0
 0
 0
 0
 0
 0
 0
 0
 0
 0
 0
 0
 0
 0
 0
 0
 0
 0
 0
 0
 0
 0
 8.79275303527547E-04

 0
 0
 0
 0
 0
 0
 0
 0
 0
 0
 0
 0
 0
 0
 0
 0
 0
 0
 0
 0
 0
 0
 0
 0
 0
 0
 0
 0

 0
 0
 0
 0
 0
 0
 0
 0
 0
 0
 0
 0
 0
 0
 0
 0
 0
 0
 0
 0
 0
 0
 0
 0
 0
 0
 0
 0
 0

 0
 0
 0
 0
 0
 0
 0
 0
 0
 0
 0
 0
 0
 0
 0
 0
 0
 0
 0
 0
 0
 0
 0
 0
 0
 0
 0
 0
 0
 0

 0
 0
 0
 0
 0
 0
 0
 0
 0
 0
 0
 0
 0
 0
 0
 0
 0
 0
 0
 0
 0
 0
 0
 0
 0
 0
 0
 0
 0
 0
 1.13624148574897E-04

 0
 0
 0
 0
 0
 0
 0
 0
 0
 0
 0
 0
 0
 0
 0
 0
 0
 0
 0
 0
 0
 0
 0
 0
 0
 0
 0
 0
 0
 0
 0
 0

 0
 0
 0
 0
 0
 0
 0
 0
 2.52638224672992E-04
 0
 5.53658159215189E-05
 6.45539345840656E-05
 7.62551823611213E-05
 0
 0
 0
 4.87218195253653E-05
 8.00284020972916E-05
 6.40255251407652E-05
 5.05051691907106E-05
 0
 8.41317378964733E-04
 3.37938510716205E-04
 0
 1.15497116279526E-04
 1.59987059876961E-03
 0
 1.75122715103412E-04
 0
 0
 2.13584633713801E-03
 1.96864892352896E-03

 0
 0
 0
 0
 0
 0
 0
 0
 2.52638224672992E-04
 0
 5.53658159215189E-05
 6.45539345840656E-05
 7.62551823611213E-05
 0
 0
 0
 4.87218195253653E-05
 8.00284020972916E-05
 6.40255251407652E-05
 5.05051691907106E-05
 0
 2.73248379492464E-04
 3.37938510716205E-04
 0
 1.15497116279526E-04
 1.59987059876961E-03
 0
 0
 0
 0
 2.13584633713801E-03
 1.75413407530999E-03

 0
 0
 0
 0
 0
 0
 0
 0
 0
 0
 0
 0
 0
 0
 0
 0
 0
 0
 0
 0
 0
 5.6806899947227E-04
 0
 0
 0
 0
 0
 1.75122715103412E-04
 0
 0
 0
 2.14514848218965E-04

 5.44891741039609E-04
 4.85566748657167E-04
 5.23703279714589E-04
 1.11571513187574E-03
 7.46484138469414E-04
 7.03131728558752E-04
 4.68960962360064E-04
 5.11308982361629E-04
 5.41367624300546E-04
 4.44141602186391E-04
 2.21463263685893E-04
 6.45539345840656E-04
 3.43148320624731E-04
 4.23080128332545E-04
 4.17736820143922E-04
 4.71612279993422E-04
 8.28270931930405E-04
 4.8017041258375E-04
 5.1220420112591E-04
 2.02020676762675E-04
 1.04005070359357E-03
 4.09872569238959E-04
 0
 1.62096814174259E-03
 6.92982697677154E-04
 1.97317373848617E-03
 3.67292215397583E-04
 5.05417456247823E-04
 7.0782320840282E-04
 7.46637872331369E-04
 5.69559023237568E-04
 5.15921786858131E-05

 3.35317994485998E-04
 1.94226699462867E-04
 4.02848676703632E-04
 6.08571890114495E-04
 3.73242069234427E-04
 1.17188621426459E-04
 2.34480481180032E-04
 4.6482634760162E-05
 3.60911749533697E-05
 0
 0
 0
 7.62551823611213E-05
 0
 1.67094728057845E-04
 0
 3.89774556202922E-04
 0
 3.84153150844591E-04
 1.01010338381254E-04
 0
 0
 0
 0
 0
 0
 0
 0
 3.53911604201618E-04
 0
 0
 0

 4.19147493107498E-05
 0
 0
 5.07143241762079E-05
 3.39310972031806E-05
 0
 0
 9.29652695201706E-05
 3.60911749533697E-05
 0
 5.53658159215189E-05
 0
 7.62551823611213E-05
 8.4616025666509E-05
 4.17736820144612E-05
 0
 0
 0
 0
 5.05051691907106E-05
 0
 1.36624189746495E-04
 0
 3.24193628348855E-04
 0
 1.06658039918114E-04
 1.04940632970668E-04
 0
 1.76955802100601E-04
 0
 4.74632519363494E-05
 0

 4.19147493107498E-05
 0
 0
 0
 0
 0
 0
 0
 3.60911749533697E-05
 0
 0
 0
 0
 0
 0
 0
 0
 0
 0
 0

 1.25744247932111E-04
 0
 0
 0
 0
 0
 0
 0
 0
 0
 0
 0
 0
 0
 0
 0
 0
 0
 0
 0
 1.04005070359357E-03

 0
 2.913400491943E-04
 0
 0
 0
 0
 0
 0
 0
 0
 0
 0
 0
 0
 0
 0
 0
 0
 0
 0
 0
 2.73248379492464E-04

 0
 0
 1.20854603010957E-04
 0
 0
 0
 0
 0
 0
 0
 0
 0
 0
 0
 0
 0
 0
 0
 0
 0
 0
 0
 0

 0
 0
 0
 4.56428917585034E-04
 0
 0
 0
 0
 0
 0
 0
 0
 0
 0
 0
 0
 0
 0
 0
 0
 0
 0
 0
 1.29677451339374E-03

 0
 0
 0
 0
 3.39310972031806E-04
 0
 0
 0
 0
 0
 0
 0
 0
 0
 0
 0
 0
 0
 0
 0
 0
 0
 0
 0
 6.92982697677154E-04

 0
 0
 0
 0
 0
 5.85943107132293E-04
 0
 0
 0
 0
 0
 0
 0
 0
 0
 0
 0
 0
 0
 0
 0
 0
 0
 0
 0
 1.86651569856805E-03

 0
 0
 0
 0
 0
 0
 2.34480481180032E-04
 0
 0
 0
 0
 0
 0
 0
 0
 0
 0
 0
 0
 0
 0
 0
 0
 0
 0
 0
 2.62351582426914E-04

 0
 0
 0
 0
 0
 0
 0
 3.71861078081296E-04
 0
 0
 0
 0
 0
 0
 0
 0
 0
 0
 0
 0
 0
 0
 0
 0
 0
 0
 0
 5.05417456247823E-04

 0
 0
 0
 0
 0
 0
 0
 0
 4.33094099440437E-04
 0
 0
 0
 0
 0
 0
 0
 0
 0
 0
 0
 0
 0
 0
 0
 0
 0
 0
 0
 1.76955802100601E-04

 0
 0
 0
 0
 0
 0
 0
 0
 0
 4.44141602186391E-04
 0
 0
 0
 0
 0
 0
 0
 0
 0
 0
 0
 0
 0
 0
 0
 0
 0
 0
 0
 7.46637872331369E-04

 0
 0
 0
 0
 0
 0
 0
 0
 0
 0
 1.66097447764374E-04
 0
 0
 0
 0
 0
 0
 0
 0
 0
 0
 0
 0
 0
 0
 0
 0
 0
 0
 0
 5.22095771301219E-04

 0
 0
 0
 0
 0
 0
 0
 0
 0
 0
 0
 6.45539345840656E-04
 0
 0
 0
 0
 0
 0
 0
 0
 0
 0
 0
 0
 0
 0
 0
 0
 0
 0
 0
 5.15921786858131E-05

 0
 0
 0
 0
 0
 0
 0
 0
 0
 0
 0
 0
 1.90637955902489E-04

 0
 0
 0
 0
 0
 0
 0
 0
 0
 0
 0
 0
 0
 3.38464102666036E-04

 0
 0
 0
 0
 0
 0
 0
 0
 0
 0
 0
 0
 0
 0
 2.08868410071616E-04

 0
 0
 0
 0
 0
 0
 0
 0
 0
 0
 0
 0
 0
 0
 0
 4.71612279993422E-04

 0
 0
 0
 0
 0
 0
 0
 0
 0
 0
 0
 0
 0
 0
 0
 0
 4.38496375727483E-04

 0
 0
 0
 0
 0
 0
 0
 0
 0
 0
 0
 0
 0
 0
 0
 0
 0
 4.8017041258375E-04

 0
 0
 0
 0
 0
 0
 0
 0
 0
 0
 0
 0
 0
 0
 0
 0
 0
 0
 1.28051050281319E-04

 0
 0
 0
 0
 0
 0
 0
 0
 0
 0
 0
 0
 0
 0
 0
 0
 0
 0
 0
 5.05051691907106E-05

 0
 0
 0
 0
 0
 0
 0
 0
 0
 0
 0
 0
 0
 0
 8.35473640287844E-05
 0
 2.92330917152192E-04
 0
 6.40255251407652E-05
 1.51515507571965E-04

 0
 0
 0
 0
 0
 0
 0
 0
 0
 0
 0
 0
 0
 0
 8.35473640287844E-05
 0
 2.92330917152192E-04
 0
 6.40255251407652E-05
 1.51515507571965E-04

 5.02976991728997E-04
 4.3701007379145E-04
 2.41709206022179E-04
 4.05714593408826E-04
 6.78621944062493E-04
 5.07817359513923E-04
 1.7586036088483E-04
 5.06660718884615E-02
 2.75375664894312E-02
 .129245206236069
 3.94758267519239E-02
 9.38614208852058E-02
 4.35417091282388E-02
 .148924205172729
 3.48810244820378E-02
 8.05867483437272E-02
 .053301670560682
 6.73038861636542E-02
 6.09522999340106E-02
 5.32324483268655E-02
 7.28035492516245E-04
 .122005401443346
 .104535645981332
 6.48387256697711E-04
 2.30994232559412E-04
 1.01325137922314E-03
 8.91995380250924E-03
 1.28039088916024E-02
 1.76955802100601E-04
 0
 6.17022275172542E-04
 4.01903071961078E-02

 5.02976991728997E-04
 3.88453398925733E-04
 2.41709206022179E-04
 3.55000269232618E-04
 3.05379874828066E-04
 2.73440116661393E-04
 1.7586036088483E-04
 2.74247545084419E-03
 4.33094099440437E-04
 3.33106201639427E-04
 1.66097447764374E-04
 3.87323607504394E-04
 3.81275911806236E-05
 1.69232051332739E-04
 4.17736820144612E-05
 3.53709209995067E-04
 2.43609097626022E-04
 1.60056804194319E-04
 2.56102100563061E-04
 0
 1.04005070359357E-04
 0
 4.50584680955173E-04
 3.24193628348855E-04
 2.30994232559412E-04
 4.26632159672457E-04
 1.73152044401871E-03
 0
 0
 0
 1.42389755809048E-04
 2.57960893428109E-04

 0
 0
 0
 0
 2.03586583219084E-04
 1.95314369043668E-04
 0
 1.15741760552673E-02
 8.87842903851823E-03
 1.27690710628313E-02
 5.42584996029605E-03
 9.61853625300766E-03
 5.1090972182023E-03
 1.00693070543048E-02
 1.12788941438838E-03
 7.25103880488038E-03
 7.30827292879675E-04
 6.80241417826318E-03
 1.21648497767348E-03
 3.5353618433414E-04
 0
 1.36624189746495E-04
 5.63230851194141E-04
 0
 0
 0
 0
 0
 0
 0
 0
 5.15921786858131E-05

 0
 4.85566748657167E-05
 0
 5.07143241762079E-05
 1.69655486015343E-04
 3.90628738088625E-05
 0
 .03634942038235
 1.82260433514726E-02
 .116143028971598
 3.38838793438635E-02
 8.38555610246938E-02
 3.83944843188559E-02
 .138685666067091
 .033711361385635
 7.29820003288517E-02
 5.23272341701763E-02
 6.03414151811967E-02
 .059479712855774
 5.28789121425314E-02
 6.24030422156887E-04
 .1218687772536
 .103521830449183
 3.24193628348855E-04
 0
 5.86619219550682E-04
 7.18843335849054E-03
 1.28039088916024E-02
 1.76955802100601E-04
 0
 4.74632519363494E-04
 3.98807541239939E-02

 0
 0
 0
 0
 0
 5.230452594746E-05
 0
 0
 0
 0
 0
 0
 5.1052198360496E-05
 0
 0
 0
 6.52376905509128E-05
 0
 0
 0
 0
 0
 0
 0
 0
 1.42813307686403E-04
 0
 0
 0
 0
 0
 2.07243158449151E-04

 0
 0
 0
 0
 0
 5.230452594746E-05
 0
 0
 0
 0
 0
 0
 5.1052198360496E-05
 0
 0
 0
 6.52376905509128E-05
 0
 0
 0

 0
 0
 0
 0
 0
 0
 0
 0
 0
 0
 0
 0
 0
 0
 0
 0
 0
 0
 0
 0
 0
 0
 0
 0
 0
 7.14066538432013E-05
 0
 0
 0
 0
 0
 0

 0
 0
 0
 0
 0
 0
 0
 0
 0
 0
 0
 0
 0
 0
 0
 0
 0
 0
 0
 0
 0
 0
 0
 0
 0
 7.14066538432013E-05
 0
 0
 0
 0
 0
 2.07243158449151E-04

 5.21898120820267E-03
 3.65036550885225E-03
 4.78607619137062E-03
 2.97251377509884E-03
 1.48831424386379E-02
 2.38913576585554E-03
 2.76365502616232E-03
 9.18032036512124E-03
 3.35115566916319E-03
 1.54213844210575E-02
 2.15873102271864E-02
 3.28194285809344E-02
 3.28720684763729E-02
 1.25067945033331E-02
 3.89627174763364E-02
 2.32107406591016E-02
 .029612650405975
 3.12033321338361E-02
 2.55348251637677E-02
 2.74145316765538E-02
 7.95135537908162E-04
 8.87836871755854E-03
 5.59778791137971E-03
 4.58524826933562E-02
 7.06395266020487E-03
 7.82348191405033E-03
 7.8267657893694E-03
 3.10193170526722E-02
 .060653028232951
 2.37840289976229E-03
 5.96306862311645E-03
 1.01237168691786E-02

 0
 6.18706018450261E-05
 5.13307184832048E-05
 1.29239729352058E-04
 0
 0
 0
 0
 0
 1.13184472815242E-03
 4.23280592690322E-04
 5.75779448788776E-04
 4.85819306978914E-05
 1.72507510390721E-03
 2.66138780575124E-04
 3.68066761261368E-03
 1.86243084314498E-04
 2.65126351463917E-03
 3.26323644265835E-04
 2.57413442972009E-04
 1.32522589651439E-04
 1.04451396677159E-03
 1.14826418694671E-03
 0
 0
 2.03854479520644E-04
 6.01716048727288E-04
 2.14666553997731E-04
 0
 0
 5.44296324625494E-04
 2.23510632176596E-03

 0
 0
 5.13307184832048E-05
 0
 0
 0
 0
 0
 0
 0
 0
 8.22542069700191E-05
 0
 0
 0
 2.25346996690158E-04
 0
 0
 0
 0

 1.44200258515589E-03
 9.89929629518375E-04
 8.21291495729581E-04
 3.87719188056815E-04
 1.27110268329048E-02
 1.29411520650487E-03
 4.48160274513448E-04
 7.69962353203641E-03
 2.71324139447301E-03
 1.35821367378103E-02
 .020388015214564
 3.08453276136553E-02
 3.14810910921823E-02
 1.00269990414653E-02
 3.81110733784941E-02
 1.85535693941631E-02
 2.87435160125086E-02
 2.74303802091576E-02
 2.48821778752363E-02
 .026964058151353
 0
 6.96342644514395E-03
 3.1577265141146E-03
 2.85028946472308E-02
 2.94331360841831E-03
 5.77587691975247E-03
 4.88058572856667E-03
 2.82286518506988E-02
 4.19385250978721E-02
 6.34240773269323E-04
 3.68911953357059E-03
 7.36270317758485E-03

 0
 0
 0
 0
 0
 0
 0
 0
 7.50316531925318E-05
 0
 0
 0
 7.92652553491912E-05
 0
 0
 0
 0
 0
 0
 0

 1.98675911732735E-04
 0
 6.36500909191739E-05
 0
 5.36111335810254E-05
 0
 0
 0
 5.70240564263242E-05
 0
 0
 0
 0
 0
 0
 0
 0
 0
 0
 0

 5.34075031540199E-05
 1.23741203689848E-04
 2.05322873932819E-04
 6.46198646761359E-05
 4.32347851459882E-05
 0
 0
 5.33050859909912E-04
 1.37961426837729E-04
 1.41480591019052E-04
 2.11640296344928E-04
 6.58033655760152E-04
 3.88655445583131E-04
 4.31268775977691E-04
 5.32277561152005E-05
 4.50693993380811E-04
 3.1040514052348E-04
 5.09858368198804E-04
 8.15809110664589E-05
 1.28706721485792E-04
 1.32522589651439E-04
 8.70428305642994E-04
 1.2917972103184E-03
 0
 2.94331360841831E-04
 3.39757465867918E-04
 5.34858709979535E-04
 2.03933226298136E-03
 0
 0
 1.81432108208303E-04
 3.28692106142268E-04

 0
 0
 0
 0
 0
 0
 0
 0
 0
 1.41480591019052E-04
 0
 0
 0
 0
 0
 0
 0
 0
 0
 0
 0
 0
 0
 0
 1.47165680420686E-04
 2.03854479520644E-04
 4.68001371232404E-04
 0
 0
 0
 0
 0

 1.12155756623354E-03
 9.2805902767437E-04
 1.38592939904399E-03
 5.16958917409087E-04
 4.75582636605157E-04
 1.49320985365971E-04
 1.49386758170903E-04
 5.92278733234322E-04
 3.21909995954297E-04
 4.24441773056689E-04
 5.64374123587095E-04
 6.58033655760152E-04
 8.74474752560441E-04
 3.23451581982912E-04
 5.32277561152005E-04
 3.00462662253874E-04
 3.1040514052348E-04
 6.11830041840584E-04
 2.44742733199107E-04
 6.43533607430022E-05
 1.32522589651439E-04
 0
 0
 1.40449046087617E-02
 1.0301597629471E-03
 8.15417918083648E-04
 1.06971741995659E-03
 4.29333107995463E-04
 1.51068880728957E-02
 1.58560193317542E-03
 2.41909477611071E-04
 1.97215263685483E-04

 2.40333764192649E-03
 1.54676504612463E-03
 2.20722089477865E-03
 1.87397607560474E-03
 1.59968705040085E-03
 9.45699573984705E-04
 2.16610799347797E-03
 3.55367239940593E-04
 4.59871422792937E-05
 0
 0
 0
 0
 0
 0
 0
 6.20810281049009E-05
 0
 0
 0
 3.97567768953844E-04
 0
 0
 3.3046834373636E-03
 2.64898224757694E-03
 2.03854479520644E-04
 1.33714677494884E-04
 1.07333276998866E-04
 3.60761506218318E-03
 1.58560193317542E-04
 1.81432108208303E-04
 0

 0
 0
 0
 0
 0
 0
 0
 0
 0
 0
 0
 0
 0
 0
 0
 0
 0
 0
 0
 0
 0
 0
 0
 0
 0
 2.80866171784368E-04
 1.38171833412021E-04
 0
 0
 0
 1.12487907089269E-03
 0

 3.22412663776731E-03
 3.12934991437011E-03
 3.5175050244779E-03
 5.79878259329543E-03
 1.07186996679208E-02
 7.22765962498258E-03
 4.75285764812142E-03
 .018935618689943
 2.37100024088066E-02
 1.81553106838463E-02
 3.34833513755766E-02
 2.75923901864995E-02
 4.91404950535787E-02
 1.59024686552189E-02
 .042523794433416
 2.69135476257452E-02
 4.92573748941253E-02
 3.30753173846614E-02
 3.85340992823572E-02
 2.58976552233637E-02
 1.03353670181736E-02
 6.78842454369258E-03
 4.19191971933722E-03
 2.08934262059731E-02
 1.24858461494001E-02
 3.43691499683284E-03
 1.09028484864277E-02
 5.16699570913362E-02
 1.98656171516113E-02
 8.79591809249252E-03
 1.97347205419653E-03
 3.53520469865491E-02

 2.70129529110164E-03
 2.32177574291958E-03
 2.34500334965101E-03
 4.4281612530595E-03
 6.91301459332645E-03
 4.95378918116438E-03
 3.77791248953277E-03
 1.27558135599474E-02
 7.12800705327194E-03
 4.84757182912266E-03
 1.61143664234356E-03
 .00322090157819
 4.43885429954424E-03
 1.23138584719656E-03
 1.82375101215623E-03
 3.18648560231993E-03
 1.41806137881387E-03
 1.33099868751285E-03
 9.31739879019991E-04
 5.24987942901705E-04
 5.62174775047066E-03
 2.84034499736135E-04
 0
 .01684953726289
 1.10451715937013E-02
 1.77389161126969E-03
 2.83615868581355E-03
 1.22585900572864E-03
 1.80262081560936E-02
 7.50240072595605E-03
 8.88062424388964E-04
 1.39434651342775E-03

 0
 0
 0
 0
 0
 0
 0
 0
 0
 0
 0
 0
 0
 0
 0
 0
 3.03870295460496E-04
 0
 1.3310569700317E-04
 3.14992765741717E-04

 8.71385577776114E-05
 3.02840314293742E-04
 3.35000478521968E-04
 4.21729643149518E-04
 4.23245791429148E-04
 3.24838634831594E-04
 0
 2.70577863393045E-03
 6.90291209369311E-03
 2.53920429144339E-03
 9.09310676751826E-03
 4.83135236727392E-03
 1.63286426019098E-02
 5.10145565267477E-03
 1.69348308271568E-02
 1.28684995478367E-02
 2.60315553111094E-02
 .013975486218852
 1.75699520043833E-02
 1.11297443895217E-02
 1.94598960593275E-03
 3.69244849656975E-03
 3.04441096935171E-03
 1.3479629810277E-03
 2.4011242594954E-04
 2.21736451408711E-04
 9.81747237397154E-04
 2.38166892541021E-02
 3.67881799103881E-04
 2.58703473307569E-04
 5.92041616258674E-04
 3.18554549605765E-02

 0
 0
 0
 0
 0
 0
 0
 0
 0
 0
 0
 0
 0
 0
 0
 0
 0
 0
 0
 0
 8.21640055837455E-04

 0
 0
 0
 0
 0
 0
 0
 0
 0
 0
 0
 0
 0
 0
 0
 0
 0
 0
 0
 0
 0

 8.71385577776114E-05

 3.48554231110446E-04
 4.03787085725433E-04
 5.02500717782952E-04
 8.43459286299037E-04
 3.17434343571279E-03
 1.78661249157108E-03
 8.53077013764815E-04
 3.28558834120195E-03
 3.90164596601661E-03
 4.61673507535092E-04
 5.7551308655073E-04
 6.71021162121624E-04
 1.18897883023656E-03
 8.79561319425282E-04
 1.73690572586157E-03
 6.12785692752588E-04
 9.1161088638082E-04
 1.33099868751285E-03
 9.31739879019991E-04
 1.25997106296825E-03
 1.94598960593275E-03
 0
 0
 0
 4.8022485189983E-04
 9.97814031337012E-04
 6.98131368815799E-03
 1.75122715103412E-04
 3.67881799103881E-04
 1.0348138932289E-03
 4.93368013548895E-04
 1.2870890893118E-03

 0
 0
 0
 0
 0
 0
 0
 0
 0
 0
 0
 0
 0
 0
 0
 0
 0
 0
 0
 0
 0
 5.39665549497616E-04

 0
 0
 0
 0
 0
 0
 0
 0
 0
 0
 0
 0
 0
 0
 0
 0
 0
 0
 0
 0
 0
 0

 0
 1.00946771431358E-04
 0
 0
 0
 0
 0
 0
 0
 0
 0
 0
 0
 0
 0
 0
 0
 0
 0
 0
 0
 0

 0
 0
 0
 0
 0
 0
 0
 0
 0
 0
 0
 0
 0
 0
 0
 0
 0
 0
 0
 0
 0
 0
 4.44952372443924E-04

 0
 0
 0
 0
 0
 0
 0
 0
 0
 0
 0
 0
 0
 0
 0
 0
 0
 0
 0
 0
 0
 0
 0

 0
 0
 3.35000478521968E-04

 0
 0
 0
 0
 0
 0
 0
 0
 0
 0
 0
 0
 0
 0
 0
 0
 0
 0
 0
 0
 0
 0
 0
 0

 0
 0
 0
 0
 0
 0
 0
 0
 0
 0
 0
 0
 0
 0
 0
 0
 0
 0
 0
 0
 0
 0
 0
 0

 0
 0
 0
 1.0543241078738E-04
 0
 0
 0
 0
 0
 0
 0
 0
 0
 0
 0
 0
 0
 0
 0
 0
 0
 2.27227599788908E-03

 0
 0
 0
 0
 6.70139169762818E-05
 0
 0
 0
 0
 0
 0
 0
 0
 0
 0
 0
 0
 0
 0
 0
 0
 0
 0
 0
 0

 0
 0
 0
 0
 0
 0
 0
 0
 0
 0
 0
 0
 0
 0
 0
 0
 0
 0
 0
 0
 0
 0
 0
 0
 0

 0
 0
 0
 0
 1.4108193047615E-04

 0
 0
 0
 0
 0
 0
 0
 0
 0
 0
 0
 0
 0
 0
 0
 0
 0
 0
 0
 0
 0
 0
 0
 0
 0
 0

 0
 0
 0
 0
 0
 0
 0
 0
 0
 0
 0
 0
 0
 0
 0
 0
 0
 0
 0
 0
 0
 0
 0
 0
 0
 0

 0
 0
 0
 0
 0
 1.62419317415529E-04
 0
 0
 0
 0
 0
 0
 0
 0
 0
 0
 0
 0
 0
 0
 0
 0
 0

 0
 0
 0
 0
 0
 0
 0
 0
 0
 0
 0
 0
 0
 0
 0
 0
 0
 0
 0
 0
 0
 0
 0
 0
 0
 0
 1.03628875059016E-04

 0
 0
 0
 0
 0
 0
 0
 0
 0
 0
 0
 0
 0
 0
 0
 0
 0
 0
 0
 0
 0
 0
 0
 0
 0
 0
 0

 0
 0
 0
 0
 0
 0
 1.21868144823832E-04

 0
 0
 0
 0
 0
 0
 0
 0
 0
 0
 0
 0
 0
 0
 0
 0
 0
 0
 0
 0
 0
 0
 0
 0
 0
 0
 0
 0

 0
 0
 0
 0
 0
 0
 0
 9.180320365132E-05
 0
 0
 0
 0
 0
 0
 0
 0
 0
 0
 0
 0
 0
 0
 0
 0
 0
 0
 0
 .026452286116402

 0
 0
 0
 0
 0
 0
 0
 9.66349512119157E-05
 0
 0
 0
 0
 0
 0
 0
 0
 0
 0
 0
 0
 0
 0
 7.02556377541584E-04

 0
 0
 0
 0
 0
 0
 0
 0
 0
 0
 0
 0
 0
 0
 0
 0
 0
 0
 0
 0
 0
 0
 0
 0
 0
 0
 0
 0
 0

 0
 0
 0
 0
 0
 0
 0
 0
 5.70240564263242E-03
 0
 0
 0
 0
 0
 0
 0
 0
 0
 0
 0
 0
 0
 0
 0
 0
 0
 0
 0
 0

 0
 0
 0
 0
 0
 0
 0
 0
 7.50316531925318E-05

 0
 0
 0
 0
 0
 0
 0
 0
 0
 2.19294916079531E-04
 0
 0
 0
 0
 0
 0
 0
 0
 0
 0
 0
 0
 0
 0
 0
 0
 0
 0
 0
 0

 0
 0
 0
 0
 0
 0
 0
 0
 0
 1.00875661396656E-02
 0
 0
 0
 0
 0
 0
 0
 0
 0
 0
 0
 0
 0
 0
 0
 0
 0
 0
 0
 0

 0
 0
 0
 0
 0
 0
 0
 0
 0
 0
 0
 0
 0
 0
 0
 0
 0
 0
 0
 0
 0
 0
 0
 2.6959259620554E-03

 0
 0
 0
 0
 0
 0
 0
 0
 0
 0
 2.18694972889638E-04
 0
 0
 0
 0
 0
 0
 0
 0
 0
 0
 0
 0
 0
 0
 0
 0
 0
 0
 0
 0

 0
 0
 0
 0
 0
 0
 0
 0
 0
 0
 2.18694972889638E-02
 0
 0
 0
 0
 0
 0
 0
 0
 0
 0
 0
 0
 0
 0
 0
 0
 0
 0
 0
 0

 0
 0
 0
 0
 0
 0
 0
 0
 0
 0
 1.15102617310526E-04

 0
 0
 0
 0
 0
 0
 0
 0
 0
 0
 0
 0
 0
 0
 0
 0
 0
 0
 0
 0
 0
 0
 0
 0
 0
 0
 0
 0
 0
 0
 0
 8.15156423233013E-04

 0
 0
 0
 0
 0
 0
 0
 0
 0
 0
 0
 .018869115078914
 0
 0
 0
 0
 0
 0
 0
 0
 0
 0
 0
 0
 0
 0
 0
 0
 0
 0
 0
 0

 0
 0
 0
 0
 0
 0
 0
 0
 0
 0
 0
 0
 0
 0
 0
 0
 0
 0
 0
 0
 0
 0
 0
 0

 0
 0
 0
 0
 0
 0
 0
 0
 0
 0
 0
 0
 2.25905977744946E-04

 0
 0
 0
 0
 0
 0
 0
 0
 0
 0
 0
 0
 2.69581133441431E-02

 0
 0
 0
 0
 0
 0
 0
 0
 0
 0
 0
 0
 0

 0
 0
 0
 0
 0
 0
 0
 0
 0
 0
 0
 0
 0
 3.34233301382159E-04

 0
 0
 0
 0
 0
 0
 0
 0
 0
 0
 0
 0
 0
 8.35583253454018E-03

 0
 0
 0
 0
 0
 0
 0
 0
 0
 0
 0
 0
 0
 0
 0
 0
 0
 0
 0
 0
 0
 0
 0
 0
 0

 0
 0
 0
 0
 0
 0
 0
 0
 0
 0
 0
 0
 0
 0
 1.65006043956849E-04

 0
 0
 0
 0
 0
 0
 0
 0
 0
 0
 0
 0
 0
 0
 2.18633008242846E-02

 0
 0
 0
 0
 0
 0
 0
 0
 0
 0
 0
 0
 0
 0
 0

 0
 0
 0
 0
 0
 0
 0
 0
 0
 0
 0
 0
 0
 0
 0
 1.16429281623376E-04

 0
 0
 0
 0
 0
 0
 0
 0
 0
 0
 0
 0
 0
 0
 0
 1.01293475012126E-02

 0
 0
 0
 0
 0
 0
 0
 0
 0
 0
 0
 0
 0
 0
 0
 0
 0
 0
 0
 0
 0
 0
 0
 0
 0

 0
 0
 0
 0
 0
 0
 0
 0
 0
 0
 0
 0
 0
 0
 0
 0
 0

 0
 0
 0
 0
 0
 0
 0
 0
 0
 0
 0
 0
 0
 0
 0
 0
 2.05922770223607E-02

 0
 0
 0
 0
 0
 0
 0
 0
 0
 0
 0
 0
 0
 0
 0
 0
 0

 0
 0
 0
 0
 0
 0
 0
 0
 0
 0
 0
 0
 0
 0
 0
 0
 0
 6.32224376568604E-04

 0
 0
 0
 0
 0
 0
 0
 0
 0
 0
 0
 0
 0
 0
 0
 0
 0
 1.58056094142151E-02

 0
 0
 0
 0
 0
 0
 0
 0
 0
 0
 0
 0
 0
 0
 0
 0
 0
 0
 0
 0
 0
 0
 0
 0
 0
 4.43472902817423E-04

 0
 0
 0
 0
 0
 0
 0
 0
 0
 0
 0
 0
 0
 0
 0
 0
 0
 0
 0

 0
 0
 0
 0
 0
 0
 0
 0
 0
 0
 0
 0
 0
 0
 0
 0
 0
 0
 1.89675618229308E-02

 0
 0
 0
 0
 0
 0
 0
 0
 0
 0
 0
 0
 0
 0
 0
 0
 0
 0
 0

 0
 0
 0
 0
 0
 0
 0
 0
 0
 0
 0
 0
 0
 0
 0
 0
 0
 0
 0
 9.97477091516534E-05

 0
 0
 0
 0
 0
 0
 0
 0
 0
 0
 0
 0
 0
 0
 0
 0
 0
 0
 0
 1.25682113530787E-02

 0
 0
 0
 0
 0
 0
 0
 0
 0
 0
 0
 0
 0
 0
 0
 0
 0
 0
 0
 0
 0
 0
 0
 0
 0
 0

 0
 0
 0
 0
 0
 0
 0
 0
 0
 0
 0
 0
 0
 0
 0
 0
 0
 0
 0
 0
 0
 0
 0
 0
 7.2033727784937E-04
 0
 0
 0
 0
 0
 0
 0

 0
 0
 0
 0
 0
 0
 0
 0
 0
 0
 0
 0
 0
 0
 0
 0
 0
 0
 0
 0
 0

 0
 0
 0
 0
 0
 0
 0
 0
 0
 0
 0
 0
 0
 0
 0
 0
 0
 0
 0
 0
 0

 0
 0
 0
 0
 0
 0
 0
 0
 0
 0
 0
 0
 0
 0
 0
 0
 0
 0
 0
 0
 0
 0
 0
 0
 0
 0
 0

 0
 0
 0
 0
 0
 0
 0
 0
 0
 0
 0
 0
 0
 0
 0
 0
 0
 0
 0
 0
 0
 0
 0
 0
 0
 0
 0

 0
 0
 0
 0
 0
 0
 0
 0
 0
 0
 0
 0
 0
 0
 0
 0
 0
 0
 0
 0
 0
 0
 0
 0
 0
 0
 0
 0

 0
 0
 0
 0
 0
 0
 0
 0
 0
 0
 0
 0
 0
 0
 0
 0
 0
 0
 0
 0
 0
 0
 0
 0
 0
 0
 0
 0

 0
 0
 0
 0
 0
 0
 0
 0
 0
 0
 0
 0
 0
 0
 0
 0
 0
 0
 0
 0
 0
 0
 0
 0
 0
 0
 0
 0
 1.10364539730991E-03

 0
 0
 0
 0
 0
 0
 0
 0
 0
 0
 0
 0
 0
 0
 0
 0
 0
 0
 0
 0
 0
 0
 0
 0
 0
 0
 0
 0
 0

 0
 0
 0
 0
 0
 0
 0
 0
 0
 0
 0
 0
 0
 0
 0
 0
 0
 0
 0
 0
 0
 0
 0
 0
 0
 0
 0
 0
 0
 0

 0
 0
 0
 0
 0
 0
 0
 0
 0
 0
 0
 0
 0
 0
 0
 0
 0
 0
 0
 0
 0
 0
 0
 0
 0
 0
 0
 0
 0
 0

 0
 0
 0
 0
 0
 0
 0
 0
 0
 0
 0
 0
 0
 0
 0
 0
 0
 0
 0
 0
 0
 0
 0
 0
 0
 0
 0
 0
 0
 0
 0

 0
 0
 0
 0
 0
 0
 0
 0
 0
 0
 0
 0
 0
 0
 0
 0
 0
 0
 0
 0
 0
 0
 0
 0
 0
 0
 0
 0
 0
 0
 0

 0
 0
 0
 0
 0
 0
 0
 0
 0
 0
 0
 0
 0
 0
 0
 0
 0
 0
 0
 0
 0
 0
 0
 0
 0
 0
 0
 0
 0
 0
 0
 0

 0
 0
 0
 0
 0
 0
 0
 0
 0
 0
 0
 0
 0
 0
 0
 0
 0
 0
 0
 0
 0
 0
 0
 0
 0
 0
 0
 0
 0
 0
 0
 0

 9.58872689782066E-02
 9.68685218653032E-02
 .100332643317181
 .270813690348267
 .116764108393175
 7.72791112262429E-02
 .250731521160291
 .296294356609558
 .199768275147766
 .245302523670448
 .232860268326914
 .180316806685896
 .127315853141701
 .335746146851914
 .192124932023412
 .17858209182008
 .217314529967003
 .254564590641961
 .272760194298392
 .375149384158926
 .299898620380659
 .351142384206349
 .161369393739446
 .130617612861373
 .471843327484415
 .1692218685004
 .244985932839733
 .306822001769422
 .182292789092437
 .143594225204634
 .157858029615308
 .310058331516474

 5.22831346665668E-04
 2.01893542862383E-04
 8.3750119630492E-05
 1.58148616180895E-03
 1.4108193047615E-03
 1.05572556320134E-03
 1.58428588270781E-03
 1.64279417060097E-03
 4.50189919155191E-04
 1.15418376883582E-03
 4.60410469242104E-04
 8.05225394548608E-04
 1.10971357488606E-03
 1.75912263885637E-04
 6.0791700405112E-04
 3.67671415652362E-04
 2.02580196973553E-03
 8.31874179692785E-04
 1.3310569700317E-04
 6.29985531484126E-04
 0
 0
 0
 0
 2.4011242594954E-04
 0
 1.52716236928266E-03
 1.75122715103412E-04
 0
 0
 0
 0

 0

 .095364437631541
 9.66666283224408E-02
 .10024889319755
 .269232204186458
 .115353289088413
 7.62233856630416E-02
 .249147235277583
 .294504677313115
 .199204037115758
 .244148339901612
 .232399857857672
 .179511581291347
 .126206139566815
 .335570234588028
 .191517015019361
 .178214420404428
 .215288727997268
 .253732716462268
 .272627088601389
 .374519398627442
 .299898620380659
 .351142384206349
 .155140060525225
 .130617612861373
 .468683447958919
 .1692218685004
 .24345877047045
 .306114506000404
 .181733608757799
 .143200995925207
 .157858029615308
 .309895300231827

 0
 0
 0
 0
 0
 0
 0
 1.46885125842112E-04
 0
 0
 0
 0
 0
 0
 0
 0
 0
 0
 0
 0
 0
 0
 0
 0
 2.91976709954641E-03
 0
 0
 5.32373053914374E-04
 5.59180334637899E-04
 3.93229279427505E-04
 0
 1.63031284647169E-04

 0
 0

 0
 0
 0

 0
 0
 0
 0

 0
 0
 0
 0
 0

 0
 0
 0
 0
 0
 0

 0
 0
 0
 0
 0
 0
 0

 0
 0
 0
 0
 0
 0
 0
 0

 0
 0
 0
 0
 0
 0
 0
 0
 1.14048112852648E-04

 0
 0
 0
 0
 0
 0
 0
 0
 0
 0

 0
 0
 0
 0
 0
 0
 0
 0
 0
 0
 0

 0
 0
 0
 0
 0
 0
 0
 0
 0
 0
 0
 0

 0
 0
 0
 0
 0
 0
 0
 0
 0
 0
 0
 0
 0

 0
 0
 0
 0
 0
 0
 0
 0
 0
 0
 0
 0
 0
 0

 0
 0
 0
 0
 0
 0
 0
 0
 0
 0
 0
 0
 0
 0
 0

 0
 0
 0
 0
 0
 0
 0
 0
 0
 0
 0
 0
 0
 0
 0
 0

 0
 0
 0
 0
 0
 0
 0
 0
 0
 0
 0
 0
 0
 0
 0
 0
 0

 0
 0
 0
 0
 0
 0
 0
 0
 0
 0
 0
 0
 0
 0
 0
 0
 0
 0

 0
 0
 0
 0
 0
 0
 0
 0
 0
 0
 0
 0
 0
 0
 0
 0
 0
 0
 0

 0
 0
 0
 0
 0
 0
 0
 0
 0
 0
 0
 0
 0
 0
 0
 0
 0
 0
 0
 0

 0
 0
 0
 0
 0
 0
 0
 0
 0
 0
 0
 0
 0
 0
 0
 0
 0
 0
 0
 0
 0
 0
 0
 0
 0
 0
 0
 0
 0
 0
 0
 0

 0
 0
 0
 0
 0
 0
 0
 0
 0
 0
 0
 0
 0
 0
 0
 0
 0
 0
 0
 0
 0
 0
 6.22933321422045E-03
 0
 0
 0
 0
 0
 0
 0
 0
 0

 0
 0
 0
 0
 0
 0
 0
 4.6482634760162E-05
 1.44364699813479E-04
 9.99318604917547E-04
 6.64389791058226E-04
 2.1948337758561E-03
 5.71913867708725E-04
 1.52308846199437E-03
 1.12788941438838E-03
 1.53273990997668E-03
 1.41293276623318E-03
 8.80312423068887E-04
 8.96357351968599E-04
 3.5353618433414E-04
 1.04005070359357E-04
 9.56369328225467E-04
 5.63230851194141E-04
 3.24193628348855E-04
 0
 1.06658039918114E-04
 4.72232848368251E-04
 5.89653698958078E-04
 7.07823208404068E-04
 0
 1.89853007745398E-04
 1.54776536056961E-04

 0
 0
 0
 0
 0
 0
 0
 4.6482634760162E-05
 1.44364699813479E-04
 9.99318604917547E-04
 6.64389791058226E-04
 2.1948337758561E-03
 5.71913867708725E-04
 1.52308846199437E-03
 1.12788941438838E-03
 1.53273990997668E-03
 1.41293276623318E-03
 8.80312423068887E-04
 8.96357351968599E-04
 3.5353618433414E-04
 1.04005070359357E-04
 9.56369328225467E-04
 5.63230851194141E-04
 3.24193628348855E-04
 0
 1.06658039918114E-04
 4.72232848368251E-04
 5.89653698958078E-04
 7.07823208404068E-04
 0
 1.89853007745398E-04
 1.54776536056961E-04

 0
 0
 0
 0
 0
 0
 0
 0
 0
 0
 0
 0
 0
 0
 0
 0
 0
 0
 0
 0
 0
 0
 0
 0
 0
 1.10868225704356E-04
 0
 0
 0
 0
 0
 0

 0
 0
 0
 0
 0
 0
 0
 0
 0
 0
 0
 0
 0
 0
 0
 0
 0
 0
 0
 0
 0
 0
 0
 0
 0
 1.10868225704356E-04
 0
 0
 0
 0
 0
 0

 2.36518942539231E-04
 1.6439902775946E-04
 2.27321753282764E-04
 2.86173686422887E-04
 2.68055667904369E-04
 4.40852432985734E-05
 3.30784964520739E-04
 2.62294867574853E-04
 8.14629377518917E-05
 1.25311380616875E-04
 0
 1.45707452346891E-04
 0
 9.54952289664888E-05
 1.41433751963091E-04
 1.99593054211282E-04
 5.49860534643408E-05
 2.70953304243389E-04
 7.22573783731493E-05
 0
 1.17377150834132E-04
 0
 6.35646246347673E-04
 3.65875666279422E-04
 0
 0
 3.55299000200691E-04
 0
 3.99414524741826E-04
 0
 0
 2.32901835209702E-04

 4.73037885078462E-05
 1.0959935183958E-04
 4.54643506565528E-05
 5.72347372845775E-05
 7.65873336870527E-05
 0
 0
 1.57376920544947E-04
 0
 0
 0
 7.28537261734455E-05
 0
 0
 0
 0
 0
 0
 0
 0
 0
 0
 3.81387747808288E-04
 0
 0
 0
 0
 0
 0
 0
 0
 5.82254588025605E-05

 4.73037885078462E-05
 1.0959935183958E-04
 4.54643506565528E-05
 5.72347372845775E-05
 7.65873336870527E-05
 0
 0
 1.57376920544947E-04
 0
 0
 0
 7.28537261734455E-05
 0
 0
 0
 0
 0
 0
 0
 0
 0
 0
 3.81387747808288E-04
 0
 0
 0
 0
 0
 0
 0
 0
 5.82254588025605E-05

 1.89215154031385E-04
 5.47996759198803E-05
 1.81857402626211E-04
 2.2893894913831E-04
 1.91468334217316E-04
 4.40852432985734E-05
 3.30784964520739E-04
 1.04917947029907E-04
 8.14629377518917E-05
 1.25311380616875E-04
 0
 7.28537261734455E-05
 0
 9.54952289664888E-05
 1.41433751963091E-04
 1.99593054211282E-04
 5.49860534643408E-05
 2.70953304243389E-04
 7.22573783731493E-05
 0
 1.17377150834132E-04
 0
 2.54258498539385E-04
 3.65875666279422E-04
 0
 0
 3.55299000200691E-04
 0
 3.99414524741826E-04
 0
 0
 1.74676376407142E-04

 0
 0
 0
 0
 0
 0
 0
 0
 4.07314688759458E-05
 0
 0
 7.28537261734455E-05
 0
 9.54952289664888E-05
 1.41433751963091E-04
 1.99593054211282E-04
 5.49860534643408E-05
 2.70953304243389E-04
 7.22573783731493E-05
 0
 0
 0
 2.54258498539385E-04
 0
 0
 0
 1.18433000066897E-04
 0
 0
 0
 0
 5.82254588025605E-05

 1.89215154031385E-04
 0
 0
 0
 0
 0
 0
 0
 0
 0
 0
 0
 0
 0
 0
 0
 0
 0
 0
 0
 1.17377150834132E-04

 0
 5.47996759198803E-05
 0
 0
 0
 0
 0
 0
 0
 0
 0
 0
 0
 0
 0
 0
 0
 0
 0
 0
 0
 0

 0
 0
 1.81857402626211E-04
 0
 0
 0
 0
 0
 0
 0
 0
 0
 0
 0
 0
 0
 0
 0
 0
 0
 0
 0
 0

 0
 0
 0
 2.2893894913831E-04
 0
 0
 0
 0
 0
 0
 0
 0
 0
 0
 0
 0
 0
 0
 0
 0
 0
 0
 0
 3.65875666279422E-04

 0
 0
 0
 0
 1.91468334217316E-04
 0
 0
 0
 0
 0
 0
 0
 0
 0
 0
 0
 0
 0
 0
 0
 0
 0
 0
 0
 0

 0
 0
 0
 0
 0
 4.40852432985734E-05
 0
 0
 0
 0
 0
 0
 0
 0
 0
 0
 0
 0
 0
 0
 0
 0
 0
 0
 0
 0

 0
 0
 0
 0
 0
 0
 3.30784964520739E-04
 0
 0
 0
 0
 0
 0
 0
 0
 0
 0
 0
 0
 0
 0
 0
 0
 0
 0
 0
 2.36866000133794E-04

 0
 0
 0
 0
 0
 0
 0
 1.04917947029907E-04
 0
 0
 0
 0
 0
 0
 0
 0
 0
 0
 0
 0
 0
 0
 0
 0
 0
 0
 0
 0

 0
 0
 0
 0
 0
 0
 0
 0
 4.07314688759458E-05
 0
 0
 0
 0
 0
 0
 0
 0
 0
 0
 0
 0
 0
 0
 0
 0
 0
 0
 0
 3.99414524741826E-04

 0
 0
 0
 0
 0
 0
 0
 0
 0
 1.25311380616875E-04
 0
 0
 0
 0
 0
 0
 0
 0
 0
 0
 0
 0
 0
 0
 0
 0
 0
 0
 0
 0

 0
 0
 0
 0
 0
 0
 0
 0
 0
 0
 0
 0
 0
 0
 0
 0
 0
 0
 0
 0
 0
 0
 0
 0
 0
 0
 0
 0
 0
 0
 0

 0
 0
 0
 0
 0
 0
 0
 0
 0
 0
 0
 0
 0
 0
 0
 0
 0
 0
 0
 0
 0
 0
 0
 0
 0
 0
 0
 0
 0
 0
 0
 1.16450917604581E-04

 0
 0
 0
 0
 0
 0
 0
 0
 0
 0
 0
 0
 0

 0
 0
 0
 0
 0
 0
 0
 0
 0
 0
 0
 0
 0
 0

 0
 0
 0
 0
 0
 0
 0
 0
 0
 0
 0
 0
 0
 0
 0

 0
 0
 0
 0
 0
 0
 0
 0
 0
 0
 0
 0
 0
 0
 0
 0

 0
 0
 0
 0
 0
 0
 0
 0
 0
 0
 0
 0
 0
 0
 0
 0
 0

 0
 0
 0
 0
 0
 0
 0
 0
 0
 0
 0
 0
 0
 0
 0
 0
 0
 0

 0
 0
 0
 0
 0
 0
 0
 0
 0
 0
 0
 0
 0
 0
 0
 0
 0
 0
 0

 0
 0
 0
 0
 0
 0
 0
 0
 0
 0
 0
 0
 0
 0
 0
 0
 0
 0
 0
 0

 0
 0
 0
 0
 0
 0
 0
 0
 0
 1.75435932863625E-04
 0
 0
 0
 0
 0
 0
 0
 0
 0
 0

 0
 0
 0
 0
 0
 0
 0
 0
 0
 1.75435932863625E-04
 0
 0
 0
 0
 0
 0
 0
 0
 0
 0

 0
 0
 0
 0
 0
 0
 0
 0
 0
 1.75435932863625E-04
 0
 0
 0
 0
 0
 0
 0
 0
 0
 0

 1.40222090145007E-02
 7.70585011796339E-03
 .012088030197837
 1.02440311680934E-02
 1.38429955050219E-02
 8.49172996778377E-03
 7.01035997221039E-03
 4.80233637997417E-02
 4.21640051701228E-02
 3.54955288302059E-02
 2.85434645651072E-02
 5.21340636446894E-02
 3.98814932435997E-02
 5.45376545565596E-02
 4.80345396151929E-02
 8.16711262557011E-02
 8.43915046163353E-02
 8.73995698500352E-02
 7.46853197225971E-02
 .100354794347034
 4.44818926781448E-03
 .136628429807363
 .253300645402852
 .125147613945629
 2.39807110580297E-02
 6.20599229788575E-03
 1.41292611027999E-02
 2.15172223272275E-02
 .147923685722078
 3.05091682314714E-03
 3.79485065870337E-03
 7.55565677810412E-02

 3.31126519554377E-04
 5.75396597158109E-04
 1.11387659108292E-03
 1.40225106346884E-03
 1.47430617347599E-03
 6.17193406180028E-04
 4.63098950329799E-04
 5.50819221907314E-04
 1.56816155172156E-03
 1.31576949647574E-03
 1.09347486444639E-03
 4.3347967073158E-03
 4.51811955489892E-04
 2.67386641106169E-03
 2.6400967033085E-03
 3.49287844869744E-03
 2.30941424550231E-03
 6.95446814224421E-03
 3.54061154027597E-03
 1.59596334642645E-03
 2.46492016751971E-03
 2.05072908809281E-02
 1.82430472701815E-02
 5.12225932790526E-03
 4.56213609304126E-03
 0
 2.69435075151326E-03
 2.99459842827738E-03
 6.29077876468294E-03
 0
 5.6243953544574E-04
 5.70609496262826E-03

 3.31126519554377E-04
 5.75396597158109E-04
 1.11387659108292E-03
 1.40225106346884E-03
 1.47430617347599E-03
 6.17193406180028E-04
 4.63098950329799E-04
 5.50819221907314E-04
 1.56816155172156E-03
 1.31576949647574E-03
 1.09347486444639E-03
 4.3347967073158E-03
 4.51811955489892E-04
 2.67386641106169E-03
 2.6400967033085E-03
 3.49287844869744E-03
 2.30941424550231E-03
 6.95446814224421E-03
 3.54061154027597E-03
 1.59596334642645E-03
 2.46492016751971E-03
 2.05072908809281E-02
 1.82430472701815E-02
 5.12225932790526E-03
 4.56213609304126E-03
 0
 2.69435075151326E-03
 2.99459842827738E-03
 6.29077876468294E-03
 0
 5.6243953544574E-04
 5.70609496262826E-03

 1.03477037360914E-04
 0
 0
 0
 0
 0
 0
 0
 0
 0
 0
 0
 0
 0
 0
 0
 0
 0
 0
 0
 0

 1.03477037360914E-04
 0
 0
 0
 0
 0
 0
 0
 0
 0
 0
 0
 0
 0
 0
 0
 0
 0
 0
 0
 0

 1.35876054575854E-02
 7.01057922973055E-03
 1.09741536067541E-02
 8.8417801046246E-03
 1.22011545391055E-02
 7.87453656160374E-03
 6.54726102188059E-03
 4.73577905732703E-02
 4.05067435302351E-02
 3.41797593337302E-02
 2.74499897006609E-02
 4.74805318853653E-02
 3.90531713252012E-02
 5.18637881454979E-02
 4.52913141344112E-02
 7.78871746029457E-02
 8.13603984191135E-02
 .080445101707791
 7.09866451671298E-02
 9.85094617277291E-02
 1.98326910029477E-03
 .116121138926435
 .23505759813267
 .119225001597737
 1.85631744475423E-02
 5.8110242438145E-03
 1.14349103512866E-02
 1.78987492263942E-02
 .140759187684522
 3.05091682314714E-03
 3.23241112325763E-03
 .069850472818413

 1.35876054575854E-02
 7.01057922973055E-03
 1.09741536067541E-02
 8.8417801046246E-03
 1.22011545391055E-02
 7.87453656160374E-03
 6.54726102188059E-03
 4.73577905732703E-02
 4.05067435302351E-02
 3.41797593337302E-02
 2.74499897006609E-02
 4.74805318853653E-02
 3.90531713252012E-02
 5.18637881454979E-02
 4.52913141344112E-02
 7.78871746029457E-02
 8.13603984191135E-02
 .080445101707791
 7.09866451671298E-02
 9.85094617277291E-02
 1.98326910029477E-03
 .116121138926435
 .23505759813267
 .119225001597737
 1.85631744475423E-02
 5.8110242438145E-03
 1.14349103512866E-02
 1.78987492263942E-02
 .140759187684522
 3.05091682314714E-03
 3.23241112325763E-03
 .069850472818413

 0
 1.19874291074738E-04
 0
 0
 0
 0
 0
 0
 0
 0
 0
 0
 0
 0
 0
 0
 0
 0
 0
 0
 0
 0

 0
 1.19874291074738E-04
 0
 0
 0
 0
 0
 0
 0
 0
 0
 0
 0
 0
 0
 0
 0
 0
 0
 0
 0
 0

 0
 0
 0
 0
 0
 0
 0
 0
 0
 0
 0
 0
 0
 0
 0
 0
 0
 0
 0
 0
 0
 0
 0

 0
 0
 0
 0
 0
 0
 0
 0
 0
 0
 0
 0
 0
 0
 0
 0
 0
 0
 0
 0
 0
 0
 0

 0
 0
 0
 0
 0
 0
 0
 0
 0
 0
 0
 0
 0
 0
 0
 0
 0
 0
 0
 0
 0
 0
 0
 8.00353019986237E-04

 0
 0
 0
 0
 0
 0
 0
 0
 0
 0
 0
 0
 0
 0
 0
 0
 0
 0
 0
 0
 0
 0
 0
 8.00353019986237E-04

 0
 0
 0
 0
 1.67534792440428E-04
 0
 0
 0
 0
 0
 0
 0
 0
 0
 0
 0
 0
 0
 0
 0
 0
 0
 0
 0
 8.55400517446127E-04

 0
 0
 0
 0
 1.67534792440428E-04
 0
 0
 0
 0
 0
 0
 0
 0
 0
 0
 0
 0
 0
 0
 0
 0
 0
 0
 0
 8.55400517446127E-04

 0
 0
 0
 0
 0
 0
 0
 0
 0
 0
 0
 0
 0
 0
 0
 0
 0
 0
 0
 0
 0
 0
 0
 0
 0
 3.94968054071247E-04

 0
 0
 0
 0
 0
 0
 0
 0
 0
 0
 0
 0
 0
 0
 0
 0
 0
 0
 0
 0
 0
 0
 0
 0
 0
 3.94968054071247E-04

 0
 0
 0
 0
 0
 0
 0
 0
 0
 0
 0
 0
 0
 0
 0
 0
 0
 0
 0
 0
 0
 0
 0
 0
 0
 0
 0

 0
 0
 0
 0
 0
 0
 0
 0
 0
 0
 0
 0
 0
 0
 0
 0
 0
 0
 0
 0
 0
 0
 0
 0
 0
 0
 0

 0
 0
 0
 0
 0
 0
 0
 1.1475400456415E-04
 0
 0
 0
 0
 0
 0
 0
 0
 0
 0
 0
 0
 0
 0
 0
 0
 0
 0
 0
 6.23874672555907E-04

 0
 0
 0
 0
 0
 0
 0
 1.1475400456415E-04
 0
 0
 0
 0
 0
 0
 0
 0
 0
 0
 0
 0
 0
 0
 0
 0
 0
 0
 0
 6.23874672555907E-04

 0
 0
 0
 0
 0
 0
 0
 0
 8.91000881661315E-05
 0
 0
 0
 0
 0
 0
 0
 0
 0
 0
 0
 0
 0
 0
 0
 0
 0
 0
 0
 8.73719272872745E-04

 0
 0
 0
 0
 0
 0
 0
 0
 8.91000881661315E-05
 0
 0
 0
 0
 0
 0
 0
 0
 0
 0
 0
 0
 0
 0
 0
 0
 0
 0
 0
 8.73719272872745E-04

 0
 0
 0
 0
 0
 0
 0
 0
 0
 0
 0
 0
 0
 0
 0
 0
 0
 0
 0
 0
 0
 0
 0
 0
 0
 0
 0
 0
 0
 0

 0
 0
 0
 0
 0
 0
 0
 0
 0
 0
 0
 0
 0
 0
 0
 0
 0
 0
 0
 0
 0
 0
 0
 0
 0
 0
 0
 0
 0
 0

 0
 0
 0
 0
 0
 0
 0
 0
 0
 0
 0
 0
 0
 0
 0
 0
 0
 0
 0
 0
 0
 0
 0
 0
 0
 0
 0
 0
 0
 0
 0

 0
 0
 0
 0
 0
 0
 0
 0
 0
 0
 0
 0
 0
 0
 0
 0
 0
 0
 0
 0
 0
 0
 0
 0
 0
 0
 0
 0
 0
 0
 0

 0
 0
 0
 0
 0
 0
 0
 0
 0
 0
 0
 3.18735052008298E-04
 0
 0
 0
 0
 0
 0
 0
 0
 0
 0
 0
 0
 0
 0
 0
 0
 0
 0
 0
 0

 0
 0
 0
 0
 0
 0
 0
 0
 0
 0
 0
 3.18735052008298E-04
 0
 0
 0
 0
 0
 0
 0
 0
 0
 0
 0
 0
 0
 0
 0
 0
 0
 0
 0
 0

 0
 0
 0
 0
 0
 0
 0
 0
 0
 0
 0
 0
 3.76509962908658E-04

 0
 0
 0
 0
 0
 0
 0
 0
 0
 0
 0
 0
 3.76509962908658E-04

 0
 0
 0
 0
 0
 0
 0
 0
 0
 0
 0
 0
 0
 0

 0
 0
 0
 0
 0
 0
 0
 0
 0
 0
 0
 0
 0
 0

 0
 0
 0
 0
 0
 0
 0
 0
 0
 0
 0
 0
 0
 0
 1.03128777473201E-04

 0
 0
 0
 0
 0
 0
 0
 0
 0
 0
 0
 0
 0
 0
 1.03128777473201E-04

 0
 0
 0
 0
 0
 0
 0
 0
 0
 0
 0
 0
 0
 0
 0
 2.9107320405796E-04

 0
 0
 0
 0
 0
 0
 0
 0
 0
 0
 0
 0
 0
 0
 0
 2.9107320405796E-04

 0
 0
 0
 0
 0
 0
 0
 0
 0
 0
 0
 0
 0
 0
 0
 0
 7.21691951719473E-04

 0
 0
 0
 0
 0
 0
 0
 0
 0
 0
 0
 0
 0
 0
 0
 0
 7.21691951719473E-04

 0
 0
 0
 0
 0
 0
 0
 0
 0
 0
 0
 0
 0
 0
 0
 0
 0
 0

 0
 0
 0
 0
 0
 0
 0
 0
 0
 0
 0
 0
 0
 0
 0
 0
 0
 0

 0
 0
 0
 0
 0
 0
 0
 0
 0
 0
 0
 0
 0
 0
 0
 0
 0
 0
 1.58063015191264E-04

 0
 0
 0
 0
 0
 0
 0
 0
 0
 0
 0
 0
 0
 0
 0
 0
 0
 0
 1.58063015191264E-04

 0
 0
 0
 0
 0
 0
 0
 0
 0
 0
 0
 0
 0
 0
 0
 0
 0
 0
 0
 2.49369272878722E-04

 0
 0
 0
 0
 0
 0
 0
 0
 0
 0
 0
 0
 0
 0
 0
 0
 0
 0
 0
 2.49369272878722E-04

 0
 0
 0
 0
 0
 0
 0
 6.55737168938E-05
 1.52743008284797E-04
 0
 7.81053474607141E-05
 9.10671577168068E-05
 1.61361412674962E-04
 1.19369036208111E-04
 1.76792189953864E-04
 8.31637725881258E-05
 2.06197700491051E-04
 2.257944202027E-04
 0
 7.12483636797524E-05
 0
 0
 0
 0
 0
 5.06398112000459E-04
 4.48124865117989E-04
 1.34891821093413E-03
 3.77824550431457E-04
 9.2993410675388E-04
 4.84446751527837E-04
 3.1079805712059E-04

 0
 0
 0
 0
 0
 0
 0
 6.55737168938E-05
 1.52743008284797E-04
 0
 7.81053474607141E-05
 9.10671577168068E-05
 1.61361412674962E-04
 1.19369036208111E-04
 1.76792189953864E-04
 8.31637725881258E-05
 2.06197700491051E-04
 2.257944202027E-04
 0
 7.12483636797524E-05
 0
 0
 0
 0
 0
 5.06398112000459E-04
 4.48124865117989E-04
 1.34891821093413E-03
 3.77824550431457E-04
 9.2993410675388E-04
 4.84446751527837E-04
 3.1079805712059E-04

 0
 0
 0
 0
 0
 0
 0
 6.55737168938E-05
 1.52743008284797E-04
 0
 7.81053474607141E-05
 9.10671577168068E-05
 1.61361412674962E-04
 1.19369036208111E-04
 1.76792189953864E-04
 8.31637725881258E-05
 2.06197700491051E-04
 2.257944202027E-04
 0
 7.12483636797524E-05
 0
 0
 0
 0
 0
 5.06398112000459E-04
 4.48124865117989E-04
 1.34891821093413E-03
 3.77824550431457E-04
 9.2993410675388E-04
 4.84446751527837E-04
 3.1079805712059E-04

 0
 0
 0
 0
 0
 0
 0
 0
 5.09143360949323E-05
 0
 7.81053474607141E-05
 0
 1.07574275116582E-04
 0
 0
 0
 1.37465133660625E-04
 0
 0
 0

 0
 0
 0
 0
 0
 0
 0
 6.55737168938E-05
 5.09143360949323E-05
 0
 0
 9.10671577168068E-05
 0
 1.19369036208111E-04
 5.89307299846863E-05
 8.31637725881258E-05
 0
 2.257944202027E-04
 0
 7.12483636797524E-05
 0
 0
 0
 0
 0
 2.21736451408419E-04
 0
 0
 0
 0
 1.31564803613039E-04
 0

 0
 0
 0
 0
 0
 0
 0
 0
 0
 0
 0
 0
 0
 0
 0
 0
 0
 0
 0
 0
 0

 0
 0
 0
 0
 0
 0
 0
 0
 0
 0
 0
 0
 0
 0
 0
 0
 0
 0
 0
 0
 0

 0
 0
 0
 0
 0
 0
 0
 0
 0
 0
 0
 0
 0
 0
 0
 0
 0
 0
 0
 0
 0
 0

 0
 0
 0
 0
 0
 0
 0
 0
 0
 0
 0
 0
 0
 0
 0
 0
 0
 0
 0
 0
 0
 0

 0
 0
 0
 0
 0
 0
 0
 0
 0
 0
 0
 0
 0
 0
 0
 0
 0
 0
 0
 0
 0
 0
 0

 0
 0
 0
 0
 0
 0
 0
 0
 0
 0
 0
 0
 0
 0
 0
 0
 0
 0
 0
 0
 0
 0
 0

 0
 0
 0
 0
 0
 0
 0
 0
 0
 0
 0
 0
 0
 0
 0
 0
 0
 0
 0
 0
 0
 0
 0
 0

 0
 0
 0
 0
 0
 0
 0
 0
 0
 0
 0
 0
 0
 0
 0
 0
 0
 0
 0
 0
 0
 0
 0
 0

 0
 0
 0
 0
 0
 0
 0
 0
 0
 0
 0
 0
 0
 0
 0
 0
 0
 0
 0
 0
 0
 0
 0
 0
 0

 0
 0
 0
 0
 0
 0
 0
 0
 0
 0
 0
 0
 0
 0
 0
 0
 0
 0
 0
 0
 0
 0
 0
 0
 0

 0
 0
 0
 0
 0
 0
 0
 0
 0
 0
 0
 0
 0
 0
 0
 0
 0
 0
 0
 0
 0
 0
 0
 0
 0
 2.8466166059204E-04

 0
 0
 0
 0
 0
 0
 0
 0
 0
 0
 0
 0
 0
 0
 0
 0
 0
 0
 0
 0
 0
 0
 0
 0
 0
 0

 0
 0
 0
 0
 0
 0
 0
 0
 0
 0
 0
 0
 0
 0
 0
 0
 0
 0
 0
 0
 0
 0
 0
 0
 0
 0
 4.48124865117989E-04

 0
 0
 0
 0
 0
 0
 0
 0
 0
 0
 0
 0
 0
 0
 0
 0
 0
 0
 0
 0
 0
 0
 0
 0
 0
 0
 0

 0
 0
 0
 0
 0
 0
 0
 0
 0
 0
 0
 0
 0
 0
 0
 0
 0
 0
 0
 0
 0
 0
 0
 0
 0
 0
 0
 1.34891821093413E-03

 0
 0
 0
 0
 0
 0
 0
 0
 0
 0
 0
 0
 0
 0
 0
 0
 0
 0
 0
 0
 0
 0
 0
 0
 0
 0
 0
 0

 0
 0
 0
 0
 0
 0
 0
 0
 0
 0
 0
 0
 0
 0
 0
 0
 0
 0
 0
 0
 0
 0
 0
 0
 0
 0
 0
 0
 3.77824550431457E-04

 0
 0
 0
 0
 0
 0
 0
 0
 5.09143360949323E-05
 0
 0
 0
 0
 0
 0
 0
 0
 0
 0
 0
 0
 0
 0
 0
 0
 0
 0
 0
 0

 0
 0
 0
 0
 0
 0
 0
 0
 0
 0
 0
 0
 0
 0
 0
 0
 0
 0
 0
 0
 0
 0
 0
 0
 0
 0
 0
 0
 0
 9.2993410675388E-04

 0
 0
 0
 0
 0
 0
 0
 0
 0
 0
 0
 0
 0
 0
 0
 0
 0
 0
 0
 0
 0
 0
 0
 0
 0
 0
 0
 0
 0
 0

 0
 0
 0
 0
 0
 0
 0
 0
 0
 0
 0
 0
 0
 0
 0
 0
 0
 0
 0
 0
 0
 0
 0
 0
 0
 0
 0
 0
 0
 0
 1.52010685255605E-04

 0
 0
 0
 0
 0
 0
 0
 0
 0
 0
 0
 0
 0
 0
 0
 0
 0
 0
 0
 0
 0
 0
 0
 0
 0
 0
 0
 0
 0
 0
 2.00871262659193E-04

 0
 0
 0
 0
 0
 0
 0
 0
 0
 0
 0
 0
 0
 0
 0
 0
 0
 0
 0
 0
 0
 0
 0
 0
 0
 0
 0
 0
 0
 0
 0
 1.65234410114864E-04

 0
 0
 0
 0
 0
 0
 0
 0
 0
 0
 0
 0
 0
 0
 0
 0
 0
 0
 0
 0
 0
 0
 0
 0
 0
 0
 0
 0
 0
 0
 0
 1.45563647005726E-04

 0
 0
 0
 0
 0
 0
 0
 0
 0
 0
 0
 0
 5.37871375583797E-05

 0
 0
 0
 0
 0
 0
 0
 0
 0
 0
 0
 0
 0

 0
 0
 0
 0
 0
 0
 0
 0
 0
 0
 0
 0
 0
 0

 0
 0
 0
 0
 0
 0
 0
 0
 0
 0
 0
 0
 0
 0

 0
 0
 0
 0
 0
 0
 0
 0
 0
 0
 0
 0
 0
 0
 0

 0
 0
 0
 0
 0
 0
 0
 0
 0
 0
 0
 0
 0
 0
 1.17861459969178E-04

 0
 0
 0
 0
 0
 0
 0
 0
 0
 0
 0
 0
 0
 0
 0
 0

 0
 0
 0
 0
 0
 0
 0
 0
 0
 0
 0
 0
 0
 0
 0
 0

 0
 0
 0
 0
 0
 0
 0
 0
 0
 0
 0
 0
 0
 0
 0
 0
 0

 0
 0
 0
 0
 0
 0
 0
 0
 0
 0
 0
 0
 0
 0
 0
 0
 6.8732566830426E-05

 0
 0
 0
 0
 0
 0
 0
 0
 0
 0
 0
 0
 0
 0
 0
 0
 0
 0

 0
 0
 0
 0
 0
 0
 0
 0
 0
 0
 0
 0
 0
 0
 0
 0
 0
 0

 0
 0
 0
 0
 0
 0
 0
 0
 0
 0
 0
 0
 0
 0
 0
 0
 0
 0
 0

 0
 0
 0
 0
 0
 0
 0
 0
 0
 0
 0
 0
 0
 0
 0
 0
 0
 0
 0

 0
 0
 0
 0
 0
 0
 0
 0
 0
 0
 0
 0
 0
 0
 0
 0
 0
 0
 0
 0

 0
 0
 0
 0
 0
 0
 0
 0
 0
 0
 0
 0
 0
 0
 0
 0
 0
 0
 0
 0

 0
 0
 0
 0
 0
 0
 0
 0
 0
 0
 1.34581521778239E-04
 7.84578589560182E-05
 4.63396877426041E-05
 0
 0
 2.14946366073689E-04
 1.18431499769154E-04
 9.72652887028622E-05
 2.33446914743764E-04
 1.22766411263371E-04
 0
 0
 0
 0
 0
 0
 0
 1.02379433445072E-04
 0
 0
 0
 0

 0
 0
 0
 0
 0
 0
 0
 0
 0
 0
 1.34581521778239E-04
 7.84578589560182E-05
 4.63396877426041E-05
 0
 0
 2.14946366073689E-04
 1.18431499769154E-04
 9.72652887028622E-05
 2.33446914743764E-04
 1.22766411263371E-04
 0
 0
 0
 0
 0
 0
 0
 1.02379433445072E-04
 0
 0
 0
 0

 0
 0
 0
 0
 0
 0
 0
 0
 0
 0
 1.34581521778239E-04
 7.84578589560182E-05
 4.63396877426041E-05
 0
 0
 2.14946366073689E-04
 1.18431499769154E-04
 9.72652887028622E-05
 2.33446914743764E-04
 1.22766411263371E-04
 0
 0
 0
 0
 0
 0
 0
 1.02379433445072E-04
 0
 0
 0
 0

 0
 0
 0
 0
 0
 0
 0
 0
 0
 0
 1.34581521778239E-04
 7.84578589560182E-05
 4.63396877426041E-05
 0
 0
 2.14946366073689E-04
 1.18431499769154E-04
 9.72652887028622E-05
 2.33446914743764E-04
 1.22766411263371E-04
 0
 0
 0
 0
 0
 0
 0
 1.02379433445072E-04
 0
 0
 0
 0

 1.13529092418721E-03
 8.21995138798023E-04
 1.40939487035134E-03
 1.5453379066817E-03
 3.10178701432545E-03
 4.54078005974287E-03
 1.65392482260784E-03
 4.82622556338454E-03
 4.56192451409585E-03
 1.17792697779899E-02
 1.11846857563598E-02
 2.90686367431723E-02
 2.49572318270456E-02
 4.02989866238154E-02
 1.84806769231696E-02
 4.29125066553115E-02
 2.26542540272757E-02
 6.43062508737785E-02
 .033166136673124
 3.03233035821064E-02
 3.16918307251904E-03
 6.32179643697296E-02
 4.88176317195082E-02
 5.85401066046791E-03
 2.99797514686016E-03
 1.68519703070145E-03
 8.94169150507738E-03
 8.19474236559146E-02
 3.99414524741826E-03
 1.40439028366966E-04
 1.01774773080606E-03
 4.71043961711117E-02

 8.98771981648296E-04
 6.57596111038563E-04
 1.18207311706887E-03
 1.20192948297518E-03
 2.91031868010775E-03
 1.85158021853572E-03
 1.3892968509906E-03
 3.46229225199437E-03
 2.15876785041908E-03
 2.50622761233336E-03
 2.81179250858055E-03
 9.98096048576082E-03
 1.20052891030112E-02
 9.74051335457239E-03
 7.6845671899883E-03
 9.51393558406073E-03
 1.07222804255328E-02
 1.49927495014572E-02
 7.58702472917471E-03
 6.55484945852311E-03
 2.58229731834838E-03
 1.14100716179478E-02
 1.62725439064764E-02
 4.75638366163107E-03
 2.60693491031336E-03
 1.08334094830876E-03
 1.06589700060372E-03
 8.17572904225129E-03
 2.99560893556487E-03
 1.40439028366966E-04
 3.21394020254709E-04
 1.14121899252565E-02

 8.98771981648296E-04
 6.57596111038563E-04
 1.18207311706887E-03
 1.20192948297518E-03
 2.91031868010775E-03
 1.85158021853572E-03
 1.3892968509906E-03
 3.46229225199437E-03
 2.15876785041908E-03
 2.50622761233336E-03
 2.81179250858055E-03
 9.98096048576082E-03
 1.20052891030112E-02
 9.74051335457239E-03
 7.6845671899883E-03
 9.51393558406073E-03
 1.07222804255328E-02
 1.49927495014572E-02
 7.58702472917471E-03
 6.55484945852311E-03
 2.58229731834838E-03
 1.14100716179478E-02
 1.62725439064764E-02
 4.75638366163107E-03
 2.60693491031336E-03
 1.08334094830876E-03
 1.06589700060372E-03
 8.17572904225129E-03
 2.99560893556487E-03
 1.40439028366966E-04
 3.21394020254709E-04
 1.14121899252565E-02

 8.98771981648296E-04
 6.57596111038563E-04
 1.18207311706887E-03
 1.20192948297518E-03
 2.91031868010775E-03
 1.85158021853572E-03
 1.3892968509906E-03
 3.46229225199437E-03
 2.15876785041908E-03
 2.50622761233336E-03
 2.81179250858055E-03
 9.98096048576082E-03
 1.20052891030112E-02
 9.74051335457239E-03
 7.6845671899883E-03
 9.51393558406073E-03
 1.07222804255328E-02
 1.49927495014572E-02
 7.58702472917471E-03
 6.55484945852311E-03
 2.58229731834838E-03
 1.14100716179478E-02
 1.62725439064764E-02
 4.75638366163107E-03
 2.60693491031336E-03
 1.08334094830876E-03
 1.06589700060372E-03
 8.17572904225129E-03
 2.99560893556487E-03
 1.40439028366966E-04
 3.21394020254709E-04
 1.14121899252565E-02

 2.36518942538919E-04
 1.6439902775946E-04
 2.27321753282464E-04
 3.4340842370652E-04
 1.91468334217695E-04
 2.68919984120716E-03
 2.64627971617246E-04
 1.36393331139017E-03
 2.40315666367677E-03
 9.27304216565658E-03
 8.37289324777927E-03
 1.90876762574114E-02
 1.29519427240344E-02
 .030558473269243
 1.07961097331813E-02
 3.33985710712508E-02
 1.19319736017429E-02
 4.93135013723213E-02
 2.55791119439493E-02
 2.37684541235833E-02
 5.86885754170659E-04
 5.18078927517818E-02
 3.25450878130318E-02
 1.09762699883684E-03
 3.91040236546801E-04
 6.01856082392697E-04
 7.87579450447366E-03
 7.37716946136633E-02
 9.98536311853392E-04
 0
 6.96353710551351E-04
 3.56922062458551E-02

 1.41911365523382E-04
 5.47996759198803E-05
 9.09287013129554E-05
 5.72347372845775E-05
 7.65873336870527E-05
 2.51285886801286E-03
 1.9847097871288E-04
 5.245897351504E-05
 5.70240564262569E-04
 2.50622761233336E-04
 6.24842779685713E-05
 1.23851334494737E-03
 0
 2.86485686899151E-04
 0
 3.99186108423004E-04
 0
 5.41906608487375E-04
 0
 0
 0
 3.39218345398561E-03
 1.94507751382385E-02
 0
 0
 0
 0
 0
 0
 0
 0
 6.98705505626407E-04

 9.46075770155362E-05
 0
 9.09287013129554E-05
 0
 7.65873336870527E-05
 2.46877362471429E-03
 0
 5.245897351504E-05
 5.29509095386624E-04
 2.50622761233336E-04
 6.24842779685713E-05
 1.23851334494737E-03
 0
 2.86485686899151E-04
 0
 3.99186108423004E-04
 0
 5.41906608487375E-04
 0
 0
 0
 3.39218345398561E-03
 1.94507751382385E-02
 0
 0
 0
 0
 0
 0
 0
 0
 6.98705505626407E-04

 4.73037885078462E-05
 5.47996759198803E-05
 0
 5.72347372845775E-05
 0
 4.40852432985734E-05
 1.9847097871288E-04
 0
 4.07314688759458E-05
 0
 0
 0
 0
 0
 0
 0
 0
 0
 0
 0

 9.46075770155362E-05
 0
 0
 0
 0
 0
 0
 0
 0
 0
 0
 0
 0
 0
 0
 0
 0
 0
 0
 0
 5.86885754170659E-04

 9.46075770155362E-05
 0
 0
 0
 0
 0
 0
 0
 0
 0
 0
 0
 0
 0
 0
 0
 0
 0
 0
 0
 5.86885754170659E-04

 0
 1.0959935183958E-04
 0
 0
 0
 0
 0
 0
 0
 0
 0
 0
 0
 0
 0
 0
 0
 0
 0
 0
 0
 4.84157092977962E-02

 0
 1.0959935183958E-04
 0
 0
 0
 0
 0
 0
 0
 0
 0
 0
 0
 0
 0
 0
 0
 0
 0
 0
 0
 4.84157092977962E-02

 0
 0
 1.36393051969508E-04
 0
 0
 0
 0
 0
 0
 0
 0
 0
 0
 0
 0
 0
 0
 0
 0
 0
 0
 0
 1.30943126747933E-02

 0
 0
 1.36393051969508E-04
 0
 0
 0
 0
 0
 0
 0
 0
 0
 0
 0
 0
 0
 0
 0
 0
 0
 0
 0
 1.30943126747933E-02

 0
 0
 0
 2.86173686421943E-04
 0
 0
 0
 0
 0
 0
 0
 0
 0
 0
 0
 0
 0
 0
 0
 0
 0
 0
 0
 1.09762699883684E-03

 0
 0
 0
 2.86173686421943E-04
 0
 0
 0
 0
 0
 0
 0
 0
 0
 0
 0
 0
 0
 0
 0
 0
 0
 0
 0
 1.09762699883684E-03

 0
 0
 0
 0
 1.14881000530642E-04
 0
 0
 0
 0
 0
 0
 0
 0
 0
 0
 0
 0
 0
 0
 0
 0
 0
 0
 0
 3.91040236546801E-04

 0
 0
 0
 0
 1.14881000530642E-04
 0
 0
 0
 0
 0
 0
 0
 0
 0
 0
 0
 0
 0
 0
 0
 0
 0
 0
 0
 3.91040236546801E-04

 0
 0
 0
 0
 0
 1.76340973194294E-04
 0
 0
 0
 0
 0
 0
 0
 0
 0
 0
 0
 0
 0
 0
 0
 0
 0
 0
 0
 6.01856082392697E-04

 0
 0
 0
 0
 0
 1.76340973194294E-04
 0
 0
 0
 0
 0
 0
 0
 0
 0
 0
 0
 0
 0
 0
 0
 0
 0
 0
 0
 6.01856082392697E-04

 0
 0
 0
 0
 0
 0
 6.61569929043662E-05
 0
 0
 0
 0
 0
 0
 0
 0
 0
 0
 0
 0
 0
 0
 0
 0
 0
 0
 0
 7.87579450447366E-03

 0
 0
 0
 0
 0
 0
 6.61569929043662E-05
 0
 0
 0
 0
 0
 0
 0
 0
 0
 0
 0
 0
 0
 0
 0
 0
 0
 0
 0
 7.87579450447366E-03

 0
 0
 0
 0
 0
 0
 0
 1.31147433787513E-03
 0
 0
 0
 0
 0
 0
 0
 0
 0
 0
 0
 0
 0
 0
 0
 0
 0
 0
 0
 7.37716946136633E-02

 0
 0
 0
 0
 0
 0
 0
 1.31147433787513E-03
 0
 0
 0
 0
 0
 0
 0
 0
 0
 0
 0
 0
 0
 0
 0
 0
 0
 0
 0
 7.37716946136633E-02

 0
 0
 0
 0
 0
 0
 0
 0
 1.8329160994142E-03
 0
 0
 0
 0
 0
 0
 0
 0
 0
 0
 0
 0
 0
 0
 0
 0
 0
 0
 0
 9.98536311853392E-04

 0
 0
 0
 0
 0
 0
 0
 0
 1.8329160994142E-03
 0
 0
 0
 0
 0
 0
 0
 0
 0
 0
 0
 0
 0
 0
 0
 0
 0
 0
 0
 9.98536311853392E-04

 0
 0
 0
 0
 0
 0
 0
 0
 0
 9.02241940442325E-03
 0
 0
 0
 0
 0
 0
 0
 0
 0
 0
 0
 0
 0
 0
 0
 0
 0
 0
 0
 0

 0
 0
 0
 0
 0
 0
 0
 0
 0
 9.02241940442325E-03
 0
 0
 0
 0
 0
 0
 0
 0
 0
 0
 0
 0
 0
 0
 0
 0
 0
 0
 0
 0

 0
 0
 0
 0
 0
 0
 0
 0
 0
 0
 8.3104089698107E-03
 0
 0
 0
 0
 0
 0
 0
 0
 0
 0
 0
 0
 0
 0
 0
 0
 0
 0
 0
 6.96353710551351E-04

 0
 0
 0
 0
 0
 0
 0
 0
 0
 0
 8.3104089698107E-03
 0
 0
 0
 0
 0
 0
 0
 0
 0
 0
 0
 0
 0
 0
 0
 0
 0
 0
 0
 6.96353710551351E-04

 0
 0
 0
 0
 0
 0
 0
 0
 0
 0
 0
 1.78491629124641E-02
 0
 0
 0
 0
 0
 0
 0
 0
 0
 0
 0
 0
 0
 0
 0
 0
 0
 0
 0
 3.49935007402287E-02

 0
 0
 0
 0
 0
 0
 0
 0
 0
 0
 0
 1.78491629124641E-02
 0
 0
 0
 0
 0
 0
 0
 0
 0
 0
 0
 0
 0
 0
 0
 0
 0
 0
 0
 3.49935007402287E-02

 0
 0
 0
 0
 0
 0
 0
 0
 0
 0
 0
 0
 1.29519427240344E-02

 0
 0
 0
 0
 0
 0
 0
 0
 0
 0
 0
 0
 1.29519427240344E-02

 0
 0
 0
 0
 0
 0
 0
 0
 0
 0
 0
 0
 0
 3.02719875823438E-02

 0
 0
 0
 0
 0
 0
 0
 0
 0
 0
 0
 0
 0
 3.02719875823438E-02

 0
 0
 0
 0
 0
 0
 0
 0
 0
 0
 0
 0
 0
 0
 1.07961097331813E-02

 0
 0
 0
 0
 0
 0
 0
 0
 0
 0
 0
 0
 0
 0
 1.07961097331813E-02

 0
 0
 0
 0
 0
 0
 0
 0
 0
 0
 0
 0
 0
 0
 0
 3.29993849628278E-02

 0
 0
 0
 0
 0
 0
 0
 0
 0
 0
 0
 0
 0
 0
 0
 3.29993849628278E-02

 0
 0
 0
 0
 0
 0
 0
 0
 0
 0
 0
 0
 0
 0
 0
 0
 1.19319736017429E-02

 0
 0
 0
 0
 0
 0
 0
 0
 0
 0
 0
 0
 0
 0
 0
 0
 1.19319736017429E-02

 0
 0
 0
 0
 0
 0
 0
 0
 0
 0
 0
 0
 0
 0
 0
 0
 0
 4.87715947638339E-02

 0
 0
 0
 0
 0
 0
 0
 0
 0
 0
 0
 0
 0
 0
 0
 0
 0
 4.87715947638339E-02

 0
 0
 0
 0
 0
 0
 0
 0
 0
 0
 0
 0
 0
 0
 0
 0
 0
 0
 2.55791119439493E-02

 0
 0
 0
 0
 0
 0
 0
 0
 0
 0
 0
 0
 0
 0
 0
 0
 0
 0
 2.55791119439493E-02

 0
 0
 0
 0
 0
 0
 0
 0
 0
 0
 0
 0
 0
 0
 0
 0
 0
 0
 0
 2.37684541235833E-02

 0
 0
 0
 0
 0
 0
 0
 0
 0
 0
 0
 0
 0
 0
 0
 0
 0
 0
 0
 2.37684541235833E-02

 4.73037885078462E-05
 0
 0
 5.72347372845775E-05
 2.29762001061537E-04
 1.32255729895575E-04
 0
 5.245897351504E-05
 1.62925875503783E-04
 0
 0
 0
 0
 0
 0
 0
 0
 0
 0
 0
 1.17377150834132E-04
 0
 0
 0
 1.30346745515465E-04
 0
 0
 9.50666167704239E-05
 1.99707262370678E-04
 0
 0
 0

 4.73037885078462E-05
 0
 0
 5.72347372845775E-05
 2.29762001061537E-04
 1.32255729895575E-04
 0
 5.245897351504E-05
 1.62925875503783E-04
 0
 0
 0
 0
 0
 0
 0
 0
 0
 0
 0
 1.17377150834132E-04
 0
 0
 0
 1.30346745515465E-04
 0
 0
 9.50666167704239E-05
 1.99707262370678E-04
 0
 0
 0

 4.73037885078462E-05
 0
 0
 5.72347372845775E-05
 2.29762001061537E-04
 1.32255729895575E-04
 0
 5.245897351504E-05
 1.62925875503783E-04
 0
 0
 0
 0
 0
 0
 0
 0
 0
 0
 0
 1.17377150834132E-04
 0
 0
 0
 1.30346745515465E-04
 0
 0
 9.50666167704239E-05
 1.99707262370678E-04
 0
 0
 0

 4.73037885078462E-05
 0
 0
 5.72347372845775E-05
 2.29762001061537E-04
 1.32255729895575E-04
 0
 5.245897351504E-05
 1.62925875503783E-04
 0
 0
 0
 0
 0
 0
 0
 0
 0
 0
 0
 1.17377150834132E-04
 0
 0
 0
 1.30346745515465E-04
 0
 0
 9.50666167704239E-05
 1.99707262370678E-04
 0
 0
 0

 4.73037885078462E-05
 0
 0
 5.72347372845775E-05
 2.29762001061537E-04
 1.32255729895575E-04
 0
 5.245897351504E-05
 1.62925875503783E-04
 0
 0
 0
 0
 0
 0
 0
 0
 0
 0
 0
 1.17377150834132E-04
 0
 0
 0
 1.30346745515465E-04
 0
 0
 9.50666167704239E-05
 1.99707262370678E-04
 0
 0
 0

 3.522622548445E-04
 1.63233077207884E-04
 1.35425725359721E-04
 2.55729677228682E-04
 7.41430570800286E-04
 7.22247602975328E-04
 3.94126766238126E-04
 1.0156950191197E-03
 8.18962512503997E-03
 1.86633971131515E-04
 2.69878902714858E-03
 2.06160544277869E-03
 1.73034365932172E-03
 8.53361620551602E-04
 7.02153378540943E-04
 3.36901751080081E-03
 1.14651771053306E-03
 3.22837979523949E-03
 8.60938976360572E-04
 3.39566669452437E-04
 8.74085165786088E-04
 2.29644914680279E-04
 0
 .012533187717244
 1.94133450767713E-04
 2.15131535834835E-03
 1.41111659654094E-03
 0
 1.07077085356026E-02
 2.71913863434136E-03
 2.55291704032571E-03
 1.73437536857887E-04

 3.522622548445E-04
 0
 0
 0
 0
 0
 0
 0
 0
 0
 0
 0
 0
 0
 0
 0
 0
 0
 0
 0
 8.74085165786088E-04

 3.522622548445E-04
 0
 0
 0
 0
 0
 0
 0
 0
 0
 0
 0
 0
 0
 0
 0
 0
 0
 0
 0
 8.74085165786088E-04

 3.522622548445E-04
 0
 0
 0
 0
 0
 0
 0
 0
 0
 0
 0
 0
 0
 0
 0
 0
 0
 0
 0
 8.74085165786088E-04

 3.522622548445E-04
 0
 0
 0
 0
 0
 0
 0
 0
 0
 0
 0
 0
 0
 0
 0
 0
 0
 0
 0
 8.74085165786088E-04

 0
 0
 0
 8.52432257429877E-05
 6.84397449969407E-04
 2.62635491991284E-04
 1.97063383119063E-04
 0
 3.63983338891231E-04
 0
 0
 0
 3.20434010985034E-04
 0
 2.80861351416377E-04
 0
 3.27576488723732E-04
 0
 2.15234744089877E-04
 0
 0
 0
 0
 .011988266512147
 0
 0
 9.70142660121228E-04
 0
 9.51796314275317E-03
 2.09164510333779E-04
 0
 0

 0
 0
 0
 8.52432257429877E-05
 6.84397449969407E-04
 2.62635491991284E-04
 1.97063383119063E-04
 0
 3.63983338891231E-04
 0
 0
 0
 3.20434010985034E-04
 0
 2.80861351416377E-04
 0
 3.27576488723732E-04
 0
 2.15234744089877E-04
 0
 0
 0
 0
 .011988266512147
 0
 0
 9.70142660121228E-04
 0
 9.51796314275317E-03
 2.09164510333779E-04
 0
 0

 0
 0
 0
 8.52432257429877E-05
 6.84397449969407E-04
 2.62635491991284E-04
 1.97063383119063E-04
 0
 3.63983338891231E-04
 0
 0
 0
 3.20434010985034E-04
 0
 2.80861351416377E-04
 0
 3.27576488723732E-04
 0
 2.15234744089877E-04
 0
 0
 0
 0
 .011988266512147
 0
 0
 9.70142660121228E-04
 0
 9.51796314275317E-03
 2.09164510333779E-04
 0
 0

 0
 0
 0
 0
 5.13298087476961E-04
 1.96976618993409E-04
 0
 0
 2.42655559260954E-04
 0
 0
 0
 3.20434010985034E-04
 0
 2.80861351416377E-04
 0
 3.27576488723732E-04
 0
 2.15234744089877E-04
 0
 0
 0
 0
 .011988266512147
 0
 0
 9.70142660121228E-04
 0
 9.51796314275317E-03
 2.09164510333779E-04
 0
 0

 0
 0
 0
 8.52432257429877E-05
 1.71099362492446E-04
 6.56588729978753E-05
 1.97063383119063E-04
 0
 1.21327779630277E-04
 0
 0
 0
 0
 0
 0
 0
 0
 0
 0
 0

 0
 1.63233077207884E-04
 0
 0
 0
 0
 0
 0
 0
 0
 0
 0
 0
 0
 0
 0
 0
 0
 0
 0
 0
 2.29644914680279E-04

 0
 1.63233077207884E-04
 0
 0
 0
 0
 0
 0
 0
 0
 0
 0
 0
 0
 0
 0
 0
 0
 0
 0
 0
 2.29644914680279E-04

 0
 1.63233077207884E-04
 0
 0
 0
 0
 0
 0
 0
 0
 0
 0
 0
 0
 0
 0
 0
 0
 0
 0
 0
 2.29644914680279E-04

 0
 1.63233077207884E-04
 0
 0
 0
 0
 0
 0
 0
 0
 0
 0
 0
 0
 0
 0
 0
 0
 0
 0
 0
 2.29644914680279E-04

 0
 0
 1.35425725359721E-04
 0
 0
 0
 0
 0
 0
 0
 0
 0
 0
 0
 0
 0
 0
 0
 0
 0
 0
 0
 0

 0
 0
 1.35425725359721E-04
 0
 0
 0
 0
 0
 0
 0
 0
 0
 0
 0
 0
 0
 0
 0
 0
 0
 0
 0
 0

 0
 0
 1.35425725359721E-04
 0
 0
 0
 0
 0
 0
 0
 0
 0
 0
 0
 0
 0
 0
 0
 0
 0
 0
 0
 0

 0
 0
 1.35425725359721E-04
 0
 0
 0
 0
 0
 0
 0
 0
 0
 0
 0
 0
 0
 0
 0
 0
 0
 0
 0
 0

 0
 0
 0
 1.70486451485694E-04
 0
 0
 0
 0
 0
 0
 0
 0
 0
 0
 0
 0
 0
 0
 0
 0
 0
 0
 0
 5.44921205097012E-04

 0
 0
 0
 1.70486451485694E-04
 0
 0
 0
 0
 0
 0
 0
 0
 0
 0
 0
 0
 0
 0
 0
 0
 0
 0
 0
 5.44921205097012E-04

 0
 0
 0
 1.70486451485694E-04
 0
 0
 0
 0
 0
 0
 0
 0
 0
 0
 0
 0
 0
 0
 0
 0
 0
 0
 0
 5.44921205097012E-04

 0
 0
 0
 1.70486451485694E-04
 0
 0
 0
 0
 0
 0
 0
 0
 0
 0
 0
 0
 0
 0
 0
 0
 0
 0
 0
 5.44921205097012E-04

 0
 0
 0
 0
 5.70331208308781E-05
 0
 0
 0
 0
 0
 0
 0
 0
 0
 0
 0
 0
 0
 0
 0
 0
 0
 0
 0
 1.94133450767713E-04

 0
 0
 0
 0
 5.70331208308781E-05
 0
 0
 0
 0
 0
 0
 0
 0
 0
 0
 0
 0
 0
 0
 0
 0
 0
 0
 0
 1.94133450767713E-04

 0
 0
 0
 0
 5.70331208308781E-05
 0
 0
 0
 0
 0
 0
 0
 0
 0
 0
 0
 0
 0
 0
 0
 0
 0
 0
 0
 1.94133450767713E-04

 0
 0
 0
 0
 5.70331208308781E-05
 0
 0
 0
 0
 0
 0
 0
 0
 0
 0
 0
 0
 0
 0
 0
 0
 0
 0
 0
 1.94133450767713E-04

 0
 0
 0
 0
 0
 4.59612110984043E-04
 0
 0
 0
 0
 0
 0
 0
 0
 0
 0
 0
 0
 0
 0
 0
 0
 0
 0
 0
 2.15131535834835E-03

 0
 0
 0
 0
 0
 4.59612110984043E-04
 0
 0
 0
 0
 0
 0
 0
 0
 0
 0
 0
 0
 0
 0
 0
 0
 0
 0
 0
 2.15131535834835E-03

 0
 0
 0
 0
 0
 4.59612110984043E-04
 0
 0
 0
 0
 0
 0
 0
 0
 0
 0
 0
 0
 0
 0
 0
 0
 0
 0
 0
 2.15131535834835E-03

 0
 0
 0
 0
 0
 4.59612110984043E-04
 0
 0
 0
 0
 0
 0
 0
 0
 0
 0
 0
 0
 0
 0
 0
 0
 0
 0
 0
 2.15131535834835E-03

 0
 0
 0
 0
 0
 0
 1.97063383119063E-04
 0
 0
 0
 0
 0
 0
 0
 0
 0
 0
 0
 0
 0
 0
 0
 0
 0
 0
 0
 4.40973936419707E-04

 0
 0
 0
 0
 0
 0
 1.97063383119063E-04
 0
 0
 0
 0
 0
 0
 0
 0
 0
 0
 0
 0
 0
 0
 0
 0
 0
 0
 0
 4.40973936419707E-04

 0
 0
 0
 0
 0
 0
 1.97063383119063E-04
 0
 0
 0
 0
 0
 0
 0
 0
 0
 0
 0
 0
 0
 0
 0
 0
 0
 0
 0
 4.40973936419707E-04

 0
 0
 0
 0
 0
 0
 1.97063383119063E-04
 0
 0
 0
 0
 0
 0
 0
 0
 0
 0
 0
 0
 0
 0
 0
 0
 0
 0
 0
 4.40973936419707E-04

 0
 0
 0
 0
 0
 0
 0
 1.0156950191197E-03
 0
 0
 0
 0
 0
 0
 0
 0
 0
 0
 0
 0
 0
 0
 0
 0
 0
 0
 0
 0

 0
 0
 0
 0
 0
 0
 0
 1.0156950191197E-03
 0
 0
 0
 0
 0
 0
 0
 0
 0
 0
 0
 0
 0
 0
 0
 0
 0
 0
 0
 0

 0
 0
 0
 0
 0
 0
 0
 1.0156950191197E-03
 0
 0
 0
 0
 0
 0
 0
 0
 0
 0
 0
 0
 0
 0
 0
 0
 0
 0
 0
 0

 0
 0
 0
 0
 0
 0
 0
 1.0156950191197E-03
 0
 0
 0
 0
 0
 0
 0
 0
 0
 0
 0
 0
 0
 0
 0
 0
 0
 0
 0
 0

 0
 0
 0
 0
 0
 0
 0
 0
 7.82564178614874E-03
 0
 0
 0
 0
 0
 0
 0
 0
 0
 0
 0
 0
 0
 0
 0
 0
 0
 0
 0
 1.18974539284939E-03

 0
 0
 0
 0
 0
 0
 0
 0
 7.82564178614874E-03
 0
 0
 0
 0
 0
 0
 0
 0
 0
 0
 0
 0
 0
 0
 0
 0
 0
 0
 0
 1.18974539284939E-03

 0
 0
 0
 0
 0
 0
 0
 0
 7.82564178614874E-03
 0
 0
 0
 0
 0
 0
 0
 0
 0
 0
 0
 0
 0
 0
 0
 0
 0
 0
 0
 1.18974539284939E-03

 0
 0
 0
 0
 0
 0
 0
 0
 7.82564178614874E-03
 0
 0
 0
 0
 0
 0
 0
 0
 0
 0
 0
 0
 0
 0
 0
 0
 0
 0
 0
 1.18974539284939E-03

 0
 0
 0
 0
 0
 0
 0
 0
 0
 1.86633971131515E-04
 0
 0
 0
 0
 0
 0
 0
 0
 0
 0
 0
 0
 0
 0
 0
 0
 0
 0
 0
 2.50997412400758E-03

 0
 0
 0
 0
 0
 0
 0
 0
 0
 1.86633971131515E-04
 0
 0
 0
 0
 0
 0
 0
 0
 0
 0
 0
 0
 0
 0
 0
 0
 0
 0
 0
 2.50997412400758E-03

 0
 0
 0
 0
 0
 0
 0
 0
 0
 1.86633971131515E-04
 0
 0
 0
 0
 0
 0
 0
 0
 0
 0
 0
 0
 0
 0
 0
 0
 0
 0
 0
 2.50997412400758E-03

 0
 0
 0
 0
 0
 0
 0
 0
 0
 1.86633971131515E-04
 0
 0
 0
 0
 0
 0
 0
 0
 0
 0
 0
 0
 0
 0
 0
 0
 0
 0
 0
 2.50997412400758E-03

 0
 0
 0
 0
 0
 0
 0
 0
 0
 0
 2.69878902714858E-03
 0
 0
 0
 0
 0
 0
 0
 0
 0
 0
 0
 0
 0
 0
 0
 0
 0
 0
 0
 2.55291704032571E-03

 0
 0
 0
 0
 0
 0
 0
 0
 0
 0
 2.69878902714858E-03
 0
 0
 0
 0
 0
 0
 0
 0
 0
 0
 0
 0
 0
 0
 0
 0
 0
 0
 0
 2.55291704032571E-03

 0
 0
 0
 0
 0
 0
 0
 0
 0
 0
 2.69878902714858E-03
 0
 0
 0
 0
 0
 0
 0
 0
 0
 0
 0
 0
 0
 0
 0
 0
 0
 0
 0
 2.55291704032571E-03

 0
 0
 0
 0
 0
 0
 0
 0
 0
 0
 2.69878902714858E-03
 0
 0
 0
 0
 0
 0
 0
 0
 0
 0
 0
 0
 0
 0
 0
 0
 0
 0
 0
 2.55291704032571E-03

 0
 0
 0
 0
 0
 0
 0
 0
 0
 0
 0
 2.06160544277869E-03
 0
 0
 0
 0
 0
 0
 0
 0
 0
 0
 0
 0
 0
 0
 0
 0
 0
 0
 0
 1.73437536857887E-04

 0
 0
 0
 0
 0
 0
 0
 0
 0
 0
 0
 2.06160544277869E-03
 0
 0
 0
 0
 0
 0
 0
 0
 0
 0
 0
 0
 0
 0
 0
 0
 0
 0
 0
 1.73437536857887E-04

 0
 0
 0
 0
 0
 0
 0
 0
 0
 0
 0
 2.06160544277869E-03
 0
 0
 0
 0
 0
 0
 0
 0
 0
 0
 0
 0
 0
 0
 0
 0
 0
 0
 0
 1.73437536857887E-04

 0
 0
 0
 0
 0
 0
 0
 0
 0
 0
 0
 2.06160544277869E-03
 0
 0
 0
 0
 0
 0
 0
 0
 0
 0
 0
 0
 0
 0
 0
 0
 0
 0
 0
 1.73437536857887E-04

 0
 0
 0
 0
 0
 0
 0
 0
 0
 0
 0
 0
 1.40990964833669E-03

 0
 0
 0
 0
 0
 0
 0
 0
 0
 0
 0
 0
 1.40990964833669E-03

 0
 0
 0
 0
 0
 0
 0
 0
 0
 0
 0
 0
 1.40990964833669E-03

 0
 0
 0
 0
 0
 0
 0
 0
 0
 0
 0
 0
 1.40990964833669E-03

 0
 0
 0
 0
 0
 0
 0
 0
 0
 0
 0
 0
 0
 8.53361620551602E-04

 0
 0
 0
 0
 0
 0
 0
 0
 0
 0
 0
 0
 0
 8.53361620551602E-04

 0
 0
 0
 0
 0
 0
 0
 0
 0
 0
 0
 0
 0
 8.53361620551602E-04

 0
 0
 0
 0
 0
 0
 0
 0
 0
 0
 0
 0
 0
 8.53361620551602E-04

 0
 0
 0
 0
 0
 0
 0
 0
 0
 0
 0
 0
 0
 0
 4.21292027124566E-04

 0
 0
 0
 0
 0
 0
 0
 0
 0
 0
 0
 0
 0
 0
 4.21292027124566E-04

 0
 0
 0
 0
 0
 0
 0
 0
 0
 0
 0
 0
 0
 0
 4.21292027124566E-04

 0
 0
 0
 0
 0
 0
 0
 0
 0
 0
 0
 0
 0
 0
 4.21292027124566E-04

 0
 0
 0
 0
 0
 0
 0
 0
 0
 0
 0
 0
 0
 0
 0
 3.36901751080081E-03

 0
 0
 0
 0
 0
 0
 0
 0
 0
 0
 0
 0
 0
 0
 0
 3.36901751080081E-03

 0
 0
 0
 0
 0
 0
 0
 0
 0
 0
 0
 0
 0
 0
 0
 3.36901751080081E-03

 0
 0
 0
 0
 0
 0
 0
 0
 0
 0
 0
 0
 0
 0
 0
 3.36901751080081E-03

 0
 0
 0
 0
 0
 0
 0
 0
 0
 0
 0
 0
 0
 0
 0
 0
 8.18941221809331E-04

 0
 0
 0
 0
 0
 0
 0
 0
 0
 0
 0
 0
 0
 0
 0
 0
 8.18941221809331E-04

 0
 0
 0
 0
 0
 0
 0
 0
 0
 0
 0
 0
 0
 0
 0
 0
 8.18941221809331E-04

 0
 0
 0
 0
 0
 0
 0
 0
 0
 0
 0
 0
 0
 0
 0
 0
 8.18941221809331E-04

 0
 0
 0
 0
 0
 0
 0
 0
 0
 0
 0
 0
 0
 0
 0
 0
 0
 3.22837979523949E-03

 0
 0
 0
 0
 0
 0
 0
 0
 0
 0
 0
 0
 0
 0
 0
 0
 0
 3.22837979523949E-03

 0
 0
 0
 0
 0
 0
 0
 0
 0
 0
 0
 0
 0
 0
 0
 0
 0
 3.22837979523949E-03

 0
 0
 0
 0
 0
 0
 0
 0
 0
 0
 0
 0
 0
 0
 0
 0
 0
 3.22837979523949E-03

 0
 0
 0
 0
 0
 0
 0
 0
 0
 0
 0
 0
 0
 0
 0
 0
 0
 0
 6.45704232270696E-04

 0
 0
 0
 0
 0
 0
 0
 0
 0
 0
 0
 0
 0
 0
 0
 0
 0
 0
 6.45704232270696E-04

 0
 0
 0
 0
 0
 0
 0
 0
 0
 0
 0
 0
 0
 0
 0
 0
 0
 0
 6.45704232270696E-04

 0
 0
 0
 0
 0
 0
 0
 0
 0
 0
 0
 0
 0
 0
 0
 0
 0
 0
 6.45704232270696E-04

 0
 0
 0
 0
 0
 0
 0
 0
 0
 0
 0
 0
 0
 0
 0
 0
 0
 0
 0
 3.39566669452437E-04

 0
 0
 0
 0
 0
 0
 0
 0
 0
 0
 0
 0
 0
 0
 0
 0
 0
 0
 0
 3.39566669452437E-04

 0
 0
 0
 0
 0
 0
 0
 0
 0
 0
 0
 0
 0
 0
 0
 0
 0
 0
 0
 3.39566669452437E-04

 0
 0
 0
 0
 0
 0
 0
 0
 0
 0
 0
 0
 0
 0
 0
 0
 0
 0
 0
 3.39566669452437E-04

 6.41557631636639E-03
 3.8359773143837E-03
 6.16610255778512E-03
 1.00160790248011E-02
 1.29001790179315E-02
 7.32917169840693E-03
 1.04197263823994E-02
 4.13114416430182E-03
 1.78200176332263E-03
 1.09647458039584E-03
 0
 0
 0
 0
 2.06257554946402E-04
 0
 0
 0
 0
 0
 1.33516509073678E-02
 0
 0
 1.28056483197839E-02
 1.36864082791416E-02
 4.47630461280816E-03
 1.26945371946741E-02
 5.82283027718846E-03
 6.11603491010613E-03
 3.07210374553557E-03
 4.45264632227198E-03
 0

 6.41557631636639E-03
 3.8359773143837E-03
 6.16610255778512E-03
 1.00160790248011E-02
 1.29001790179315E-02
 7.32917169840693E-03
 1.04197263823994E-02
 4.13114416430182E-03
 1.78200176332263E-03
 1.09647458039584E-03
 0
 0
 0
 0
 2.06257554946402E-04
 0
 0
 0
 0
 0
 1.33516509073678E-02
 0
 0
 1.28056483197839E-02
 1.36864082791416E-02
 4.47630461280816E-03
 1.26945371946741E-02
 5.82283027718846E-03
 6.11603491010613E-03
 3.07210374553557E-03
 4.45264632227198E-03
 0

 6.41557631636639E-03
 3.8359773143837E-03
 6.16610255778512E-03
 1.00160790248011E-02
 1.29001790179315E-02
 7.32917169840693E-03
 1.04197263823994E-02
 4.13114416430182E-03
 1.78200176332263E-03
 1.09647458039584E-03
 0
 0
 0
 0
 2.06257554946402E-04
 0
 0
 0
 0
 0
 1.33516509073678E-02
 0
 0
 1.28056483197839E-02
 1.36864082791416E-02
 4.47630461280816E-03
 1.26945371946741E-02
 5.82283027718846E-03
 6.11603491010613E-03
 3.07210374553557E-03
 4.45264632227198E-03
 0

 6.41557631636639E-03
 3.8359773143837E-03
 6.16610255778512E-03
 1.00160790248011E-02
 1.29001790179315E-02
 7.32917169840693E-03
 1.04197263823994E-02
 4.13114416430182E-03
 1.78200176332263E-03
 1.09647458039584E-03
 0
 0
 0
 0
 2.06257554946402E-04
 0
 0
 0
 0
 0
 1.33516509073678E-02
 0
 0
 1.28056483197839E-02
 1.36864082791416E-02
 4.47630461280816E-03
 1.26945371946741E-02
 5.82283027718846E-03
 6.11603491010613E-03
 3.07210374553557E-03
 4.45264632227198E-03
 0

 6.41557631636639E-03
 3.8359773143837E-03
 6.16610255778512E-03
 1.00160790248011E-02
 1.29001790179315E-02
 7.32917169840693E-03
 1.04197263823994E-02
 4.13114416430182E-03
 1.78200176332263E-03
 1.09647458039584E-03
 0
 0
 0
 0
 2.06257554946402E-04
 0
 0
 0
 0
 0
 1.33516509073678E-02
 0
 0
 1.28056483197839E-02
 1.36864082791416E-02
 4.47630461280816E-03
 1.26945371946741E-02
 5.82283027718846E-03
 6.11603491010613E-03
 3.07210374553557E-03
 4.45264632227198E-03
 0

 0
 8.1616538604077E-05
 6.77128626799722E-05
 0
 5.70331208308781E-05
 0
 9.85316915596943E-05
 0
 0
 0
 0
 0
 0
 0
 0
 0
 0
 0
 0
 0

 0

 0

 0

 0

 0
 8.1616538604077E-05

 0
 8.1616538604077E-05

 0
 8.1616538604077E-05

 0
 8.1616538604077E-05

 0
 0
 6.77128626799722E-05

 0
 0
 6.77128626799722E-05

 0
 0
 6.77128626799722E-05

 0
 0
 6.77128626799722E-05

 0
 0
 0
 0

 0
 0
 0
 0

 0
 0
 0
 0

 0
 0
 0
 0

 0
 0
 0
 0
 5.70331208308781E-05

 0
 0
 0
 0
 5.70331208308781E-05

 0
 0
 0
 0
 5.70331208308781E-05

 0
 0
 0
 0
 5.70331208308781E-05

 0
 0
 0
 0
 0
 0

 0
 0
 0
 0
 0
 0

 0
 0
 0
 0
 0
 0

 0
 0
 0
 0
 0
 0

 0
 0
 0
 0
 0
 0
 9.85316915596943E-05

 0
 0
 0
 0
 0
 0
 9.85316915596943E-05

 0
 0
 0
 0
 0
 0
 9.85316915596943E-05

 0
 0
 0
 0
 0
 0
 9.85316915596943E-05

 0
 0
 0
 0
 0
 0
 0
 0

 0
 0
 0
 0
 0
 0
 0
 0

 0
 0
 0
 0
 0
 0
 0
 0

 0
 0
 0
 0
 0
 0
 0
 0

 0
 0
 0
 0
 0
 0
 0
 0
 0

 0
 0
 0
 0
 0
 0
 0
 0
 0

 0
 0
 0
 0
 0
 0
 0
 0
 0

 0
 0
 0
 0
 0
 0
 0
 0
 0

 0
 0
 0
 0
 0
 0
 0
 0
 0
 0

 0
 0
 0
 0
 0
 0
 0
 0
 0
 0

 0
 0
 0
 0
 0
 0
 0
 0
 0
 0

 0
 0
 0
 0
 0
 0
 0
 0
 0
 0

 0
 0
 0
 0
 0
 0
 0
 0
 0
 0
 0

 0
 0
 0
 0
 0
 0
 0
 0
 0
 0
 0

 0
 0
 0
 0
 0
 0
 0
 0
 0
 0
 0

 0
 0
 0
 0
 0
 0
 0
 0
 0
 0
 0

 0
 0
 0
 0
 0
 0
 0
 0
 0
 0
 0
 0

 0
 0
 0
 0
 0
 0
 0
 0
 0
 0
 0
 0

 0
 0
 0
 0
 0
 0
 0
 0
 0
 0
 0
 0

 0
 0
 0
 0
 0
 0
 0
 0
 0
 0
 0
 0

 0
 0
 0
 0
 0
 0
 0
 0
 0
 0
 0
 0
 0

 0
 0
 0
 0
 0
 0
 0
 0
 0
 0
 0
 0
 0

 0
 0
 0
 0
 0
 0
 0
 0
 0
 0
 0
 0
 0

 0
 0
 0
 0
 0
 0
 0
 0
 0
 0
 0
 0
 0

 0
 0
 0
 0
 0
 0
 0
 0
 0
 0
 0
 0
 0
 0

 0
 0
 0
 0
 0
 0
 0
 0
 0
 0
 0
 0
 0
 0

 0
 0
 0
 0
 0
 0
 0
 0
 0
 0
 0
 0
 0
 0

 0
 0
 0
 0
 0
 0
 0
 0
 0
 0
 0
 0
 0
 0

 0
 0
 0
 0
 0
 0
 0
 0
 0
 0
 0
 0
 0
 0
 0

 0
 0
 0
 0
 0
 0
 0
 0
 0
 0
 0
 0
 0
 0
 0

 0
 0
 0
 0
 0
 0
 0
 0
 0
 0
 0
 0
 0
 0
 0

 0
 0
 0
 0
 0
 0
 0
 0
 0
 0
 0
 0
 0
 0
 0

 0
 0
 0
 0
 0
 0
 0
 0
 0
 0
 0
 0
 0
 0
 0
 0

 0
 0
 0
 0
 0
 0
 0
 0
 0
 0
 0
 0
 0
 0
 0
 0

 0
 0
 0
 0
 0
 0
 0
 0
 0
 0
 0
 0
 0
 0
 0
 0

 0
 0
 0
 0
 0
 0
 0
 0
 0
 0
 0
 0
 0
 0
 0
 0

 0
 0
 0
 0
 0
 0
 0
 0
 0
 0
 0
 0
 0
 0
 0
 0
 0

 0
 0
 0
 0
 0
 0
 0
 0
 0
 0
 0
 0
 0
 0
 0
 0
 0

 0
 0
 0
 0
 0
 0
 0
 0
 0
 0
 0
 0
 0
 0
 0
 0
 0

 0
 0
 0
 0
 0
 0
 0
 0
 0
 0
 0
 0
 0
 0
 0
 0
 0

 0
 0
 0
 0
 0
 0
 0
 0
 0
 0
 0
 0
 0
 0
 0
 0
 0
 0

 0
 0
 0
 0
 0
 0
 0
 0
 0
 0
 0
 0
 0
 0
 0
 0
 0
 0

 0
 0
 0
 0
 0
 0
 0
 0
 0
 0
 0
 0
 0
 0
 0
 0
 0
 0

 0
 0
 0
 0
 0
 0
 0
 0
 0
 0
 0
 0
 0
 0
 0
 0
 0
 0

 0
 0
 0
 0
 0
 0
 0
 0
 0
 0
 0
 0
 0
 0
 0
 0
 0
 0
 0

 0
 0
 0
 0
 0
 0
 0
 0
 0
 0
 0
 0
 0
 0
 0
 0
 0
 0
 0

 0
 0
 0
 0
 0
 0
 0
 0
 0
 0
 0
 0
 0
 0
 0
 0
 0
 0
 0

 0
 0
 0
 0
 0
 0
 0
 0
 0
 0
 0
 0
 0
 0
 0
 0
 0
 0
 0

 0
 0
 0
 0
 0
 0
 0
 0
 0
 0
 0
 0
 0
 0
 0
 0
 0
 0
 0
 0

 0
 0
 0
 0
 0
 0
 0
 0
 0
 0
 0
 0
 0
 0
 0
 0
 0
 0
 0
 0

 0
 0
 0
 0
 0
 0
 0
 0
 0
 0
 0
 0
 0
 0
 0
 0
 0
 0
 0
 0

 0
 0
 0
 0
 0
 0
 0
 0
 0
 0
 0
 0
 0
 0
 0
 0
 0
 0
 0
 0

 1.40904901938033E-03
 6.52932308832616E-04
 1.48968297895715E-03
 9.37675483171458E-04
 .003136821645703
 1.51015407895005E-03
 2.95595074678757E-04
 1.71886849389448E-03
 .106647118295095
 2.93015334675832E-02
 .127122269347916
 1.61673268933533E-02
 6.92137463729535E-03
 5.68907747034401E-03
 3.01925952772721E-03
 6.04441376936252E-03
 3.19387076504693E-03
 5.51514881684619E-03
 1.93711269680853E-03
 9.338083409928E-04
 0
 1.1482245734014E-03
 7.19497453312873E-03
 4.35936964077751E-03
 5.82400352303746E-03
 1.34457209896949E-03
 3.96876542777164E-03
 2.59107098048713E-02
 6.24616331244882E-03
 5.85660628934358E-03
 1.75513046522537E-03
 2.35875050127112E-02

 1.40904901938033E-03
 6.52932308832616E-04
 1.48968297895715E-03
 9.37675483171458E-04
 .003136821645703
 1.51015407895005E-03
 2.95595074678757E-04
 1.71886849389448E-03
 .106647118295095
 2.93015334675832E-02
 .127122269347916
 1.61673268933533E-02
 6.92137463729535E-03
 5.68907747034401E-03
 3.01925952772721E-03
 6.04441376936252E-03
 3.19387076504693E-03
 5.51514881684619E-03
 1.93711269680853E-03
 9.338083409928E-04
 0
 1.1482245734014E-03
 7.19497453312873E-03
 4.35936964077751E-03
 5.82400352303746E-03
 1.34457209896949E-03
 3.96876542777164E-03
 2.59107098048713E-02
 6.24616331244882E-03
 5.85660628934358E-03
 1.75513046522537E-03
 2.35875050127112E-02

 1.40904901938033E-03
 6.52932308832616E-04
 1.48968297895715E-03
 9.37675483171458E-04
 .003136821645703
 1.51015407895005E-03
 2.95595074678757E-04
 1.71886849389448E-03
 .106647118295095
 2.93015334675832E-02
 .127122269347916
 1.61673268933533E-02
 6.92137463729535E-03
 5.68907747034401E-03
 3.01925952772721E-03
 6.04441376936252E-03
 3.19387076504693E-03
 5.51514881684619E-03
 1.93711269680853E-03
 9.338083409928E-04
 0
 1.1482245734014E-03
 7.19497453312873E-03
 4.35936964077751E-03
 5.82400352303746E-03
 1.34457209896949E-03
 3.96876542777164E-03
 2.59107098048713E-02
 6.24616331244882E-03
 5.85660628934358E-03
 1.75513046522537E-03
 2.35875050127112E-02

 1.40904901938033E-03
 6.52932308832616E-04
 1.48968297895715E-03
 9.37675483171458E-04
 .003136821645703
 1.51015407895005E-03
 2.95595074678757E-04
 1.71886849389448E-03
 .106647118295095
 2.93015334675832E-02
 .127122269347916
 1.61673268933533E-02
 6.92137463729535E-03
 5.68907747034401E-03
 3.01925952772721E-03
 6.04441376936252E-03
 3.19387076504693E-03
 5.51514881684619E-03
 1.93711269680853E-03
 9.338083409928E-04
 0
 1.1482245734014E-03
 7.19497453312873E-03
 4.35936964077751E-03
 5.82400352303746E-03
 1.34457209896949E-03
 3.96876542777164E-03
 2.59107098048713E-02
 6.24616331244882E-03
 5.85660628934358E-03
 1.75513046522537E-03
 2.35875050127112E-02

 1.40904901938033E-03
 6.52932308832616E-04
 1.48968297895715E-03
 9.37675483171458E-04
 .003136821645703
 1.51015407895005E-03
 2.95595074678757E-04
 1.71886849389448E-03
 .106647118295095
 2.93015334675832E-02
 .127122269347916
 1.61673268933533E-02
 6.92137463729535E-03
 5.68907747034401E-03
 3.01925952772721E-03
 6.04441376936252E-03
 3.19387076504693E-03
 5.51514881684619E-03
 1.93711269680853E-03
 9.338083409928E-04
 0
 1.1482245734014E-03
 7.19497453312873E-03
 4.35936964077751E-03
 5.82400352303746E-03
 1.34457209896949E-03
 3.96876542777164E-03
 2.59107098048713E-02
 6.24616331244882E-03
 5.85660628934358E-03
 1.75513046522537E-03
 2.35875050127112E-02

 .10559015129508
 .115953687311281
 .115481055580491
 .158013547487151
 .171681533746225
 .183098657238437
 .106247673583512
 2.35597285060506E-02
 4.16732306604677E-02
 3.08673663827229E-02
 3.58389090877215E-02
 1.63654798489175E-02
 1.64596719113039E-02
 3.14743529595561E-02
 6.43963869883708E-02
 9.22998617414931E-02
 3.71329425699895E-02
 3.95471567112494E-02
 4.39411880783477E-02
 3.70637874843071E-02
 .170877609551496
 8.08363233241478E-02
 9.41287971266599E-03
 .025013010619843
 .147367299011775
 .380124793901023
 .26498258925834
 4.43199993445897E-02
 2.73295508880309E-02
 .256867974880241
 .282231415668866
 5.40366020143068E-02

 7.08462880354974E-04
 1.22482082669837E-03
 6.53250933116934E-04
 2.77542834093609E-03
 2.68055667904832E-04
 6.17193406179688E-04
 3.98102606424519E-04
 2.44808543069782E-04
 4.90206800857065E-04
 0
 1.45796648593333E-04
 0
 1.00402656775642E-04
 0
 0
 0
 0
 0
 0
 0
 4.32442134652065E-04
 0
 4.44952372443924E-04
 0
 3.39358895342492E-03
 3.36169438532322E-02
 1.58508026186327E-03
 3.32733158696484E-04
 1.63094264269497E-03
 8.51996772093975E-03
 8.01157571014654E-03
 2.03789105808962E-04

 0
 0
 3.35000478521415E-04
 4.21729643148822E-04
 0
 0
 0
 0
 0
 0
 0
 0
 0
 0
 0
 0
 0
 0
 0
 0
 0
 0
 4.44952372443924E-04
 0
 4.8022485189908E-04
 0
 0
 3.32733158696484E-04
 6.98975418297374E-04
 0
 1.8747984514858E-04
 2.03789105808962E-04

 0
 0
 3.35000478521415E-04
 4.21729643148822E-04
 0
 0
 0
 0
 0
 0
 0
 0
 0
 0
 0
 0
 0
 0
 0
 0
 0
 0
 4.44952372443924E-04
 0
 4.8022485189908E-04
 0
 0
 3.32733158696484E-04
 6.98975418297374E-04
 0
 1.8747984514858E-04
 2.03789105808962E-04

 0
 0
 3.35000478521415E-04
 4.21729643148822E-04
 0
 0
 0
 0
 0
 0
 0
 0
 0
 0
 0
 0
 0
 0
 0
 0
 0
 0
 0
 0
 4.8022485189908E-04
 0
 0
 0
 0
 0
 0
 0

 0
 0
 0
 0
 0
 0
 0
 0
 0
 0
 0
 0
 0
 0
 0
 0
 0
 0
 0
 0
 0
 0
 4.44952372443924E-04
 0
 0
 0
 0
 3.32733158696484E-04
 6.98975418297374E-04
 0
 1.8747984514858E-04
 2.03789105808962E-04

 5.22831346665093E-04
 5.85491274301878E-04
 0
 6.11507982566802E-04
 0
 3.08596703090014E-04
 2.43736289647665E-04
 0
 3.00126612769632E-04
 0
 0
 0
 0
 0
 0
 0
 0
 0
 0
 0
 4.32442134652065E-04
 0
 0
 0
 1.39265207050733E-03
 5.85758591961837E-03
 5.14248552924439E-04
 0
 0
 0
 2.80566215289338E-03
 0

 5.22831346665093E-04
 2.01893542862717E-04
 0
 2.10864821574759E-04
 0
 0
 2.43736289647665E-04
 0
 3.00126612769632E-04
 0
 0
 0
 0
 0
 0
 0
 0
 0
 0
 0
 4.32442134652065E-04
 0
 0
 0
 4.8022485189908E-04
 4.87820193099515E-03
 2.18166052755823E-04
 0
 0
 0
 1.18408323251735E-03
 0

 5.22831346665093E-04
 2.01893542862717E-04
 0
 2.10864821574759E-04
 0
 0
 2.43736289647665E-04
 0
 3.00126612769632E-04
 0
 0
 0
 0
 0
 0
 0
 0
 0
 0
 0
 0
 0
 0
 0
 4.8022485189908E-04
 3.32604677113505E-03
 0
 0
 0
 0
 9.8673602709779E-04
 0

 0
 0
 0
 0
 0
 0
 0
 0
 0
 0
 0
 0
 0
 0
 0
 0
 0
 0
 0
 0
 4.32442134652065E-04
 0
 0
 0
 0
 1.5521551598601E-03
 2.18166052755823E-04
 0
 0
 0
 1.97347205419558E-04
 0

 0
 3.83597731439162E-04
 0
 4.00643160992042E-04
 0
 3.08596703090014E-04
 0
 0
 0
 0
 0
 0
 0
 0
 0
 0
 0
 0
 0
 0
 0
 0
 0
 0
 9.12427218608253E-04
 0
 0
 0
 0
 0
 7.4991938059432E-04
 0

 0
 3.83597731439162E-04
 0
 4.00643160992042E-04
 0
 3.08596703090014E-04
 0
 0
 0
 0
 0
 0
 0
 0
 0
 0
 0
 0
 0
 0
 0
 0
 0
 0
 9.12427218608253E-04
 0
 0
 0
 0
 0
 7.4991938059432E-04
 0

 0
 0
 0
 0
 0
 0
 0
 0
 0
 0
 0
 0
 0
 0
 0
 0
 0
 0
 0
 0
 0
 0
 0
 0
 0
 9.02784123591422E-04
 2.96082500168616E-04
 0
 0
 0
 8.03485050636772E-04
 0

 0
 0
 0
 0
 0
 0
 0
 0
 0
 0
 0
 0
 0
 0
 0
 0
 0
 0
 0
 0
 0
 0
 0
 0
 0
 9.02784123591422E-04
 2.96082500168616E-04
 0
 0
 0
 8.03485050636772E-04
 0

 0
 0
 0
 0
 0
 0
 0
 0
 0
 0
 0
 0
 0
 0
 0
 0
 0
 0
 0
 0
 0
 0
 0
 0
 0
 7.65998650317978E-05
 0
 0
 0
 0
 6.81744891449382E-05
 0

 0
 0
 0
 0
 0
 0
 0
 0
 0
 0
 0
 0
 0
 0
 0
 0
 0
 0
 0
 0
 0
 0
 0
 0
 0
 7.65998650317978E-05
 0
 0
 0
 0
 6.81744891449382E-05
 0

 1.10375506518308E-04
 6.39329552396492E-04
 3.18250454595519E-04
 1.46902492363528E-03
 2.68055667904832E-04
 3.08596703089674E-04
 1.54366316776854E-04
 2.44808543069782E-04
 1.90080188087433E-04
 0
 1.45796648593333E-04
 0
 1.00402656775642E-04
 0
 0
 0
 0
 0
 0
 0
 0
 0
 0
 0
 1.5207120310185E-03
 2.65155220299973E-02
 1.07083170893884E-03
 0
 9.31967224397595E-04
 8.51996772093975E-03
 4.92915759536717E-03
 0

 1.10375506518308E-04
 6.39329552396492E-04
 3.18250454595519E-04
 1.46902492363528E-03
 2.68055667904832E-04
 3.08596703089674E-04
 1.54366316776854E-04
 2.44808543069782E-04
 1.90080188087433E-04
 0
 1.45796648593333E-04
 0
 1.00402656775642E-04
 0
 0
 0
 0
 0
 0
 0
 0
 0
 0
 0
 1.5207120310185E-03
 2.65155220299973E-02
 1.07083170893884E-03
 0
 9.31967224397595E-04
 8.51996772093975E-03
 4.92915759536717E-03
 0

 1.10375506518308E-04
 6.39329552396492E-04
 3.18250454595519E-04
 1.46902492363528E-03
 2.68055667904832E-04
 3.08596703089674E-04
 1.54366316776854E-04
 2.44808543069782E-04
 1.90080188087433E-04
 0
 1.45796648593333E-04
 0
 1.00402656775642E-04
 0
 0
 0
 0
 0
 0
 0
 0
 0
 0
 0
 1.5207120310185E-03
 2.02223643684523E-02
 1.38171833412021E-04
 0
 9.31967224397595E-04
 8.51996772093975E-03
 4.24954315670356E-03
 0

 0
 0
 0
 0
 0
 0
 0
 0
 0
 0
 0
 0
 0
 0
 0
 0
 0
 0
 0
 0
 0
 0
 0
 0
 0
 3.02808841455281E-03
 5.18144375292675E-04
 0
 0
 0
 1.17174903217863E-04
 0

 0
 0
 0
 0
 0
 0
 0
 0
 0
 0
 0
 0
 0
 0
 0
 0
 0
 0
 0
 0
 0
 0
 0
 0
 0
 9.21592126166937E-04
 0
 0
 0
 0
 0
 0

 0
 0
 0
 0
 0
 0
 0
 0
 0
 0
 0
 0
 0
 0
 0
 0
 0
 0
 0
 0
 0
 0
 0
 0
 0
 1.68519703070621E-03
 4.1451550023414E-04
 0
 0
 0
 5.6243953544574E-04
 0

 0
 0
 0
 0
 0
 0
 0
 0
 0
 0
 0
 0
 0
 0
 0
 0
 0
 0
 0
 0
 0
 0
 0
 0
 0
 6.58280090119092E-04
 0
 0
 0
 0
 0
 0

 7.52560271715735E-05
 0
 0
 2.73165791585183E-04
 0
 0
 0
 0
 0
 0
 0
 0
 0
 0
 0
 0
 0
 0
 0
 0
 0
 0
 0
 0
 0
 4.01237388263382E-04
 0
 0
 0
 0
 8.92761167374191E-05
 0

 7.52560271715735E-05
 0
 0
 2.73165791585183E-04
 0
 0
 0
 0
 0
 0
 0
 0
 0
 0
 0
 0
 0
 0
 0
 0

 7.52560271715735E-05
 0
 0
 2.73165791585183E-04
 0
 0
 0
 0
 0
 0
 0
 0
 0
 0
 0
 0
 0
 0
 0
 0

 0
 0
 0
 0
 0
 0
 0
 0
 0
 0
 0
 0
 0
 0
 0
 0
 0
 0
 0
 0
 0
 0
 0
 0
 0
 4.01237388263382E-04
 0
 0
 0
 0
 8.92761167374191E-05
 0

 0
 0
 0
 0
 0
 0
 0
 0
 0
 0
 0
 0
 0
 0
 0
 0
 0
 0
 0
 0
 0
 0
 0
 0
 0
 4.01237388263382E-04
 0
 0
 0
 0
 8.92761167374191E-05
 0

 0
 0
 0
 0
 0
 0
 0
 0
 0
 0
 0
 0
 0
 0
 0
 0
 0
 0
 0
 0
 0
 0
 0
 0
 0
 8.42598515353103E-04
 0
 0
 0
 0
 0
 0

 0
 0
 0
 0
 0
 0
 0
 0
 0
 0
 0
 0
 0
 0
 0
 0
 0
 0
 0
 0
 0
 0
 0
 0
 0
 8.42598515353103E-04
 0
 0
 0
 0
 0
 0

 0
 0
 0
 0
 0
 0
 0
 0
 0
 0
 0
 0
 0
 0
 0
 0
 0
 0
 0
 0
 0
 0
 0
 0
 0
 8.42598515353103E-04
 0
 0
 0
 0
 0
 0

 4.16970999176689E-03
 2.60702567350939E-03
 2.87286828737991E-03
 1.11800959835804E-02
 5.21406796262984E-03
 1.43488259021321E-02
 7.81030395087241E-03
 2.89360656808814E-03
 5.25929031925131E-03
 4.38589832159061E-04
 2.18694972889999E-04
 0
 1.00402656775642E-04
 3.34233301382711E-04
 8.25030219785608E-05
 8.15004971361711E-04
 7.69804748500771E-04
 3.16112188284302E-04
 2.52900824306023E-04
 2.9924312745496E-04
 .112123222479326
 3.59777032999104E-04
 3.47486614670019E-03
 1.46808260916237E-02
 3.81698719784231E-02
 .219490456850782
 .103550020691072
 7.04285185907557E-03
 1.03159952613663E-02
 .109212476259213
 .203589024015016
 3.36139097968992E-02

 3.3970814461391E-03
 2.09556203159051E-03
 2.47505521913543E-03
 9.17688017862238E-03
 3.7174238168288E-03
 9.51414422039734E-03
 5.95790814955067E-03
 2.22038307464513E-03
 5.25929031925131E-03
 4.38589832159061E-04
 2.18694972889999E-04
 0
 0
 3.34233301382711E-04
 0
 0
 0
 3.16112188284302E-04
 2.52900824306023E-04
 1.99495418303307E-04
 .111096172409528
 3.59777032999104E-04
 3.47486614670019E-03
 .012119696427671
 3.68012311505107E-02
 .218226559077753
 9.87830924383785E-02
 6.65466317392967E-03
 .009850011649168
 .102330963869235
 .203214064324719
 3.32063315852832E-02

 0
 0
 1.59125227297935E-04
 0
 1.53174667374105E-04
 0
 0
 6.55737168937393E-04
 0
 4.38589832159061E-04
 2.18694972889999E-04
 0
 0
 3.34233301382711E-04
 0
 0
 0
 3.16112188284302E-04
 2.52900824306023E-04
 1.99495418303307E-04
 2.32798015820686E-03
 3.59777032999104E-04
 3.47486614670019E-03
 0
 3.04142406202751E-04
 .14354267565125
 .097085451804847
 6.65466317392967E-03
 0
 9.83073198570072E-04
 .188738638395103
 3.28779757370488E-02

 0
 0
 0
 0
 1.53174667374105E-04
 0
 0
 1.0491794703008E-04
 0
 0
 0
 0
 0
 0
 0
 0
 0
 0
 0
 0
 0
 0
 2.28832648684973E-03
 0
 0
 3.25002284493577E-03
 7.10598000401383E-04
 0
 0
 0
 2.95682498634208E-02
 0

 0
 0
 1.59125227297935E-04
 0
 0
 0
 0
 5.50819221907314E-04
 0
 4.38589832159061E-04
 2.18694972889999E-04
 0
 0
 3.34233301382711E-04
 0
 0
 0
 3.16112188284302E-04
 2.52900824306023E-04
 1.99495418303307E-04
 1.23246008375692E-03
 0
 8.89904744887847E-04
 0
 0
 4.21299257676551E-04
 2.90160850163706E-03
 1.9963989521789E-03
 0
 0
 1.12487907089148E-03
 1.01894552904103E-03

 0
 0
 0
 0
 0
 0
 0
 0
 0
 0
 0
 0
 0
 0
 0
 0
 0
 0
 0
 0
 0
 0
 0
 0
 3.04142406202751E-04
 0
 0
 2.21822105797656E-04
 0
 0
 0
 0

 0
 0
 0
 0
 0
 0
 0
 0
 0
 0
 0
 0
 0
 0
 0
 0
 0
 0
 0
 0
 0
 0
 0
 0
 0
 8.42598515353103E-03
 2.27983525128489E-03
 0
 0
 9.83073198570072E-04
 4.68699612872356E-03
 1.42652374065706E-03

 0
 0
 0
 0
 0
 0
 0
 0
 0
 0
 0
 0
 0
 0
 0
 0
 0
 0
 0
 0
 0
 0
 0
 0
 0
 0
 0
 0
 0
 0
 1.24986563432387E-04
 0

 0
 0
 0
 0
 0
 0
 0
 0
 0
 0
 0
 0
 0
 0
 0
 0
 0
 0
 0
 0
 1.09552007444994E-03
 0
 0
 0
 0
 .131445368395106
 9.11934100515236E-02
 4.43644211595311E-03
 0
 0
 .153233526768635
 3.04325064673507E-02

 0
 0
 0
 0
 0
 0
 0
 0
 0
 0
 0
 0
 0
 0
 0
 0
 0
 0
 0
 0
 0
 3.59777032999104E-04
 2.96634914962616E-04
 0
 0
 0
 0
 0
 0
 0
 0
 0

 3.3970814461391E-03
 2.09556203159051E-03
 2.31592999183749E-03
 9.17688017862238E-03
 3.56424914945469E-03
 9.51414422039734E-03
 5.95790814955067E-03
 1.56464590570773E-03
 5.25929031925131E-03
 0
 0
 0
 0
 0
 0
 0
 0
 0
 0
 0
 .108768192251321
 0
 0
 1.14082715210166E-02
 3.64970887443079E-02
 7.40987455686196E-02
 1.69764063353158E-03
 0
 .009850011649168
 .101347890670664
 1.38504931124534E-02
 3.28355848234389E-04

 0
 0
 0
 0
 8.9351889301709E-05
 0
 0
 0
 0
 0
 0
 0
 0
 0
 0
 0
 0
 0
 0
 0
 2.73880018612974E-04
 0
 0
 0
 0
 1.40433085891629E-03
 1.38171833412021E-04
 0
 0
 0
 3.7495969029716E-04
 0

 4.41502026073231E-04
 3.8359773143874E-04
 1.0608348486529E-04
 4.00643160991601E-04
 9.82870782317324E-04
 2.98310146319837E-03
 1.54366316776854E-04
 3.67212814604876E-04
 6.65280658305547E-04
 0
 0
 0
 0
 0
 0
 0
 0
 0
 0
 0
 0
 0
 0
 4.26854943992105E-03
 9.12427218609202E-03
 4.7747249203287E-03
 4.14515500234781E-04
 0
 6.9897541829847E-03
 7.30751077603749E-02
 7.4991938059432E-04
 0

 1.00777636386043E-03
 5.83735678275609E-04
 8.99403458639359E-04
 6.18384009356851E-03
 7.57548626687441E-04
 4.89729550554895E-03
 3.62425265474763E-03
 1.19743309110286E-03
 4.09085622189052E-03
 0
 0
 0
 0
 0
 0
 0
 0
 0
 0
 0
 .107527676872899
 0
 0
 1.11353463650114E-03
 9.12427218610111E-03
 .064202343398106
 9.01120652687094E-04
 0
 1.21560942312873E-03
 1.90203292766981E-02
 1.20639204704493E-02
 8.86039590473747E-05

 1.94780305620543E-03
 1.12822862187616E-03
 1.31044304833284E-03
 2.59239692406227E-03
 1.73447785114822E-03
 1.63374725165002E-03
 2.17928917802618E-03
 0
 5.03153439055248E-04
 0
 0
 0
 0
 0
 0
 0
 0
 0
 0
 0
 9.6663535980877E-04
 0
 0
 6.02618744459442E-03
 1.82485443721148E-02
 3.71734639126858E-03
 2.43832647197684E-04
 0
 1.64464804305458E-03
 9.25245363359143E-03
 6.61693571112636E-04
 2.39751889187014E-04

 0
 0
 0
 0
 0
 0
 0
 0
 0
 0
 0
 0
 0
 0
 0
 0
 0
 0
 0
 0
 0
 0
 0
 0
 0
 0
 0
 0
 0
 0
 0
 0

 0
 0
 0
 0
 0
 0
 0
 0
 0
 0
 0
 0
 0
 0
 0
 0
 0
 0
 0
 0
 0
 0
 0
 7.11424906654433E-04
 0
 5.85137857883637E-04
 0
 0
 0
 0
 6.24932817161934E-04
 0

 0
 0
 0
 0
 0
 0
 0
 0
 0
 0
 0
 0
 0
 0
 0
 0
 0
 0
 0
 0
 0
 0
 0
 7.11424906654433E-04
 0
 5.85137857883637E-04
 0
 0
 0
 0
 6.24932817161934E-04
 0

 0
 0
 7.95626136489674E-05
 0
 3.35069584880303E-04
 0
 0
 5.5081922190792E-04
 0
 0
 0
 0
 0
 0
 8.25030219785608E-05
 8.15004971361711E-04
 7.69804748500771E-04
 0
 0
 9.97477091516534E-05
 2.05410013959731E-04
 0
 0
 0
 0
 0
 4.76692825269357E-03
 1.66366579348242E-04
 0
 0
 9.373992257429E-05
 4.07578211616034E-04

 0
 0
 7.95626136489674E-05
 0
 3.35069584880303E-04
 0
 0
 5.5081922190792E-04
 0
 0
 0
 0
 0
 0
 8.25030219785608E-05
 8.15004971361711E-04
 7.69804748500771E-04
 0
 0
 9.97477091516534E-05
 2.05410013959731E-04
 0
 0
 0
 0
 0
 4.76692825269357E-03
 1.66366579348242E-04
 0
 0
 9.373992257429E-05
 4.07578211616034E-04

 0
 0
 7.95626136489674E-05
 0
 3.35069584880303E-04
 0
 0
 5.5081922190792E-04
 0
 0
 0
 0
 0
 0
 8.25030219785608E-05
 8.15004971361711E-04
 7.69804748500771E-04
 0
 0
 9.97477091516534E-05
 2.05410013959731E-04
 0
 0
 0
 0
 0
 4.76692825269357E-03
 1.66366579348242E-04
 0
 0
 9.373992257429E-05
 4.07578211616034E-04

 7.7262854562779E-04
 5.11463641918882E-04
 3.18250454595519E-04
 2.00321580495801E-03
 1.16157456092074E-03
 4.83468168173472E-03
 1.85239580132174E-03
 1.22404271535093E-04
 0
 0
 0
 0
 1.00402656775642E-04
 0
 0
 0
 0
 0
 0
 0
 8.21640055837944E-04
 0
 0
 2.56112966395263E-03
 1.36864082791238E-03
 2.10649628838276E-04
 0
 2.21822105797656E-04
 4.6598361219825E-04
 6.88151238997871E-03
 9.373992257429E-05
 0

 7.7262854562779E-04
 5.11463641918882E-04
 3.18250454595519E-04
 2.00321580495801E-03
 1.16157456092074E-03
 4.83468168173472E-03
 1.85239580132174E-03
 1.22404271535093E-04
 0
 0
 0
 0
 1.00402656775642E-04
 0
 0
 0
 0
 0
 0
 0
 8.21640055837944E-04
 0
 0
 2.56112966395263E-03
 1.36864082791238E-03
 2.10649628838276E-04
 0
 2.21822105797656E-04
 4.6598361219825E-04
 6.88151238997871E-03
 9.373992257429E-05
 0

 4.41502026073231E-04
 5.11463641918882E-04
 3.18250454595519E-04
 5.34190881322723E-04
 0
 2.05731135393003E-04
 1.54366316776854E-03
 1.22404271535093E-04
 0
 0
 0
 0
 1.00402656775642E-04
 0
 0
 0
 0
 0
 0
 0

 3.31126519554559E-04
 0
 0
 1.46902492363528E-03
 1.16157456092074E-03
 4.62895054634172E-03
 3.08732633553199E-04
 0
 0
 0
 0
 0
 0
 0
 0
 0
 0
 0
 0
 0
 8.21640055837944E-04
 0
 0
 2.56112966395263E-03
 0
 0
 0
 2.21822105797656E-04
 4.6598361219825E-04
 0
 0
 0

 0
 0
 0
 0
 0
 0
 0
 0
 0
 0
 0
 0
 0
 0
 0
 0
 0
 0
 0
 0
 0
 0
 0
 0
 1.36864082791238E-03
 2.10649628838276E-04
 0
 0
 0
 6.88151238997871E-03
 9.373992257429E-05
 0

 0
 0
 0
 0
 0
 0
 0
 0
 0
 0
 0
 0
 0
 0
 0
 0
 0
 0
 0
 0
 0
 0
 0
 0
 0
 1.05324814419055E-03
 0
 0
 0
 0
 1.8747984514858E-04
 0

 0
 0
 0
 0
 0
 0
 0
 0
 0
 0
 0
 0
 0
 0
 0
 0
 0
 0
 0
 0
 0
 0
 0
 0
 0
 1.05324814419055E-03
 0
 0
 0
 0
 1.8747984514858E-04
 0

 0
 0
 0
 0
 0
 0
 0
 0
 0
 0
 0
 0
 0
 0
 0
 0
 0
 0
 0
 0
 0
 0
 0
 0
 0
 1.05324814419055E-03
 0
 0
 0
 0
 1.8747984514858E-04
 0

 9.97506391431143E-02
 .111357600020494
 .111070871067611
 .142991830706882
 .160405043794828
 .166938706198815
 9.62297998137894E-02
 1.17075450339665E-02
 1.22619539696054E-02
 3.10386650450805E-03
 9.35840026440244E-04
 1.60112149202837E-03
 2.98890985939414E-03
 0
 1.84045202874979E-03
 1.5092684654838E-03
 3.47892530572048E-03
 2.3415717650689E-04
 1.65358231276597E-03
 1.41181372953008E-03
 4.68050153672622E-02
 1.07933109899523E-03
 1.10414107236085E-03
 5.89715061666512E-03
 8.80683455558889E-02
 4.77606071891762E-02
 2.47082247754279E-02
 5.81666855202742E-03
 1.06018659163605E-02
 .124037799455708
 2.81279900492611E-02
 7.17442159679362E-03

 0
 0
 4.24333939460459E-04
 0
 5.36111335809664E-04
 2.05731135393343E-04
 0
 0
 0
 0
 0
 0
 0
 0
 0
 0
 0
 0
 0
 0
 0
 0
 0
 0
 6.08284812405502E-04
 0
 0
 0
 0
 6.55382132379175E-04
 0
 0

 0
 0
 4.24333939460459E-04
 0
 5.36111335809664E-04
 2.05731135393343E-04
 0
 0
 0
 0
 0
 0
 0
 0
 0
 0
 0
 0
 0
 0
 0
 0
 0
 0
 6.08284812405502E-04
 0
 0
 0
 0
 6.55382132379175E-04
 0
 0

 0
 0
 4.24333939460459E-04
 0
 5.36111335809664E-04
 2.05731135393343E-04
 0
 0
 0
 0
 0
 0
 0
 0
 0
 0
 0
 0
 0
 0
 0
 0
 0
 0
 6.08284812405502E-04
 0
 0
 0
 0
 6.55382132379175E-04
 0
 0

 0
 0
 0
 0
 0
 0
 0
 0
 2.8512028213115E-04
 1.75435932863625E-03
 4.37389945779277E-04
 1.01995216642824E-03
 9.03623910980779E-04
 0
 8.25030219782884E-04
 1.16429281622992E-03
 2.88676780687471E-03
 0
 1.26450412152594E-03
 7.97981673213227E-04
 0
 1.07933109899523E-03
 4.44952372443924E-04
 0
 0
 4.8449414632845E-03
 1.20209495067958E-02
 5.32373053914374E-03
 0
 0
 1.01239116380269E-02
 5.50230585682685E-03

 0
 0
 0
 0
 0
 0
 0
 0
 2.8512028213115E-04
 1.75435932863625E-03
 4.37389945779277E-04
 1.01995216642824E-03
 9.03623910980779E-04
 0
 8.25030219782884E-04
 1.16429281622992E-03
 2.88676780687471E-03
 0
 1.26450412152594E-03
 7.97981673213227E-04
 0
 1.07933109899523E-03
 4.44952372443924E-04
 0
 0
 4.8449414632845E-03
 1.20209495067958E-02
 5.32373053914374E-03
 0
 0
 1.01239116380269E-02
 5.50230585682685E-03

 0
 0
 0
 0
 0
 0
 0
 0
 2.8512028213115E-04
 1.75435932863625E-03
 4.37389945779277E-04
 1.01995216642824E-03
 9.03623910980779E-04
 0
 8.25030219782884E-04
 1.16429281622992E-03
 2.88676780687471E-03
 0
 1.26450412152594E-03
 7.97981673213227E-04
 0
 1.07933109899523E-03
 4.44952372443924E-04
 0
 0
 4.8449414632845E-03
 1.20209495067958E-02
 5.32373053914374E-03
 0
 0
 1.01239116380269E-02
 5.50230585682685E-03

 3.52489520816002E-03
 3.58849490700538E-03
 4.82508753740261E-03
 1.25362537471682E-02
 1.57374617931312E-02
 6.27148138537935E-03
 1.59843831242859E-02
 2.96139366616966E-03
 2.11540854484599E-03
 0
 0
 0
 0
 0
 0
 0
 0
 0
 0
 0
 1.21920782479248E-02
 0
 0
 4.13085429669779E-03
 1.76598816504823E-03
 1.73276307592638E-03
 1.33714677495504E-04
 0
 5.86237447605098E-03
 9.51361159906099E-04
 .001354693074622
 0

 3.52489520816002E-03
 3.58849490700538E-03
 4.82508753740261E-03
 1.25362537471682E-02
 1.57374617931312E-02
 6.27148138537935E-03
 1.59843831242859E-02
 2.96139366616966E-03
 2.11540854484599E-03
 0
 0
 0
 0
 0
 0
 0
 0
 0
 0
 0
 1.21920782479248E-02
 0
 0
 4.13085429669779E-03
 1.76598816504823E-03
 6.79514931735837E-04
 1.33714677495504E-04
 0
 5.86237447605098E-03
 9.51361159906099E-04
 6.04773694027678E-04
 0

 3.52489520816002E-03
 3.58849490700538E-03
 4.82508753740261E-03
 1.25362537471682E-02
 1.57374617931312E-02
 6.27148138537935E-03
 1.59843831242859E-02
 2.96139366616966E-03
 2.11540854484599E-03
 0
 0
 0
 0
 0
 0
 0
 0
 0
 0
 0
 1.21920782479248E-02
 0
 0
 4.13085429669779E-03
 1.76598816504823E-03
 6.79514931735837E-04
 1.33714677495504E-04
 0
 5.86237447605098E-03
 9.51361159906099E-04
 6.04773694027678E-04
 0

 0
 0
 0
 0
 0
 0
 0
 0
 0
 0
 0
 0
 0
 0
 0
 0
 0
 0
 0
 0
 0
 0
 0
 0
 0
 0
 0
 0
 0
 0
 3.7495969029716E-04
 0

 0
 0
 0
 0
 0
 0
 0
 0
 0
 0
 0
 0
 0
 0
 0
 0
 0
 0
 0
 0
 0
 0
 0
 0
 0
 0
 0
 0
 0
 0
 3.7495969029716E-04
 0

 0
 0
 0
 0
 0
 0
 0
 0
 0
 0
 0
 0
 0
 0
 0
 0
 0
 0
 0
 0
 0
 0
 0
 0
 0
 1.05324814419055E-03
 0
 0
 0
 0
 3.7495969029716E-04
 0

 0
 0
 0
 0
 0
 0
 0
 0
 0
 0
 0
 0
 0
 0
 0
 0
 0
 0
 0
 0
 0
 0
 0
 0
 0
 1.05324814419055E-03
 0
 0
 0
 0
 3.7495969029716E-04
 0

 1.22639451687009E-04
 5.68292935465425E-04
 2.35741077478033E-04
 0
 0
 2.2859015043667E-04
 3.43036259503554E-04
 2.72009492299758E-04
 2.11200208986037E-04
 0
 1.61996276214814E-04
 1.88880030820044E-04
 0
 0
 0
 3.44975649253878E-04
 0
 2.3415717650689E-04
 0
 0
 1.2172445271666E-03
 0
 6.59188699916924E-04
 0
 1.01380802067689E-03
 2.71503966058284E-02
 5.21982481776182E-03
 4.92938012883679E-04
 2.58879784554583E-03
 3.64101184655097E-04
 1.44428917744789E-02
 1.35859403872221E-03

 1.22639451687009E-04
 5.68292935465425E-04
 2.35741077478033E-04
 0
 0
 2.2859015043667E-04
 3.43036259503554E-04
 2.72009492299758E-04
 2.11200208986037E-04
 0
 1.61996276214814E-04
 1.88880030820044E-04
 0
 0
 0
 3.44975649253878E-04
 0
 2.3415717650689E-04
 0
 0

 1.22639451687009E-04
 5.68292935465425E-04
 2.35741077478033E-04
 0
 0
 2.2859015043667E-04
 3.43036259503554E-04
 2.72009492299758E-04
 2.11200208986037E-04
 0
 1.61996276214814E-04
 1.88880030820044E-04
 0
 0
 0
 3.44975649253878E-04
 0
 2.3415717650689E-04
 0
 0

 0
 0
 0
 0
 0
 0
 0
 0
 0
 0
 0
 0
 0
 0
 0
 0
 0
 0
 0
 0
 1.2172445271666E-03

 0
 0
 0
 0
 0
 0
 0
 0
 0
 0
 0
 0
 0
 0
 0
 0
 0
 0
 0
 0
 1.2172445271666E-03

 0
 0
 0
 0
 0
 0
 0
 0
 0
 0
 0
 0
 0
 0
 0
 0
 0
 0
 0
 0
 0
 0

 0
 0
 0
 0
 0
 0
 0
 0
 0
 0
 0
 0
 0
 0
 0
 0
 0
 0
 0
 0
 0
 0

 0
 0
 0
 0
 0
 0
 0
 0
 0
 0
 0
 0
 0
 0
 0
 0
 0
 0
 0
 0
 0
 0
 6.59188699916924E-04

 0
 0
 0
 0
 0
 0
 0
 0
 0
 0
 0
 0
 0
 0
 0
 0
 0
 0
 0
 0
 0
 0
 6.59188699916924E-04

 0
 0
 0
 0
 0
 0
 0
 0
 0
 0
 0
 0
 0
 0
 0
 0
 0
 0
 0
 0
 0
 0
 0
 0

 0
 0
 0
 0
 0
 0
 0
 0
 0
 0
 0
 0
 0
 0
 0
 0
 0
 0
 0
 0
 0
 0
 0
 0

 0
 0
 0
 0
 0
 0
 0
 0
 0
 0
 0
 0
 0
 0
 0
 0
 0
 0
 0
 0
 0
 0
 0
 0
 1.01380802067689E-03

 0
 0
 0
 0
 0
 0
 0
 0
 0
 0
 0
 0
 0
 0
 0
 0
 0
 0
 0
 0
 0
 0
 0
 0
 1.01380802067689E-03

 0
 0
 0
 0
 0
 0
 0
 0
 0
 0
 0
 0
 0
 0
 0
 0
 0
 0
 0
 0
 0
 0
 0
 0
 0
 2.71503966058284E-02

 0
 0
 0
 0
 0
 0
 0
 0
 0
 0
 0
 0
 0
 0
 0
 0
 0
 0
 0
 0
 0
 0
 0
 0
 0
 2.71503966058284E-02

 0
 0
 0
 0
 0
 0
 0
 0
 0
 0
 0
 0
 0
 0
 0
 0
 0
 0
 0
 0
 0
 0
 0
 0
 0
 0
 5.21982481776182E-03

 0
 0
 0
 0
 0
 0
 0
 0
 0
 0
 0
 0
 0
 0
 0
 0
 0
 0
 0
 0
 0
 0
 0
 0
 0
 0
 5.21982481776182E-03

 0
 0
 0
 0
 0
 0
 0
 0
 0
 0
 0
 0
 0
 0
 0
 0
 0
 0
 0
 0
 0
 0
 0
 0
 0
 0
 0
 4.92938012883679E-04

 0
 0
 0
 0
 0
 0
 0
 0
 0
 0
 0
 0
 0
 0
 0
 0
 0
 0
 0
 0
 0
 0
 0
 0
 0
 0
 0
 4.92938012883679E-04

 0
 0
 0
 0
 0
 0
 0
 0
 0
 0
 0
 0
 0
 0
 0
 0
 0
 0
 0
 0
 0
 0
 0
 0
 0
 0
 0
 0
 2.58879784554583E-03

 0
 0
 0
 0
 0
 0
 0
 0
 0
 0
 0
 0
 0
 0
 0
 0
 0
 0
 0
 0
 0
 0
 0
 0
 0
 0
 0
 0
 2.58879784554583E-03

 0
 0
 0
 0
 0
 0
 0
 0
 0
 0
 0
 0
 0
 0
 0
 0
 0
 0
 0
 0
 0
 0
 0
 0
 0
 0
 0
 0
 0
 3.64101184655097E-04

 0
 0
 0
 0
 0
 0
 0
 0
 0
 0
 0
 0
 0
 0
 0
 0
 0
 0
 0
 0
 0
 0
 0
 0
 0
 0
 0
 0
 0
 3.64101184655097E-04

 0
 0
 0
 0
 0
 0
 0
 0
 0
 0
 0
 0
 0
 0
 0
 0
 0
 0
 0
 0
 0
 0
 0
 0
 0
 0
 0
 0
 0
 0
 1.44428917744789E-02

 0
 0
 0
 0
 0
 0
 0
 0
 0
 0
 0
 0
 0
 0
 0
 0
 0
 0
 0
 0
 0
 0
 0
 0
 0
 0
 0
 0
 0
 0
 1.44428917744789E-02

 0
 0
 0
 0
 0
 0
 0
 0
 0
 0
 0
 0
 0
 0
 0
 0
 0
 0
 0
 0
 0
 0
 0
 0
 0
 0
 0
 0
 0
 0
 0
 1.35859403872221E-03

 0
 0
 0
 0
 0
 0
 0
 0
 0
 0
 0
 0
 0
 0
 0
 0
 0
 0
 0
 0
 0
 0
 0
 0
 0
 0
 0
 0
 0
 0
 0
 1.35859403872221E-03

 9.61031044832673E-02
 .107200812178023
 .10558570851327
 .130455576959713
 .144131470665887
 .160232903527605
 .07990238043
 8.47414187549713E-03
 9.65022493364218E-03
 1.34950717587181E-03
 3.36453804446153E-04
 3.92289294780091E-04
 2.08528594841336E-03
 0
 1.0154218089669E-03
 0
 5.9215749884577E-04
 0
 3.89078191240035E-04
 6.13832056316854E-04
 .032865602233566
 0
 0
 0
 8.42240509484541E-02
 1.36112067864604E-02
 7.33373577337473E-03
 0
 2.15069359476368E-03
 .121749834592133
 2.01901371698471E-03
 3.13521701244557E-04

 3.31126519554923E-04
 3.83597731439162E-04
 3.18250454595869E-04
 4.00643160992042E-04
 0
 0
 4.63098950330563E-04
 0
 0
 0
 0
 0
 0
 0
 0
 0
 0
 0
 0
 0

 3.31126519554923E-04
 0
 0
 0
 0
 0
 4.63098950330563E-04
 0
 0
 0
 0
 0
 0
 0
 0
 0
 0
 0
 0
 0

 0
 3.83597731439162E-04
 3.18250454595869E-04
 4.00643160992042E-04
 0
 0
 0
 0
 0
 0
 0
 0
 0
 0
 0
 0
 0
 0
 0
 0

 9.57719779637124E-02
 .106817214446584
 .105267458058674
 .130054933798721
 .144131470665887
 .160232903527605
 7.94392814796695E-02
 8.47414187549713E-03
 9.65022493364218E-03
 1.34950717587181E-03
 3.36453804446153E-04
 3.92289294780091E-04
 2.08528594841336E-03
 0
 1.0154218089669E-03
 0
 5.9215749884577E-04
 0
 3.89078191240035E-04
 6.13832056316854E-04
 .032865602233566
 0
 0
 0
 8.42240509484541E-02
 1.36112067864604E-02
 7.33373577337473E-03
 0
 2.15069359476368E-03
 .121749834592133
 2.01901371698471E-03
 3.13521701244557E-04

 9.57719779637124E-02
 .106817214446584
 .105267458058674
 .130054933798721
 .144131470665887
 .160232903527605
 7.94392814796695E-02
 8.47414187549713E-03
 9.65022493364218E-03
 1.34950717587181E-03
 3.36453804446153E-04
 3.92289294780091E-04
 2.08528594841336E-03
 0
 1.0154218089669E-03
 0
 5.9215749884577E-04
 0
 3.89078191240035E-04
 6.13832056316854E-04
 .032865602233566
 0
 0
 0
 8.42240509484541E-02
 1.36112067864604E-02
 7.33373577337473E-03
 0
 2.15069359476368E-03
 .121749834592133
 2.01901371698471E-03
 3.13521701244557E-04

 0
 0
 0
 0
 0
 0
 0
 0
 0
 0
 0
 0
 0
 0
 0
 0
 0
 0
 0
 0
 5.3009035860481E-04
 0
 0
 0
 4.56213609304126E-04
 4.21299257676551E-04
 0
 0
 0
 3.17120386635084E-04
 1.8747984514858E-04
 0

 0
 0
 0
 0
 0
 0
 0
 0
 0
 0
 0
 0
 0
 0
 0
 0
 0
 0
 0
 0
 5.3009035860481E-04
 0
 0
 0
 0
 0
 0
 0
 0
 3.17120386635084E-04
 0
 0

 0
 0
 0
 0
 0
 0
 0
 0
 0
 0
 0
 0
 0
 0
 0
 0
 0
 0
 0
 0
 5.3009035860481E-04
 0
 0
 0
 0
 0
 0
 0
 0
 3.17120386635084E-04
 0
 0

 0
 0
 0
 0
 0
 0
 0
 0
 0
 0
 0
 0
 0
 0
 0
 0
 0
 0
 0
 0
 0
 0
 0
 0
 4.56213609304126E-04
 4.21299257676551E-04
 0
 0
 0
 0
 1.8747984514858E-04
 0

 0
 0
 0
 0
 0
 0
 0
 0
 0
 0
 0
 0
 0
 0
 0
 0
 0
 0
 0
 0
 0
 0
 0
 0
 4.56213609304126E-04
 4.21299257676551E-04
 0
 0
 0
 0
 1.8747984514858E-04
 0

 0
 0
 0
 0
 0
 0
 0
 0
 0
 0
 0
 0
 0
 0
 0
 0
 0
 0
 0
 0
 0
 0
 0
 1.76629631996733E-03
 0
 0
 0
 0
 0
 0
 0
 0

 0
 0
 0
 0
 0
 0
 0
 0
 0
 0
 0
 0
 0
 0
 0
 0
 0
 0
 0
 0
 0
 0
 0
 1.76629631996733E-03
 0
 0
 0
 0
 0
 0
 0
 0

 0
 0
 0
 0
 0
 0
 0
 0
 0
 0
 0
 0
 0
 0
 0
 0
 0
 0
 0
 0
 0
 0
 0
 1.76629631996733E-03
 0
 0
 0
 0
 0
 0
 0
 0

 5.62915083243005E-04
 1.27865910479721E-04
 5.30417424324698E-04
 7.34512461817422E-04
 3.03796423625516E-03
 8.84643882189675E-04
 1.55909979944623E-03
 1.22404271535093E-04
 0
 0
 0
 0
 0
 0
 0
 1.25743624152972E-03
 0
 9.03177680812291E-04
 5.41930337798023E-04
 1.42496727359505E-04
 7.14826848579198E-03
 0
 0
 1.70741977596842E-03
 1.36559940385515E-02
 3.62949310488303E-02
 3.93789725222337E-03
 2.66186526957187E-04
 1.49114755903615E-03
 6.29166847085266E-03
 2.49348194047775E-02
 8.15156423235847E-05

 5.62915083243005E-04
 1.27865910479721E-04
 5.30417424324698E-04
 7.34512461817422E-04
 3.03796423625516E-03
 8.84643882189675E-04
 1.55909979944623E-03
 1.22404271535093E-04
 0
 0
 0
 0
 0
 0
 0
 1.25743624152972E-03
 0
 9.03177680812291E-04
 5.41930337798023E-04
 1.42496727359505E-04
 7.14826848579198E-03
 0
 0
 1.70741977596842E-03
 1.36559940385515E-02
 3.62949310488303E-02
 3.93789725222337E-03
 2.66186526957187E-04
 1.49114755903615E-03
 6.29166847085266E-03
 2.49348194047775E-02
 8.15156423235847E-05

 6.62253039109846E-05
 0
 0
 4.0064316099072E-04
 0
 3.70316043708017E-04
 9.26197900661126E-05
 0
 0
 0
 0
 0
 0
 0
 0
 1.25743624152972E-03
 0
 9.03177680812291E-04
 5.41930337798023E-04
 1.42496727359505E-04
 9.85968067007882E-04
 0
 0
 0
 9.85421396100901E-03
 .024603876648304
 2.07257750116878E-03
 2.66186526957187E-04
 5.59180334638557E-04
 6.29166847085266E-03
 2.02478232760539E-02
 8.15156423235847E-05

 6.62253039109846E-05
 0
 0
 4.0064316099072E-04
 0
 3.70316043708017E-04
 9.26197900661126E-05
 0
 0
 0
 0
 0
 0
 0
 0
 9.31434252987009E-05
 0
 0
 0
 0
 9.85968067007882E-04
 0
 0
 0
 9.85421396100901E-03
 .024603876648304
 2.07257750116878E-03
 2.66186526957187E-04
 5.59180334638557E-04
 6.29166847085266E-03
 2.02478232760539E-02
 8.15156423235847E-05

 0
 0
 0
 0
 0
 0
 0
 0
 0
 0
 0
 0
 0
 0
 0
 1.16429281623102E-03
 0
 9.03177680812291E-04
 5.41930337798023E-04
 1.42496727359505E-04

 4.9668977933202E-04
 1.27865910479721E-04
 5.30417424324698E-04
 3.33869300826702E-04
 3.03796423625516E-03
 5.14327838481659E-04
 1.46648000938012E-03
 1.22404271535093E-04
 0
 0
 0
 0
 0
 0
 0
 0
 0
 0
 0
 0
 6.16230041878409E-03
 0
 0
 1.70741977596842E-03
 3.80178007754246E-03
 1.16910544005264E-02
 1.86531975105459E-03
 0
 9.31967224397595E-04
 0
 4.68699612872356E-03
 0

 1.65563259777462E-04
 0
 0
 2.00321580496021E-04
 0
 0
 2.31549475165281E-04
 0
 0
 0
 0
 0
 0
 0
 0
 0
 0
 0
 0
 0
 4.10820027919462E-04
 0
 0
 0
 3.19349526513601E-03
 1.07431310707562E-02
 1.86531975105459E-03
 0
 0
 0
 4.68699612872356E-03
 0

 3.31126519554559E-04
 1.27865910479721E-04
 5.30417424324698E-04
 1.33547720330681E-04
 3.03796423625516E-03
 5.14327838481659E-04
 1.23493053421483E-03
 1.22404271535093E-04
 0
 0
 0
 0
 0
 0
 0
 0
 0
 0
 0
 0
 5.75148039086463E-03
 0
 0
 1.70741977596842E-03
 6.08284812406451E-04
 0
 0
 0
 9.31967224397595E-04
 0
 0
 0

 0
 0
 0
 0
 0
 0
 0
 0
 0
 0
 0
 0
 0
 0
 0
 0
 0
 0
 0
 0
 0
 0
 0
 0
 0
 9.47923329770161E-04
 0
 0
 0
 0
 0
 0

 3.98424196601283E-04
 6.3637488009949E-04
 3.53647868058742E-04
 3.31679993935818E-04
 2.75640208460784E-03
 3.09287849121302E-04
 2.503674129793E-04
 8.59136408939107E-03
 .023661779570754
 2.73249100460558E-02
 3.45385774397979E-02
 1.47643583568891E-02
 1.32699567383585E-02
 3.11401196581734E-02
 6.24734319376425E-02
 8.87181520631179E-02
 3.28842125157682E-02
 3.80937096656459E-02
 4.14927746034777E-02
 3.52102338999625E-02
 4.3686610844643E-03
 7.93972151921535E-02
 4.38892012116103E-03
 2.72761413558579E-03
 3.88536503471897E-03
 4.29618549590023E-02
 .131201366277753
 .030861559247833
 3.28959950857309E-03
 8.80606297352724E-03
 1.75680064896652E-02
 1.29629658724814E-02

 0
 0
 0
 0
 0
 0
 0
 0
 0
 0
 0
 0
 0
 0
 0
 7.6347069916968E-05
 0
 0
 0
 0
 1.34695091121135E-04
 1.76939524425789E-04
 0
 0
 1.4957823255873E-04
 1.10504723324997E-03
 1.35906721388243E-04
 0
 2.29172268294221E-04
 0
 1.16790723207134E-03
 2.47219570980248E-03

 0
 0
 0
 0
 0
 0
 0
 0
 0
 0
 0
 0
 0
 0
 0
 7.6347069916968E-05
 0
 0
 0
 0
 1.34695091121135E-04
 1.76939524425789E-04
 0
 0
 1.4957823255873E-04
 1.10504723324997E-03
 1.35906721388243E-04
 0
 2.29172268294221E-04
 0
 1.16790723207134E-03
 2.47219570980248E-03

 0
 0
 0
 0
 0
 0
 0
 0
 0
 0
 0
 0
 0
 0
 0
 7.6347069916968E-05
 0
 0
 0
 0
 1.34695091121135E-04
 1.76939524425789E-04
 0
 0
 1.4957823255873E-04
 1.10504723324997E-03
 1.35906721388243E-04
 0
 2.29172268294221E-04
 0
 1.16790723207134E-03
 2.47219570980248E-03

 5.42830359926104E-05
 0
 0
 0
 0
 0
 0
 0
 0
 0
 0
 0
 0
 0
 0
 0
 0
 0
 0
 0
 5.38780364483577E-04

 5.42830359926104E-05
 0
 0
 0
 0
 0
 0
 0
 0
 0
 0
 0
 0
 0
 0
 0
 0
 0
 0
 0
 5.38780364483577E-04

 5.42830359926104E-05
 0
 0
 0
 0
 0
 0
 0
 0
 0
 0
 0
 0
 0
 0
 0
 0
 0
 0
 0
 5.38780364483577E-04

 1.86550151861929E-04
 5.40278494984735E-05
 1.34472023068529E-04
 0
 1.76403802822308E-04
 0
 0
 0
 4.91586693330381E-05
 0
 0
 0
 5.19324086770563E-05
 1.15252862545762E-04
 0
 8.02960562919836E-05
 0
 0
 0
 0
 3.99048108711938E-04
 0
 0
 4.41574079992406E-04
 0
 2.17913409142757E-04
 0
 0
 0
 1.6949537906358E-04
 2.40814628682228E-04
 0

 1.86550151861929E-04
 5.40278494984735E-05
 1.34472023068529E-04
 0
 1.76403802822308E-04
 0
 0
 0
 4.91586693330381E-05
 0
 0
 0
 5.19324086770563E-05
 1.15252862545762E-04
 0
 8.02960562919836E-05
 0
 0
 0
 0
 3.99048108711938E-04
 0
 0
 4.41574079992406E-04
 0
 2.17913409142757E-04
 0
 0
 0
 1.6949537906358E-04
 2.40814628682228E-04
 0

 1.86550151861929E-04
 5.40278494984735E-05
 1.34472023068529E-04
 0
 3.77543194232573E-05
 0
 0
 0
 0
 0
 0
 0
 0
 0
 0
 0
 0
 0
 0
 0
 1.15723951526609E-04
 0
 0
 0
 0
 0
 0
 0
 0
 0
 0
 0

 0
 0
 0
 0
 1.38649483399051E-04
 0
 0
 0
 4.91586693330381E-05
 0
 0
 0
 5.19324086770563E-05
 1.15252862545762E-04
 0
 8.02960562919836E-05
 0
 0
 0
 0
 2.83324157185329E-04
 0
 0
 4.41574079992406E-04
 0
 2.17913409142757E-04
 0
 0
 0
 1.6949537906358E-04
 1.93944667395083E-04
 0

 0
 0
 0
 0
 0
 0
 0
 0
 0
 0
 0
 0
 0
 0
 0
 0
 0
 0
 0
 0
 0
 0
 0
 0
 0
 0
 0
 0
 0
 0
 4.6869961287145E-05
 0

 7.04524509691326E-05
 0
 1.35425725359721E-04
 0
 2.50945731654734E-03
 6.56588729978753E-05
 9.85316915596943E-05
 7.26612590600776E-03
 2.35375892482284E-02
 2.61287559583506E-02
 3.43397638280798E-02
 1.29098998724742E-02
 1.29441989021119E-02
 3.07210183398252E-02
 .062323426443136
 .086444612907393
 3.21843900171311E-02
 3.69442107991575E-02
 4.12398737791717E-02
 3.50107384816592E-02
 3.49634066313811E-04
 7.66200689292382E-02
 2.9900168290103E-03
 1.07848988508784E-03
 3.1079327442376E-03
 4.13787913543052E-03
 .127609691015804
 2.98191445147408E-02
 2.47794772503037E-03
 6.37612832543476E-03
 3.44211666048728E-03
 8.67187684293454E-05

 7.04524509691326E-05
 0
 1.35425725359721E-04
 0
 2.50945731654734E-03
 6.56588729978753E-05
 9.85316915596943E-05
 7.26612590600776E-03
 2.35375892482284E-02
 2.61287559583506E-02
 3.43397638280798E-02
 1.29098998724742E-02
 1.29441989021119E-02
 3.07210183398252E-02
 .062323426443136
 .086444612907393
 3.21843900171311E-02
 3.69442107991575E-02
 4.12398737791717E-02
 3.50107384816592E-02
 3.49634066313811E-04
 7.66200689292382E-02
 2.9900168290103E-03
 1.07848988508784E-03
 1.92515672011316E-03
 4.05986075437961E-03
 .127456166756458
 2.98191445147408E-02
 1.18354880225745E-03
 1.64281292491268E-03
 3.44211666048728E-03
 8.67187684293454E-05

 0
 0
 0
 0
 0
 0
 0
 0
 0
 0
 0
 1.06245017336275E-04
 6.27516604847763E-05
 .01336933205529
 4.60641872712383E-03
 .021442392698951
 3.84902374249856E-03
 8.56137176602231E-03
 4.00426305152246E-03
 2.32744654686917E-03
 0
 4.58715717073511E-02
 1.85396821851635E-03
 0
 7.60356015506877E-04
 2.98420307520544E-03
 .102851658495619
 2.25981270281324E-02
 0
 1.4336484145789E-03
 3.20278068795717E-03
 0

 7.04524509691326E-05
 0
 1.35425725359721E-04
 0
 2.50945731654734E-03
 6.56588729978753E-05
 9.85316915596943E-05
 7.26612590600776E-03
 2.35375892482284E-02
 2.61287559583506E-02
 3.43397638280798E-02
 1.28036548551379E-02
 1.28814472416271E-02
 1.73516862845351E-02
 5.77170077160122E-02
 .065002220208442
 2.83353662746326E-02
 2.83828390331352E-02
 3.72356107276492E-02
 .03268329193479
 3.49634066313811E-04
 2.96241939937649E-02
 1.13604861049395E-03
 5.44921205097012E-04
 1.16480070460628E-03
 1.07565767917417E-03
 2.45181508649563E-02
 7.22101748660838E-03
 8.92309044633547E-04
 2.09164510333779E-04
 2.39335972530102E-04
 8.67187684293454E-05

 0
 0
 0
 0
 0
 0
 0
 0
 0
 0
 0
 0
 0
 0
 0
 0
 0
 0
 0
 0
 0
 1.1243032281222E-03
 0
 5.33568679990825E-04
 0
 0
 8.63573958825131E-05
 0
 2.91239757623906E-04
 0
 0
 0

 0
 0
 0
 0
 0
 0
 0
 0
 0
 0
 0
 0
 0
 0
 0
 0
 0
 0
 0
 0
 0
 0
 0
 0
 1.18277602412445E-03
 7.80183810509051E-05
 1.53524259345978E-04
 0
 1.29439892277292E-03
 4.73331540052208E-03
 0
 0

 0
 0
 0
 0
 0
 0
 0
 0
 0
 0
 0
 0
 0
 0
 0
 0
 0
 0
 0
 0
 0
 0
 0
 0
 1.18277602412445E-03
 7.80183810509051E-05
 1.53524259345978E-04
 0
 1.29439892277292E-03
 4.73331540052208E-03
 0
 0

 8.71385577776114E-05
 3.93692408581964E-04
 8.3750119630492E-05
 2.00321580496021E-04
 7.05409652381913E-05
 2.43628976123427E-04
 0
 1.26503936131687E-03
 7.50316531925318E-05
 1.19615408770521E-03
 1.98813611718181E-04
 1.85445848441497E-03
 2.73825427569481E-04
 3.03848455802464E-04
 1.50005494506474E-04
 2.11689602951593E-03
 6.99822498637064E-04
 1.14949886648837E-03
 2.52900824306023E-04
 1.99495418303307E-04
 2.94650345383384E-03
 9.81210089995665E-04
 1.2135064702991E-03
 6.73981490514726E-04
 2.4011242594954E-04
 3.48126228711633E-03
 6.21773250352172E-04
 6.65466317392967E-04
 0
 1.73331327116202E-03
 1.49983876118864E-03
 7.41051293847335E-04

 8.71385577776114E-05
 3.93692408581964E-04
 8.3750119630492E-05
 2.00321580496021E-04
 7.05409652381913E-05
 2.43628976123427E-04
 0
 1.26503936131687E-03
 7.50316531925318E-05
 1.19615408770521E-03
 1.98813611718181E-04
 1.85445848441497E-03
 2.73825427569481E-04
 3.03848455802464E-04
 1.50005494506474E-04
 2.11689602951593E-03
 6.99822498637064E-04
 1.14949886648837E-03
 2.52900824306023E-04
 1.99495418303307E-04
 2.94650345383384E-03
 9.81210089995665E-04
 1.2135064702991E-03
 6.73981490514726E-04
 2.4011242594954E-04
 3.48126228711633E-03
 6.21773250352172E-04
 6.65466317392967E-04
 0
 1.73331327116202E-03
 1.49983876118864E-03
 7.41051293847335E-04

 0
 0
 0
 0
 0
 0
 0
 1.16840441010495E-03
 0
 1.19615408770521E-03
 1.98813611718181E-04
 1.85445848441497E-03
 2.73825427569481E-04
 3.03848455802464E-04
 1.50005494506474E-04
 2.11689602951593E-03
 6.99822498637064E-04
 1.14949886648837E-03
 0
 0
 3.73472752654056E-04
 9.81210089995665E-04
 1.2135064702991E-03
 0
 0
 0
 0
 0
 0
 0
 0
 7.41051293847335E-04

 0
 1.91798865719581E-04
 0
 2.00321580496021E-04
 0
 0
 0
 0
 0
 0
 0
 0
 0
 0
 0
 0
 0
 0
 2.52900824306023E-04
 1.99495418303307E-04
 4.10820027919462E-04
 0
 0
 0
 0
 3.37039406141241E-03
 6.21773250352172E-04
 6.65466317392967E-04
 0
 1.47460979785445E-03
 1.49983876118864E-03
 0

 8.71385577776114E-05
 2.01893542862383E-04
 8.3750119630492E-05
 0
 7.05409652381913E-05
 2.43628976123427E-04
 0
 9.66349512119157E-05
 7.50316531925318E-05
 0
 0
 0
 0
 0
 0
 0
 0
 0
 0
 0
 2.16221067326032E-03
 0
 0
 6.73981490514726E-04
 2.4011242594954E-04
 1.10868225703918E-04
 0
 0
 0
 2.58703473307569E-04
 0
 0

 0
 1.88654622019052E-04
 0
 0
 0
 0
 0
 0
 0
 0
 0
 0
 0
 0
 0
 0
 0
 0
 0
 0
 0
 0

 0
 1.88654622019052E-04
 0
 0
 0
 0
 0
 0
 0
 0
 0
 0
 0
 0
 0
 0
 0
 0
 0
 0
 0
 0

 0
 1.88654622019052E-04
 0
 0
 0
 0
 0
 0
 0
 0
 0
 0
 0
 0
 0
 0
 0
 0
 0
 0
 0
 0

 0
 0
 0
 0
 0
 0
 0
 0
 0
 0
 0
 0
 0
 0
 0
 0
 0
 0
 0
 0
 0
 0
 0

 0
 0
 0
 0
 0
 0
 0
 0
 0
 0
 0
 0
 0
 0
 0
 0
 0
 0
 0
 0
 0
 0
 0

 0
 0
 0
 0
 0
 0
 0
 0
 0
 0
 0
 0
 0
 0
 0
 0
 0
 0
 0
 0
 0
 0
 0

 0
 0
 0
 1.31358413439797E-04
 0
 0
 0
 0
 0
 0
 0
 0
 0
 0
 0
 0
 0
 0
 0
 0
 0
 0
 0
 0

 0
 0
 0
 1.31358413439797E-04
 0
 0
 0
 0
 0
 0
 0
 0
 0
 0
 0
 0
 0
 0
 0
 0
 0
 0
 0
 0

 0
 0
 0
 1.31358413439797E-04
 0
 0
 0
 0
 0
 0
 0
 0
 0
 0
 0
 0
 0
 0
 0
 0
 0
 0
 0
 0

 0
 0
 0
 0
 0
 0
 0
 0
 0
 0
 0
 0
 0
 0
 0
 0
 0
 0
 0
 0
 0
 0
 0
 0
 2.99156465117927E-04

 0
 0
 0
 0
 0
 0
 0
 0
 0
 0
 0
 0
 0
 0
 0
 0
 0
 0
 0
 0
 0
 0
 0
 0
 2.99156465117927E-04

 0
 0
 0
 0
 0
 0
 0
 0
 0
 0
 0
 0
 0
 0
 0
 0
 0
 0
 0
 0
 0
 0
 0
 0
 2.99156465117927E-04

 0
 0
 0
 0
 0
 0
 0
 0
 0
 0
 0
 0
 0
 0
 0
 0
 0
 0
 0
 0
 0
 0
 0
 0
 0
 4.8345816454659E-04

 0
 0
 0
 0
 0
 0
 0
 0
 0
 0
 0
 0
 0
 0
 0
 0
 0
 0
 0
 0
 0
 0
 0
 0
 0
 4.8345816454659E-04

 0
 0
 0
 0
 0
 0
 0
 0
 0
 0
 0
 0
 0
 0
 0
 0
 0
 0
 0
 0
 0
 0
 0
 0
 0
 4.8345816454659E-04

 0
 0
 0
 0
 0
 0
 1.51835721419606E-04
 0
 0
 0
 0
 0
 0
 0
 0
 0
 0
 0
 0
 0
 0
 0
 0
 0
 0
 0
 0

 0
 0
 0
 0
 0
 0
 1.51835721419606E-04
 0
 0
 0
 0
 0
 0
 0
 0
 0
 0
 0
 0
 0
 0
 0
 0
 0
 0
 0
 0

 0
 0
 0
 0
 0
 0
 1.51835721419606E-04
 0
 0
 0
 0
 0
 0
 0
 0
 0
 0
 0
 0
 0
 0
 0
 0
 0
 0
 0
 0

 0
 0
 0
 0
 0
 0
 0
 6.01988220664393E-05
 0
 0
 0
 0
 0
 0
 0
 0
 0
 0
 0
 0
 0
 0
 0
 0
 0
 0
 0
 1.0909283891688E-04

 0
 0
 0
 0
 0
 0
 0
 6.01988220664393E-05
 0
 0
 0
 0
 0
 0
 0
 0
 0
 0
 0
 0
 0
 0
 0
 0
 0
 0
 0
 1.0909283891688E-04

 0
 0
 0
 0
 0
 0
 0
 6.01988220664393E-05
 0
 0
 0
 0
 0
 0
 0
 0
 0
 0
 0
 0
 0
 0
 0
 0
 0
 0
 0
 1.0909283891688E-04

 0
 0
 0
 0
 0
 0
 0
 0
 0
 0
 0
 0
 0
 0
 0
 0
 0
 0
 0
 0
 0
 0
 0
 0
 0
 0
 0
 0
 0

 0
 0
 0
 0
 0
 0
 0
 0
 0
 0
 0
 0
 0
 0
 0
 0
 0
 0
 0
 0
 0
 0
 0
 0
 0
 0
 0
 0
 0

 0
 0
 0
 0
 0
 0
 0
 0
 0
 0
 0
 0
 0
 0
 0
 0
 0
 0
 0
 0
 0
 0
 0
 0
 0
 0
 0
 0
 0

 0
 0
 0
 0
 0
 0
 0
 0
 0
 0
 0
 0
 0
 0
 0
 0
 0
 0
 0
 0
 0
 0
 0
 0
 0
 0
 0
 0
 0
 3.22319081498384E-04

 0
 0
 0
 0
 0
 0
 0
 0
 0
 0
 0
 0
 0
 0
 0
 0
 0
 0
 0
 0
 0
 0
 0
 0
 0
 0
 0
 0
 0
 3.22319081498384E-04

 0
 0
 0
 0
 0
 0
 0
 0
 0
 0
 0
 0
 0
 0
 0
 0
 0
 0
 0
 0
 0
 0
 0
 0
 0
 0
 0
 0
 0
 3.22319081498384E-04

 0
 0
 0
 0
 0
 0
 0
 0
 0
 0
 0
 0
 0
 0
 0
 0
 0
 0
 0
 0
 0
 0
 0
 0
 0
 0
 0
 0
 0
 0
 5.53219215193125E-04

 0
 0
 0
 0
 0
 0
 0
 0
 0
 0
 0
 0
 0
 0
 0
 0
 0
 0
 0
 0
 0
 0
 0
 0
 0
 0
 0
 0
 0
 0
 5.53219215193125E-04

 0
 0
 0
 0
 0
 0
 0
 0
 0
 0
 0
 0
 0
 0
 0
 0
 0
 0
 0
 0
 0
 0
 0
 0
 0
 0
 0
 0
 0
 0
 5.53219215193125E-04

 0
 0
 0
 0
 0
 0
 0
 0
 0
 0
 0
 0
 0
 0
 0
 0
 0
 0
 0
 0
 0
 0
 0
 0
 0
 0
 0
 0
 0
 0
 0
 0

 0
 0
 0
 0
 0
 0
 0
 0
 0
 0
 0
 0
 0
 0
 0
 0
 0
 0
 0
 0
 0
 0
 0
 0
 0
 0
 0
 0
 0
 0
 0
 0

 0
 0
 0
 0
 0
 0
 0
 0
 0
 0
 0
 0
 0
 0
 0
 0
 0
 0
 0
 0
 0
 0
 0
 0
 0
 0
 0
 0
 0
 0
 0
 0

 0
 0
 0
 0
 0
 0
 0
 0
 0
 0
 0
 0
 0

 0
 0
 0
 0
 0
 0
 0
 0
 0
 0
 0
 0
 0

 0
 0
 0
 0
 0
 0
 0
 0
 0
 0
 0
 0
 0

 0
 0
 0
 0
 0
 0
 0
 0
 0
 0
 0
 0
 0
 0

 0
 0
 0
 0
 0
 0
 0
 0
 0
 0
 0
 0
 0
 0

 0
 0
 0
 0
 0
 0
 0
 0
 0
 0
 0
 0
 0
 0

 0
 0
 0
 0
 0
 0
 0
 0
 0
 0
 0
 0
 0
 0
 0

 0
 0
 0
 0
 0
 0
 0
 0
 0
 0
 0
 0
 0
 0
 0

 0
 0
 0
 0
 0
 0
 0
 0
 0
 0
 0
 0
 0
 0
 0

 0
 0
 0
 0
 0
 0
 0
 0
 0
 0
 0
 0
 0
 0
 0
 0

 0
 0
 0
 0
 0
 0
 0
 0
 0
 0
 0
 0
 0
 0
 0
 0

 0
 0
 0
 0
 0
 0
 0
 0
 0
 0
 0
 0
 0
 0
 0
 0

 0
 0
 0
 0
 0
 0
 0
 0
 0
 0
 0
 0
 0
 0
 0
 0
 0

 0
 0
 0
 0
 0
 0
 0
 0
 0
 0
 0
 0
 0
 0
 0
 0
 0

 0
 0
 0
 0
 0
 0
 0
 0
 0
 0
 0
 0
 0
 0
 0
 0
 0

 0
 0
 0
 0
 0
 0
 0
 0
 0
 0
 0
 0
 0
 0
 0
 0
 0
 0

 0
 0
 0
 0
 0
 0
 0
 0
 0
 0
 0
 0
 0
 0
 0
 0
 0
 0

 0
 0
 0
 0
 0
 0
 0
 0
 0
 0
 0
 0
 0
 0
 0
 0
 0
 0

 0
 0
 0
 0
 0
 0
 0
 0
 0
 0
 0
 0
 0
 0
 0
 0
 0
 0
 0

 0
 0
 0
 0
 0
 0
 0
 0
 0
 0
 0
 0
 0
 0
 0
 0
 0
 0
 0

 0
 0
 0
 0
 0
 0
 0
 0
 0
 0
 0
 0
 0
 0
 0
 0
 0
 0
 0

 0
 0
 0
 0
 0
 0
 0
 0
 0
 0
 0
 0
 0
 0
 0
 0
 0
 0
 0
 0

 0
 0
 0
 0
 0
 0
 0
 0
 0
 0
 0
 0
 0
 0
 0
 0
 0
 0
 0
 0

 0
 0
 0
 0
 0
 0
 0
 0
 0
 0
 0
 0
 0
 0
 0
 0
 0
 0
 0
 0

 0
 0
 0
 0
 0
 0
 0
 0
 0
 0
 0
 0
 0
 0
 0
 0
 0
 0
 0
 0
 0
 0
 1.85396821851635E-04
 5.33568679990825E-04
 0
 1.47357217210784E-02
 2.24529229293573E-03
 1.38638816123535E-04
 5.82479515248497E-04
 2.04806916368492E-04
 1.73303128463189E-03
 4.24560637100429E-04

 0
 0
 0
 0
 0
 0
 0
 0
 0
 0
 0
 0
 0
 0
 0
 0
 0
 0
 0
 0
 0
 0
 1.85396821851635E-04
 5.33568679990825E-04
 0
 1.39555379105652E-02
 2.24529229293573E-03
 1.38638816123535E-04
 5.82479515248497E-04
 2.04806916368492E-04
 1.64044864505234E-03
 4.24560637100429E-04

 0
 0
 0
 0
 0
 0
 0
 0
 0
 0
 0
 0
 0
 0
 0
 0
 0
 0
 0
 0
 0
 0
 1.85396821851635E-04
 5.33568679990825E-04
 0
 1.39555379105652E-02
 2.24529229293573E-03
 1.38638816123535E-04
 5.82479515248497E-04
 2.04806916368492E-04
 1.64044864505234E-03
 4.24560637100429E-04

 0
 0
 0
 0
 0
 0
 0
 0
 0
 0
 0
 0
 0
 0
 0
 0
 0
 0
 0
 0
 0
 0
 0
 0
 0
 7.80183810513159E-04
 0
 0
 0
 0
 9.25826395795457E-05
 0

 0
 0
 0
 0
 0
 0
 0
 0
 0
 0
 0
 0
 0
 0
 0
 0
 0
 0
 0
 0
 0
 0
 0
 0
 0
 7.80183810513159E-04
 0
 0
 0
 0
 9.25826395795457E-05
 0

 0
 0
 0
 0
 0
 0
 0
 0
 0
 0
 0
 0
 0
 0
 0
 0
 0
 0
 0
 0
 0
 0
 0
 0
 0
 9.15867951470041E-04
 1.80224130537419E-04
 0
 0
 0
 0
 0

 0
 0
 0
 0
 0
 0
 0
 0
 0
 0
 0
 0
 0
 0
 0
 0
 0
 0
 0
 0
 0
 0
 0
 0
 0
 9.15867951470041E-04
 1.80224130537419E-04
 0
 0
 0
 0
 0

 0
 0
 0
 0
 0
 0
 0
 0
 0
 0
 0
 0
 0
 0
 0
 0
 0
 0
 0
 0
 0
 0
 0
 0
 0
 9.15867951470041E-04
 1.80224130537419E-04
 0
 0
 0
 0
 0

 0
 0
 0
 0
 0
 0
 0
 0
 0
 0
 0
 0
 0
 0
 0
 0
 0
 0
 0
 0
 0
 1.61899664849389E-03
 0
 0
 0
 0
 0
 0
 0
 0
 0
 0

 0
 0
 0
 0
 0
 0
 0
 0
 0
 0
 0
 0
 0
 0
 0
 0
 0
 0
 0
 0
 0
 1.61899664849389E-03
 0
 0
 0
 0
 0
 0
 0
 0
 0
 0

 0
 0
 0
 0
 0
 0
 0
 0
 0
 0
 0
 0
 0
 0
 0
 0
 0
 0
 0
 0
 0
 1.61899664849389E-03
 0
 0
 0
 0
 0
 0
 0
 0
 0
 0

 0
 0
 0
 0
 0
 0
 0
 0
 0
 0
 0
 0
 0
 0
 0
 0
 0
 0
 0
 0
 0
 0
 0
 0
 0
 1.73626360739044E-03
 0
 0
 0
 0
 0
 0

 0
 0
 0
 0
 0
 0
 0
 0
 0
 0
 0
 0
 0
 0
 0
 0
 0
 0
 0
 0
 0
 0
 0
 0
 0
 8.42598515349776E-04
 0
 0
 0
 0
 0
 0

 0
 0
 0
 0
 0
 0
 0
 0
 0
 0
 0
 0
 0
 0
 0
 0
 0
 0
 0
 0
 0
 0
 0
 0
 0
 8.42598515349776E-04
 0
 0
 0
 0
 0
 0

 0
 0
 0
 0
 0
 0
 0
 0
 0
 0
 0
 0
 0
 0
 0
 0
 0
 0
 0
 0
 0

 0
 0
 0
 0
 0
 0
 0
 0
 0
 0
 0
 0
 0
 0
 0
 0
 0
 0
 0
 0
 0

 0
 0
 0
 0
 0
 0
 0
 0
 0
 0
 0
 0
 0
 0
 0
 0
 0
 0
 0
 0
 0
 0

 0
 0
 0
 0
 0
 0
 0
 0
 0
 0
 0
 0
 0
 0
 0
 0
 0
 0
 0
 0
 0
 0

 0
 0
 0
 0
 0
 0
 0
 0
 0
 0
 0
 0
 0
 0
 0
 0
 0
 0
 0
 0
 0
 0
 0

 0
 0
 0
 0
 0
 0
 0
 0
 0
 0
 0
 0
 0
 0
 0
 0
 0
 0
 0
 0
 0
 0
 0

 0
 0
 0
 0
 0
 0
 0
 0
 0
 0
 0
 0
 0
 0
 0
 0
 0
 0
 0
 0
 0
 0
 0
 0

 0
 0
 0
 0
 0
 0
 0
 0
 0
 0
 0
 0
 0
 0
 0
 0
 0
 0
 0
 0
 0
 0
 0
 0

 0
 0
 0
 0
 0
 0
 0
 0
 0
 0
 0
 0
 0
 0
 0
 0
 0
 0
 0
 0
 0
 0
 0
 0
 0

 0
 0
 0
 0
 0
 0
 0
 0
 0
 0
 0
 0
 0
 0
 0
 0
 0
 0
 0
 0
 0
 0
 0
 0
 0

 0
 0
 0
 0
 0
 0
 0
 0
 0
 0
 0
 0
 0
 0
 0
 0
 0
 0
 0
 0
 0
 0
 0
 0
 0
 8.93665092040666E-04

 0
 0
 0
 0
 0
 0
 0
 0
 0
 0
 0
 0
 0
 0
 0
 0
 0
 0
 0
 0
 0
 0
 0
 0
 0
 8.93665092040666E-04

 0
 0
 0
 0
 0
 0
 0
 0
 0
 0
 0
 0
 0
 0
 0
 0
 0
 0
 0
 0
 0
 0
 0
 0
 0
 0
 0

 0
 0
 0
 0
 0
 0
 0
 0
 0
 0
 0
 0
 0
 0
 0
 0
 0
 0
 0
 0
 0
 0
 0
 0
 0
 0
 0

 0
 0
 0
 0
 0
 0
 0
 0
 0
 0
 0
 0
 0
 0
 0
 0
 0
 0
 0
 0
 0
 0
 0
 0
 0
 0
 0
 0

 0
 0
 0
 0
 0
 0
 0
 0
 0
 0
 0
 0
 0
 0
 0
 0
 0
 0
 0
 0
 0
 0
 0
 0
 0
 0
 0
 0

 0
 0
 0
 0
 0
 0
 0
 0
 0
 0
 0
 0
 0
 0
 0
 0
 0
 0
 0
 0
 0
 0
 0
 0
 0
 0
 0
 0
 0

 0
 0
 0
 0
 0
 0
 0
 0
 0
 0
 0
 0
 0
 0
 0
 0
 0
 0
 0
 0
 0
 0
 0
 0
 0
 0
 0
 0
 0

 0
 0
 0
 0
 0
 0
 0
 0
 0
 0
 0
 0
 0
 0
 0
 0
 0
 0
 0
 0
 0
 0
 0
 0
 0
 0
 0
 0
 0
 0

 0
 0
 0
 0
 0
 0
 0
 0
 0
 0
 0
 0
 0
 0
 0
 0
 0
 0
 0
 0
 0
 0
 0
 0
 0
 0
 0
 0
 0
 0

 0
 0
 0
 0
 0
 0
 0
 0
 0
 0
 0
 0
 0
 0
 0
 0
 0
 0
 0
 0
 0
 0
 0
 0
 0
 0
 0
 0
 0
 0
 0

 0
 0
 0
 0
 0
 0
 0
 0
 0
 0
 0
 0
 0
 0
 0
 0
 0
 0
 0
 0
 0
 0
 0
 0
 0
 0
 0
 0
 0
 0
 0

 0
 0
 0
 0
 0
 0
 0
 0
 0
 0
 0
 0
 0
 0
 0
 0
 0
 0
 0
 0
 0
 0
 0
 0
 0
 0
 0
 0
 0
 0
 0
 0

 0
 0
 0
 0
 0
 0
 0
 0
 0
 0
 0
 0
 0
 0
 0
 0
 0
 0
 0
 0
 0
 0
 0
 0
 0
 0
 0
 0
 0
 0
 0
 0

 0
 0
 0
 0
 0
 0
 0
 0
 0
 0
 0
 0
 0
 0
 0
 0
 0
 0
 0
 0
 0
 0
 0
 0
 0
 1.22878950155619E-02
 0
 0
 0
 0
 8.43659303169516E-03
 9.17050976136549E-03

 0
 0
 0
 0
 0
 0
 0
 0
 0
 0
 0
 0
 0
 0
 0
 0
 0
 0
 0
 0
 0
 0
 0
 0
 0
 1.11644303284245E-02
 0
 0
 0
 0
 8.43659303169516E-03
 9.17050976136549E-03

 0
 0
 0
 0
 0
 0
 0
 0
 0
 0
 0
 0
 0
 0
 0
 0
 0
 0
 0
 0
 0
 0
 0
 0
 0
 1.11644303284245E-02
 0
 0
 0
 0
 8.43659303169516E-03
 9.17050976136549E-03

 0
 0
 0
 0
 0
 0
 0
 0
 0
 0
 0
 0
 0
 0
 0
 0
 0
 0
 0
 0
 0
 0
 0
 0
 0
 1.12346468713747E-03
 0
 0
 0
 0
 0
 0

 0
 0
 0
 0
 0
 0
 0
 0
 0
 0
 0
 0
 0
 0
 0
 0
 0
 0
 0
 0
 0
 0
 0
 0
 0
 1.12346468713747E-03
 0
 0
 0
 0
 0
 0

 0
 0
 0
 0
 0
 0
 0
 0
 0
 0
 0
 0
 0
 0
 0
 0
 0
 0
 0
 0
 0
 0
 0
 0
 8.85851668551702E-05
 3.8605464340153E-03
 4.08478866736092E-04
 1.29216760658829E-04
 0
 0
 4.94485675715511E-04
 6.79297019363206E-05

 0
 0
 0
 0
 0
 0
 0
 0
 0
 0
 0
 0
 0
 0
 0
 0
 0
 0
 0
 0
 0
 0
 0
 0
 8.85851668551702E-05
 3.8605464340153E-03
 4.08478866736092E-04
 1.29216760658829E-04
 0
 0
 4.94485675715511E-04
 6.79297019363206E-05

 0
 0
 0
 0
 0
 0
 0
 0
 0
 0
 0
 0
 0
 0
 0
 0
 0
 0
 0
 0
 0
 0
 0
 0
 8.85851668551702E-05
 9.81668173226916E-04
 2.01221116618701E-04
 1.29216760658829E-04
 0
 0
 1.82019267134544E-04
 0

 0
 0
 0
 0
 0
 0
 0
 0
 0
 0
 0
 0
 0
 0
 0
 0
 0
 0
 0
 0
 0
 0
 0
 0
 0
 2.87887826078838E-03
 2.07257750117391E-04
 0
 0
 0
 3.12466408580967E-04
 6.79297019363206E-05

 0
 0
 0
 0
 0
 0
 0
 0
 0
 0
 0
 0
 0
 0
 0
 0
 0
 0
 0
 0
 0

 0
 0
 0
 0
 0
 0
 0
 0
 0
 0
 0
 0
 0
 0
 0
 0
 0
 0
 0
 0
 0

 0
 0
 0
 0
 0
 0
 0
 0
 0
 0
 0
 0
 0
 0
 0
 0
 0
 0
 0
 0
 0

 0
 0
 0
 0
 0
 0
 0
 0
 0
 0
 0
 0
 0
 0
 0
 0
 0
 0
 0
 0
 0

 0
 0
 0
 0
 0
 0
 0
 0
 0
 0
 0
 0
 0
 0
 0
 0
 0
 0
 0
 0
 0
 0

 0
 0
 0
 0
 0
 0
 0
 0
 0
 0
 0
 0
 0
 0
 0
 0
 0
 0
 0
 0
 0
 0

 0
 0
 0
 0
 0
 0
 0
 0
 0
 0
 0
 0
 0
 0
 0
 0
 0
 0
 0
 0
 0
 0

 0
 0
 0
 0
 0
 0
 0
 0
 0
 0
 0
 0
 0
 0
 0
 0
 0
 0
 0
 0
 0
 0

 0
 0
 0
 0
 0
 0
 0
 0
 0
 0
 0
 0
 0
 0
 0
 0
 0
 0
 0
 0
 0
 0
 0

 0
 0
 0
 0
 0
 0
 0
 0
 0
 0
 0
 0
 0
 0
 0
 0
 0
 0
 0
 0
 0
 0
 0

 0
 0
 0
 0
 0
 0
 0
 0
 0
 0
 0
 0
 0
 0
 0
 0
 0
 0
 0
 0
 0
 0
 0

 0
 0
 0
 0
 0
 0
 0
 0
 0
 0
 0
 0
 0
 0
 0
 0
 0
 0
 0
 0
 0
 0
 0

 0
 0
 0
 0
 0
 0
 0
 0
 0
 0
 0
 0
 0
 0
 0
 0
 0
 0
 0
 0
 0
 0
 0
 0

 0
 0
 0
 0
 0
 0
 0
 0
 0
 0
 0
 0
 0
 0
 0
 0
 0
 0
 0
 0
 0
 0
 0
 0

 0
 0
 0
 0
 0
 0
 0
 0
 0
 0
 0
 0
 0
 0
 0
 0
 0
 0
 0
 0
 0
 0
 0
 0

 0
 0
 0
 0
 0
 0
 0
 0
 0
 0
 0
 0
 0
 0
 0
 0
 0
 0
 0
 0
 0
 0
 0
 0

 0
 0
 0
 0
 0
 0
 0
 0
 0
 0
 0
 0
 0
 0
 0
 0
 0
 0
 0
 0
 0
 0
 0
 0
 1.94133450767713E-04

 0
 0
 0
 0
 0
 0
 0
 0
 0
 0
 0
 0
 0
 0
 0
 0
 0
 0
 0
 0
 0
 0
 0
 0
 1.94133450767713E-04

 0
 0
 0
 0
 0
 0
 0
 0
 0
 0
 0
 0
 0
 0
 0
 0
 0
 0
 0
 0
 0
 0
 0
 0
 1.94133450767713E-04

 0
 0
 0
 0
 0
 0
 0
 0
 0
 0
 0
 0
 0
 0
 0
 0
 0
 0
 0
 0
 0
 0
 0
 0
 1.94133450767713E-04

 0
 0
 0
 0
 0
 0
 0
 0
 0
 0
 0
 0
 0
 0
 0
 0
 0
 0
 0
 0
 0
 0
 0
 0
 0
 0

 0
 0
 0
 0
 0
 0
 0
 0
 0
 0
 0
 0
 0
 0
 0
 0
 0
 0
 0
 0
 0
 0
 0
 0
 0
 0

 0
 0
 0
 0
 0
 0
 0
 0
 0
 0
 0
 0
 0
 0
 0
 0
 0
 0
 0
 0
 0
 0
 0
 0
 0
 0

 0
 0
 0
 0
 0
 0
 0
 0
 0
 0
 0
 0
 0
 0
 0
 0
 0
 0
 0
 0
 0
 0
 0
 0
 0
 0

 0
 0
 0
 0
 0
 0
 0
 0
 0
 0
 0
 0
 0
 0
 0
 0
 0
 0
 0
 0
 0
 0
 0
 0
 0
 0
 0

 0
 0
 0
 0
 0
 0
 0
 0
 0
 0
 0
 0
 0
 0
 0
 0
 0
 0
 0
 0
 0
 0
 0
 0
 0
 0
 0

 0
 0
 0
 0
 0
 0
 0
 0
 0
 0
 0
 0
 0
 0
 0
 0
 0
 0
 0
 0
 0
 0
 0
 0
 0
 0
 0

 0
 0
 0
 0
 0
 0
 0
 0
 0
 0
 0
 0
 0
 0
 0
 0
 0
 0
 0
 0
 0
 0
 0
 0
 0
 0
 0

 0
 0
 0
 0
 0
 0
 0
 0
 0
 0
 0
 0
 0
 0
 0
 0
 0
 0
 0
 0
 0
 0
 0
 0
 0
 0
 0
 0

 0
 0
 0
 0
 0
 0
 0
 0
 0
 0
 0
 0
 0
 0
 0
 0
 0
 0
 0
 0
 0
 0
 0
 0
 0
 0
 0
 0

 0
 0
 0
 0
 0
 0
 0
 0
 0
 0
 0
 0
 0
 0
 0
 0
 0
 0
 0
 0
 0
 0
 0
 0
 0
 0
 0
 0

 0
 0
 0
 0
 0
 0
 0
 0
 0
 0
 0
 0
 0
 0
 0
 0
 0
 0
 0
 0
 0
 0
 0
 0
 0
 0
 0
 0

 0
 0
 0
 0
 0
 0
 0
 0
 0
 0
 0
 0
 0
 0
 0
 0
 0
 0
 0
 0
 0
 0
 0
 0
 0
 0
 0
 0
 0

 0
 0
 0
 0
 0
 0
 0
 0
 0
 0
 0
 0
 0
 0
 0
 0
 0
 0
 0
 0
 0
 0
 0
 0
 0
 0
 0
 0
 0

 0
 0
 0
 0
 0
 0
 0
 0
 0
 0
 0
 0
 0
 0
 0
 0
 0
 0
 0
 0
 0
 0
 0
 0
 0
 0
 0
 0
 0

 0
 0
 0
 0
 0
 0
 0
 0
 0
 0
 0
 0
 0
 0
 0
 0
 0
 0
 0
 0
 0
 0
 0
 0
 0
 0
 0
 0
 0

 0
 0
 0
 0
 0
 0
 0
 0
 0
 0
 0
 0
 0
 0
 0
 0
 0
 0
 0
 0
 0
 0
 0
 0
 0
 0
 0
 0
 0
 0

 0
 0
 0
 0
 0
 0
 0
 0
 0
 0
 0
 0
 0
 0
 0
 0
 0
 0
 0
 0
 0
 0
 0
 0
 0
 0
 0
 0
 0
 0

 0
 0
 0
 0
 0
 0
 0
 0
 0
 0
 0
 0
 0
 0
 0
 0
 0
 0
 0
 0
 0
 0
 0
 0
 0
 0
 0
 0
 0
 0

 0
 0
 0
 0
 0
 0
 0
 0
 0
 0
 0
 0
 0
 0
 0
 0
 0
 0
 0
 0
 0
 0
 0
 0
 0
 0
 0
 0
 0
 0

 0
 0
 0
 0
 0
 0
 0
 0
 0
 0
 0
 0
 0
 0
 0
 0
 0
 0
 0
 0
 0
 0
 0
 0
 0
 0
 0
 0
 0
 0
 0

 0
 0
 0
 0
 0
 0
 0
 0
 0
 0
 0
 0
 0
 0
 0
 0
 0
 0
 0
 0
 0
 0
 0
 0
 0
 0
 0
 0
 0
 0
 0

 0
 0
 0
 0
 0
 0
 0
 0
 0
 0
 0
 0
 0
 0
 0
 0
 0
 0
 0
 0
 0
 0
 0
 0
 0
 0
 0
 0
 0
 0
 0

 0
 0
 0
 0
 0
 0
 0
 0
 0
 0
 0
 0
 0
 0
 0
 0
 0
 0
 0
 0
 0
 0
 0
 0
 0
 0
 0
 0
 0
 0
 0

 0
 0
 0
 0
 0
 0
 0
 0
 0
 0
 0
 0
 0
 0
 0
 0
 0
 0
 0
 0
 0
 0
 0
 0
 0
 0
 0
 0
 0
 0
 0
 0

 0
 0
 0
 0
 0
 0
 0
 0
 0
 0
 0
 0
 0
 0
 0
 0
 0
 0
 0
 0
 0
 0
 0
 0
 0
 0
 0
 0
 0
 0
 0
 0

 0
 0
 0
 0
 0
 0
 0
 0
 0
 0
 0
 0
 0
 0
 0
 0
 0
 0
 0
 0
 0
 0
 0
 0
 0
 0
 0
 0
 0
 0
 0
 0

 0
 0
 0
 0
 0
 0
 0
 0
 0
 0
 0
 0
 0
 0
 0
 0
 0
 0
 0
 0
 0
 0
 0
 0
 0
 0
 0
 0
 0
 0
 0
 0

 2.11357352907165E-04
 0
 0
 0
 0
 0
 0
 0
 0
 0
 0
 0
 0
 0
 0
 0
 0
 0
 0
 0
 3.32152362997964E-03

 2.11357352907165E-04
 0
 0
 0
 0
 0
 0
 0
 0
 0
 0
 0
 0
 0
 0
 0
 0
 0
 0
 0
 3.32152362997964E-03

 2.11357352907165E-04
 0
 0
 0
 0
 0
 0
 0
 0
 0
 0
 0
 0
 0
 0
 0
 0
 0
 0
 0
 3.32152362997964E-03

 2.11357352907165E-04
 0
 0
 0
 0
 0
 0
 0
 0
 0
 0
 0
 0
 0
 0
 0
 0
 0
 0
 0
 3.32152362997964E-03

 2.11357352907165E-04
 0
 0
 0
 0
 0
 0
 0
 0
 0
 0
 0
 0
 0
 0
 0
 0
 0
 0
 0
 3.32152362997964E-03

 3.83444509644069E-02
 .031173708974908
 6.62173112528901E-02
 4.70221523283633E-02
 .10347842300019
 3.55811998662426E-02
 3.65230705493388E-02
 .101626146441935
 6.78111071002164E-02
 .033990711992273
 5.70429387620423E-02
 6.79118150812808E-02
 6.61402501508817E-02
 3.02481137750719E-02
 .037566376007545
 .10668803172744
 .051352391764523
 2.61319408980837E-02
 3.38465603196025E-02
 .03713939704076
 4.84219872906501E-02
 4.02950276958997E-03
 2.74387296340051E-03
 1.22934223869859E-02
 .022384881096513
 4.55705363718958E-03
 1.10537466729104E-03
 1.43075258239488E-02
 1.67288116779066E-02
 1.17968783828356E-02
 1.20612033712253E-03
 4.07578211615404E-03

 3.83444509644069E-02
 .031173708974908
 6.62173112528901E-02
 4.70221523283633E-02
 .10347842300019
 3.55811998662426E-02
 3.65230705493388E-02
 .101626146441935
 6.78111071002164E-02
 .033990711992273
 5.70429387620423E-02
 6.79118150812808E-02
 6.61402501508817E-02
 3.02481137750719E-02
 .037566376007545
 .10668803172744
 .051352391764523
 2.61319408980837E-02
 3.38465603196025E-02
 .03713939704076
 4.84219872906501E-02
 4.02950276958997E-03
 2.74387296340051E-03
 1.22934223869859E-02
 .022384881096513
 4.55705363718958E-03
 1.10537466729104E-03
 1.43075258239488E-02
 1.67288116779066E-02
 1.17968783828356E-02
 1.20612033712253E-03
 4.07578211615404E-03

 3.83444509644069E-02
 .031173708974908
 6.62173112528901E-02
 4.70221523283633E-02
 .10347842300019
 3.55811998662426E-02
 3.65230705493388E-02
 .101626146441935
 6.78111071002164E-02
 .033990711992273
 5.70429387620423E-02
 6.79118150812808E-02
 6.61402501508817E-02
 3.02481137750719E-02
 .037566376007545
 .10668803172744
 .051352391764523
 2.61319408980837E-02
 3.38465603196025E-02
 .03713939704076
 4.84219872906501E-02
 4.02950276958997E-03
 2.74387296340051E-03
 1.22934223869859E-02
 .022384881096513
 4.55705363718958E-03
 1.10537466729104E-03
 1.43075258239488E-02
 1.67288116779066E-02
 1.17968783828356E-02
 1.20612033712253E-03
 4.07578211615404E-03

 9.9337955866477E-04
 3.83597731438529E-04
 9.54751363787608E-04
 6.00964741487402E-04
 5.36111335810254E-04
 1.54298351545007E-04
 4.63098950329799E-04
 0
 0
 0
 0
 0
 0
 0
 0
 0
 0
 0
 0
 0
 0
 0
 0
 0
 0
 0
 0
 0
 6.98975418297374E-04
 0
 0
 0

 9.9337955866477E-04
 3.83597731438529E-04
 9.54751363787608E-04
 6.00964741487402E-04
 5.36111335810254E-04
 1.54298351545007E-04
 4.63098950329799E-04
 0
 0
 0
 0
 0
 0
 0
 0
 0
 0
 0
 0
 0
 0
 0
 0
 0
 0
 0
 0
 0
 6.98975418297374E-04
 0
 0
 0

 2.20751013036616E-04
 0
 0
 0
 0
 0
 0
 0
 0
 0
 0
 0
 0
 0
 0
 0
 0
 0
 0
 0
 2.90312819728891E-02

 2.20751013036616E-04
 0
 0
 0
 0
 0
 0
 0
 0
 0
 0
 0
 0
 0
 0
 0
 0
 0
 0
 0
 2.90312819728891E-02

 3.71303203927055E-02
 3.07901112434694E-02
 6.52625598891025E-02
 .045886996705554
 9.82960134206965E-02
 3.54269015146976E-02
 3.54425063319026E-02
 .101626146441935
 6.76210269121287E-02
 .033990711992273
 5.70429387620423E-02
 6.79118150812808E-02
 6.61402501508817E-02
 3.02481137750719E-02
 .037566376007545
 .10668803172744
 .051352391764523
 2.61319408980837E-02
 3.38465603196025E-02
 .03713939704076
 .019390705317761
 4.02950276958997E-03
 2.74387296340051E-03
 1.22934223869859E-02
 2.11683114717001E-02
 3.99532129362085E-03
 1.10537466729104E-03
 1.43075258239488E-02
 1.60298362596093E-02
 1.17968783828356E-02
 1.20612033712253E-03
 4.07578211615404E-03

 3.09051418250533E-03
 1.79012274671187E-03
 1.10326824260041E-02
 2.93804984727057E-03
 7.14815114413672E-03
 4.3203538432568E-03
 5.24845477040795E-03
 6.46294553704808E-02
 5.36026130406631E-02
 2.63153899294954E-02
 4.66549275497703E-02
 5.13375923768711E-02
 5.22093815232676E-02
 2.49560865031835E-02
 3.56413054947165E-02
 9.40748595515906E-02
 4.79844959898348E-02
 1.98096971324064E-02
 3.06853000157807E-02
 3.16532730374273E-02
 0
 3.59777032999104E-03
 2.37307931969724E-03
 0
 0
 2.80866171783259E-03
 1.10537466729104E-03
 1.15347495014781E-02
 9.31967224396499E-04
 0
 7.4991938059432E-04
 4.07578211615404E-03

 0
 0
 0
 0
 0
 0
 0
 0
 0
 0
 0
 0
 0
 0
 0
 0
 0
 0
 0
 0
 0

 3.40398062102002E-02
 2.89999884967576E-02
 5.42298774630983E-02
 4.29489468582835E-02
 9.09244825533058E-02
 3.11065476714408E-02
 3.01940515614946E-02
 4.40655377525851E-03
 2.85120282131433E-03
 0
 0
 0
 0
 0
 0
 0
 0
 0
 0
 0
 .019390705317761
 4.31732439598925E-04
 0
 1.22934223869859E-02
 2.11683114717001E-02
 1.01111821842372E-03
 0
 0
 1.50978690352128E-02
 1.17968783828356E-02
 2.99967752237728E-04
 0

 0
 0
 0
 0
 0
 0
 0
 0
 0
 0
 0
 0
 0
 0
 0
 0
 0
 0
 0
 0
 0
 0

 0
 0
 0
 0
 0
 0
 0
 0
 0
 0
 0
 0
 0
 0
 0
 0
 0
 0
 0
 0
 0
 0
 3.7079364370327E-04

 0
 0
 0
 0
 0
 0
 0
 0
 0
 0
 0
 0
 0
 0
 0
 0
 0
 0
 0
 0
 0
 0
 0
 0

 0
 0
 0
 0
 2.23379723253904E-04
 0
 0
 0
 0
 0
 0
 0
 0
 0
 0
 0
 0
 0
 0
 0
 0
 0
 0
 0
 0

 0
 0
 0
 0
 0
 0
 0
 0
 0
 0
 0
 0
 0
 0
 0
 0
 0
 0
 0
 0
 0
 0
 0
 0
 0
 1.75541357364537E-04

 0
 0
 0
 0
 0
 0
 0
 0
 0
 0
 0
 0
 0
 0
 0
 0
 0
 0
 0
 0
 0
 0
 0
 0
 0
 0
 0

 0
 0
 0
 0
 0
 0
 0
 3.25901372961959E-02
 0
 0
 0
 0
 0
 0
 0
 0
 0
 0
 0
 0
 0
 0
 0
 0
 0
 0
 0
 2.7727763224707E-03

 0
 0
 0
 0
 0
 0
 0
 0
 1.11672110501512E-02
 0
 0
 0
 0
 0
 0
 0
 0
 0
 0
 0
 0
 0
 0
 0
 0
 0
 0
 0
 0

 0
 0
 0
 0
 0
 0
 0
 0
 0
 7.67532206277754E-03
 0
 0
 0
 0
 0
 0
 0
 0
 0
 0
 0
 0
 0
 0
 0
 0
 0
 0
 0
 0

 0
 0
 0
 0
 0
 0
 0
 0
 0
 0
 .010388011212272
 0
 0
 0
 0
 0
 0
 0
 0
 0
 0
 0
 0
 0
 0
 0
 0
 0
 0
 0
 1.56233204290483E-04

 0
 0
 0
 0
 0
 0
 0
 0
 0
 0
 0
 1.65742227044097E-02
 0
 0
 0
 0
 0
 0
 0
 0
 0
 0
 0
 0
 0
 0
 0
 0
 0
 0
 0
 0

 0
 0
 0
 0
 0
 0
 0
 0
 0
 0
 0
 0
 1.39308686276141E-02

 0
 0
 0
 0
 0
 0
 0
 0
 0
 0
 0
 0
 0
 5.29202727188832E-03

 0
 0
 0
 0
 0
 0
 0
 0
 0
 0
 0
 0
 0
 0
 1.92507051282855E-03

 0
 0
 0
 0
 0
 0
 0
 0
 0
 0
 0
 0
 0
 0
 0
 1.26131721758497E-02

 0
 0
 0
 0
 0
 0
 0
 0
 0
 0
 0
 0
 0
 0
 0
 0
 3.36789577468823E-03

 0
 0
 0
 0
 0
 0
 0
 0
 0
 0
 0
 0
 0
 0
 0
 0
 0
 6.32224376567734E-03

 0
 0
 0
 0
 0
 0
 0
 0
 0
 0
 0
 0
 0
 0
 0
 0
 0
 0
 3.1612603038218E-03

 0
 0
 0
 0
 0
 0
 0
 0
 0
 0
 0
 0
 0
 0
 0
 0
 0
 0
 0
 5.4861240033327E-03

 0
 0
 0
 0
 0
 0
 0
 0
 0
 0
 0
 0
 0
 0
 0
 0
 0
 0
 0
 0
 0
 0

 0
 0
 0
 0
 0
 0
 0
 0
 0
 0
 0
 0
 0
 0
 0
 0
 0
 0
 0
 0
 0
 0

 0
 0
 0
 0
 0
 0
 0
 0
 0
 0
 0
 0
 0
 0
 0
 0
 0
 0
 0
 0
 0
 0
 0

 0
 0
 0
 0
 0
 0
 0
 0
 0
 0
 0
 0
 0
 0
 0
 0
 0
 0
 0
 0
 0
 0
 0

 0
 0
 0
 5.34190881321841E-04
 0
 0
 0
 0
 0
 0
 0
 0
 0
 0
 0
 0
 0
 0
 0
 0
 0
 0
 0
 0

 0
 0
 0
 5.34190881321841E-04
 0
 0
 0
 0
 0
 0
 0
 0
 0
 0
 0
 0
 0
 0
 0
 0
 0
 0
 0
 0

 0
 0
 0
 0
 4.64629824368297E-03
 0
 0
 0
 0
 0
 0
 0
 0
 0
 0
 0
 0
 0
 0
 0
 0
 0
 0
 0
 0

 0
 0
 0
 0
 4.64629824368297E-03
 0
 0
 0
 0
 0
 0
 0
 0
 0
 0
 0
 0
 0
 0
 0
 0
 0
 0
 0
 0

 0
 0
 0
 0
 0
 0
 0
 0
 0
 0
 0
 0
 0
 0
 0
 0
 0
 0
 0
 0
 0
 0
 0
 0
 0
 5.61732343568735E-04

 0
 0
 0
 0
 0
 0
 0
 0
 0
 0
 0
 0
 0
 0
 0
 0
 0
 0
 0
 0
 0
 0
 0
 0
 0
 5.61732343568735E-04

 0
 0
 0
 0
 0
 0
 6.17465267106398E-04
 0
 0
 0
 0
 0
 0
 0
 0
 0
 0
 0
 0
 0
 0
 0
 0
 0
 0
 0
 0

 0
 0
 0
 0
 0
 0
 6.17465267106398E-04
 0
 0
 0
 0
 0
 0
 0
 0
 0
 0
 0
 0
 0
 0
 0
 0
 0
 0
 0
 0

 0
 0
 0
 0
 0
 0
 0
 0
 0
 0
 0
 0
 0
 0
 0
 0
 0
 0
 0
 0
 0
 0
 0
 0
 0
 0
 0
 0

 0
 0
 0
 0
 0
 0
 0
 0
 0
 0
 0
 0
 0
 0
 0
 0
 0
 0
 0
 0
 0
 0
 0
 0
 0
 0
 0
 0

 0
 0
 0
 0
 0
 0
 0
 0
 1.90080188087747E-04
 0
 0
 0
 0
 0
 0
 0
 0
 0
 0
 0
 0
 0
 0
 0
 0
 0
 0
 0
 0

 0
 0
 0
 0
 0
 0
 0
 0
 1.90080188087747E-04
 0
 0
 0
 0
 0
 0
 0
 0
 0
 0
 0
 0
 0
 0
 0
 0
 0
 0
 0
 0

 0
 0
 0
 0
 0
 0
 0
 0
 0
 0
 0
 0
 0
 0
 0
 0
 0
 0
 0
 0
 0
 0
 0
 0
 0
 0
 0
 0
 0
 0

 0
 0
 0
 0
 0
 0
 0
 0
 0
 0
 0
 0
 0
 0
 0
 0
 0
 0
 0
 0
 0
 0
 0
 0
 0
 0
 0
 0
 0
 0

 0
 0
 0
 0
 0
 0
 0
 0
 0
 0
 0
 0
 0
 0
 0
 0
 0
 0
 0
 0
 0
 0
 0
 0
 0
 0
 0
 0
 0
 0
 0

 0
 0
 0
 0
 0
 0
 0
 0
 0
 0
 0
 0
 0
 0
 0
 0
 0
 0
 0
 0
 0
 0
 0
 0
 0
 0
 0
 0
 0
 0
 0

 0
 0
 0
 0
 0
 0
 0
 0
 0
 0
 0
 0
 0
 0
 0
 0
 0
 0
 0
 0
 0
 0
 0
 0
 0
 0
 0
 0
 0
 0
 0
 0

 0
 0
 0
 0
 0
 0
 0
 0
 0
 0
 0
 0
 0
 0
 0
 0
 0
 0
 0
 0
 0
 0
 0
 0
 0
 0
 0
 0
 0
 0
 0
 0

 0
 0
 0
 0
 0
 0
 0
 0
 0
 0
 0
 0
 0

 0
 0
 0
 0
 0
 0
 0
 0
 0
 0
 0
 0
 0

 0
 0
 0
 0
 0
 0
 0
 0
 0
 0
 0
 0
 0
 0

 0
 0
 0
 0
 0
 0
 0
 0
 0
 0
 0
 0
 0
 0

 0
 0
 0
 0
 0
 0
 0
 0
 0
 0
 0
 0
 0
 0
 0

 0
 0
 0
 0
 0
 0
 0
 0
 0
 0
 0
 0
 0
 0
 0

 0
 0
 0
 0
 0
 0
 0
 0
 0
 0
 0
 0
 0
 0
 0
 0

 0
 0
 0
 0
 0
 0
 0
 0
 0
 0
 0
 0
 0
 0
 0
 0

 0
 0
 0
 0
 0
 0
 0
 0
 0
 0
 0
 0
 0
 0
 0
 0
 0

 0
 0
 0
 0
 0
 0
 0
 0
 0
 0
 0
 0
 0
 0
 0
 0
 0

 0
 0
 0
 0
 0
 0
 0
 0
 0
 0
 0
 0
 0
 0
 0
 0
 0
 0

 0
 0
 0
 0
 0
 0
 0
 0
 0
 0
 0
 0
 0
 0
 0
 0
 0
 0

 0
 0
 0
 0
 0
 0
 0
 0
 0
 0
 0
 0
 0
 0
 0
 0
 0
 0
 0

 0
 0
 0
 0
 0
 0
 0
 0
 0
 0
 0
 0
 0
 0
 0
 0
 0
 0
 0

 0
 0
 0
 0
 0
 0
 0
 0
 0
 0
 0
 0
 0
 0
 0
 0
 0
 0
 0
 0

 0
 0
 0
 0
 0
 0
 0
 0
 0
 0
 0
 0
 0
 0
 0
 0
 0
 0
 0
 0

 0
 0
 0
 0
 0
 0
 0
 0
 0
 0
 0
 0
 0
 0
 0
 0
 0
 0
 0
 0
 0
 0
 0
 0
 1.2165696248129E-03
 0
 0
 0
 0
 0
 0
 0

 0
 0
 0
 0
 0
 0
 0
 0
 0
 0
 0
 0
 0
 0
 0
 0
 0
 0
 0
 0
 0
 0
 0
 0
 1.2165696248129E-03
 0
 0
 0
 0
 0
 0
 0

 .344854473177812
 .28325495818987
 .239397274251795
 .226922617039291
 .173264482341964
 .169213858860691
 7.00340683427223E-02
 5.85780941964968E-02
 .185357883414884
 3.50506374200109E-02
 .10131955848175
 8.74396492677666E-02
 .112952988872512
 7.64976468536849E-02
 8.48062313419726E-02
 8.77876783441361E-02
 8.57851166610401E-02
 .089420235260469
 5.80723517811869E-02
 4.77874649925956E-02
 .22834746551775
 .101344692982785
 3.28523168321282E-02
 9.75897115702604E-02
 4.36634441906151E-02
 .189435455801195
 .135460211180774
 .115084081264182
 .133824668628777
 .223976843741001
 7.60152655476602E-02
 .160110307463016

 .344854473177812
 .28325495818987
 .239397274251795
 .226922617039291
 .173264482341964
 .169213858860691
 7.00340683427223E-02
 5.85780941964968E-02
 .185357883414884
 3.50506374200109E-02
 .10131955848175
 8.74396492677666E-02
 .112952988872512
 7.64976468536849E-02
 8.48062313419726E-02
 8.77876783441361E-02
 8.57851166610401E-02
 .089420235260469
 5.80723517811869E-02
 4.77874649925956E-02
 .22834746551775
 .101344692982785
 3.28523168321282E-02
 9.75897115702604E-02
 4.36634441906151E-02
 .189435455801195
 .135460211180774
 .115084081264182
 .133824668628777
 .223976843741001
 7.60152655476602E-02
 .160110307463016

 .344854473177812
 .28325495818987
 .239397274251795
 .226922617039291
 .173264482341964
 .169213858860691
 7.00340683427223E-02
 5.85780941964968E-02
 .185357883414884
 3.50506374200109E-02
 .10131955848175
 8.74396492677666E-02
 .112952988872512
 7.64976468536849E-02
 8.48062313419726E-02
 8.77876783441361E-02
 8.57851166610401E-02
 .089420235260469
 5.80723517811869E-02
 4.77874649925956E-02
 .22834746551775
 .101344692982785
 3.28523168321282E-02
 9.75897115702604E-02
 4.36634441906151E-02
 .189435455801195
 .135460211180774
 .115084081264182
 .133824668628777
 .223976843741001
 7.60152655476602E-02
 .160110307463016

 .344854473177812
 .28325495818987
 .239397274251795
 .226922617039291
 .173264482341964
 .169213858860691
 7.00340683427223E-02
 5.85780941964968E-02
 .185357883414884
 3.50506374200109E-02
 .10131955848175
 8.74396492677666E-02
 .112952988872512
 7.64976468536849E-02
 8.48062313419726E-02
 8.77876783441361E-02
 8.57851166610401E-02
 .089420235260469
 5.80723517811869E-02
 4.77874649925956E-02
 .22834746551775
 .101344692982785
 3.28523168321282E-02
 9.75897115702604E-02
 4.36634441906151E-02
 .189435455801195
 .135460211180774
 .115084081264182
 .133824668628777
 .223976843741001
 7.60152655476602E-02
 .160110307463016

 9.31293336246513E-04
 9.58994328597904E-04
 1.09398593767166E-03
 6.26004939047999E-04
 1.34027833952287E-03
 8.67928227439072E-04
 7.23592109889116E-04
 9.180320365132E-04
 7.12800705329052E-04
 0
 4.10053074168298E-04
 1.59367526004412E-04
 3.76509962908658E-04
 8.35583253456776E-04
 5.15643887364302E-04
 8.73219612175321E-04
 9.62255935625964E-04
 5.92710353032414E-04
 1.42256713671877E-03
 1.12216172795404E-03
 1.54057510469982E-03
 0
 0
 5.60247113990678E-03
 2.28106804652063E-03
 1.57987221628707E-03
 3.10886625176086E-03
 3.95120625952638E-03
 3.93173672792684E-03
 5.5297867419542E-03
 8.20224322525038E-04
 3.82104573390622E-04

 3.00083408346137E-03
 2.3974858214908E-03
 2.28742514240617E-03
 4.88283852457605E-03
 5.10981116942351E-03
 3.37527644005499E-03
 6.94648425494889E-03
 3.21311212779241E-03
 4.63320458464472E-03
 5.48237290197922E-04
 0
 3.18735052008298E-04
 1.88254981454018E-04
 0
 2.06257554946061E-04
 0
 0
 0
 0
 0
 3.08115020939046E-03
 0
 2.78095232777452E-04
 1.28056483197839E-02
 7.12833764538143E-03
 3.94968054071247E-03
 1.03628875058535E-03
 0
 1.26689294566235E-02
 1.59749394767948E-02
 5.85874516089313E-04
 1.27368191130601E-04

 1.10375506518308E-03
 1.40652501527482E-03
 2.01558621243876E-03
 6.67738601651199E-04
 1.16157456092074E-03
 1.90301300238757E-02
 3.08732633553199E-04
 8.8131075505348E-03
 .125928124608093
 2.57306034866456E-02
 9.72463646116785E-02
 7.53064682879542E-02
 .106928829465984
 7.39769707057604E-02
 8.19530018318827E-02
 8.30528875581236E-02
 8.05728970097389E-02
 8.64039981305903E-02
 .053783575302339
 4.44209798087335E-02
 1.6432801116798E-03
 8.49073797877053E-02
 3.17399359010201E-02
 6.48819514868221E-02
 .009732556998545
 4.64833514303489E-02
 .120900354235038
 .107583721311893
 .111370083315978
 1.86783907728493E-02
 3.08716811678732E-02
 .152298391740369

 .339404682543478
 .278491953024506
 .233701917158095
 .220620833986205
 .165569050875877
 .145554778290459
 6.20552593443311E-02
 4.52895804679643E-02
 5.27472521943263E-02
 4.38589832158579E-03
 .00174955978312
 2.88986447154386E-03
 1.50603985163297E-03
 2.22822200921807E-04
 1.10004029304748E-03
 1.24191233731601E-03
 6.41503957081858E-04
 8.42965835424805E-04
 3.3720109907414E-04
 0
 .221568935057082
 7.19554065996821E-04
 0
 3.41483955193684E-03
 2.28106804652775E-02
 .134394463199294
 9.11934100515236E-03
 2.21822105797656E-04
 2.79590167319607E-03
 .183179306000296
 4.17455121864679E-02
 2.71718807744023E-03

 4.13908149443654E-04
 0
 2.98359801183299E-04
 1.25200987810013E-04
 8.37673962203522E-05
 3.85745878862517E-04
 0
 3.44262013692071E-04
 1.3365013224905E-03
 4.38589832158156E-03
 1.91358101278298E-03
 8.76521393025581E-03
 3.95335461053159E-03
 1.46227069354591E-03
 1.03128777473201E-03
 2.61965883652116E-03
 3.60845975859339E-03
 1.58056094142151E-03
 2.52900824305501E-03
 2.24432345590808E-03
 5.13525034898409E-04
 1.51780935795847E-02
 8.3428569833063E-04
 9.60423623983276E-03
 1.71080103489047E-03
 3.02808841455281E-03
 1.2953609382377E-03
 3.32733158696484E-03
 3.05801745505307E-03
 6.14420749106295E-04
 1.9919733547048E-03
 4.58525488068511E-03

 0
 0
 0
 0
 0
 0
 0
 0
 0
 0
 0
 0
 0
 0
 0
 0
 0
 0
 0
 0
 0
 5.39665549498656E-04
 0
 1.28056483197798E-03
 0
 0
 0
 0
 0
 0
 0
 0

 1.11378920213806E-03
 3.48725210398662E-04
 1.21513809936535E-03
 4.77129582635236E-03
 .004077695311763
 2.62774768388538E-03
 2.38566125927356E-03
 1.98072366665878E-03
 .009020168925608
 9.14393347045261E-03
 6.70664583528675E-03
 8.14416351071299E-03
 .011327245187143
 2.41053108269306E-03
 8.36030622715224E-03
 5.37691591496594E-03
 1.08239213122433E-02
 6.83951825560106E-03
 8.06217173240914E-03
 7.66546031540153E-03
 2.01675286432994E-03
 9.81210089995665E-04
 0
 1.61428778818984E-02
 1.66725337218706E-02
 3.49806050313147E-03
 2.39665325590616E-02
 2.50457323091699E-02
 2.68576009212709E-02
 1.75761571865499E-03
 3.82913380697897E-03
 3.05066115966849E-03

 1.11378920213806E-03
 3.48725210398662E-04
 1.21513809936535E-03
 4.77129582635236E-03
 .004077695311763
 2.62774768388538E-03
 2.38566125927356E-03
 1.98072366665878E-03
 .009020168925608
 9.14393347045261E-03
 6.70664583528675E-03
 8.14416351071299E-03
 .011327245187143
 2.41053108269306E-03
 8.36030622715224E-03
 5.37691591496594E-03
 1.08239213122433E-02
 6.83951825560106E-03
 8.06217173240914E-03
 7.66546031540153E-03
 2.01675286432994E-03
 9.81210089995665E-04
 0
 1.61428778818984E-02
 1.66725337218706E-02
 3.49806050313147E-03
 2.39665325590616E-02
 2.50457323091699E-02
 2.68576009212709E-02
 1.75761571865499E-03
 3.82913380697897E-03
 3.05066115966849E-03

 4.51536163028944E-04
 3.48725210398662E-04
 5.78637190174308E-04
 2.36743686040452E-03
 1.21843485411421E-03
 9.81898600738638E-04
 8.41998091510115E-04
 1.00148949437804E-03
 3.8880038472451E-03
 7.97436058469704E-03
 4.37389945779343E-03
 4.40433890048174E-03
 6.70872297547014E-03
 1.51924227900731E-03
 3.30012087913748E-03
 4.44548166197996E-03
 5.94849123840927E-03
 4.31062074932665E-03
 3.67855744444365E-03
 5.80350307790839E-03
 3.73472752654056E-04
 9.81210089995665E-04
 0
 9.31319877802469E-03
 1.11979704102116E-02
 1.53199730064201E-03
 2.14794395576554E-02
 .022383867039598
 6.35432198452158E-03
 4.46851453894892E-04
 3.57916068011419E-03
 2.77894235192321E-03

 4.51536163028944E-04
 3.48725210398662E-04
 5.78637190174308E-04
 2.36743686040452E-03
 1.21843485411421E-03
 9.81898600738638E-04
 8.41998091510115E-04
 1.00148949437804E-03
 3.8880038472451E-03
 7.97436058469704E-03
 4.37389945779343E-03
 4.40433890048174E-03
 6.70872297547014E-03
 1.51924227900731E-03
 3.30012087913748E-03
 4.44548166197996E-03
 5.94849123840927E-03
 4.31062074932665E-03
 3.67855744444365E-03
 5.80350307790839E-03
 3.73472752654056E-04
 9.81210089995665E-04
 0
 9.31319877802469E-03
 1.11979704102116E-02
 1.53199730064201E-03
 2.14794395576554E-02
 .022383867039598
 6.35432198452158E-03
 4.46851453894892E-04
 3.57916068011419E-03
 2.77894235192321E-03

 4.51536163028944E-04
 3.48725210398662E-04
 5.78637190174308E-04
 2.36743686040452E-03
 1.21843485411421E-03
 9.81898600738638E-04
 8.41998091510115E-04
 1.00148949437804E-03
 3.8880038472451E-03
 7.97436058469704E-03
 4.37389945779343E-03
 4.40433890048174E-03
 6.70872297547014E-03
 1.51924227900731E-03
 3.30012087913748E-03
 4.44548166197996E-03
 5.94849123840927E-03
 4.31062074932665E-03
 3.67855744444365E-03
 5.80350307790839E-03
 3.73472752654056E-04
 9.81210089995665E-04
 0
 9.31319877802469E-03
 1.11979704102116E-02
 1.53199730064201E-03
 2.14794395576554E-02
 .022383867039598
 6.35432198452158E-03
 4.46851453894892E-04
 3.57916068011419E-03
 2.77894235192321E-03

 6.62253039109118E-04
 0
 6.36500909191038E-04
 2.40385896594785E-03
 2.85926045764879E-03
 1.64584908314674E-03
 1.54366316776345E-03
 9.79234172280746E-04
 5.1321650783629E-03
 1.16957288575557E-03
 2.33274637749333E-03
 3.73982461023125E-03
 4.61852221167291E-03
 8.91288803685757E-04
 5.06018534801476E-03
 9.31434252985984E-04
 4.87543007383398E-03
 2.52889750627442E-03
 4.38361428796549E-03
 1.86195723749314E-03
 1.64328011167589E-03
 0
 0
 6.82967910387367E-03
 5.47456331165901E-03
 1.96606320248946E-03
 2.48709300140612E-03
 2.66186526957187E-03
 2.05032789367493E-02
 1.3107642647601E-03
 2.49973126864773E-04
 2.71718807745282E-04

 0
 0
 2.1216696973058E-04
 0
 0
 0
 0
 0
 1.90080188087747E-04
 0
 0
 0
 2.00805313551284E-04
 0
 0
 0
 0
 0
 0
 0
 0
 0
 0
 0
 0
 0
 0
 8.87288423190623E-04
 0
 0
 0
 0

 0
 0
 2.1216696973058E-04
 0
 0
 0
 0
 0
 1.90080188087747E-04
 0
 0
 0
 2.00805313551284E-04
 0
 0
 0
 0
 0
 0
 0
 0
 0
 0
 0
 0
 0
 0
 8.87288423190623E-04
 0
 0
 0
 0

 6.62253039109118E-04
 0
 0
 0
 0
 0
 0
 0
 0
 0
 0
 0
 0
 0
 0
 0
 0
 0
 0
 0
 1.64328011167589E-03

 6.62253039109118E-04
 0
 0
 0
 0
 0
 0
 0
 0
 0
 0
 0
 0
 0
 0
 0
 0
 0
 0
 0
 1.64328011167589E-03

 0
 0
 0
 0
 0
 0
 0
 0
 0
 0
 0
 0
 0
 0
 0
 0
 0
 0
 0
 0
 0
 0

 0
 0
 0
 0
 0
 0
 0
 0
 0
 0
 0
 0
 0
 0
 0
 0
 0
 0
 0
 0
 0
 0

 0
 0
 4.24333939460459E-04
 0
 0
 0
 0
 0
 0
 0
 0
 0
 0
 0
 0
 0
 0
 0
 0
 0
 0
 0
 0

 0
 0
 4.24333939460459E-04
 0
 0
 0
 0
 0
 0
 0
 0
 0
 0
 0
 0
 0
 0
 0
 0
 0
 0
 0
 0

 0
 0
 0
 2.40385896594785E-03
 0
 0
 0
 0
 0
 0
 0
 0
 0
 0
 0
 0
 0
 0
 0
 0
 0
 0
 0
 6.82967910387367E-03

 0
 0
 0
 2.40385896594785E-03
 0
 0
 0
 0
 0
 0
 0
 0
 0
 0
 0
 0
 0
 0
 0
 0
 0
 0
 0
 6.82967910387367E-03

 0
 0
 0
 0
 2.85926045764879E-03
 0
 0
 0
 0
 0
 0
 0
 0
 0
 0
 0
 0
 0
 0
 0
 0
 0
 0
 0
 5.47456331165901E-03

 0
 0
 0
 0
 2.85926045764879E-03
 0
 0
 0
 0
 0
 0
 0
 0
 0
 0
 0
 0
 0
 0
 0
 0
 0
 0
 0
 5.47456331165901E-03

 0
 0
 0
 0
 0
 1.64584908314674E-03
 0
 0
 0
 0
 0
 0
 0
 0
 0
 0
 0
 0
 0
 0
 0
 0
 0
 0
 0
 1.96606320248946E-03

 0
 0
 0
 0
 0
 1.64584908314674E-03
 0
 0
 0
 0
 0
 0
 0
 0
 0
 0
 0
 0
 0
 0
 0
 0
 0
 0
 0
 1.96606320248946E-03

 0
 0
 0
 0
 0
 0
 1.54366316776345E-03
 0
 0
 0
 0
 0
 0
 0
 0
 0
 0
 0
 0
 0
 0
 0
 0
 0
 0
 0
 2.48709300140612E-03

 0
 0
 0
 0
 0
 0
 1.54366316776345E-03
 0
 0
 0
 0
 0
 0
 0
 0
 0
 0
 0
 0
 0
 0
 0
 0
 0
 0
 0
 2.48709300140612E-03

 0
 0
 0
 0
 0
 0
 0
 9.79234172280746E-04
 0
 0
 0
 0
 0
 0
 0
 0
 0
 0
 0
 0
 0
 0
 0
 0
 0
 0
 0
 1.77457684638125E-03

 0
 0
 0
 0
 0
 0
 0
 9.79234172280746E-04
 0
 0
 0
 0
 0
 0
 0
 0
 0
 0
 0
 0
 0
 0
 0
 0
 0
 0
 0
 1.77457684638125E-03

 0
 0
 0
 0
 0
 0
 0
 0
 4.94208489027515E-03
 0
 0
 0
 0
 0
 0
 0
 0
 0
 0
 0
 0
 0
 0
 0
 0
 0
 0
 0
 2.05032789367493E-02

 0
 0
 0
 0
 0
 0
 0
 0
 4.94208489027515E-03
 0
 0
 0
 0
 0
 0
 0
 0
 0
 0
 0
 0
 0
 0
 0
 0
 0
 0
 0
 2.05032789367493E-02

 0
 0
 0
 0
 0
 0
 0
 0
 0
 1.16957288575557E-03
 0
 0
 0
 0
 0
 0
 0
 0
 0
 0
 0
 0
 0
 0
 0
 0
 0
 0
 0
 1.3107642647601E-03

 0
 0
 0
 0
 0
 0
 0
 0
 0
 1.16957288575557E-03
 0
 0
 0
 0
 0
 0
 0
 0
 0
 0
 0
 0
 0
 0
 0
 0
 0
 0
 0
 1.3107642647601E-03

 0
 0
 0
 0
 0
 0
 0
 0
 0
 0
 2.33274637749333E-03
 0
 0
 0
 0
 0
 0
 0
 0
 0
 0
 0
 0
 0
 0
 0
 0
 0
 0
 0
 2.49973126864773E-04

 0
 0
 0
 0
 0
 0
 0
 0
 0
 0
 2.33274637749333E-03
 0
 0
 0
 0
 0
 0
 0
 0
 0
 0
 0
 0
 0
 0
 0
 0
 0
 0
 0
 2.49973126864773E-04

 0
 0
 0
 0
 0
 0
 0
 0
 0
 0
 0
 3.73982461023125E-03
 0
 0
 0
 0
 0
 0
 0
 0
 0
 0
 0
 0
 0
 0
 0
 0
 0
 0
 0
 2.71718807745282E-04

 0
 0
 0
 0
 0
 0
 0
 0
 0
 0
 0
 3.73982461023125E-03
 0
 0
 0
 0
 0
 0
 0
 0
 0
 0
 0
 0
 0
 0
 0
 0
 0
 0
 0
 2.71718807745282E-04

 0
 0
 0
 0
 0
 0
 0
 0
 0
 0
 0
 0
 4.41771689812162E-03

 0
 0
 0
 0
 0
 0
 0
 0
 0
 0
 0
 0
 4.41771689812162E-03

 0
 0
 0
 0
 0
 0
 0
 0
 0
 0
 0
 0
 0
 8.91288803685757E-04

 0
 0
 0
 0
 0
 0
 0
 0
 0
 0
 0
 0
 0
 8.91288803685757E-04

 0
 0
 0
 0
 0
 0
 0
 0
 0
 0
 0
 0
 0
 0
 5.06018534801476E-03

 0
 0
 0
 0
 0
 0
 0
 0
 0
 0
 0
 0
 0
 0
 5.06018534801476E-03

 0
 0
 0
 0
 0
 0
 0
 0
 0
 0
 0
 0
 0
 0
 0
 9.31434252985984E-04

 0
 0
 0
 0
 0
 0
 0
 0
 0
 0
 0
 0
 0
 0
 0
 9.31434252985984E-04

 0
 0
 0
 0
 0
 0
 0
 0
 0
 0
 0
 0
 0
 0
 0
 0
 4.87543007383398E-03

 0
 0
 0
 0
 0
 0
 0
 0
 0
 0
 0
 0
 0
 0
 0
 0
 4.87543007383398E-03

 0
 0
 0
 0
 0
 0
 0
 0
 0
 0
 0
 0
 0
 0
 0
 0
 0
 2.52889750627442E-03

 0
 0
 0
 0
 0
 0
 0
 0
 0
 0
 0
 0
 0
 0
 0
 0
 0
 2.52889750627442E-03

 0
 0
 0
 0
 0
 0
 0
 0
 0
 0
 0
 0
 0
 0
 0
 0
 0
 0
 4.38361428796549E-03

 0
 0
 0
 0
 0
 0
 0
 0
 0
 0
 0
 0
 0
 0
 0
 0
 0
 0
 4.38361428796549E-03

 0
 0
 0
 0
 0
 0
 0
 0
 0
 0
 0
 0
 0
 0
 0
 0
 0
 0
 0
 1.86195723749314E-03

 0
 0
 0
 0
 0
 0
 0
 0
 0
 0
 0
 0
 0
 0
 0
 0
 0
 0
 0
 1.86195723749314E-03

 3.29470886956957E-02
 4.29629459211481E-02
 3.21432959141671E-02
 4.70755714165154E-02
 5.10646047358537E-02
 3.05510736058757E-02
 6.99279414998156E-02
 2.23999816909039E-02
 1.02643301567478E-02
 0
 4.37389945779277E-04
 6.79968110951316E-04
 0
 3.34233301382711E-04
 0
 3.10478084328491E-04
 3.84902374249962E-04
 3.16112188284302E-04
 0
 1.99495418303307E-04
 2.99898620381148E-02
 1.83486286829404E-02
 5.30976497782475E-02
 1.28056483197798E-02
 2.41793212932113E-02
 8.42598515353103E-04
 2.07257750118031E-04
 0
 1.74743854574344E-02
 1.08138051842616E-02
 1.8747984514858E-04
 .00373613360648

 3.29470886956957E-02
 4.29629459211481E-02
 3.21432959141671E-02
 4.70755714165154E-02
 5.10646047358537E-02
 3.05510736058757E-02
 6.99279414998156E-02
 2.23999816909039E-02
 1.02643301567478E-02
 0
 4.37389945779277E-04
 6.79968110951316E-04
 0
 3.34233301382711E-04
 0
 3.10478084328491E-04
 3.84902374249962E-04
 3.16112188284302E-04
 0
 1.99495418303307E-04
 2.99898620381148E-02
 1.83486286829404E-02
 5.30976497782475E-02
 1.28056483197798E-02
 2.41793212932113E-02
 8.42598515353103E-04
 2.07257750118031E-04
 0
 1.74743854574344E-02
 1.08138051842616E-02
 1.8747984514858E-04
 .00373613360648

 3.29470886956957E-02
 4.29629459211481E-02
 3.21432959141671E-02
 4.70755714165154E-02
 5.10646047358537E-02
 3.05510736058757E-02
 6.99279414998156E-02
 2.23999816909039E-02
 1.02643301567478E-02
 0
 4.37389945779277E-04
 6.79968110951316E-04
 0
 3.34233301382711E-04
 0
 3.10478084328491E-04
 3.84902374249962E-04
 3.16112188284302E-04
 0
 1.99495418303307E-04
 2.99898620381148E-02
 1.83486286829404E-02
 5.30976497782475E-02
 1.28056483197798E-02
 2.41793212932113E-02
 8.42598515353103E-04
 2.07257750118031E-04
 0
 1.74743854574344E-02
 1.08138051842616E-02
 1.8747984514858E-04
 .00373613360648

 3.29470886956957E-02
 4.29629459211481E-02
 3.21432959141671E-02
 4.70755714165154E-02
 5.10646047358537E-02
 3.05510736058757E-02
 6.99279414998156E-02
 2.23999816909039E-02
 1.02643301567478E-02
 0
 4.37389945779277E-04
 6.79968110951316E-04
 0
 3.34233301382711E-04
 0
 3.10478084328491E-04
 3.84902374249962E-04
 3.16112188284302E-04
 0
 1.99495418303307E-04
 2.99898620381148E-02
 1.83486286829404E-02
 5.30976497782475E-02
 1.28056483197798E-02
 2.41793212932113E-02
 8.42598515353103E-04
 2.07257750118031E-04
 0
 1.74743854574344E-02
 1.08138051842616E-02
 1.8747984514858E-04
 .00373613360648

 0
 0
 0
 0
 0
 0
 0
 0
 0
 0
 0
 1.69992027738039E-04
 0
 0
 0
 3.10478084328491E-04
 3.84902374249962E-04
 0
 0
 0
 0
 1.83486286829404E-02
 5.30976497782475E-02
 0
 0
 0
 0
 0
 0
 0
 0
 3.53234450067104E-03

 3.29470886956957E-02
 4.29629459211481E-02
 3.21432959141671E-02
 4.70755714165154E-02
 5.10646047358537E-02
 3.05510736058757E-02
 6.99279414998156E-02
 2.23999816909039E-02
 1.02643301567478E-02
 0
 4.37389945779277E-04
 5.09976083213276E-04
 0
 3.34233301382711E-04
 0
 0
 0
 3.16112188284302E-04
 0
 1.99495418303307E-04
 2.99898620381148E-02
 0
 0
 1.28056483197798E-02
 2.41793212932113E-02
 8.42598515353103E-04
 2.07257750118031E-04
 0
 1.74743854574344E-02
 1.08138051842616E-02
 1.8747984514858E-04
 2.03789105808962E-04

 6.20862224164798E-04
 2.39748582149476E-04
 1.19343920473451E-03
 5.00803951239226E-04
 1.67534792440704E-04
 0
 5.78873687912248E-04
 2.295080091283E-04
 0
 0
 0
 0
 0
 0
 0
 0
 0
 0
 0
 0
 1.02705006979682E-03
 0
 0
 1.60070603997247E-03
 0
 0
 0
 0
 1.74743854574549E-03
 6.14420749105476E-04
 2.34349806435725E-04
 0

 6.20862224164798E-04
 2.39748582149476E-04
 1.19343920473451E-03
 5.00803951239226E-04
 1.67534792440704E-04
 0
 5.78873687912248E-04
 2.295080091283E-04
 0
 0
 0
 0
 0
 0
 0
 0
 0
 0
 0
 0
 1.02705006979682E-03
 0
 0
 1.60070603997247E-03
 0
 0
 0
 0
 1.74743854574549E-03
 6.14420749105476E-04
 2.34349806435725E-04
 0

 6.20862224164798E-04
 2.39748582149476E-04
 1.19343920473451E-03
 5.00803951239226E-04
 1.67534792440704E-04
 0
 5.78873687912248E-04
 2.295080091283E-04
 0
 0
 0
 0
 0
 0
 0
 0
 0
 0
 0
 0
 1.02705006979682E-03
 0
 0
 1.60070603997247E-03
 0
 0
 0
 0
 1.74743854574549E-03
 6.14420749105476E-04
 2.34349806435725E-04
 0

 6.20862224164798E-04
 2.39748582149476E-04
 1.19343920473451E-03
 5.00803951239226E-04
 1.67534792440704E-04
 0
 5.78873687912248E-04
 2.295080091283E-04
 0
 0
 0
 0
 0
 0
 0
 0
 0
 0
 0
 0
 1.02705006979682E-03
 0
 0
 1.60070603997247E-03
 0
 0
 0
 0
 1.74743854574549E-03
 6.14420749105476E-04
 2.34349806435725E-04
 0

 6.20862224164798E-04
 2.39748582149476E-04
 1.19343920473451E-03
 5.00803951239226E-04
 1.67534792440704E-04
 0
 5.78873687912248E-04
 2.295080091283E-04
 0
 0
 0
 0
 0
 0
 0
 0
 0
 0
 0
 0
 1.02705006979682E-03
 0
 0
 1.60070603997247E-03
 0
 0
 0
 0
 1.74743854574549E-03
 6.14420749105476E-04
 2.34349806435725E-04
 0

 0
 8.1616538604077E-05
 0
 0
 0
 0
 0
 0
 0
 0
 0
 0
 0
 0
 0
 0
 0
 0
 0
 0
 0
 0

 0
 8.1616538604077E-05
 0
 0
 0
 0
 0
 0
 0
 0
 0
 0
 0
 0
 0
 0
 0
 0
 0
 0
 0
 0

 0
 8.1616538604077E-05
 0
 0
 0
 0
 0
 0
 0
 0
 0
 0
 0
 0
 0
 0
 0
 0
 0
 0
 0
 0

 0
 8.1616538604077E-05
 0
 0
 0
 0
 0
 0
 0
 0
 0
 0
 0
 0
 0
 0
 0
 0
 0
 0
 0
 0

 0
 8.1616538604077E-05
 0
 0
 0
 0
 0
 0
 0
 0
 0
 0
 0
 0
 0
 0
 0
 0
 0
 0
 0
 0

 0
 2.44849615811961E-04
 0
 0
 0
 0
 0
 0
 0
 0
 0
 0
 0
 0
 0
 0
 0
 0
 0
 0
 0
 0

 0
 2.44849615811961E-04
 0
 0
 0
 0
 0
 0
 0
 0
 0
 0
 0
 0
 0
 0
 0
 0
 0
 0
 0
 0

 0
 2.44849615811961E-04
 0
 0
 0
 0
 0
 0
 0
 0
 0
 0
 0
 0
 0
 0
 0
 0
 0
 0
 0
 0

 0
 2.44849615811961E-04
 0
 0
 0
 0
 0
 0
 0
 0
 0
 0
 0
 0
 0
 0
 0
 0
 0
 0
 0
 0

 0
 2.44849615811961E-04
 0
 0
 0
 0
 0
 0
 0
 0
 0
 0
 0
 0
 0
 0
 0
 0
 0
 0
 0
 0

 0
 0
 6.77128626799722E-05
 0
 0
 0
 0
 0
 0
 0
 0
 0
 0
 0
 0
 0
 0
 0
 0
 0
 0
 0
 0

 0
 0
 6.77128626799722E-05
 0
 0
 0
 0
 0
 0
 0
 0
 0
 0
 0
 0
 0
 0
 0
 0
 0
 0
 0
 0

 0
 0
 6.77128626799722E-05
 0
 0
 0
 0
 0
 0
 0
 0
 0
 0
 0
 0
 0
 0
 0
 0
 0
 0
 0
 0

 0
 0
 6.77128626799722E-05
 0
 0
 0
 0
 0
 0
 0
 0
 0
 0
 0
 0
 0
 0
 0
 0
 0
 0
 0
 0

 0
 0
 6.77128626799722E-05
 0
 0
 0
 0
 0
 0
 0
 0
 0
 0
 0
 0
 0
 0
 0
 0
 0
 0
 0
 0

 0
 0
 4.73990038758688E-04
 0
 0
 0
 0
 0
 0
 0
 0
 0
 0
 0
 0
 0
 0
 0
 0
 0
 0
 0
 0

 0
 0
 4.73990038758688E-04
 0
 0
 0
 0
 0
 0
 0
 0
 0
 0
 0
 0
 0
 0
 0
 0
 0
 0
 0
 0

 0
 0
 4.73990038758688E-04
 0
 0
 0
 0
 0
 0
 0
 0
 0
 0
 0
 0
 0
 0
 0
 0
 0
 0
 0
 0

 0
 0
 4.73990038758688E-04
 0
 0
 0
 0
 0
 0
 0
 0
 0
 0
 0
 0
 0
 0
 0
 0
 0
 0
 0
 0

 0
 0
 4.73990038758688E-04
 0
 0
 0
 0
 0
 0
 0
 0
 0
 0
 0
 0
 0
 0
 0
 0
 0
 0
 0
 0

 0
 0
 0
 5.96702580199507E-04
 0
 0
 0
 0
 0
 0
 0
 0
 0
 0
 0
 0
 0
 0
 0
 0
 0
 0
 0
 5.44921205097012E-04

 0
 0
 0
 5.96702580199507E-04
 0
 0
 0
 0
 0
 0
 0
 0
 0
 0
 0
 0
 0
 0
 0
 0
 0
 0
 0
 5.44921205097012E-04

 0
 0
 0
 5.96702580199507E-04
 0
 0
 0
 0
 0
 0
 0
 0
 0
 0
 0
 0
 0
 0
 0
 0
 0
 0
 0
 5.44921205097012E-04

 0
 0
 0
 5.96702580199507E-04
 0
 0
 0
 0
 0
 0
 0
 0
 0
 0
 0
 0
 0
 0
 0
 0
 0
 0
 0
 5.44921205097012E-04

 0
 0
 0
 5.96702580199507E-04
 0
 0
 0
 0
 0
 0
 0
 0
 0
 0
 0
 0
 0
 0
 0
 0
 0
 0
 0
 5.44921205097012E-04

 0
 0
 0
 8.52432257429877E-05
 0
 0
 0
 0
 0
 0
 0
 0
 0
 0
 0
 0
 0
 0
 0
 0
 0
 0
 0
 1.63476361528891E-03

 0
 0
 0
 8.52432257429877E-05
 0
 0
 0
 0
 0
 0
 0
 0
 0
 0
 0
 0
 0
 0
 0
 0
 0
 0
 0
 1.63476361528891E-03

 0
 0
 0
 8.52432257429877E-05
 0
 0
 0
 0
 0
 0
 0
 0
 0
 0
 0
 0
 0
 0
 0
 0
 0
 0
 0
 1.63476361528891E-03

 0
 0
 0
 8.52432257429877E-05
 0
 0
 0
 0
 0
 0
 0
 0
 0
 0
 0
 0
 0
 0
 0
 0
 0
 0
 0
 1.63476361528891E-03

 0
 0
 0
 8.52432257429877E-05
 0
 0
 0
 0
 0
 0
 0
 0
 0
 0
 0
 0
 0
 0
 0
 0
 0
 0
 0
 1.63476361528891E-03

 0
 0
 0
 0
 1.14066241661568E-04
 0
 0
 0
 0
 0
 0
 0
 0
 0
 0
 0
 0
 0
 0
 0
 0
 0
 0
 0
 0

 0
 0
 0
 0
 1.14066241661568E-04
 0
 0
 0
 0
 0
 0
 0
 0
 0
 0
 0
 0
 0
 0
 0
 0
 0
 0
 0
 0

 0
 0
 0
 0
 1.14066241661568E-04
 0
 0
 0
 0
 0
 0
 0
 0
 0
 0
 0
 0
 0
 0
 0
 0
 0
 0
 0
 0

 0
 0
 0
 0
 1.14066241661568E-04
 0
 0
 0
 0
 0
 0
 0
 0
 0
 0
 0
 0
 0
 0
 0
 0
 0
 0
 0
 0

 0
 0
 0
 0
 1.14066241661568E-04
 0
 0
 0
 0
 0
 0
 0
 0
 0
 0
 0
 0
 0
 0
 0
 0
 0
 0
 0
 0

 0
 0
 0
 0
 2.85165604153449E-04
 0
 0
 0
 0
 0
 0
 0
 0
 0
 0
 0
 0
 0
 0
 0
 0
 0
 0
 0
 2.71786831075405E-03

 0
 0
 0
 0
 2.85165604153449E-04
 0
 0
 0
 0
 0
 0
 0
 0
 0
 0
 0
 0
 0
 0
 0
 0
 0
 0
 0
 2.71786831075405E-03

 0
 0
 0
 0
 2.85165604153449E-04
 0
 0
 0
 0
 0
 0
 0
 0
 0
 0
 0
 0
 0
 0
 0
 0
 0
 0
 0
 2.71786831075405E-03

 0
 0
 0
 0
 2.85165604153449E-04
 0
 0
 0
 0
 0
 0
 0
 0
 0
 0
 0
 0
 0
 0
 0
 0
 0
 0
 0
 2.71786831075405E-03

 0
 0
 0
 0
 2.85165604153449E-04
 0
 0
 0
 0
 0
 0
 0
 0
 0
 0
 0
 0
 0
 0
 0
 0
 0
 0
 0
 2.71786831075405E-03

 0
 0
 0
 0
 0
 6.56588729978753E-05
 0
 0
 0
 0
 0
 0
 0
 0
 0
 0
 0
 0
 0
 0
 0
 0
 0
 0
 0
 8.96381399308272E-05

 0
 0
 0
 0
 0
 6.56588729978753E-05
 0
 0
 0
 0
 0
 0
 0
 0
 0
 0
 0
 0
 0
 0
 0
 0
 0
 0
 0
 8.96381399308272E-05

 0
 0
 0
 0
 0
 6.56588729978753E-05
 0
 0
 0
 0
 0
 0
 0
 0
 0
 0
 0
 0
 0
 0
 0
 0
 0
 0
 0
 8.96381399308272E-05

 0
 0
 0
 0
 0
 6.56588729978753E-05
 0
 0
 0
 0
 0
 0
 0
 0
 0
 0
 0
 0
 0
 0
 0
 0
 0
 0
 0
 8.96381399308272E-05

 0
 0
 0
 0
 0
 6.56588729978753E-05
 0
 0
 0
 0
 0
 0
 0
 0
 0
 0
 0
 0
 0
 0
 0
 0
 0
 0
 0
 8.96381399308272E-05

 0
 0
 0
 0
 0
 6.56588729978753E-05
 0
 0
 0
 0
 0
 0
 0
 0
 0
 0
 0
 0
 0
 0
 0
 0
 0
 0
 0
 2.15131535834835E-03

 0
 0
 0
 0
 0
 6.56588729978753E-05
 0
 0
 0
 0
 0
 0
 0
 0
 0
 0
 0
 0
 0
 0
 0
 0
 0
 0
 0
 2.15131535834835E-03

 0
 0
 0
 0
 0
 6.56588729978753E-05
 0
 0
 0
 0
 0
 0
 0
 0
 0
 0
 0
 0
 0
 0
 0
 0
 0
 0
 0
 2.15131535834835E-03

 0
 0
 0
 0
 0
 6.56588729978753E-05
 0
 0
 0
 0
 0
 0
 0
 0
 0
 0
 0
 0
 0
 0
 0
 0
 0
 0
 0
 2.15131535834835E-03

 0
 0
 0
 0
 0
 6.56588729978753E-05
 0
 0
 0
 0
 0
 0
 0
 0
 0
 0
 0
 0
 0
 0
 0
 0
 0
 0
 0
 2.15131535834835E-03

 0
 0
 0
 0
 0
 0
 1.97063383119063E-04
 0
 0
 0
 0
 0
 0
 0
 0
 0
 0
 0
 0
 0
 0
 0
 0
 0
 0
 0
 8.81947872842687E-05

 0
 0
 0
 0
 0
 0
 1.97063383119063E-04
 0
 0
 0
 0
 0
 0
 0
 0
 0
 0
 0
 0
 0
 0
 0
 0
 0
 0
 0
 8.81947872842687E-05

 0
 0
 0
 0
 0
 0
 1.97063383119063E-04
 0
 0
 0
 0
 0
 0
 0
 0
 0
 0
 0
 0
 0
 0
 0
 0
 0
 0
 0
 8.81947872842687E-05

 0
 0
 0
 0
 0
 0
 1.97063383119063E-04
 0
 0
 0
 0
 0
 0
 0
 0
 0
 0
 0
 0
 0
 0
 0
 0
 0
 0
 0
 8.81947872842687E-05

 0
 0
 0
 0
 0
 0
 1.97063383119063E-04
 0
 0
 0
 0
 0
 0
 0
 0
 0
 0
 0
 0
 0
 0
 0
 0
 0
 0
 0
 8.81947872842687E-05

 0
 0
 0
 0
 0
 0
 9.85316915596943E-05
 0
 0
 0
 0
 0
 0
 0
 0
 0
 0
 0
 0
 0
 0
 0
 0
 0
 0
 0
 8.81947872842687E-05

 0
 0
 0
 0
 0
 0
 9.85316915596943E-05
 0
 0
 0
 0
 0
 0
 0
 0
 0
 0
 0
 0
 0
 0
 0
 0
 0
 0
 0
 8.81947872842687E-05

 0
 0
 0
 0
 0
 0
 9.85316915596943E-05
 0
 0
 0
 0
 0
 0
 0
 0
 0
 0
 0
 0
 0
 0
 0
 0
 0
 0
 0
 8.81947872842687E-05

 0
 0
 0
 0
 0
 0
 9.85316915596943E-05
 0
 0
 0
 0
 0
 0
 0
 0
 0
 0
 0
 0
 0
 0
 0
 0
 0
 0
 0
 8.81947872842687E-05

 0
 0
 0
 0
 0
 0
 9.85316915596943E-05
 0
 0
 0
 0
 0
 0
 0
 0
 0
 0
 0
 0
 0
 0
 0
 0
 0
 0
 0
 8.81947872842687E-05

 0
 0
 0
 0
 0
 0
 0
 0
 0
 0
 0
 0
 0
 0
 0
 0
 0
 0
 0
 0
 0
 0
 0
 0
 0
 0
 0
 2.83177156337433E-04

 0
 0
 0
 0
 0
 0
 0
 0
 0
 0
 0
 0
 0
 0
 0
 0
 0
 0
 0
 0
 0
 0
 0
 0
 0
 0
 0
 2.83177156337433E-04

 0
 0
 0
 0
 0
 0
 0
 0
 0
 0
 0
 0
 0
 0
 0
 0
 0
 0
 0
 0
 0
 0
 0
 0
 0
 0
 0
 2.83177156337433E-04

 0
 0
 0
 0
 0
 0
 0
 0
 0
 0
 0
 0
 0
 0
 0
 0
 0
 0
 0
 0
 0
 0
 0
 0
 0
 0
 0
 2.83177156337433E-04

 0
 0
 0
 0
 0
 0
 0
 0
 0
 0
 0
 0
 0
 0
 0
 0
 0
 0
 0
 0
 0
 0
 0
 0
 0
 0
 0
 2.83177156337433E-04

 0
 0
 0
 0
 0
 0
 0
 7.81303860862297E-05
 0
 0
 0
 0
 0
 0
 0
 0
 0
 0
 0
 0
 0
 0
 0
 0
 0
 0
 0
 4.38924592323405E-03

 0
 0
 0
 0
 0
 0
 0
 7.81303860862297E-05
 0
 0
 0
 0
 0
 0
 0
 0
 0
 0
 0
 0
 0
 0
 0
 0
 0
 0
 0
 4.38924592323405E-03

 0
 0
 0
 0
 0
 0
 0
 7.81303860862297E-05
 0
 0
 0
 0
 0
 0
 0
 0
 0
 0
 0
 0
 0
 0
 0
 0
 0
 0
 0
 4.38924592323405E-03

 0
 0
 0
 0
 0
 0
 0
 7.81303860862297E-05
 0
 0
 0
 0
 0
 0
 0
 0
 0
 0
 0
 0
 0
 0
 0
 0
 0
 0
 0
 4.38924592323405E-03

 0
 0
 0
 0
 0
 0
 0
 7.81303860862297E-05
 0
 0
 0
 0
 0
 0
 0
 0
 0
 0
 0
 0
 0
 0
 0
 0
 0
 0
 0
 4.38924592323405E-03

 0
 0
 0
 0
 0
 0
 0
 0
 1.45593335556372E-03
 0
 0
 0
 0
 0
 0
 0
 0
 0
 0
 0
 0
 0
 0
 0
 0
 0
 0
 0
 2.67692713390763E-03

 0
 0
 0
 0
 0
 0
 0
 0
 1.45593335556372E-03
 0
 0
 0
 0
 0
 0
 0
 0
 0
 0
 0
 0
 0
 0
 0
 0
 0
 0
 0
 2.67692713390763E-03

 0
 0
 0
 0
 0
 0
 0
 0
 1.45593335556372E-03
 0
 0
 0
 0
 0
 0
 0
 0
 0
 0
 0
 0
 0
 0
 0
 0
 0
 0
 0
 2.67692713390763E-03

 0
 0
 0
 0
 0
 0
 0
 0
 1.45593335556372E-03
 0
 0
 0
 0
 0
 0
 0
 0
 0
 0
 0
 0
 0
 0
 0
 0
 0
 0
 0
 2.67692713390763E-03

 0
 0
 0
 0
 0
 0
 0
 0
 1.45593335556372E-03
 0
 0
 0
 0
 0
 0
 0
 0
 0
 0
 0
 0
 0
 0
 0
 0
 0
 0
 0
 2.67692713390763E-03

 0
 0
 0
 0
 0
 0
 0
 0
 4.85311118521908E-04
 0
 0
 0
 0
 0
 0
 0
 0
 0
 0
 0
 0
 0
 0
 0
 0
 0
 0
 0
 6.24616331244882E-03

 0
 0
 0
 0
 0
 0
 0
 0
 4.85311118521908E-04
 0
 0
 0
 0
 0
 0
 0
 0
 0
 0
 0
 0
 0
 0
 0
 0
 0
 0
 0
 6.24616331244882E-03

 0
 0
 0
 0
 0
 0
 0
 0
 4.85311118521908E-04
 0
 0
 0
 0
 0
 0
 0
 0
 0
 0
 0
 0
 0
 0
 0
 0
 0
 0
 0
 6.24616331244882E-03

 0
 0
 0
 0
 0
 0
 0
 0
 4.85311118521908E-04
 0
 0
 0
 0
 0
 0
 0
 0
 0
 0
 0
 0
 0
 0
 0
 0
 0
 0
 0
 6.24616331244882E-03

 0
 0
 0
 0
 0
 0
 0
 0
 4.85311118521908E-04
 0
 0
 0
 0
 0
 0
 0
 0
 0
 0
 0
 0
 0
 0
 0
 0
 0
 0
 0
 6.24616331244882E-03

 0
 0
 0
 0
 0
 0
 0
 0
 0
 1.86633971131515E-04
 0
 0
 0
 0
 0
 0
 0
 0
 0
 0
 0
 0
 0
 0
 0
 0
 0
 0
 0
 0

 0
 0
 0
 0
 0
 0
 0
 0
 0
 1.86633971131515E-04
 0
 0
 0
 0
 0
 0
 0
 0
 0
 0
 0
 0
 0
 0
 0
 0
 0
 0
 0
 0

 0
 0
 0
 0
 0
 0
 0
 0
 0
 1.86633971131515E-04
 0
 0
 0
 0
 0
 0
 0
 0
 0
 0
 0
 0
 0
 0
 0
 0
 0
 0
 0
 0

 0
 0
 0
 0
 0
 0
 0
 0
 0
 1.86633971131515E-04
 0
 0
 0
 0
 0
 0
 0
 0
 0
 0
 0
 0
 0
 0
 0
 0
 0
 0
 0
 0

 0
 0
 0
 0
 0
 0
 0
 0
 0
 1.86633971131515E-04
 0
 0
 0
 0
 0
 0
 0
 0
 0
 0
 0
 0
 0
 0
 0
 0
 0
 0
 0
 0

 0
 0
 0
 0
 0
 0
 0
 0
 0
 3.73267942262415E-04
 0
 0
 0
 0
 0
 0
 0
 0
 0
 0
 0
 0
 0
 0
 0
 0
 0
 0
 0
 1.50598447440343E-02

 0
 0
 0
 0
 0
 0
 0
 0
 0
 3.73267942262415E-04
 0
 0
 0
 0
 0
 0
 0
 0
 0
 0
 0
 0
 0
 0
 0
 0
 0
 0
 0
 1.50598447440343E-02

 0
 0
 0
 0
 0
 0
 0
 0
 0
 3.73267942262415E-04
 0
 0
 0
 0
 0
 0
 0
 0
 0
 0
 0
 0
 0
 0
 0
 0
 0
 0
 0
 1.50598447440343E-02

 0
 0
 0
 0
 0
 0
 0
 0
 0
 3.73267942262415E-04
 0
 0
 0
 0
 0
 0
 0
 0
 0
 0
 0
 0
 0
 0
 0
 0
 0
 0
 0
 1.50598447440343E-02

 0
 0
 0
 0
 0
 0
 0
 0
 0
 3.73267942262415E-04
 0
 0
 0
 0
 0
 0
 0
 0
 0
 0
 0
 0
 0
 0
 0
 0
 0
 0
 0
 1.50598447440343E-02

 0
 0
 0
 0
 0
 0
 0
 0
 0
 0
 6.51431834138888E-04
 0
 0
 0
 0
 0
 0
 0
 0
 0
 0
 0
 0
 0
 0
 0
 0
 0
 0
 0
 7.97786575100341E-05

 0
 0
 0
 0
 0
 0
 0
 0
 0
 0
 6.51431834138888E-04
 0
 0
 0
 0
 0
 0
 0
 0
 0
 0
 0
 0
 0
 0
 0
 0
 0
 0
 0
 7.97786575100341E-05

 0
 0
 0
 0
 0
 0
 0
 0
 0
 0
 6.51431834138888E-04
 0
 0
 0
 0
 0
 0
 0
 0
 0
 0
 0
 0
 0
 0
 0
 0
 0
 0
 0
 7.97786575100341E-05

 0
 0
 0
 0
 0
 0
 0
 0
 0
 0
 6.51431834138888E-04
 0
 0
 0
 0
 0
 0
 0
 0
 0
 0
 0
 0
 0
 0
 0
 0
 0
 0
 0
 7.97786575100341E-05

 0
 0
 0
 0
 0
 0
 0
 0
 0
 0
 6.51431834138888E-04
 0
 0
 0
 0
 0
 0
 0
 0
 0
 0
 0
 0
 0
 0
 0
 0
 0
 0
 0
 7.97786575100341E-05

 0
 0
 0
 0
 0
 0
 0
 0
 0
 0
 5.58370143548935E-04
 0
 0
 0
 0
 0
 0
 0
 0
 0
 0
 0
 0
 0
 0
 0
 0
 0
 0
 0
 3.19114630040136E-04

 0
 0
 0
 0
 0
 0
 0
 0
 0
 0
 5.58370143548935E-04
 0
 0
 0
 0
 0
 0
 0
 0
 0
 0
 0
 0
 0
 0
 0
 0
 0
 0
 0
 3.19114630040136E-04

 0
 0
 0
 0
 0
 0
 0
 0
 0
 0
 5.58370143548935E-04
 0
 0
 0
 0
 0
 0
 0
 0
 0
 0
 0
 0
 0
 0
 0
 0
 0
 0
 0
 3.19114630040136E-04

 0
 0
 0
 0
 0
 0
 0
 0
 0
 0
 5.58370143548935E-04
 0
 0
 0
 0
 0
 0
 0
 0
 0
 0
 0
 0
 0
 0
 0
 0
 0
 0
 0
 3.19114630040136E-04

 0
 0
 0
 0
 0
 0
 0
 0
 0
 0
 5.58370143548935E-04
 0
 0
 0
 0
 0
 0
 0
 0
 0
 0
 0
 0
 0
 0
 0
 0
 0
 0
 0
 3.19114630040136E-04

 0
 0
 0
 0
 0
 0
 0
 0
 0
 0
 0
 5.42527748098335E-04
 0
 0
 0
 0
 0
 0
 0
 0
 0
 0
 0
 0
 0
 0
 0
 0
 0
 0
 0
 0

 0
 0
 0
 0
 0
 0
 0
 0
 0
 0
 0
 5.42527748098335E-04
 0
 0
 0
 0
 0
 0
 0
 0
 0
 0
 0
 0
 0
 0
 0
 0
 0
 0
 0
 0

 0
 0
 0
 0
 0
 0
 0
 0
 0
 0
 0
 5.42527748098335E-04
 0
 0
 0
 0
 0
 0
 0
 0
 0
 0
 0
 0
 0
 0
 0
 0
 0
 0
 0
 0

 0
 0
 0
 0
 0
 0
 0
 0
 0
 0
 0
 5.42527748098335E-04
 0
 0
 0
 0
 0
 0
 0
 0
 0
 0
 0
 0
 0
 0
 0
 0
 0
 0
 0
 0

 0
 0
 0
 0
 0
 0
 0
 0
 0
 0
 0
 5.42527748098335E-04
 0
 0
 0
 0
 0
 0
 0
 0
 0
 0
 0
 0
 0
 0
 0
 0
 0
 0
 0
 0

 0
 0
 0
 0
 0
 0
 0
 0
 0
 0
 0
 3.25516648859717E-04
 0
 0
 0
 0
 0
 0
 0
 0
 0
 0
 0
 0
 0
 0
 0
 0
 0
 0
 0
 0

 0
 0
 0
 0
 0
 0
 0
 0
 0
 0
 0
 3.25516648859717E-04
 0
 0
 0
 0
 0
 0
 0
 0
 0
 0
 0
 0
 0
 0
 0
 0
 0
 0
 0
 0

 0
 0
 0
 0
 0
 0
 0
 0
 0
 0
 0
 3.25516648859717E-04
 0
 0
 0
 0
 0
 0
 0
 0
 0
 0
 0
 0
 0
 0
 0
 0
 0
 0
 0
 0

 0
 0
 0
 0
 0
 0
 0
 0
 0
 0
 0
 3.25516648859717E-04
 0
 0
 0
 0
 0
 0
 0
 0
 0
 0
 0
 0
 0
 0
 0
 0
 0
 0
 0
 0

 0
 0
 0
 0
 0
 0
 0
 0
 0
 0
 0
 3.25516648859717E-04
 0
 0
 0
 0
 0
 0
 0
 0
 0
 0
 0
 0
 0
 0
 0
 0
 0
 0
 0
 0

 0
 0
 0
 0
 0
 0
 0
 0
 0
 0
 0
 0
 6.40868021972184E-04

 0
 0
 0
 0
 0
 0
 0
 0
 0
 0
 0
 0
 6.40868021972184E-04

 0
 0
 0
 0
 0
 0
 0
 0
 0
 0
 0
 0
 6.40868021972184E-04

 0
 0
 0
 0
 0
 0
 0
 0
 0
 0
 0
 0
 6.40868021972184E-04

 0
 0
 0
 0
 0
 0
 0
 0
 0
 0
 0
 0
 6.40868021972184E-04

 0
 0
 0
 0
 0
 0
 0
 0
 0
 0
 0
 0
 2.81981929666491E-03

 0
 0
 0
 0
 0
 0
 0
 0
 0
 0
 0
 0
 2.81981929666491E-03

 0
 0
 0
 0
 0
 0
 0
 0
 0
 0
 0
 0
 2.81981929666491E-03

 0
 0
 0
 0
 0
 0
 0
 0
 0
 0
 0
 0
 2.81981929666491E-03

 0
 0
 0
 0
 0
 0
 0
 0
 0
 0
 0
 0
 2.81981929666491E-03

 0
 0
 0
 0
 0
 0
 0
 0
 0
 0
 0
 0
 0
 9.95588557307854E-04

 0
 0
 0
 0
 0
 0
 0
 0
 0
 0
 0
 0
 0
 9.95588557307854E-04

 0
 0
 0
 0
 0
 0
 0
 0
 0
 0
 0
 0
 0
 9.95588557307854E-04

 0
 0
 0
 0
 0
 0
 0
 0
 0
 0
 0
 0
 0
 9.95588557307854E-04

 0
 0
 0
 0
 0
 0
 0
 0
 0
 0
 0
 0
 0
 9.95588557307854E-04

 0
 0
 0
 0
 0
 0
 0
 0
 0
 0
 0
 0
 0
 0

 0
 0
 0
 0
 0
 0
 0
 0
 0
 0
 0
 0
 0
 0

 0
 0
 0
 0
 0
 0
 0
 0
 0
 0
 0
 0
 0
 0

 0
 0
 0
 0
 0
 0
 0
 0
 0
 0
 0
 0
 0
 0

 0
 0
 0
 0
 0
 0
 0
 0
 0
 0
 0
 0
 0
 0

 0
 0
 0
 0
 0
 0
 0
 0
 0
 0
 0
 0
 0
 0
 1.40430675707957E-03

 0
 0
 0
 0
 0
 0
 0
 0
 0
 0
 0
 0
 0
 0
 1.40430675707957E-03

 0
 0
 0
 0
 0
 0
 0
 0
 0
 0
 0
 0
 0
 0
 1.40430675707957E-03

 0
 0
 0
 0
 0
 0
 0
 0
 0
 0
 0
 0
 0
 0
 1.40430675707957E-03

 0
 0
 0
 0
 0
 0
 0
 0
 0
 0
 0
 0
 0
 0
 1.40430675707957E-03

 0
 0
 0
 0
 0
 0
 0
 0
 0
 0
 0
 0
 0
 0
 1.26387608137138E-03

 0
 0
 0
 0
 0
 0
 0
 0
 0
 0
 0
 0
 0
 0
 1.26387608137138E-03

 0
 0
 0
 0
 0
 0
 0
 0
 0
 0
 0
 0
 0
 0
 1.26387608137138E-03

 0
 0
 0
 0
 0
 0
 0
 0
 0
 0
 0
 0
 0
 0
 1.26387608137138E-03

 0
 0
 0
 0
 0
 0
 0
 0
 0
 0
 0
 0
 0
 0
 1.26387608137138E-03

 0
 0
 0
 0
 0
 0
 0
 0
 0
 0
 0
 0
 0
 0
 0
 2.97266250952974E-04

 0
 0
 0
 0
 0
 0
 0
 0
 0
 0
 0
 0
 0
 0
 0
 2.97266250952974E-04

 0
 0
 0
 0
 0
 0
 0
 0
 0
 0
 0
 0
 0
 0
 0
 2.97266250952974E-04

 0
 0
 0
 0
 0
 0
 0
 0
 0
 0
 0
 0
 0
 0
 0
 2.97266250952974E-04

 0
 0
 0
 0
 0
 0
 0
 0
 0
 0
 0
 0
 0
 0
 0
 2.97266250952974E-04

 0
 0
 0
 0
 0
 0
 0
 0
 0
 0
 0
 0
 0
 0
 0
 9.90887503177669E-05

 0
 0
 0
 0
 0
 0
 0
 0
 0
 0
 0
 0
 0
 0
 0
 9.90887503177669E-05

 0
 0
 0
 0
 0
 0
 0
 0
 0
 0
 0
 0
 0
 0
 0
 9.90887503177669E-05

 0
 0
 0
 0
 0
 0
 0
 0
 0
 0
 0
 0
 0
 0
 0
 9.90887503177669E-05

 0
 0
 0
 0
 0
 0
 0
 0
 0
 0
 0
 0
 0
 0
 0
 9.90887503177669E-05

 0
 0
 0
 0
 0
 0
 0
 0
 0
 0
 0
 0
 0
 0
 0
 0
 2.12924717670156E-03

 0
 0
 0
 0
 0
 0
 0
 0
 0
 0
 0
 0
 0
 0
 0
 0
 2.12924717670156E-03

 0
 0
 0
 0
 0
 0
 0
 0
 0
 0
 0
 0
 0
 0
 0
 0
 2.12924717670156E-03

 0
 0
 0
 0
 0
 0
 0
 0
 0
 0
 0
 0
 0
 0
 0
 0
 2.12924717670156E-03

 0
 0
 0
 0
 0
 0
 0
 0
 0
 0
 0
 0
 0
 0
 0
 0
 2.12924717670156E-03

 0
 0
 0
 0
 0
 0
 0
 0
 0
 0
 0
 0
 0
 0
 0
 0
 1.96545893233969E-03

 0
 0
 0
 0
 0
 0
 0
 0
 0
 0
 0
 0
 0
 0
 0
 0
 1.96545893233969E-03

 0
 0
 0
 0
 0
 0
 0
 0
 0
 0
 0
 0
 0
 0
 0
 0
 1.96545893233969E-03

 0
 0
 0
 0
 0
 0
 0
 0
 0
 0
 0
 0
 0
 0
 0
 0
 1.96545893233969E-03

 0
 0
 0
 0
 0
 0
 0
 0
 0
 0
 0
 0
 0
 0
 0
 0
 1.96545893233969E-03

 0
 0
 0
 0
 0
 0
 0
 0
 0
 0
 0
 0
 0
 0
 0
 0
 0
 6.72579124006933E-04

 0
 0
 0
 0
 0
 0
 0
 0
 0
 0
 0
 0
 0
 0
 0
 0
 0
 6.72579124006933E-04

 0
 0
 0
 0
 0
 0
 0
 0
 0
 0
 0
 0
 0
 0
 0
 0
 0
 6.72579124006933E-04

 0
 0
 0
 0
 0
 0
 0
 0
 0
 0
 0
 0
 0
 0
 0
 0
 0
 6.72579124006933E-04

 0
 0
 0
 0
 0
 0
 0
 0
 0
 0
 0
 0
 0
 0
 0
 0
 0
 6.72579124006933E-04

 0
 0
 0
 0
 0
 0
 0
 0
 0
 0
 0
 0
 0
 0
 0
 0
 0
 0

 0
 0
 0
 0
 0
 0
 0
 0
 0
 0
 0
 0
 0
 0
 0
 0
 0
 0

 0
 0
 0
 0
 0
 0
 0
 0
 0
 0
 0
 0
 0
 0
 0
 0
 0
 0

 0
 0
 0
 0
 0
 0
 0
 0
 0
 0
 0
 0
 0
 0
 0
 0
 0
 0

 0
 0
 0
 0
 0
 0
 0
 0
 0
 0
 0
 0
 0
 0
 0
 0
 0
 0

 0
 0
 0
 0
 0
 0
 0
 0
 0
 0
 0
 0
 0
 0
 0
 0
 0
 0
 4.30469488180464E-04

 0
 0
 0
 0
 0
 0
 0
 0
 0
 0
 0
 0
 0
 0
 0
 0
 0
 0
 4.30469488180464E-04

 0
 0
 0
 0
 0
 0
 0
 0
 0
 0
 0
 0
 0
 0
 0
 0
 0
 0
 4.30469488180464E-04

 0
 0
 0
 0
 0
 0
 0
 0
 0
 0
 0
 0
 0
 0
 0
 0
 0
 0
 4.30469488180464E-04

 0
 0
 0
 0
 0
 0
 0
 0
 0
 0
 0
 0
 0
 0
 0
 0
 0
 0
 4.30469488180464E-04

 0
 0
 0
 0
 0
 0
 0
 0
 0
 0
 0
 0
 0
 0
 0
 0
 0
 0
 4.30469488180464E-04

 0
 0
 0
 0
 0
 0
 0
 0
 0
 0
 0
 0
 0
 0
 0
 0
 0
 0
 4.30469488180464E-04

 0
 0
 0
 0
 0
 0
 0
 0
 0
 0
 0
 0
 0
 0
 0
 0
 0
 0
 4.30469488180464E-04

 0
 0
 0
 0
 0
 0
 0
 0
 0
 0
 0
 0
 0
 0
 0
 0
 0
 0
 4.30469488180464E-04

 0
 0
 0
 0
 0
 0
 0
 0
 0
 0
 0
 0
 0
 0
 0
 0
 0
 0
 4.30469488180464E-04

 0
 0
 0
 0
 0
 0
 0
 0
 0
 0
 0
 0
 0
 0
 0
 0
 0
 0
 0
 8.48916673631092E-04

 0
 0
 0
 0
 0
 0
 0
 0
 0
 0
 0
 0
 0
 0
 0
 0
 0
 0
 0
 8.48916673631092E-04

 0
 0
 0
 0
 0
 0
 0
 0
 0
 0
 0
 0
 0
 0
 0
 0
 0
 0
 0
 8.48916673631092E-04

 0
 0
 0
 0
 0
 0
 0
 0
 0
 0
 0
 0
 0
 0
 0
 0
 0
 0
 0
 8.48916673631092E-04

 0
 0
 0
 0
 0
 0
 0
 0
 0
 0
 0
 0
 0
 0
 0
 0
 0
 0
 0
 8.48916673631092E-04

 0
 0
 0
 0
 0
 0
 0
 0
 0
 0
 0
 0
 0
 0
 0
 0
 0
 0
 0
 4.24458336814145E-04

 0
 0
 0
 0
 0
 0
 0
 0
 0
 0
 0
 0
 0
 0
 0
 0
 0
 0
 0
 4.24458336814145E-04

 0
 0
 0
 0
 0
 0
 0
 0
 0
 0
 0
 0
 0
 0
 0
 0
 0
 0
 0
 4.24458336814145E-04

 0
 0
 0
 0
 0
 0
 0
 0
 0
 0
 0
 0
 0
 0
 0
 0
 0
 0
 0
 4.24458336814145E-04

 0
 0
 0
 0
 0
 0
 0
 0
 0
 0
 0
 0
 0
 0
 0
 0
 0
 0
 0
 4.24458336814145E-04

 0
 0
 0
 0
 0
 0
 0
 0
 0
 0
 0
 0
 0
 0
 0
 0
 0
 0
 0
 0
 3.49634066313811E-04
 .024112716041385
 8.70970601378185E-03
 1.08984241019261E-03
 9.12427218610374E-03
 1.79276279862362E-04
 7.93753085555145E-04
 0
 1.18974539284939E-03
 8.36658041334001E-04
 2.39335972530102E-04
 8.67187684293454E-05

 0
 0
 0
 0
 0
 0
 0
 0
 0
 0
 0
 0
 0
 0
 0
 0
 0
 0
 0
 0
 3.49634066313811E-04
 .024112716041385
 8.70970601378185E-03
 1.08984241019261E-03
 9.12427218610374E-03
 1.79276279862362E-04
 7.93753085555145E-04
 0
 1.18974539284939E-03
 8.36658041334001E-04
 2.39335972530102E-04
 8.67187684293454E-05

 0
 0
 0
 0
 0
 0
 0
 0
 0
 0
 0
 0
 0
 0
 0
 0
 0
 0
 0
 0
 3.49634066313811E-04
 .024112716041385
 8.70970601378185E-03
 1.08984241019261E-03
 9.12427218610374E-03
 1.79276279862362E-04
 7.93753085555145E-04
 0
 1.18974539284939E-03
 8.36658041334001E-04
 2.39335972530102E-04
 8.67187684293454E-05

 0
 0
 0
 0
 0
 0
 0
 0
 0
 0
 0
 0
 0
 0
 0
 0
 0
 0
 0
 0
 3.49634066313811E-04
 .024112716041385
 8.70970601378185E-03
 1.08984241019261E-03
 9.12427218610374E-03
 1.79276279862362E-04
 7.93753085555145E-04
 0
 1.18974539284939E-03
 8.36658041334001E-04
 2.39335972530102E-04
 8.67187684293454E-05

 0
 0
 0
 0
 0
 0
 0
 0
 0
 0
 0
 0
 0
 0
 0
 0
 0
 0
 0
 0
 3.49634066313811E-04
 .024112716041385
 8.70970601378185E-03
 1.08984241019261E-03
 9.12427218610374E-03
 1.79276279862362E-04
 7.93753085555145E-04
 0
 1.18974539284939E-03
 8.36658041334001E-04
 2.39335972530102E-04
 8.67187684293454E-05

 0
 0
 0
 0
 0
 0
 0
 0
 0
 0
 0
 0
 0
 0
 0
 0
 0
 0
 0
 0
 0
 0
 0
 1.28056483197798E-03
 0
 0
 0
 0
 0
 0
 0
 0

 0
 0
 0
 0
 0
 0
 0
 0
 0
 0
 0
 0
 0
 0
 0
 0
 0
 0
 0
 0
 0
 0
 0
 1.28056483197798E-03
 0
 0
 0
 0
 0
 0
 0
 0

 0
 0
 0
 0
 0
 0
 0
 0
 0
 0
 0
 0
 0
 0
 0
 0
 0
 0
 0
 0
 0

 0
 0
 0
 0
 0
 0
 0
 0
 0
 0
 0
 0
 0
 0
 0
 0
 0
 0
 0
 0
 0

 0
 0
 0
 0
 0
 0
 0
 0
 0
 0
 0
 0
 0
 0
 0
 0
 0
 0
 0
 0
 0

 0
 0
 0
 0
 0
 0
 0
 0
 0
 0
 0
 0
 0
 0
 0
 0
 0
 0
 0
 0
 0
 0

 0
 0
 0
 0
 0
 0
 0
 0
 0
 0
 0
 0
 0
 0
 0
 0
 0
 0
 0
 0
 0
 0

 0
 0
 0
 0
 0
 0
 0
 0
 0
 0
 0
 0
 0
 0
 0
 0
 0
 0
 0
 0
 0
 0

 0
 0
 0
 0
 0
 0
 0
 0
 0
 0
 0
 0
 0
 0
 0
 0
 0
 0
 0
 0
 0
 0
 0

 0
 0
 0
 0
 0
 0
 0
 0
 0
 0
 0
 0
 0
 0
 0
 0
 0
 0
 0
 0
 0
 0
 0

 0
 0
 0
 0
 0
 0
 0
 0
 0
 0
 0
 0
 0
 0
 0
 0
 0
 0
 0
 0
 0
 0
 0

 0
 0
 0
 0
 0
 0
 0
 0
 0
 0
 0
 0
 0
 0
 0
 0
 0
 0
 0
 0
 0
 0
 0
 1.28056483197798E-03

 0
 0
 0
 0
 0
 0
 0
 0
 0
 0
 0
 0
 0
 0
 0
 0
 0
 0
 0
 0
 0
 0
 0
 1.28056483197798E-03

 0
 0
 0
 0
 0
 0
 0
 0
 0
 0
 0
 0
 0
 0
 0
 0
 0
 0
 0
 0
 0
 0
 0
 1.28056483197798E-03

 0
 0
 0
 0
 0
 0
 0
 0
 0
 0
 0
 0
 0
 0
 0
 0
 0
 0
 0
 0
 0
 0
 0
 0
 0

 0
 0
 0
 0
 0
 0
 0
 0
 0
 0
 0
 0
 0
 0
 0
 0
 0
 0
 0
 0
 0
 0
 0
 0
 0

 0
 0
 0
 0
 0
 0
 0
 0
 0
 0
 0
 0
 0
 0
 0
 0
 0
 0
 0
 0
 0
 0
 0
 0
 0

 0
 0
 0
 0
 0
 0
 0
 0
 0
 0
 0
 0
 0
 0
 0
 0
 0
 0
 0
 0
 0
 0
 0
 0
 0
 0

 0
 0
 0
 0
 0
 0
 0
 0
 0
 0
 0
 0
 0
 0
 0
 0
 0
 0
 0
 0
 0
 0
 0
 0
 0
 0

 0
 0
 0
 0
 0
 0
 0
 0
 0
 0
 0
 0
 0
 0
 0
 0
 0
 0
 0
 0
 0
 0
 0
 0
 0
 0

 0
 0
 0
 0
 0
 0
 0
 0
 0
 0
 0
 0
 0
 0
 0
 0
 0
 0
 0
 0
 0
 0
 0
 0
 0
 0
 0

 0
 0
 0
 0
 0
 0
 0
 0
 0
 0
 0
 0
 0
 0
 0
 0
 0
 0
 0
 0
 0
 0
 0
 0
 0
 0
 0

 0
 0
 0
 0
 0
 0
 0
 0
 0
 0
 0
 0
 0
 0
 0
 0
 0
 0
 0
 0
 0
 0
 0
 0
 0
 0
 0

 0
 0
 0
 0
 0
 0
 0
 0
 0
 0
 0
 0
 0
 0
 0
 0
 0
 0
 0
 0
 0
 0
 0
 0
 0
 0
 0
 0

 0
 0
 0
 0
 0
 0
 0
 0
 0
 0
 0
 0
 0
 0
 0
 0
 0
 0
 0
 0
 0
 0
 0
 0
 0
 0
 0
 0

 0
 0
 0
 0
 0
 0
 0
 0
 0
 0
 0
 0
 0
 0
 0
 0
 0
 0
 0
 0
 0
 0
 0
 0
 0
 0
 0
 0

 0
 0
 0
 0
 0
 0
 0
 0
 0
 0
 0
 0
 0
 0
 0
 0
 0
 0
 0
 0
 0
 0
 0
 0
 0
 0
 0
 0
 0

 0
 0
 0
 0
 0
 0
 0
 0
 0
 0
 0
 0
 0
 0
 0
 0
 0
 0
 0
 0
 0
 0
 0
 0
 0
 0
 0
 0
 0

 0
 0
 0
 0
 0
 0
 0
 0
 0
 0
 0
 0
 0
 0
 0
 0
 0
 0
 0
 0
 0
 0
 0
 0
 0
 0
 0
 0
 0

 0
 0
 0
 0
 0
 0
 0
 0
 0
 0
 0
 0
 0
 0
 0
 0
 0
 0
 0
 0
 0
 0
 0
 0
 0
 0
 0
 0
 0
 0

 0
 0
 0
 0
 0
 0
 0
 0
 0
 0
 0
 0
 0
 0
 0
 0
 0
 0
 0
 0
 0
 0
 0
 0
 0
 0
 0
 0
 0
 0

 0
 0
 0
 0
 0
 0
 0
 0
 0
 0
 0
 0
 0
 0
 0
 0
 0
 0
 0
 0
 0
 0
 0
 0
 0
 0
 0
 0
 0
 0

 0
 0
 0
 0
 0
 0
 0
 0
 0
 0
 0
 0
 0
 0
 0
 0
 0
 0
 0
 0
 0
 0
 0
 0
 0
 0
 0
 0
 0
 0
 0

 0
 0
 0
 0
 0
 0
 0
 0
 0
 0
 0
 0
 0
 0
 0
 0
 0
 0
 0
 0
 0
 0
 0
 0
 0
 0
 0
 0
 0
 0
 0

 0
 0
 0
 0
 0
 0
 0
 0
 0
 0
 0
 0
 0
 0
 0
 0
 0
 0
 0
 0
 0
 0
 0
 0
 0
 0
 0
 0
 0
 0
 0

 0
 0
 0
 0
 0
 0
 0
 0
 0
 0
 0
 0
 0
 0
 0
 0
 0
 0
 0
 0
 0
 0
 0
 0
 0
 0
 0
 0
 0
 0
 0
 0

 0
 0
 0
 0
 0
 0
 0
 0
 0
 0
 0
 0
 0
 0
 0
 0
 0
 0
 0
 0
 0
 0
 0
 0
 0
 0
 0
 0
 0
 0
 0
 0

 0
 0
 0
 0
 0
 0
 0
 0
 0
 0
 0
 0
 0
 0
 0
 0
 0
 0
 0
 0
 0
 0
 0
 0
 0
 0
 0
 0
 0
 0
 0
 0

 0
 0
 0
 0
 0
 0
 0
 0
 0
 0
 0
 0
 0
 0
 0
 0
 0
 0
 0
 0
 1.74817033157218E-04

 0
 0
 0
 0
 0
 0
 0
 0
 0
 0
 0
 0
 0
 0
 0
 0
 0
 0
 0
 0
 1.74817033157218E-04

 0
 0
 0
 0
 0
 0
 0
 0
 0
 0
 0
 0
 0
 0
 0
 0
 0
 0
 0
 0
 1.74817033157218E-04

 0
 0
 0
 0
 0
 0
 0
 0
 0
 0
 0
 0
 0
 0
 0
 0
 0
 0
 0
 0
 1.74817033157218E-04

 0
 0
 0
 0
 0
 0
 0
 0
 0
 0
 0
 0
 0
 0
 0
 0
 0
 0
 0
 0
 1.74817033157218E-04

 0
 0
 0
 0
 0
 0
 0
 0
 0
 0
 0
 0
 0
 0
 0
 0
 0
 0
 0
 0
 0
 0
 0
 0
 0
 0
 0
 0
 0
 0
 0
 0

 0
 0
 0
 0
 0
 0
 0
 0
 0
 0
 0
 0
 0
 0
 0
 0
 0
 0
 0
 0
 0
 0
 0
 0
 0
 0
 0
 0
 0
 0
 0
 0

 0
 0
 0
 0
 0
 0
 0
 0
 0
 0
 0
 0
 0
 0
 0
 0
 0
 0
 0
 0
 0
 0
 0
 0
 0
 0
 0
 0
 0
 0
 0
 0

 0
 0
 0
 0
 0
 0
 0
 0
 0
 0
 0
 0
 0
 0
 0
 0
 0
 0
 0
 0
 0
 0
 0
 0
 0
 0
 0
 0
 0
 0
 0
 0

 0
 0
 0
 0
 0
 0
 0
 0
 0
 0
 0
 0
 0
 0
 0
 0
 0
 0
 0
 0
 0
 0
 0
 0
 0
 0
 0
 0
 0
 0
 0
 0

 0
 0
 0
 0
 0
 0
 0
 0
 0
 0
 0
 0
 0
 0
 0
 0
 0
 0
 0
 0
 0
 1.07933109899731E-03
 0
 0
 0
 8.42598515353103E-04
 0
 0
 0
 0
 1.12487907089148E-03
 4.07578211617924E-04

 0
 0
 0
 0
 0
 0
 0
 0
 0
 0
 0
 0
 0
 0
 0
 0
 0
 0
 0
 0
 0
 0
 0
 0
 0
 8.42598515353103E-04
 0
 0
 0
 0
 1.12487907089148E-03
 0

 0
 0
 0
 0
 0
 0
 0
 0
 0
 0
 0
 0
 0
 0
 0
 0
 0
 0
 0
 0
 0
 0
 0
 0
 0
 8.42598515353103E-04
 0
 0
 0
 0
 1.12487907089148E-03
 0

 0
 0
 0
 0
 0
 0
 0
 0
 0
 0
 0
 0
 0
 0
 0
 0
 0
 0
 0
 0
 0
 0
 0
 0
 0
 8.42598515353103E-04
 0
 0
 0
 0
 1.12487907089148E-03
 0

 0
 0
 0
 0
 0
 0
 0
 0
 0
 0
 0
 0
 0
 0
 0
 0
 0
 0
 0
 0
 0
 0
 0
 0
 0
 8.42598515353103E-04
 0
 0
 0
 0
 1.12487907089148E-03
 0

 0
 0
 0
 0
 0
 0
 0
 0
 0
 0
 0
 0
 0
 0
 0
 0
 0
 0
 0
 0
 0
 1.07933109899731E-03
 0
 0
 0
 0
 0
 0
 0
 0
 0
 4.07578211617924E-04

 0
 0
 0
 0
 0
 0
 0
 0
 0
 0
 0
 0
 0
 0
 0
 0
 0
 0
 0
 0
 0
 1.07933109899731E-03
 0
 0
 0
 0
 0
 0
 0
 0
 0
 4.07578211617924E-04

 0
 0
 0
 0
 0
 0
 0
 0
 0
 0
 0
 0
 0
 0
 0
 0
 0
 0
 0
 0
 0
 1.07933109899731E-03
 0
 0
 0
 0
 0
 0
 0
 0
 0
 4.07578211617924E-04

 0
 0
 0
 0
 0
 0
 0
 0
 0
 0
 0
 0
 0
 0
 0
 0
 0
 0
 0
 0
 0
 1.07933109899731E-03
 0
 0
 0
 0
 0
 0
 0
 0
 0
 4.07578211617924E-04

 0
 0
 0
 0
 0
 0
 0
 0
 0
 0
 0
 0
 0
 0
 0
 0
 0
 0
 0
 0
 0
 2.29644914680279E-04

 0
 0
 0
 0
 0
 0
 0
 0
 0
 0
 0
 0
 0
 0
 0
 0
 0
 0
 0
 0
 0
 2.29644914680279E-04

 0
 0
 0
 0
 0
 0
 0
 0
 0
 0
 0
 0
 0
 0
 0
 0
 0
 0
 0
 0
 0
 2.29644914680279E-04

 0
 0
 0
 0
 0
 0
 0
 0
 0
 0
 0
 0
 0
 0
 0
 0
 0
 0
 0
 0
 0
 2.29644914680279E-04

 0
 0
 0
 0
 0
 0
 0
 0
 0
 0
 0
 0
 0
 0
 0
 0
 0
 0
 0
 0
 0
 2.29644914680279E-04

 0
 0
 0
 0
 0
 0
 0
 0
 0
 0
 0
 0
 0
 0
 0
 0
 0
 0
 0
 0
 0
 0
 0

 0
 0
 0
 0
 0
 0
 0
 0
 0
 0
 0
 0
 0
 0
 0
 0
 0
 0
 0
 0
 0
 0
 0

 0
 0
 0
 0
 0
 0
 0
 0
 0
 0
 0
 0
 0
 0
 0
 0
 0
 0
 0
 0
 0
 0
 0

 0
 0
 0
 0
 0
 0
 0
 0
 0
 0
 0
 0
 0
 0
 0
 0
 0
 0
 0
 0
 0
 0
 0

 0
 0
 0
 0
 0
 0
 0
 0
 0
 0
 0
 0
 0
 0
 0
 0
 0
 0
 0
 0
 0
 0
 0

 0
 0
 0
 0
 0
 0
 0
 0
 0
 0
 0
 0
 0
 0
 0
 0
 0
 0
 0
 0
 0
 0
 0
 .161841597914091

 0
 0
 0
 0
 0
 0
 0
 0
 0
 0
 0
 0
 0
 0
 0
 0
 0
 0
 0
 0
 0
 0
 0
 .161841597914091

 0
 0
 0
 0
 0
 0
 0
 0
 0
 0
 0
 0
 0
 0
 0
 0
 0
 0
 0
 0
 0
 0
 0
 .161841597914091

 0
 0
 0
 0
 0
 0
 0
 0
 0
 0
 0
 0
 0
 0
 0
 0
 0
 0
 0
 0
 0
 0
 0
 .161841597914091

 0
 0
 0
 0
 0
 0
 0
 0
 0
 0
 0
 0
 0
 0
 0
 0
 0
 0
 0
 0
 0
 0
 0
 .161841597914091

 0
 0
 0
 0
 0
 0
 0
 0
 0
 0
 0
 0
 0
 0
 0
 0
 0
 0
 0
 0
 0
 0
 0
 0
 5.24160317073735E-03

 0
 0
 0
 0
 0
 0
 0
 0
 0
 0
 0
 0
 0
 0
 0
 0
 0
 0
 0
 0
 0
 0
 0
 0
 5.24160317073735E-03

 0
 0
 0
 0
 0
 0
 0
 0
 0
 0
 0
 0
 0
 0
 0
 0
 0
 0
 0
 0
 0
 0
 0
 0
 5.24160317073735E-03

 0
 0
 0
 0
 0
 0
 0
 0
 0
 0
 0
 0
 0
 0
 0
 0
 0
 0
 0
 0
 0
 0
 0
 0
 5.24160317073735E-03

 0
 0
 0
 0
 0
 0
 0
 0
 0
 0
 0
 0
 0
 0
 0
 0
 0
 0
 0
 0
 0
 0
 0
 0
 5.24160317073735E-03

 0
 0
 0
 0
 0
 0
 0
 0
 0
 0
 0
 0
 0
 0
 0
 0
 0
 0
 0
 0
 0
 0
 0
 0
 0
 7.17105119449449E-04

 0
 0
 0
 0
 0
 0
 0
 0
 0
 0
 0
 0
 0
 0
 0
 0
 0
 0
 0
 0
 0
 0
 0
 0
 0
 7.17105119449449E-04

 0
 0
 0
 0
 0
 0
 0
 0
 0
 0
 0
 0
 0
 0
 0
 0
 0
 0
 0
 0
 0
 0
 0
 0
 0
 7.17105119449449E-04

 0
 0
 0
 0
 0
 0
 0
 0
 0
 0
 0
 0
 0
 0
 0
 0
 0
 0
 0
 0
 0
 0
 0
 0
 0
 7.17105119449449E-04

 0
 0
 0
 0
 0
 0
 0
 0
 0
 0
 0
 0
 0
 0
 0
 0
 0
 0
 0
 0
 0
 0
 0
 0
 0
 7.17105119449449E-04

 0
 0
 0
 0
 0
 0
 0
 0
 0
 0
 0
 0
 0
 0
 0
 0
 0
 0
 0
 0
 0
 0
 0
 0
 0
 0
 2.02848010752918E-03

 0
 0
 0
 0
 0
 0
 0
 0
 0
 0
 0
 0
 0
 0
 0
 0
 0
 0
 0
 0
 0
 0
 0
 0
 0
 0
 2.02848010752918E-03

 0
 0
 0
 0
 0
 0
 0
 0
 0
 0
 0
 0
 0
 0
 0
 0
 0
 0
 0
 0
 0
 0
 0
 0
 0
 0
 2.02848010752918E-03

 0
 0
 0
 0
 0
 0
 0
 0
 0
 0
 0
 0
 0
 0
 0
 0
 0
 0
 0
 0
 0
 0
 0
 0
 0
 0
 2.02848010752918E-03

 0
 0
 0
 0
 0
 0
 0
 0
 0
 0
 0
 0
 0
 0
 0
 0
 0
 0
 0
 0
 0
 0
 0
 0
 0
 0
 2.02848010752918E-03

 0
 0
 0
 0
 0
 0
 0
 0
 0
 0
 0
 0
 0
 0
 0
 0
 0
 0
 0
 0
 0
 0
 0
 0
 0
 0
 0
 1.14686748316391E-02

 0
 0
 0
 0
 0
 0
 0
 0
 0
 0
 0
 0
 0
 0
 0
 0
 0
 0
 0
 0
 0
 0
 0
 0
 0
 0
 0
 1.14686748316391E-02

 0
 0
 0
 0
 0
 0
 0
 0
 0
 0
 0
 0
 0
 0
 0
 0
 0
 0
 0
 0
 0
 0
 0
 0
 0
 0
 0
 1.14686748316391E-02

 0
 0
 0
 0
 0
 0
 0
 0
 0
 0
 0
 0
 0
 0
 0
 0
 0
 0
 0
 0
 0
 0
 0
 0
 0
 0
 0
 1.14686748316391E-02

 0
 0
 0
 0
 0
 0
 0
 0
 0
 0
 0
 0
 0
 0
 0
 0
 0
 0
 0
 0
 0
 0
 0
 0
 0
 0
 0
 1.14686748316391E-02

 0
 0
 0
 0
 0
 0
 0
 0
 0
 0
 0
 0
 0
 0
 0
 0
 0
 0
 0
 0
 0
 0
 0
 0
 0
 0
 0
 0
 .106779649008027

 0
 0
 0
 0
 0
 0
 0
 0
 0
 0
 0
 0
 0
 0
 0
 0
 0
 0
 0
 0
 0
 0
 0
 0
 0
 0
 0
 0
 .106779649008027

 0
 0
 0
 0
 0
 0
 0
 0
 0
 0
 0
 0
 0
 0
 0
 0
 0
 0
 0
 0
 0
 0
 0
 0
 0
 0
 0
 0
 .106779649008027

 0
 0
 0
 0
 0
 0
 0
 0
 0
 0
 0
 0
 0
 0
 0
 0
 0
 0
 0
 0
 0
 0
 0
 0
 0
 0
 0
 0
 .106779649008027

 0
 0
 0
 0
 0
 0
 0
 0
 0
 0
 0
 0
 0
 0
 0
 0
 0
 0
 0
 0
 0
 0
 0
 0
 0
 0
 0
 0
 .106779649008027

 0
 0
 0
 0
 0
 0
 0
 0
 0
 0
 0
 0
 0
 0
 0
 0
 0
 0
 0
 0
 0
 0
 0
 0
 0
 0
 0
 0
 0
 0

 0
 0
 0
 0
 0
 0
 0
 0
 0
 0
 0
 0
 0
 0
 0
 0
 0
 0
 0
 0
 0
 0
 0
 0
 0
 0
 0
 0
 0
 0

 0
 0
 0
 0
 0
 0
 0
 0
 0
 0
 0
 0
 0
 0
 0
 0
 0
 0
 0
 0
 0
 0
 0
 0
 0
 0
 0
 0
 0
 0

 0
 0
 0
 0
 0
 0
 0
 0
 0
 0
 0
 0
 0
 0
 0
 0
 0
 0
 0
 0
 0
 0
 0
 0
 0
 0
 0
 0
 0
 0

 0
 0
 0
 0
 0
 0
 0
 0
 0
 0
 0
 0
 0
 0
 0
 0
 0
 0
 0
 0
 0
 0
 0
 0
 0
 0
 0
 0
 0
 0

 0
 0
 0
 0
 0
 0
 0
 0
 0
 0
 0
 0
 0
 0
 0
 0
 0
 0
 0
 0
 0
 0
 0
 0
 0
 0
 0
 0
 0
 0
 6.38229260080273E-04

 0
 0
 0
 0
 0
 0
 0
 0
 0
 0
 0
 0
 0
 0
 0
 0
 0
 0
 0
 0
 0
 0
 0
 0
 0
 0
 0
 0
 0
 0
 6.38229260080273E-04

 0
 0
 0
 0
 0
 0
 0
 0
 0
 0
 0
 0
 0
 0
 0
 0
 0
 0
 0
 0
 0
 0
 0
 0
 0
 0
 0
 0
 0
 0
 6.38229260080273E-04

 0
 0
 0
 0
 0
 0
 0
 0
 0
 0
 0
 0
 0
 0
 0
 0
 0
 0
 0
 0
 0
 0
 0
 0
 0
 0
 0
 0
 0
 0
 6.38229260080273E-04

 0
 0
 0
 0
 0
 0
 0
 0
 0
 0
 0
 0
 0
 0
 0
 0
 0
 0
 0
 0
 0
 0
 0
 0
 0
 0
 0
 0
 0
 0
 6.38229260080273E-04

 0
 0
 0
 0
 0
 0
 0
 0
 0
 0
 0
 0
 0
 0
 0
 0
 0
 0
 0
 0
 0
 0
 0
 0
 0
 0
 0
 0
 0
 0
 0
 4.33593842145119E-04

 0
 0
 0
 0
 0
 0
 0
 0
 0
 0
 0
 0
 0
 0
 0
 0
 0
 0
 0
 0
 0
 0
 0
 0
 0
 0
 0
 0
 0
 0
 0
 4.33593842145119E-04

 0
 0
 0
 0
 0
 0
 0
 0
 0
 0
 0
 0
 0
 0
 0
 0
 0
 0
 0
 0
 0
 0
 0
 0
 0
 0
 0
 0
 0
 0
 0
 4.33593842145119E-04

 0
 0
 0
 0
 0
 0
 0
 0
 0
 0
 0
 0
 0
 0
 0
 0
 0
 0
 0
 0
 0
 0
 0
 0
 0
 0
 0
 0
 0
 0
 0
 4.33593842145119E-04

 0
 0
 0
 0
 0
 0
 0
 0
 0
 0
 0
 0
 0
 0
 0
 0
 0
 0
 0
 0
 0
 0
 0
 0
 0
 0
 0
 0
 0
 0
 0
 4.33593842145119E-04
