# Supplementary material for: Ammonia Inhibition of Anaerobic Volatile Fatty Acid Degrading Microbial Communities
Source: Front Microbiol. 2018 Nov 30;9:2921. doi: 10.3389/fmicb.2018.02921 (PMC6284035; doi:10.3389/fmicb.2018.02921)
Supplement: Supplementary file 1 [file Data_Sheet_1.zip › Supplementary_Material A.pdf]

Supplementary Material A

Ammonia inhibition of anaerobic volatile fatty acid degrading microbial communities

Fabian Bonk, Denny Popp, Sören Weinrich, Heike Sträuber, Sabine Kleinsteuber, Hauke Harms, Florian Centler*

*** Correspondence:** Corresponding Author: florian.centler@ufz.de

1 Medium composition

Table S1 Composition of mineral medium

Compound	Concentration in partitioned media (mg L^{-1})	Final concentration in CSTR influent (mg L^{-1})
**component A**		
Acetic acid	31,420	15,710
Propionic acid	4,920	2,460
Butyric acid	18,440	9,220
KCl	600	300
$MgCl_2 \times 6H_2O$	600	300
$CaCl_2 \times 2H_2O$	200	100
$Na_2S \times 9H_2O$	500	250
$FeCl_2 \times 4H_2O$	42.36	21.18
$CuCl_2 \times 2H_2O$	0.86	0.43
$CoCl_2 \times 6H_2O$	1.94	0.97
$MnCl_2 \times 4H_2O$	1.64	0.82
$Na_2MoO_4 \times 2H_2O$	0.86	0.43
$NiCl_2 \times 6H_2O$	3.28	1.64
$Na_2WO_4 \times 2H_2O$	0.36	0.18
$Na_2SeO_3 \times 5H_2O$	0.8	0.40
$ZnCl_2$	4.72	2.36
H_3BO_3	4.96	2.48

Biotin	0.04	0.02
Folic acid	0.04	0.02
Pyridoxine	0.2	0.1
Thiamine	0.1	0.05
Riboflavin	0.1	0.05
Nicotinic acid	0.1	0.05
Ca-Pantothenate	0.1	0.05
B12	0.1	0.05
p-Aminobenzoate	0.1	0.05
Lipoic acid	0.1	0.05
**component B**		
KH_2PO_4	1000	500
NH_4HCO_3	8,190 (10,949 starting day 21)	4,095 (21,899 starting day 21)
NaOH	5,600	2,800

2 Microscopy images of aggregates

After the increase of ammonia concentration, R_{NH3} and $R_{NH3,HCl}$ started to show aggregates, which were not observed before day 21 and not observed for R_{ctrl} throughout the experiment. The aggregates were examined in an AZ100M microscope (Nikon, Germany, Figure S1A) and by epifluorescence microscopy (excitation 395-440 nm, emission 470 nm) using an Axioplan2 (Zeiss, Germany, Figure S1B+C). Aggregates were up to 1 mm in width and showed autofluorescence (Figure S1). Most likely they contained *Methanosarcina* cells, which are known to form aggregates (De Vrieze et al., 2012) and to show autofluorescence at 420 nm excitation (Demirel and Scherer, 2008).

(A)

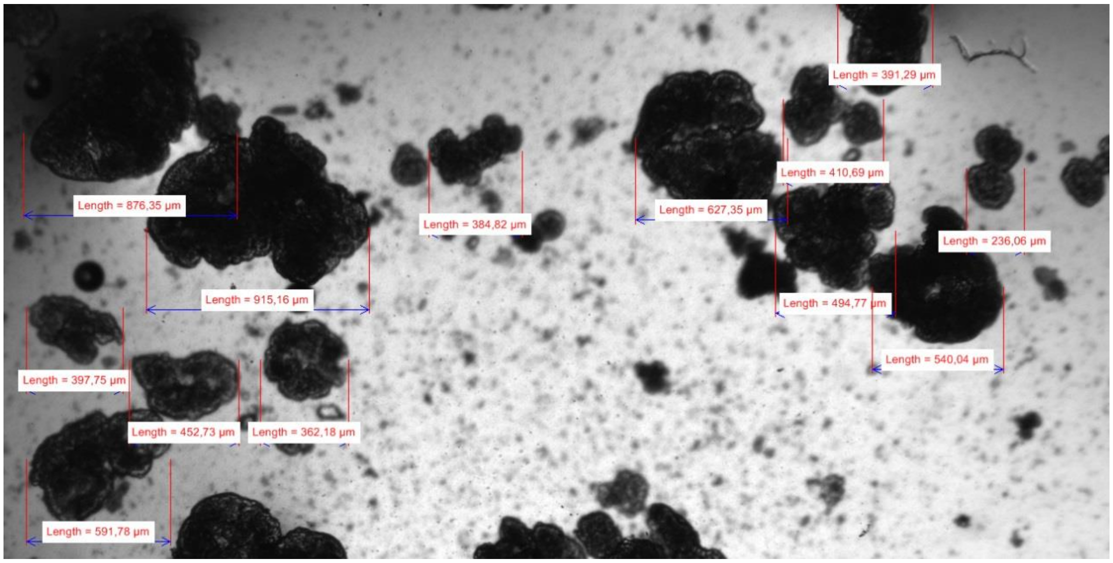

(B) (C)

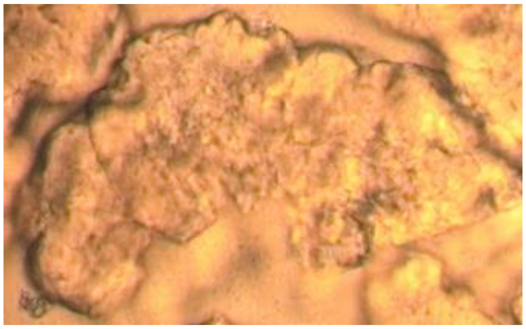

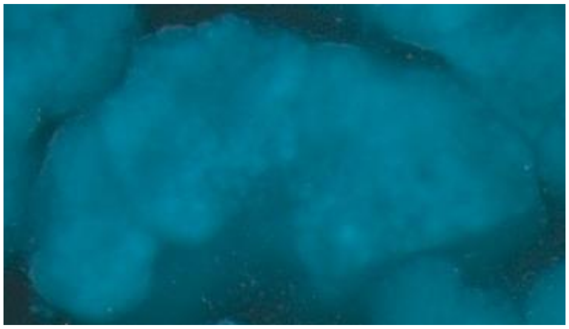

Figure S1 Microscopic picture of aggregates in R_{NH3} at day 52. (A) and (B) light microscopy. (C) epifluorescence microscopy (excitation 395-440 nm, emission 470 nm).

3 Additional experiments

In addition to the experiment presented in the manuscript (Experiment 1), three additional experiments were conducted (Experiments 2-4, see Table S2). In general, the same medium as described above was used in all experiments, except for the absolute VFA, the ammonium bicarbonate and the chloride concentration. The microbial community in Experiment 2 was subjected to ammonia inhibition from the start of the experiment. A further disturbance was induced in this experiment by decreasing the HCl concentration in the influent on day 46. Experiment 3 started with a 73 d long acclimatization phase to a low ammonia concentration of 51.8 mM before the microbial communities were subjected to ammonia inhibition. In this experiment, continuous feeding (R_{conti}) was compared to discontinuous feeding (R_{disco}). Discontinuous feeding meant that 75% of the daily feed was added to the reactor in the course of 20 minutes while the other 25% were continuously fed. Discontinuous feeding was stopped on day 108 and switched to continuous feeding and higher ammonia as well as VFA influent concentrations (Experiment 4). All reactors were inoculated from a reactor running at a VFA influent concentration of 12.4 gCOD L^{-1} and an ammonium bicarbonate influent concentration of 51.8 mM, except Experiment 4 which used the reactor content of R_{disco} at day 108 in Experiment 3 as inoculum. The bacterial communities were monitored by 16S rRNA gene amplicon sequencing as described in the Method section. However, more stringent quality filtering was applied (only reads with quality score of 30 or higher were retained). The OTU table was rarefied to 11,074 sequences per sample. Bacterial community compositions were visualized as Krona plots, see Supplementary Material C.

Table S2 Experimental set-up of additional experiments

**Time (d)**	**Influent concentration**			**Comment**
	**VFA (gCOD L^{-1})**	**NH_4HCO_3 (mM)**	**Chloride (mM)**	
**Experiment 2:** HRT=8 d, V_w=8 L, continuous feeding				
0-4	18.6	444	208	
4-7	24.8	444	208	
7-11	31	444	208	
11-46	37.2	444	208	
46-103	37.2	444	8.6	
103	37.2	444	291	
104	37.2	444	754	
105	37.2	444	767	
106-109	37.2	444	396	
109-113	37.2	444	8.6	
**Experiment 3**: continuous (R_{conti}) and discontinuous feeding (R_{disco}), HRT=8 d; V_w=8 L				
0-73	12.4	51.8	8.6	Day 73: 414 mmol NH_4HCO_3 directly added to reactors
73-108	12.4	277	8.6	
108-157 only R_{conti}	12.4	277	100	R_{disco} continued as Experiment 4
**Experiment 4**: HRT=8 d, V_w=8 L, continuous feeding				
0-49	24.8	444	8.6	

4 Results of additional experiments

4.1 Process performance

4.1.1 VFA degradation efficiencies Experiment 2-4

VFA degradation efficiencies were calculated for Experiment 2-4 in the same manner as for Experiment 1. As in Experiment 1, propionate and acetate degradation were inhibited most (Figure S2).

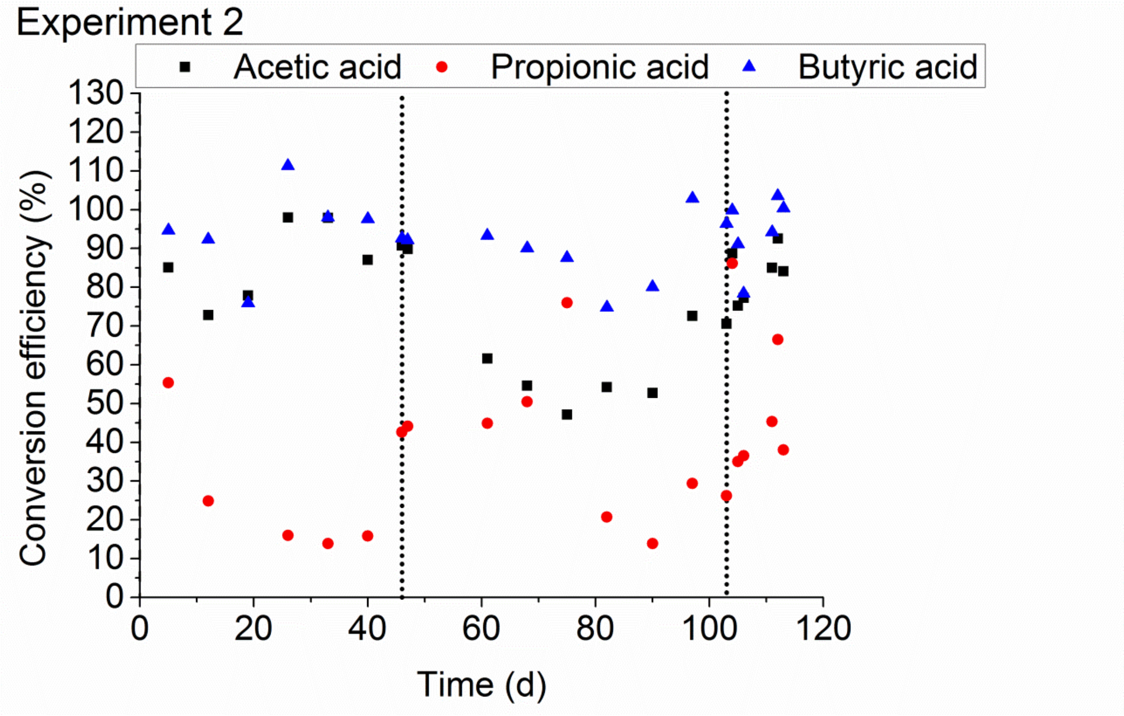

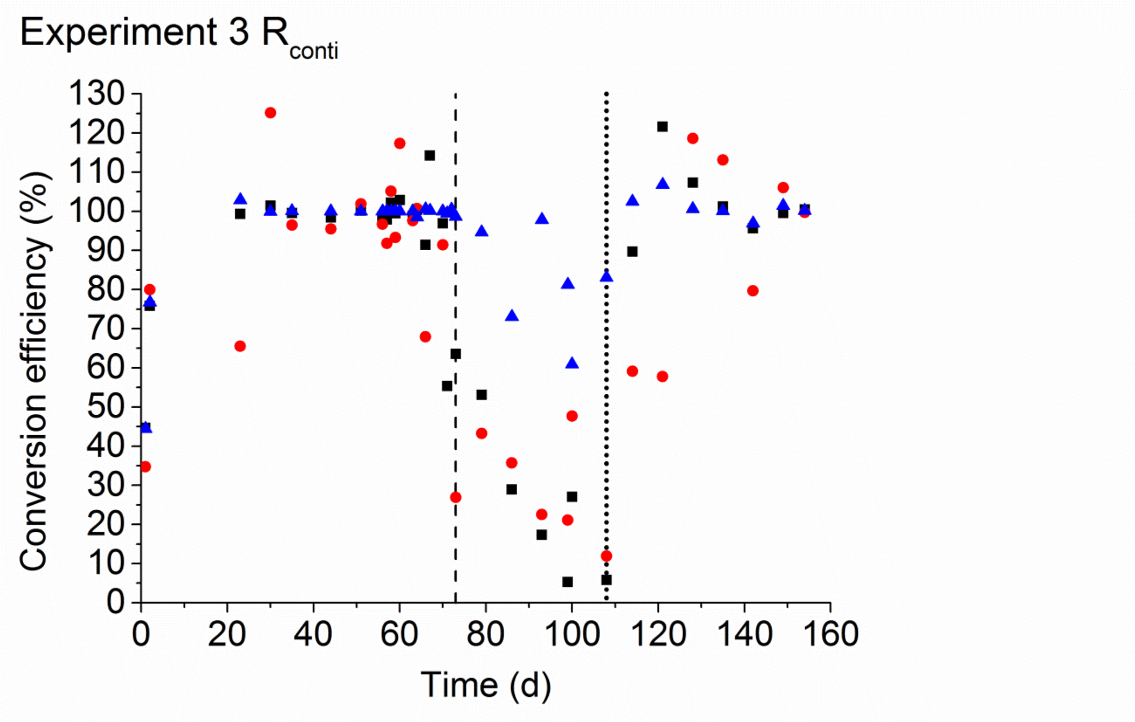
Experiment 3 Rconti
Conversion efficiency (%)
130
120
110
100
90
80
70
60
50
40
30
20
10
0
0
20
40
60
80
100
120
140
160
Time (d)

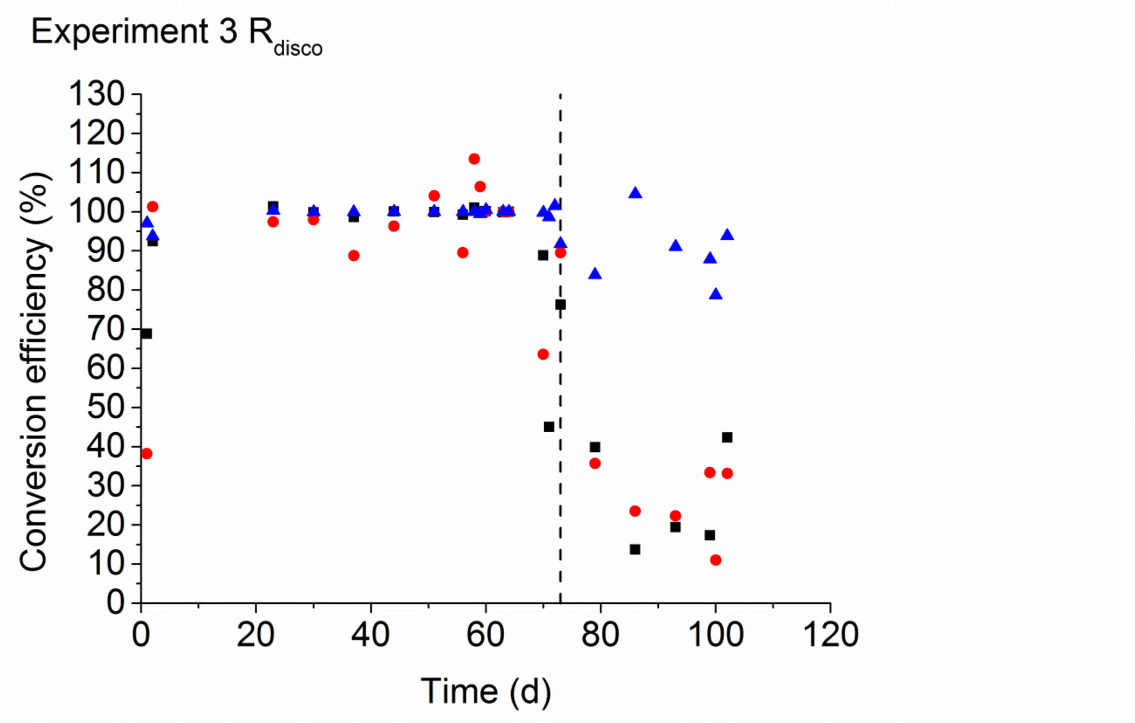
Experiment 3 Rdisco
Conversion efficiency (%)
130
120
110
100
90
80
70
60
50
40
30
20
10
0
0
20
40
60
80
100
120
Time (d)

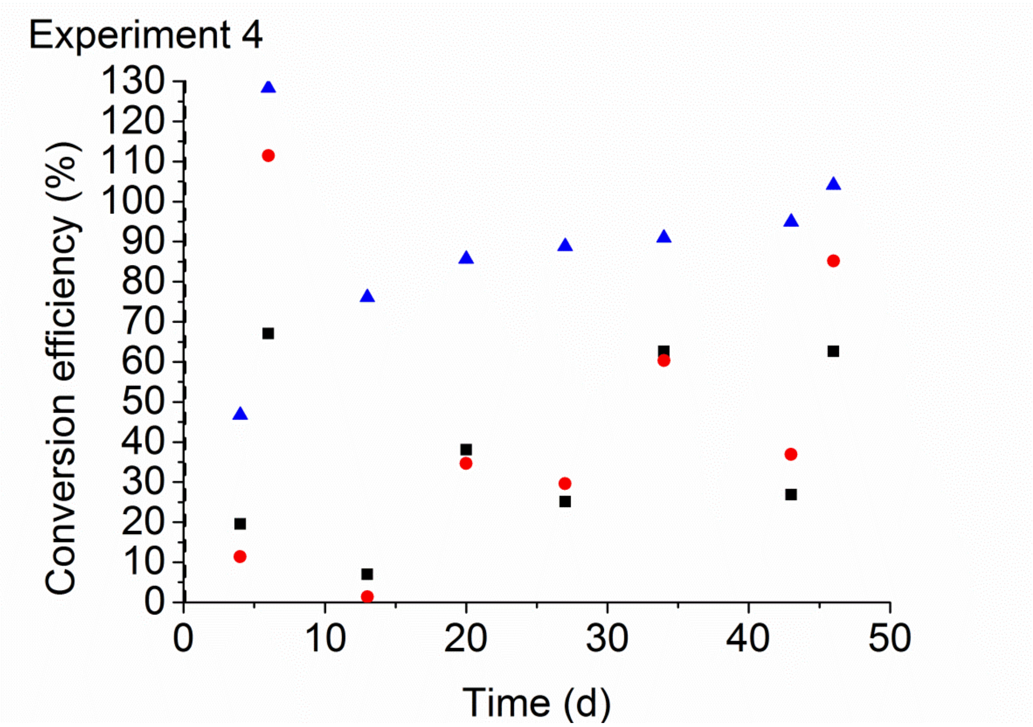

Figure S2 VFA degradation efficiencies of additional experiments (Experiments 2-4). Dotted lines mark a change in HCl concentration in the influent. Dashed lines mark a change in ammonia concentration in the influent (see Table S2).

4.2 Methanogenic community analysis

The methanogenic community compositions for Experiment 2, Experiment 3 and Experiment 4 are shown in Figures S3, S4, and S5, respectively.

4.2.1 Experiment 2 DNA

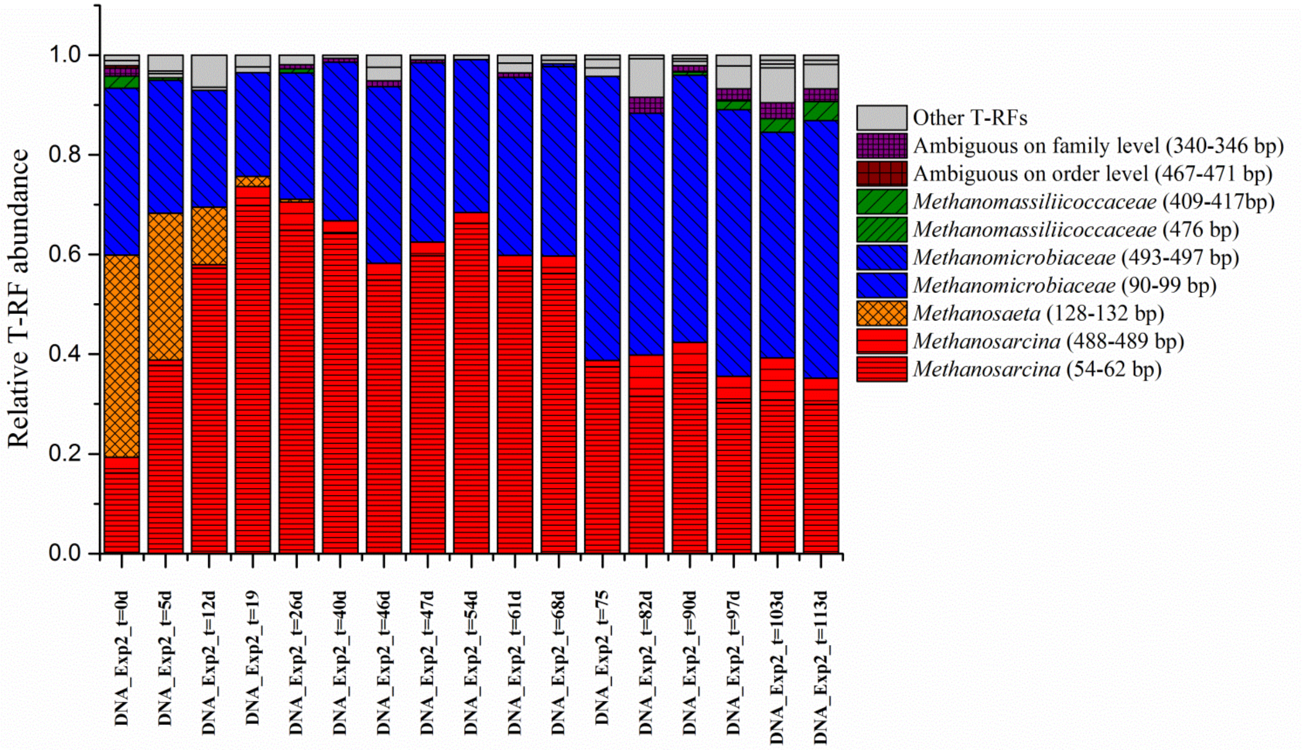

Figure S3 Methanogenic community analysis by T-RFLP of the *mcrA* gene using *Bst*NI as restriction enzyme. T-RFs with a length of 340-346 bp can be *Methanocorpusculaceae, Methanomicrobiaceae*, *Methanoregulaceae*, and/or *Methanospirillacea*e. T-RFs with a length of 467-471 bp can be *Methanobacteriales*, *Methanococcales*, and/or *Methanomassiliicoccales*.

4.2.2 Experiment 3 DNA

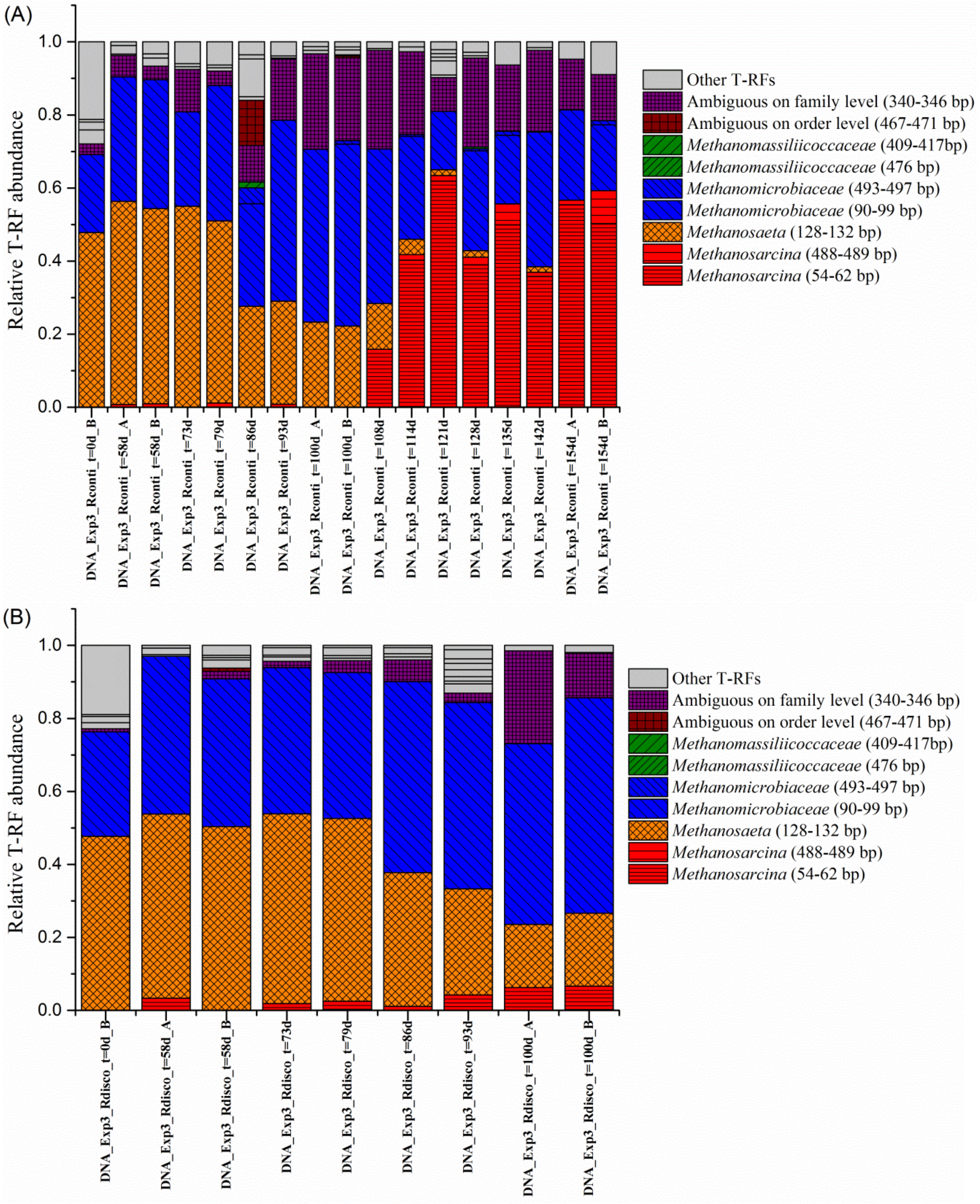

Figure S4 Methanogenic community analysis by T-RFLP of the *mcrA* gene using *Bst*NI as restriction enzyme. (A) continuously fed reactor R_{conti}, (B) discontinuously fed reactor R_{disco}. T-RFs with a length of 340-346 bp can be *Methanocorpusculaceae*, *Methanomicrobiaceae*, *Methanoregulaceae*, and/or *Methanospirillaceae*. T-RFs with a length of 467-471 bp can be *Methanobacteriales*, *Methanococcales*, and/or *Methanomassiliicoccales*. A and B indicate technical replicates.

4.2.3 Experiment 4 DNA

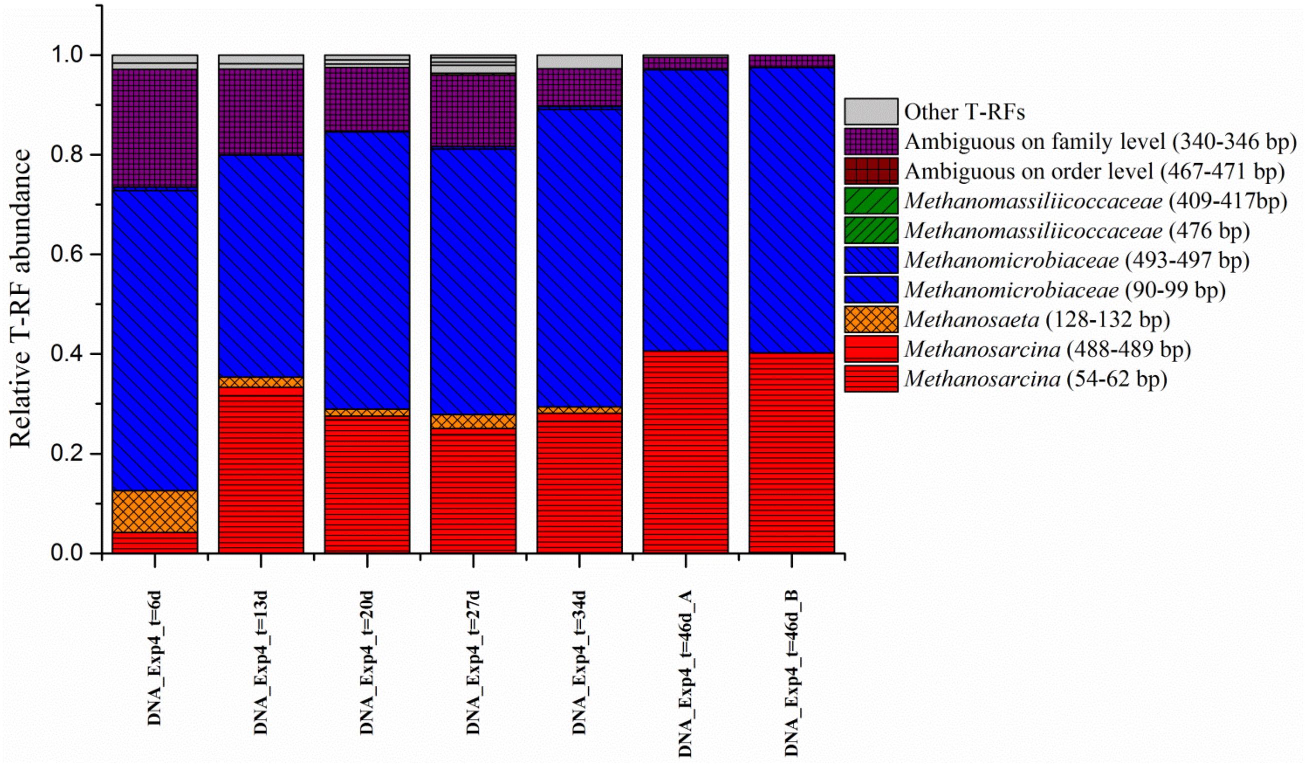

Figure S5 Methanogenic community analysis by T-RFLP of the *mcrA* gene using *Bst*NI as restriction enzyme. T-RFs with a length of 340-346 bp can be *Methanocorpusculaceae, Methanomicrobiaceae, Methanoregulaceae*, and/or *Methanospirillacea*. T-RFs with a length of 467-471 bp can be *Methanobacteriales, Methanococcales*, and/or *Methanomassiliicoccales*.

5 Additional remarks to multi-taxa extension of ADM1

5.1 Implementation of two competing propionic acid oxidizers in ADM1

The propionic acid oxidizers (X_{pro}) were replaced by two competing populations: an unknown taxon ($X_{pro,1}$, see Equation S1a) and *Syntrophobacter* ($X_{pro,2}$, see Equation S1b). Accordingly, the differential equation for propionic acid was adapted to Equation S1c. The original ADM1 inhibition functions (Batstone et al., 2002) were used with different parameter values for $X_{pro,1}$ and $X_{pro,2}$ concerning the ammonia inhibition function (Equation S1d).

$$\frac{dX_{pro,1}}{dt} = \frac{1}{HRT} \cdot (X_{in,pro,1} - X_{pro,1}) + (Y_{pro,1} \cdot k_{m,pro,1} \cdot \frac{S_{pro}}{S_{pro}+K_{S,pro,1}} \cdot I_{pro,1} - k_{dec}) \cdot X_{pro,1}$$ (Equation S1a)

$$\frac{dX_{pro,2}}{dt} = \frac{1}{HRT} \cdot (X_{in,pro,2} - X_{pro,2}) + (Y_{pro,2} \cdot k_{m,pro,2} \cdot \frac{S_{pro}}{S_{pro}+K_{S,pro,2}} \cdot I_{pro,2} - k_{dec}) \cdot X_{pro,2}$$ (Equation S1b)

$$\frac{dS_{pro}}{dt} = \frac{1}{HRT} \cdot (S_{pro,in} - S_{pro}) - k_{m,pro,1} \cdot \frac{S_{pro}}{S_{pro}+K_{S,pro,1}} \cdot I_{pro,1} \cdot X_{pro,1} - k_{m,pro,2} \cdot \frac{S_{pro}}{S_{pro}+K_{S,pro,2}} \cdot I_{pro,2} \cdot X_{pro,2} + \upsilon_{6,5} \cdot \rho_5 + \upsilon_{6,6} \cdot \rho_6 + \upsilon_{6,8} \cdot \rho_8$$ (Equation S1c)

$$I_{nh3,i} = \frac{1}{1+\frac{S_{nh3}}{K_{I,nh3,i}}}$$ (Equation S1d)

with Y being the biomass yield, k_m the maximum substrate uptake rate, K_S the half saturation constant, k_{dec} the decay rate, I the inhibition functions, S the substrate concentration, *HRT* the hydraulic retention time, *pro* the index for propionic acid, *pro1* the index for an unknown taxon, *pro2* the index for *Syntrophobacter*, $\upsilon_{6,5} \cdot \rho_5 + \upsilon_{6,6} \cdot \rho_6 + \upsilon_{6,8} \cdot \rho_8$ the propionic acid producing reactions (see Peterson Matrix, Supplementary Materials B), $I_{nh3,i}$ the inhibition ammonia inhibition function, S_{nh3} the ammonia concentration and $K_{I,nh3,i}$ the empirical ammonia inhibition parameter with different values for the unknown taxon (*i=pro,1*) and *Syntrophobacter* (*i=pro,2*).

5.2 Local sensitivity analysis

The parameter values inferred in the ADM1 simulations (see Table 1 of the manuscript) were varied one at a time (OAT) by up to ± 100% to test the local sensitivity of each parameter. The coefficients of determination of simulated and experimental results were calculated for either acetic acid concentrations (Figure S6a) or propionic acid concentrations (Figure S6b).

For both acetoclastic methanogens (Figure S6a) and propionic acid oxidizing bacteria (Figure S6b), changing the ammonia inhibition parameter ($K_{I,nh3,ac1}$ and $K_{I,nh3,pro1}$) values to higher values (less inhibition) had no impact on the ammonia resistant taxa $X_{ac,1}$ and $X_{pro,1}$. This makes sense, because the ammonia concentrations in our experiment are not inhibitory for $X_{ac,1}$ and $X_{pro,1}$. Therefore, a broad range of parameter values fit to the experimental data. Thus, in future pure or defined co-culture experiments, higher ammonia concentrations than 277 mM would need to be applied to accurately determine the ammonia inhibition parameter values for the taxa present in our experiment.

The initial biomass concentrations for $X_{ac,1}$ (t=0) and $X_{pro,1}$ (t=0) were sensitive towards both lower and higher values. At higher initial concentrations, their abundance increases too fast in the simulations which lead to lower VFA accumulations than observed. For too small initial concentrations, acclimatization in the simulations will take longer than observed. Fitting the initial concentrations may become obsolete in the future as metaproteomics based techniques are currently under development to determine taxon-specific microbial protein concentrations as estimator for biomass concentrations (Kleiner et al., 2017).

The simulations were very sensitive to both lower and higher values of the half saturation constant ($K_{S,ac1}$ and $K_{S,pro1}$) for $X_{ac,1}$ and $X_{pro,1}$, because at lower values, these taxa would be too competitive at low ammonia concentrations and at higher values, VFA concentrations would slowly or never decrease to the observed values. The higher r^2 value for K_S ($X_{pro,1}$) at -100% is an artefact because a half-saturation constant value of 0 M makes no sense from a physiological point of view, because it would mean that this taxon could grow at maximum rate independent of the substrate concentration.

The simulations were very sensitive to both lower and higher values of the ammonia inhibition parameter ($K_{I,nh3,ac2}$ and $K_{I,nh3,pro2}$) for the ammonia sensitive taxa $X_{ac,2}$ and $X_{pro,2}$. This makes sense, because at lower values (higher inhibition), these taxa would already be washed out under the lower ammonia concentrations at the start of the experiment. At higher values (lower inhibition), they would not be washed out as fast as observed and as a consequence, VFA accumulation would be lower than observed.

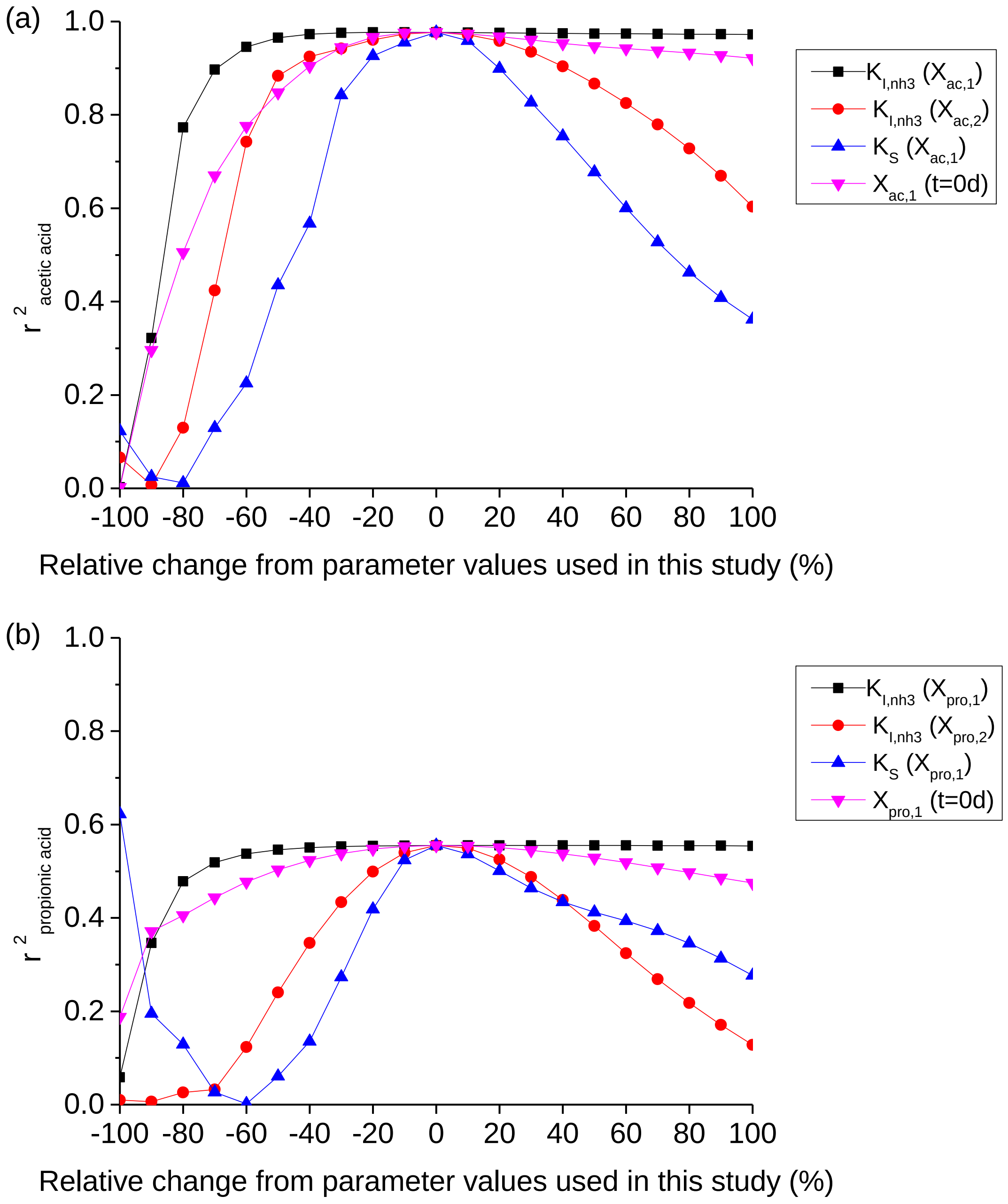

Figure S6 Sensitivity analysis on the parameter values inferred in this study for the ADM1 simulations. (a) Coefficient of determination of simulated and experimental acetic acid concentrations. $X_{ac,1}$ = *Methanosarcina*, $X_{ac,2}$=*Methanosaeta*. (b) Coefficient of determination of simulated and experimental propionic acid concentration. Propionic acid oxidizing bacteria: $X_{pro,1}$ = (unknown taxon), $X_{pro,2}$ (*Syntrophobacter*).

References

De Vrieze, J., Hennebel, T., Boon, N., and Verstraete, W. (2012). *Methanosarcina*: The rediscovered methanogen for heavy duty biomethanation. *Bioresour. Technol.* 112, 1–9. doi:10.1016/j.biortech.2012.02.079.

Demirel, B., and Scherer, P. (2008). The roles of acetotrophic and hydrogenotrophic methanogens during anaerobic conversion of biomass to methane: A review. *Rev. Environ. Sci. Biotechnol.* 7, 173–190. doi:10.1007/s11157-008-9131-1.

Kleiner, M., Thorson, E., Sharp, C. E., Dong, X., Liu, D., Li, C., et al. (2017). Assessing species biomass contributions in microbial communities via metaproteomics. *Nat. Commun.* 8, 1558. doi:10.1038/s41467-017-01544-x.
